# Continuous Flow Electroselenocyclization of Allylamides and Unsaturated Oximes to Selenofunctionalized Oxazolines and Isoxazolines

**DOI:** 10.1021/acsorginorgau.4c00008

**Published:** 2024-03-11

**Authors:** Ohud Alzaidi, Thomas Wirth

**Affiliations:** † School of Chemistry, Cardiff University, Park Place, Main Building, Cardiff CF10 3AT, U.K.; ‡ Department of Chemistry, College of Science – Al Khurma, Taif University, P.O. Box 11099, Taif 21944, Saudi Arabia

**Keywords:** electrosynthesis, selenylation, heterocycles, cyclization, flow chemistry

## Abstract

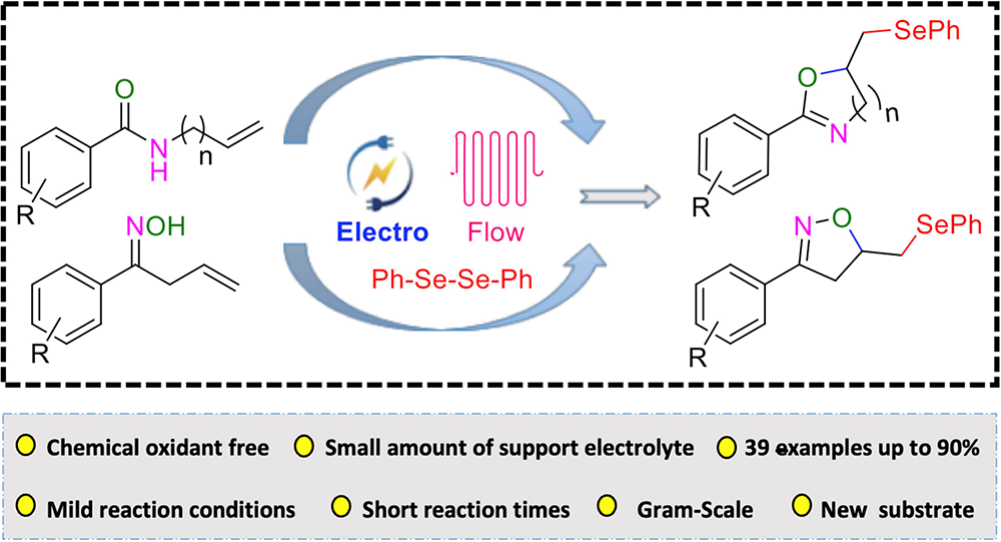

The synthesis of selenofunctionalized oxazolines and isoxazolines from *N*-allyl benzamides and unsaturated oximes with diselenides was studied by utilizing a continuous flow electrochemical approach. At mild reaction conditions and short reaction times of 10 min product yields of up to 90% were achieved including a scale-up reaction. A broad substrate scope was studied and the reaction was shown to have a wide functional group tolerance.

## Introduction

**Figure 1:**
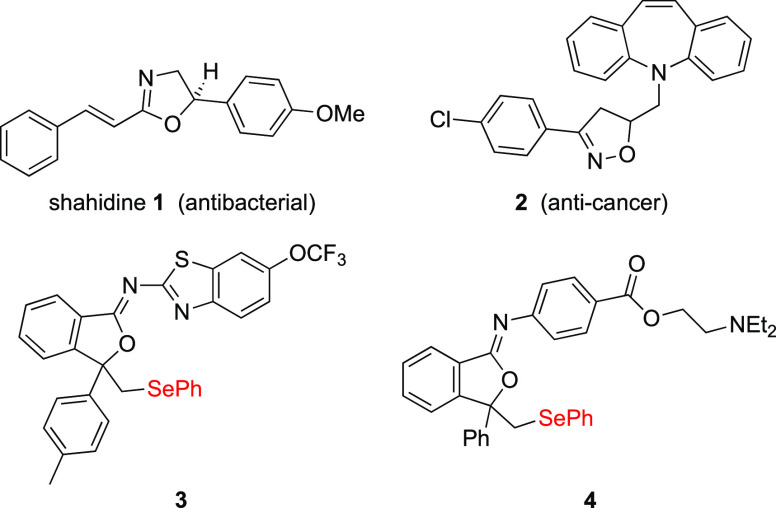
Examples of biologically active heterocycles and heterocyclic selenium-containing compounds.

*N*-Heterocyclic compounds, especially oxazolines and isoxazolines, are attractive synthetic targets because of their pharmacological and biological activities,^1−3^ and additionally because of their high value as valuable synthetic building blocks. Oxazolines and isoxazolines are found in biologically active products, for example in shahidine **1**(4) which is a strong antibacterial reagent, and dibenzoazepine **2**(5) which has anticancer properties (Figure 1). Because of their importance, a lot of progress has been made recently to develop suitable approaches for the production of these five-membered heterocycles.^6−9^ Various successful methods for the synthesis of heterocyclic systems have been published in the last decades.^10,11^

Oxidative cyclization of *N*-allyl benzamides and unsaturated oximes has emerged as an alternative method for producing a wide range of functionalized oxazolines and isoxazolines.^12−14^ Despite the significant progress in this area, reducing the amount of oxidant, chemical additives, or transition metals has been needed in the synthesis as the mentioned earlier processes are impractical, especially in industrial processes. As a result, more effective methodologies for producing oxazole and isoxazole derivatives remain in high demand.

Organoselenium compounds have gained interest as reagents and catalysts due to their applications in medicinal and material science,^15^ especially selenylative heterocyclization resulting in modified drugs,^16^ such as **3** derived from riluzole and **4** from procaine (Figure 1).

**Scheme 1:**
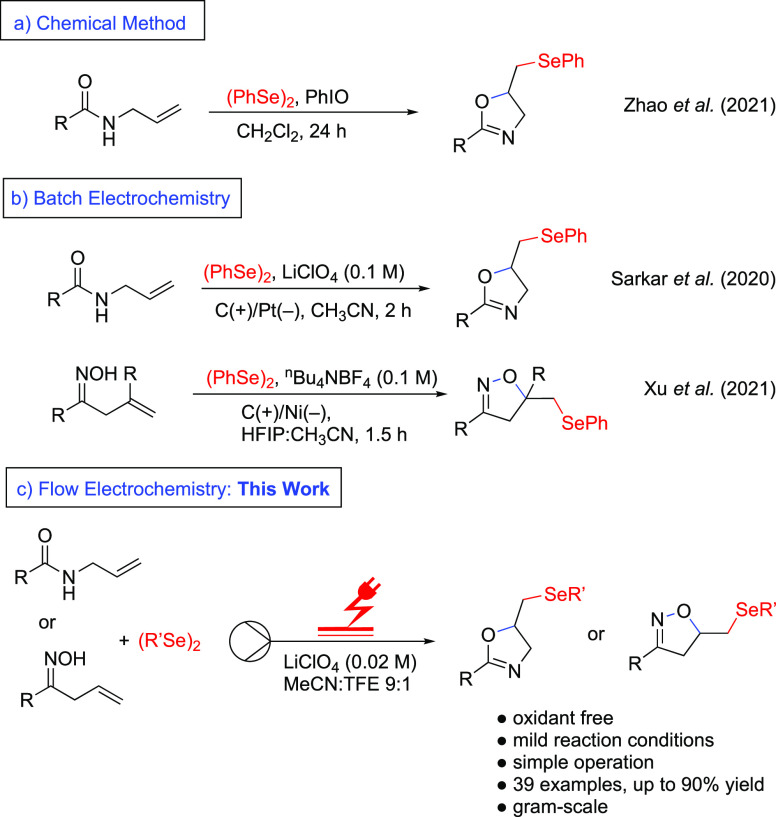
Electroselenocyclization of *N*-Allyl Amides and Unsaturated Oximes

Such selenocyclizations are typically performed by different Lewis acids^17^ or Bro̷nsted acids.^18^ Another approach to selenylative cyclizations is using transition metal catalysis.^19^ Recently, Zhao et al. reported that hypervalent iodine reagents were used as oxidants to produce selenomethyl-substituted heterocycles (Scheme 1a).^20^ However, the requirement for stoichiometric oxidants, expensive reagents, hazardous chlorinated solvents, and long reaction times in these reactions is not environmentally friendly and should be improved upon.

To foster a sustainable methodology, batch electrochemical synthesis has been investigated to develop a sustainable approach that is less costly.^21−24^ The most remarkable aspect of electrochemistry is the utilization of electrons as oxidizing or reducing reagents, which eliminates the use of transition-metal catalysts or hazardous reagents in redox reactions. We have previously examined many various aspects of selenium chemistry, such as some batch electrochemical reactions to form carbon–selenium bonds.^25^ Although the bioactivity of organoselenides is extensively known, the production of C–Se bonds has been significantly less investigated. The electrochemical selenylation of terminal alkenes is a very promising approach as shown by Sarkar and co-workers^26^ and Xu and co-workers^27^ (Scheme 1b). These procedures are efficient, but effort is still necessary to generate effective and reliable reaction conditions with a small amount of supporting electrolytes. In this context, we present the use of continuous flow electrochemistry for the selenocyclization of *N*-allyl benzamides and unsaturated oximes to form selenofunctionalized oxazolines and isoxazolines. This method is shown to have a wide functional group tolerance and is easily scalable (Scheme 1c).

## Results and Discussion

The electrolysis was performed in an undivided cell using an ion electrochemical flow reactor (reactor volume 0.6 mL, spacer 0.5 mm)^28^ with each electrode in the reactor possessing an active surface area of 12 cm^2^. The initial phase of our investigations involved utilizing *N*-allyl benzamide **5a** as the substrate to optimize the conditions for the synthesis of selenylated oxazoline **6a** (Table 1). Subsequently, we systematically explored electrolysis parameters by varying electrode materials, solvent systems, flow rates, and current density (see the Supporting Information).

**Table 1:**
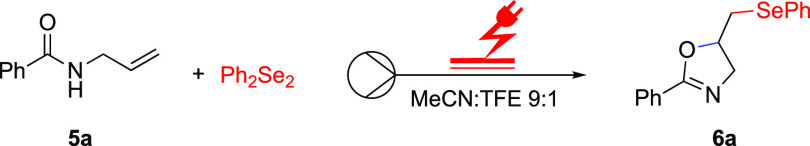
Optimization of the Electrosynthetic Oxidative Selenocyclization of *N*-Allyl Benzamides^a^

entry	[**5a**] (M)	Ph_2_Se_2_ (M)	cathode material	flow rate (mL/min)	*Q* (F)	*I* (mA)	**6a** (%)^b^
1	0.05	0.04	Gr	0.15	2.25	27	70
2	0.05	0.04	Gr	0.15	2.5	30	73
3	0.05	0.04	Gr	0.15	3	36	89
**4**	**0.05**	**0.04**	**Gr**	**0.15**	**3.5**	**42**	**93**
5	0.05	0.04	Pt	0.15	3.5	42	73
6	0.05	0.04	GC	0.15	3.5	42	70
7	0.05	0.04	SS	0.15	3.5	42	40
8	0.075	0.06	Gr	0.15	3.5	63	58
9	0.1	0.08	Gr	0.15	3.5	84	41
10	0.025	0.04	Gr	0.15	3.5	21	78
11	0.05	0.04	Gr	0.1	3.5	42	69
12	0.05	0.04	Gr	0.2	3.5	42	70

a Standard reaction conditions: undivided flow cell, Gr electrodes (active surface area: 12 cm^2^), interelectrode distance: 0.5 mm, **5a** (0.05 M, 0.5 mmol), Ph_2_Se_2_ (0.04 M), LiClO_4_ (0.02 M) dissolved in a mixture of MeCN and TFE (9:1 v/v).

b Yield determined by ^1^H NMR using 1,3,5-trimethoxybenzene as internal standard. TFE: 2,2,2-trifluoroethanol.

Initially, with graphite electrodes as both the anode and cathode, a flow rate of 0.15 mL min^–1^, and an applied charge of 2.25 F, we achieved the desired product **6a** in 70% yield (entry 1, Table 1). While the two-electron oxidation theoretically requires only 2.0 F, it was observed that increasing the charge to 2.5 and 3 F resulted in yield improvements to 73 and 89%, respectively (entries 2–3, Table 1). Further increase of the charge to 3.5 F demonstrated a yield increase of 93% (entry 4, Table 1).

Various cathodic electrode materials were tested, yielding 73% (Pt), 70% (GC), and 40% (SS) yields of the desired product, respectively (entries 5–7, Table 1). Additionally, diverse anodic materials were explored (see Supporting Information), revealing that graphite electrode was more effective as the anode compared to platinum. Varying the concentration of *N*-allyl benzamide **5a**, a significant decrease in the yield of **6a** upon increasing the concentration from 0.05 to 0.075 and 0.1 M was observed (entries 8–9, Table 1). Conversely, reducing the concentration to 0.025 M resulted in a decrease in the observed yield (entry 10, Table 1). To identify the optimal flow rate for overcoming mass-transfer constraints, we examined the impact of flow rate/residence time on product yield.^29^ Increasing the flow rate to 0.2 mL min^–1^ led to a decrease in yield (entry 12, Table 1), potentially attributed to a decreased reaction time at higher flow rates. Various solvents and solvent mixtures, including acetonitrile, 1,1,1,3,3,3-hexafluoro-2-propanol, methanol, and acetonitrile/2,2,2-trifluoroethanol, were screened, and acetonitrile/2,2,2-trifluoroethanol emerged as the most suitable solvent for this reaction (see Supporting Information). In 2021, Xu and coauthors reported a selenocyclization using unsaturated oximes in a batch electrochemical operation that used a stoichiometric amount of tetrabutylammonium tetrafluoroborate (Bu_4_NBF_4_) as a supporting electrolyte.^27^ We observed that under flow conditions, only small amounts of LiClO_4_ were required to achieve the results in much shorter reaction times; however, without the addition of electrolyte, the reaction did not occur in the electrochemical flow reactor. The presence of an electrolyte has an important effect on the yield (see Supporting Information).

**Scheme 2:**
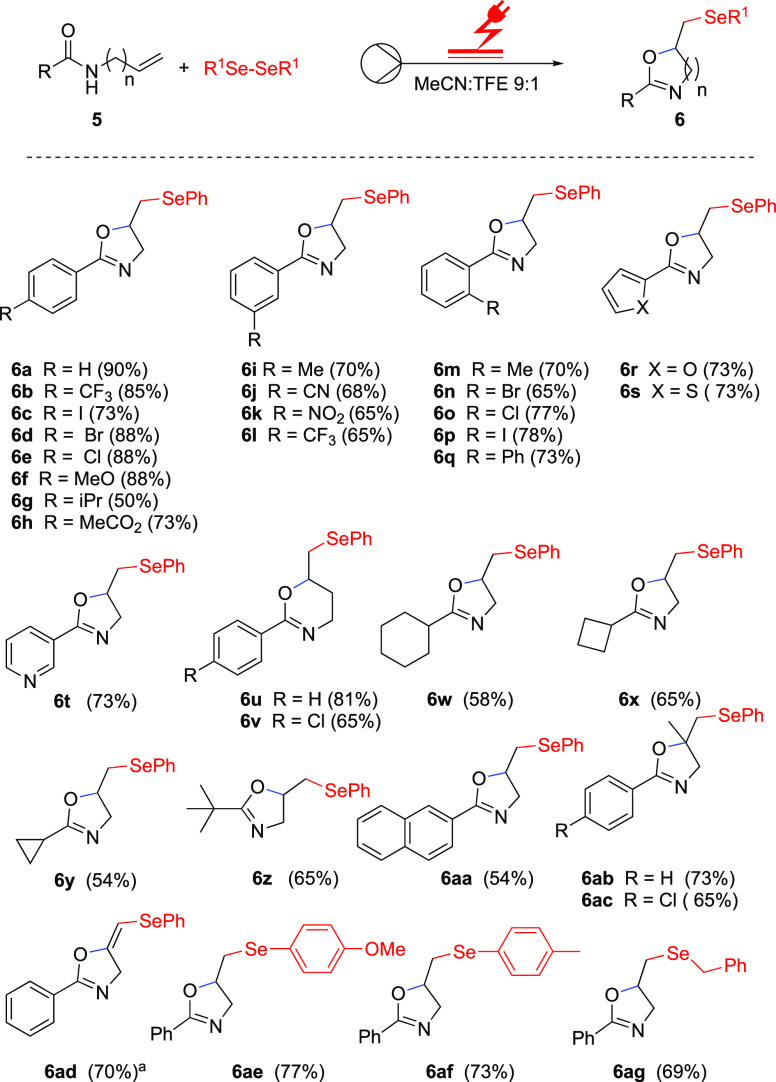
Substrate Scope of the Electrochemical Selenocyclization of Allyl and Homoallyl Benzamides **5** in Flow

With the optimized reaction conditions in hand, most of the investigated substrates have been converted to the corresponding products in good to excellent yield, demonstrating good functional group tolerance (Scheme 2). Starting from *N*-allylbenzamide **5a**, 2-phenyl-5-((phenylselanyl)methyl)-4,5 dihydrooxazole **6a** was obtained in 90% yield under the optimal reaction conditions. Several *para*-substituted derivatives (**5b**–**h**) were effectively transformed to the corresponding seleno oxazoline derivatives (**6b**–**h**) in moderate to excellent yields. Products with electron-withdrawing groups at the *para*-position (**6b**–**e**) were obtained in excellent yields.

**Scheme 3:**
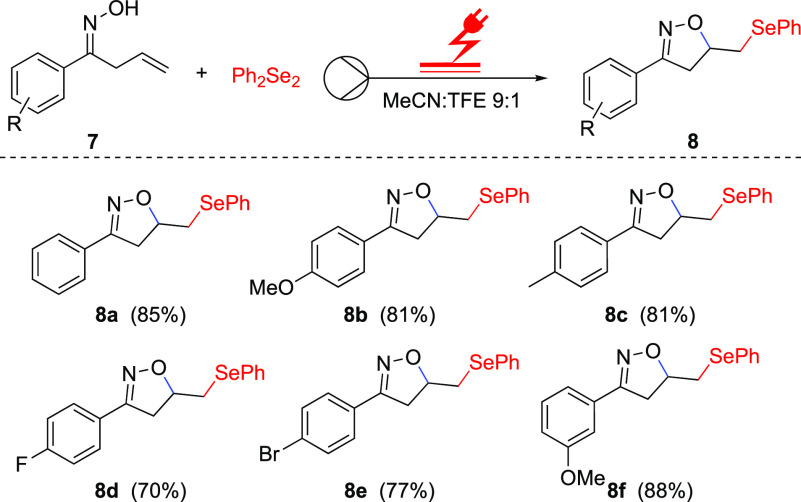
Substrate Scope of the Electrochemical Selenocyclization of Unsaturated Oximes **7** in Flow

The method was also successful with electron-rich substrates containing methoxy, isopropyl, or ester substituents, as the products were formed in moderate to good yields (**6f**–**h**). Substrates containing electron-rich and -withdrawing groups at the *meta*-position (**5i**–**l**) were also investigated, resulting in the products being obtained in good yields of 70 and 68% (**6i**–**j**), and 65% (**6k,l**). Gratifyingly, *ortho*-substituents were also suitable for this transformation, providing the desired products in good yields ranging between 65 and 78% (**6m**–**q**). In addition, substrates with furan, thiophene, and pyridine moieties (**5r**–**t**) afforded the corresponding seleno oxazolines in good yields of 73% (**6r–t**). *N*-Homoallylic amides **5u** and **5v** afforded the corresponding six-membered dihydro-2*H* pyran products **6u** and **6v** in 81 and 65% yields, respectively. Substrates (**5w**–**y**) with cyclohexyl, cyclobutyl, and cyclopropyl moieties yielded the desired products in moderate yields (**6w**–**y**). Similar results were obtained when the *N*-allyl pivalamide (**5z**) and *N*-allyl-2-naphthamide (**5aa**) were employed, which allowed the seleno oxazolines to be obtained in 65 and 54% yield, respectively (**6z** and **6aa**). Substrates with a methyl group on the alkene moiety (**5ab** and **5ac**) were successful and led to the corresponding desired products (**6ab** and **6ac**) in good yields of 73 and 65%, respectively. Furthermore, *N*-propargylamides (**5ad**) also delivered the desired product (**6ad**) in a high yield of 70% with *E*-configuration, which was established by ^1^H NMR data comparison to a known compound.^26^ Other diselenides (**5ae–ag**) also reacted successfully with *N*-allyl benzamide **5a**, leading to the desired products (**6ae–ag**) in 69–77% yield. To further study the scope, the selenocyclization of unsaturated oxime **7** was studied. The results showed that electron-rich or withdrawing substituents on the *para-* and *ortho*-position resulted in the compounds **8a**–**f** being obtained in high yields ranging from 70 to 88% (Scheme 3).

**Scheme 4:**
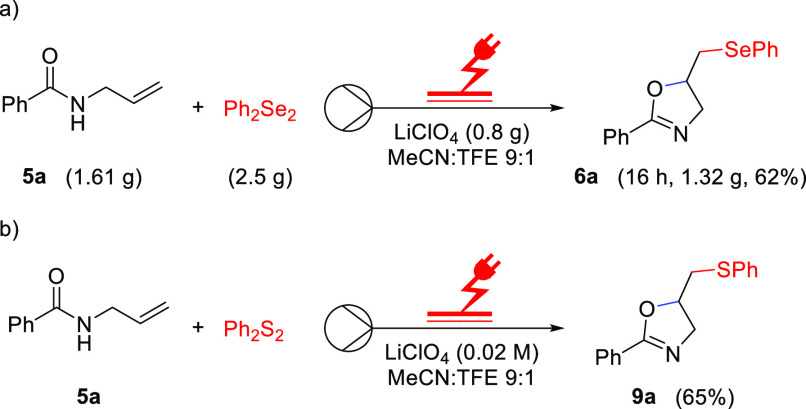
(a) Scale-Up Experiment; (b) Sulfur Functionalization

To illustrate the scalability of this approach, *N*-allylbenzamide **5a** proceeded under the optimal flow electrochemical conditions leading to obtaining the desired product **6a** in a good yield of 62% after 16 h (Scheme 4). The lower yield in the scale-up reaction compared to the small-scale reaction is due to some fouling of the electrode, which is visible already after 8 h of reaction time. In Scheme 4b it is illustrated that the same flow approach can be used to synthesize sulfur-functionalized oxazolidines **9**.

**Scheme 5:**
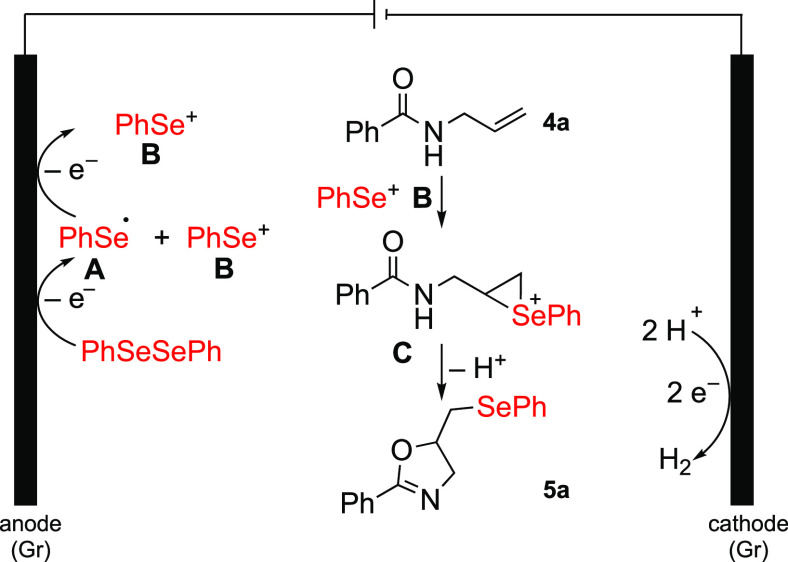
Proposed Reaction Mechanism for the Electrochemical Selenocyclization

Based on previously published reports^26,27^ for electrochemical selenylations, a possible reaction mechanism is shown in Scheme 5. Initially, the reaction pathway shows the cathodic reduction of diphenyl diselenide, producing seleno radical **A** and selenium cation **B** from diphenyl diselenide. Following that, the phenyl selenium radical is oxidized by another one-electron transfer to **B**. The selenium cation **B** is added to the double bond of **4a** to generate intermediate **C**. This is followed by a nucleophilic cyclization to form product **5a** (Scheme 5). Although phenylselenyl radicals **A** must be produced at the electrode, their direct involvement in a radical reaction is excluded as substrates such as cyclopropyl derivative **5y** would be undergoing ring opening.

## Conclusions

In summary, we have presented the electrochemical selenocyclization of *N*-allyl benzamides and unsaturated oximes to selenofunctionalized oxazolines and isoxazolines via a continuous flow electrochemical approach. This approach is suitable for a wide substrate scope, allowing the synthesis of selenofunctionalized oxazolines and isoxazolines in good yields. Furthermore, mild reaction conditions were utilized without the use of any hazardous expensive oxidants and less toxic solvents due to minimizing any harsh reaction conditions. Selenofunctionalized oxazoline derivatives were demonstrated to be easily scaled up safely.

## Experimental Section

General flow electrolysis procedure for the preparation of selenylated oxazolines **6**:

The electrolysis was performed in an undivided cell using a Vaportec Ion Electrochemical Flow Reactor (reactor volume 0.6 mL, spacer 0.5 mm), employing a graphite electrode as the anode and as the cathode (active surface area = 12 cm^2^ for each electrode). A solution of *N*-allylbenzamide **5** (0.05 M, 0.5 mmol) was placed in a vial with a mixture of diphenyl diselenide (125 mg, 0.4 mmol) and LiClO_4_ (21 mg, 0.2 mmol) in a mixture of acetonitrile (9 mL) and 2,2,2-trifluoroethanol (1 mL) was pumped with a flow rate of 0.15 mL min^–1^ and was electrolyzed under constant current conditions (*j* = 3.5 mA cm^–2^, active surface area 12 cm^2^ for each electrode, 3.5 F/mol) at 25 °C. After reaching a steady state and collection for a known period, the solvent was removed under vacuum. The crude product was purified by column chromatography (petroleum ether/ethyl acetate, 7:3).

## Data Availability

The data underlying this study are available in the published article and its online Supporting Information.

## Supporting Information Available

Reaction optimization studies, synthetic procedures, and characterization data, spectroscopic data for new compounds, and copies of NMR spectra (PDF)
