# Supplementary material for: Continuous Flow Electroselenocyclization of Allylamides and Unsaturated Oximes to Selenofunctionalized Oxazolines and Isoxazolines
Source: ACS Org Inorg Au. 2024 Mar 11;4(3):350–5. doi: 10.1021/acsorginorgau.4c00008 (PMC11157512; doi:10.1021/acsorginorgau.4c00008)
Supplement: Supplementary file 1 — gg4c00008_si_001.pdf [file gg4c00008_si_001.pdf]

Supporting Information

Continuous Flow Electroselenocyclization of Allylamides and Unsaturated Oximes to Selenofunctionalized Oxazolines and Isoxazolines

Ohud Alzaidi[a,b], and Thomas Wirth*[a]

[a] School of Chemistry, Cardiff University, Park Place, Main Building, Cardiff CF10 3AT, United Kingdom

[b] Department of Chemistry, College of Science – Al Khurma, Taif University, Saudi Arabia

*E-mail: wirth@cf.ac.uk

Table of Contents:

General Information..S3

General Procedures for the Synthesis of the Starting Materials..S4

General Procedure GP1 for the Synthesis of *N*-Allyl/Homoallyl Amide Substrates:.................S4

General Procedure GP2 for the Synthesis of Unsaturated Oximes:..S6

General Electrolysis Procedures S7

General Batch Electrolysis Procedure GP3 for Selenylated Oxazolines.................................S7

General flow Electrolysis Procedure GP4 for Selenylated Oxazolines..................................S7

General flow Electrolysis Procedure GP5 for Selenylated Isoxazoline..................................S7

Optimization reaction conditions:...S10

Characterization of the Products:...S14

NMR Spectra for the Products:...S35

References:..S112

General Information

General*:*

Unless otherwise noted, reactions involving air and moisture sensitive reagents were performed under an argon atmosphere using flame-dried glassware. Low temperature reactions at 0°C were cooled using ice/water baths. Room temperature (rt) refers to 20 – 25 °C. Thin layer chromatography was performed on pre-coated aluminium sheets of Merck silica gel 60 F254 (0.20 mm) and visualized by UV radiation (254 nm). Flash column chromatography was performed on Biotage® Isolera Four using Biotage® cartridges SNAP Ultra 10 g or 25 g and Biotage® cartridges Sfär 10 g or 25 g. Non-UV-visible compounds were separated by manual flash chromatography using silica gel (Sigma-Aldrich, technical grade, pore size 60 Å, 230-400 mesh particle size, 40-63 µm particle size).
All chemicals were purchased from commercial suppliers (Sigma Aldrich, Alfa Aesar, Acros Organic, VWR and FluoroChem) without further purification and used as received.
Dry solvents such THF, diethyl ether and toluene were obtained after passing these previously degassed solvents through activated alumina columns (Mbraun, SPS-800). Dry dichloromethane was obtained using phosphorous pentoxide, and then distilling when needed. Deuterated solvents for NMR analysis were purchased from Sigma Aldrich.

NMR:

^{1}H NMR, ^{13}C NMR and ^{19}F NMR spectra were recorded on a Bruker Fourier 300 apparatus and referenced relative to the residual solvent peaks (^{1}H: $CDCl_3$, δ 7.26 ppm; ^{13}C: $CDCl_3$, δ 77.2 ppm). The chemical shifts δ values are given in parts per million (ppm). The multiplicity of the signals was declared as followed: s = singlet, d = doublet, t = triplet, q = quartet, quin = quintet, sex = sextet, hep = septet, dd = doublet of doublets, m = multiplet, b = broad; and coupling constants (J) in Hertz.

IR:

Infrared spectroscopy was conducted with a Shimadzu FTIR Affinity-1S apparatus. Wavenumbers are quoted in cm^{-1}. All compounds were measured neat directly on the crystal of the IR machine.

HRMS:

High resolution mass spectra were obtained from Cardiff University on a Waters GC-TOF spectrometer. Ions were generated using Electron Ionisation (EI), Chemical Ionisation (CI) or Atmospheric Pressure Chemical Ionisation (APCI) techniques. All signals are reported with the quotient from mass to charge m/z.

Melting points:

Melting points of solids were measured using a Gallenkamp variable heater with samples in open capillary tubes.

Electrolysis:

Batch electrochemical experiments were carried out with an Electrasyn 2.0 device. Graphite (5 cm × 0.8 cm × 0.2 cm) electrodes, distance 0.5 cm, submerged surface area 2.4 cm^2 from IKA were used in 5 mL Electrasyn vial. Flow electrochemical experiments were performed using a stand-alone Vapourtec Ion Electrochemical Reactor with an Aim-TTi EX354RD Dual Power Supply from Thurlby Thandar Instruments Ltd. Chemyx Fusion 100 Touch Syringe Pumps or a Vapourtec SF-10 Reagent Pump were used in the flow set-ups. PTFE Capillary Tubing 1/16'' OD x 1.00 mm ID from Polyflon Technology Limited was employed. Electrode materials employed were Graphite (Gr), Platinum (Pt), Stainless Steel (SS), and Copper (Cu), Glassy Carbon (GC) purchased from Goodfellow. The electrodes (5 × 5 cm^2) are separated by a 0.5 mm spacer with a channel volume of 0.6 mL and an exposed electrode surface area of 12 cm^2. (each electrode).

General Procedures for the Synthesis of the Starting Materials

General Procedure GP1 for the Synthesis of *N*-Allyl/Homoallyl Amide Substrates:[1]

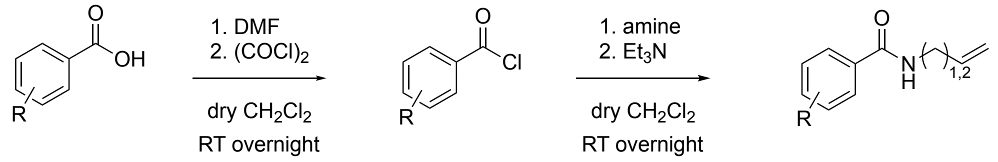

To a solution of the acid derivative (2 mmol, 1 equiv) in dry CH_2Cl_2 (15 mL) under a dry argon atmosphere atmosphere was added a catalytic amount of DMF (100 µL) at 0 ℃. The mixture was stirred at 0 ℃ for 15 min, and then oxalyl chloride (330 mg, 2.6 mmol) was added dropwise at 0 ℃. The reaction mixture was stirred overnight at room temperature and then the solvent was removed in vacuo. The crude acid chloride was dissolved in dry CH_2Cl_2 (10 mL) and was added slowly to a stirring solution of the amine (2 mmol, 1 equiv) and Et_3N (445 mg, 4.4 mmol, 2.2 equiv) in dry CH_2Cl_2 (15 mL). The solution was stirred for 24 h at room temperature. The reaction was quenched carefully with an aqueous solution of NaOH (1 M, 10 mL), and extracted with CH_2Cl_2 (3 × 20 mL). The combined organic layers were washed with brine (15 mL), dried over $MgSO_4$ (4 g), filtered, and concentrated in vacuo. The crude product was purified by flash chromatography starting with 100% petroleum ether and increasing up to 75% ethyl acetate as the eluent.

Table S1: Starting materials:

Various *N*-Allyl/Homoallyl Amide were prepared:

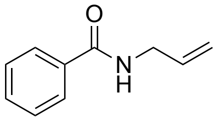

4a
[Ref. 2]

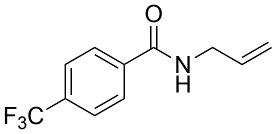

4b
[Ref. 5]

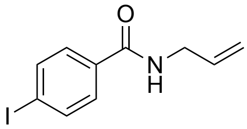

4c
[Ref. 4]

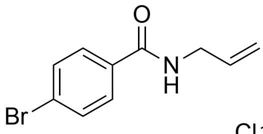

4d
[Ref. 2]

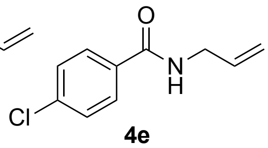

4e
[Ref. 3]

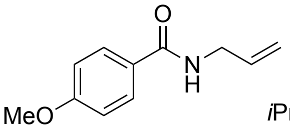

4f
[Ref. 2]

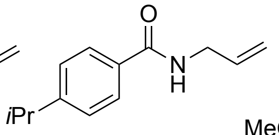

4g
[Ref. 4]

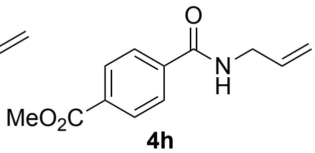

4h
[Ref. 6]

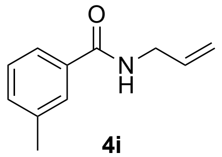

4i
[Ref. 7]

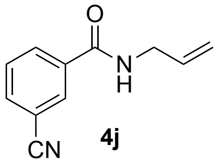

4j
[Ref. 1]

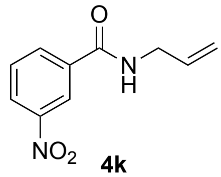

4k
[Ref. 3]

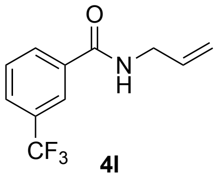

4l
[Ref. 1]

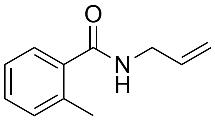

4m
[Ref. 1]

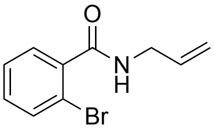

4n
[Ref. 2]

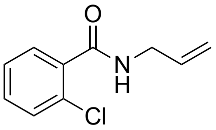

4o
[Ref. 5]

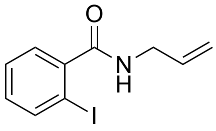

4p
[Ref. 4]

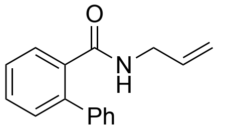

4q
[Ref. 5]

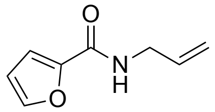

4r
[Ref. 2]

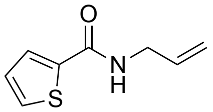

4s
[Ref. 2]

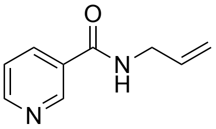

4t
[Ref. 8]

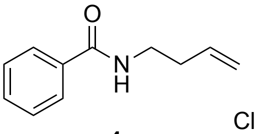

4u
[Ref. 9]

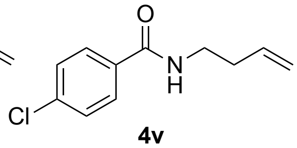

4v
[Ref. 10]

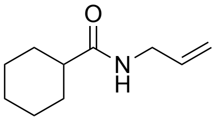

4w
[Ref. 3]

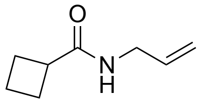

4x
[Ref. 11]

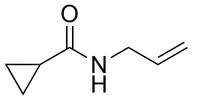

4y
[Ref. 12]

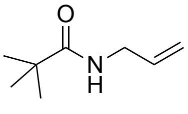

4z
[Ref. 2]

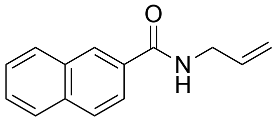

4aa
[Ref. 4]

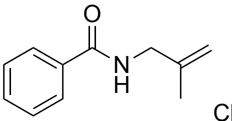

4ab
[Ref. 3]

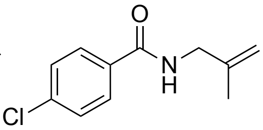

4ac
[Ref. 3]

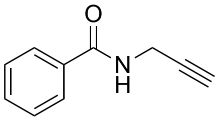

4ad
[Ref. 24]

General Procedure GP2 for the Synthesis of Unsaturated Oximes:[13]

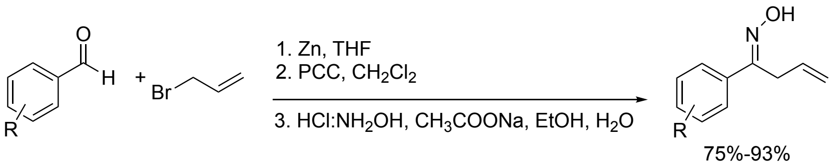

1. To a 100 mL round-bottom flask were added allylbromide (3.02 mL, 35 mmol, 3.5 equiv) dry THF (30 mL), and zinc dust (2287 mg, 35 mmol, 3.5 equiv) was slowly added at 0 °C and stirred for 10 min. The aldehyde derivative (10 mmol, 1 equiv) was dissolved in dry THF (15 mL) and added dropwise under N_2 to the stirring solution at 0 ℃ for 30 minutes. After that added an aqueous saturated solution of NH_4Cl (35 mmol, 3.5 equiv), and then stirring for 3 hours. After that filtered and extracted with ethyl acetate (3 × 15 mL). The combined organic layers were washed with water (1×10 mL) and brine (1× 10 mL), then dried over $MgSO_4$ (5 g), filtered, and concentrated under reduced pressure.
2. A round-bottom flask was charged with a solution of crude homoallylic alcohol product in CH_2Cl_2 (30 mL), and pyridinium chlorochromate (7.5 g, 35.0 mmol, 3.5 equiv)) was added slowly at 0 °C. The resulting suspension was stirred for 2 h at room temperature. After the reaction is completed diluted with water (20 mL) and extracted with ethyl acetate (3 × 20 mL). The organic layers were washed with brine and dried with $MgSO_4$ (5 g), filtered and the solvent was removed in vacuo. The crude product was used for the next step without purification.
3. The crude ketone was dissolved in ethanol (15 mL) and added to the solution of hydroxylamine hydrochloride (3.5 g, 50.0 mmol) was dissolved in ethanol (30 mL) and mixed with a solution of CH_3COONa (5.74 g, 70.0 mmol) in 30 mL water. The reaction was stirred for 24 h until the reaction was complete (monitored by TLC). The solvent was concentrated in vacuo and extracted with ethyl acetate (3 x 20 mL). The organic layers were washed with brine and dried with $MgSO_4$ (5 g), filtered and the solvent was removed in vacuo. The crude product was purified by silica gel flash column chromatography starting with 100% cyclohexane and increasing up to 70% ethyl acetateas the eluent.

Unsaturated oximes prepared:

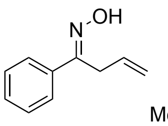

7a
[Ref. 13]

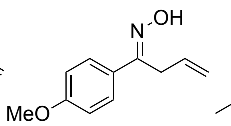

7b
[Ref. 13]

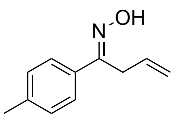

7c
[Ref. 14]

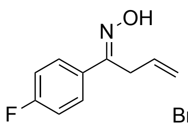

7d
[Ref. 13]

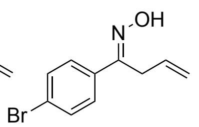

7e
[Ref. 13]

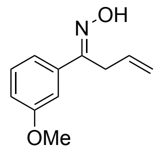

7f
[Ref. 14]

General Electrolysis Procedures

General Batch Electrolysis Procedure GP3:

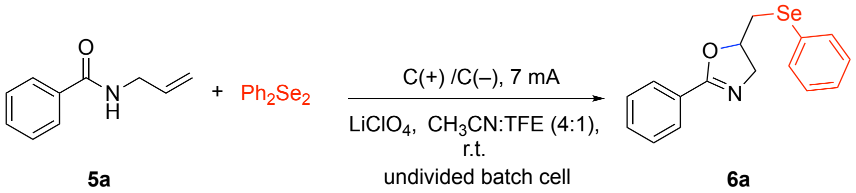

Batch electrolysis was performed using the IKA Electrasyn 2.0.[15] A solution of substrate **1** (24 mg, 0.15 mmol, 1 equiv.) , diphenyl diselenide (38 mg, 0.12 mmol, 0.8 equiv.) and $LiClO_4$ electrolyte (53 mg, 0.5 mmol) in a mixture of acetonitrile (4 mL) and 2,2,2-trifluoroethanol (1 mL) was electrolyzed using an ElectraSyn oven-dried undivided cell (5 mL glass vial) equipped with graphite anode and graphite cathode under constant current of (j = 2.92 mA·cm^{-2}, active surface area 2.4 cm^2, 2.5 F/mol) with stirring (500 rpm) the size of stir bar (12 x 4.5 mm) for 1.26 h at 25 °C. After electrolysis, solvent was evaporated in vacuo. The crude product was purified by flash chromatography on silica gel using (petroleum ether/ ethyl acetate, 7:3). The yield determined by ^{1}H NMR using 1,3,5-trimethoxybenzene as internal standard.

General Flow Electrolysis Procedure for Preparation of Selenylated Oxazolines GP4:

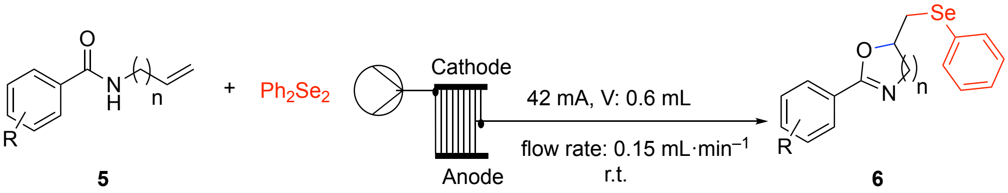

The electrolysis was performed in an undivided cell using a Vapourtec Ion Electrochemical Flow Reactor (reactor volume 0.6 mL, spacer 0.5 mm),[16] employing a graphite electrode as the anode and as the cathode (active surface area = 12 cm^2 for each electrode). A solution of *N*-allylbenzamide **5** (0.05 M, 0.5 mmol) was placed in vial with a mixture of diphenyl diselenide (125 mg, 0.4 mmol) and $LiClO_4$ (21 mg, 0.2 mmol) in a mixture of acetonitrile(9 mL) and 2,2,2-trifluoroethanol (1 mL) were pumped with a flow rate of 0.15 mL min^{-1} and were electrolyzed under constant current conditions (j = 3.5 mA·cm^{-2}, active surface area 12 cm^2 for each electrode, 3.5 F/mol) at 25 °C. After reaching a steady state and collection for a known period, the solvent was removed under vacuum. The crude product was purified by column chromatography (petroleum ether/ ethyl acetate, 7:3).

General Flow Electrolysis Procedure for Preparation of Selenylated Isoxazolines GP5:

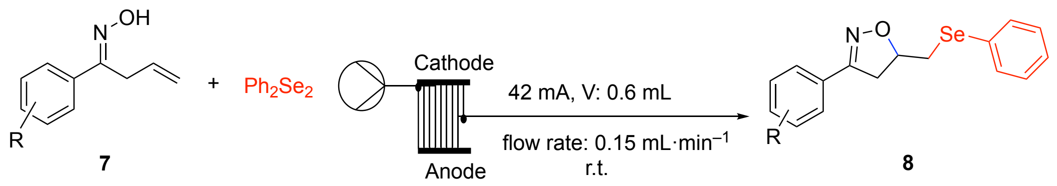

The electrolysis was performed in an undivided cell using a Vapourtec Ion Electrochemical Flow Reactor (reactor volume 0.6 mL, spacer 0.5 mm),[16] employing a graphite electrode as the anode and as the cathode (active surface area = 12 cm^2 for each electrode). A solution of unsaturated oxime derivative **7** (0.05 M, 0.5 mmol) was placed in vial with a mixture of diphenyl diselenide (125 mg, 0.4 mmol) and $LiClO_4$ (21 mg, 0.2 mmol) in a mixture of acetonitrile (9 mL) and 2,2,2-trifluoroethanol (1 mL) were pumped with a flow rate of 0.15 mL min^{-1} and were electrolysed under constant current conditions (j = 3.5 mA·cm^{-2}, active surface area 12 cm^2 for each electrode, 3.5 F/mol) at 25 °C. The first one and a half reactor volumes of each run were discarded to ensure that a steady state had been reached and the result of the run was an accurate representation of the system. After collection for a known period, and the solvent was removed under vacuum. The crude product was purified by column chromatography (petroleum ether/ethyl acetate 7:3).

Scale-up Flow Electrolysis Procedure GP 6:

The scale-up electrolysis was performed in an undivided cell using a Vapourtec Ion Electrochemical Flow Reactor (reactor volume 0.6 mL, spacer 0.5 mm),[16] employing a graphite electrode as the anode and as the cathode (active surface area = 12 cm^2 for each electrode). A solution of *N*-allylbenzamide (1.61 g, 10 mmol) was placed in vial with a mixture of diphenyl diselenide (2.51 g, 8 mmol) and $LiClO_4$ (426 mg, 4 mmol) in a mixture of acetonitrile (190 mL) and 2,2,2-trifluoroethanol (10 mL) were pumped with a flow rate of 0.15 mL min^{-1} and were electrolysed under constant current conditions (j = 3.5 mA·cm^{-2}, active surface area 12 cm^2 for each electrode, 3.5 F/mol) at 25 °C. The first one and a half reactor volumes of each run were discarded to ensure that a steady state had been reached and the result of the run was an accurate representation of the system. After collection for a known period (16 h), and the solvent was removed under vacuum. The crude product was purified by column chromatography (petroleum ether/ ethyl acetate, 7:3).

Electrochemical Flow Equipment

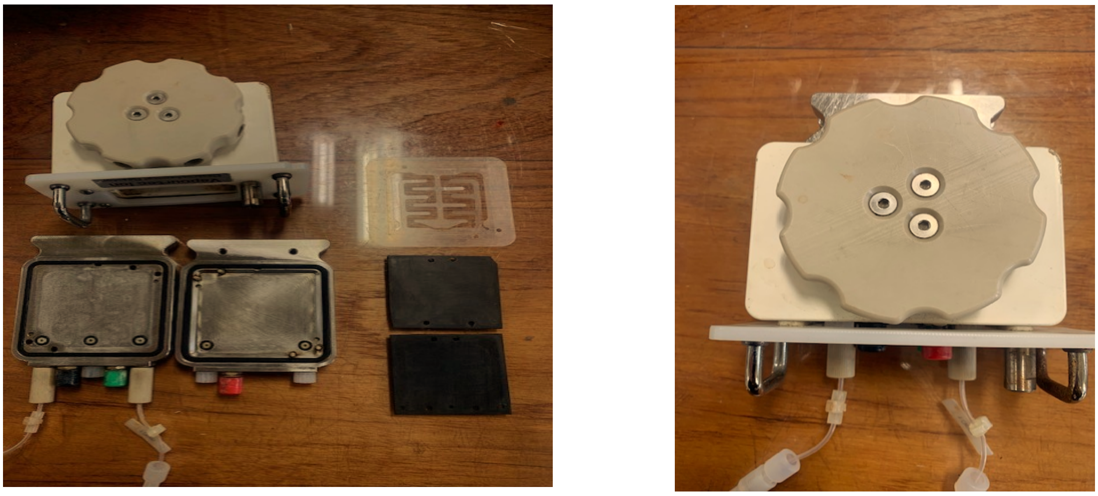

Figure S1: Left: Vapourtec Ion Electrochemical Flow Reactor (reactor volume = 0.6 mL) opened reactor, Gr electrodes and spacer 0.5 mm. Right: Vapourtec Ion Electrochemical Flow Reactor closed reactor.

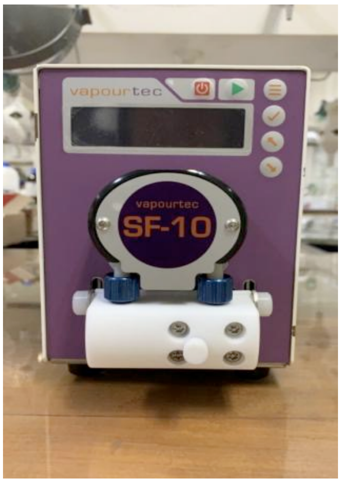

Figure S2: Vapourtec SF-10 reagent pump.

Optimisation Studies:

Table S1: Optimisation of Solvent

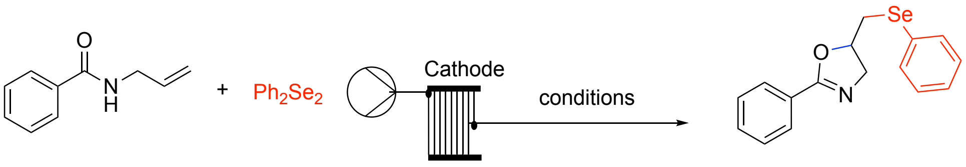

entry	solvent	**6a** yield (%)
1	MeCN	85
2	MeOH	75
3	HFIP	0
**4**	**MeCN:TFE (9:1 V/V)**	**93**

Reaction conditions: **5a** (0.05 M, 1 equiv.), $(PhSe)_2$ (0.04 M, 0.8 equiv.), $LiClO_4$ (0.02 M), MeCN/TFE (9:1 v/v), Gr anode, Gr cathode (active surface area 12 cm^2), undivided flow cell, flow rate: 0.15 mL min^{-1}, constant current = 42 mA, charge: 3.5 F /mol at rt. The yield was determined by ^1H NMR using 1,3,5-trimethoxybenzene as internal standard.

Table S2: Optimisation of electrode materials

A: Optimisation of anodic material

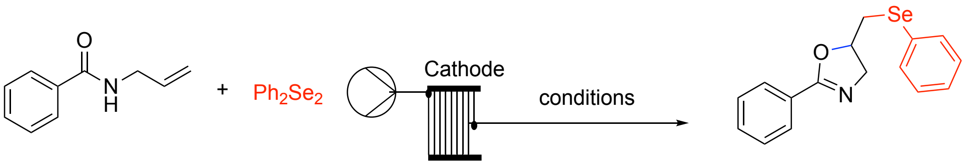

entry	anode	cathode	current (mA)	charge (F/mol)	**6a** yield (%)
1	Gr	Pt	42	3.5	73
**2**	**Pt**	**Pt**	**42**	**3.5**	89
3	GC	Pt	42	3.5	75

Reaction conditions: **5a** (0.05 M, 1 equiv.), $(PhSe)_2$ (0.04 M, 0.8 equiv.), $LiClO_4$ (0.02 M), MeCN/TFE (9:1 v/v), X anode, Pt cathode (active surface area 12 cm^2), undivided flow cell, flow rate: 0.15 mL min^{-1}, constant current = 42 mA, charge: 3.5 F/mol at rt. The yield was determined by ^1H NMR using 1,3,5-trimethoxybenzene as internal standard.

B: Optimisation of cathodic material.

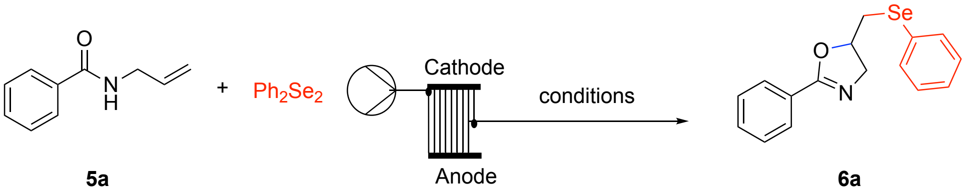

entry	anode	cathode	current (mA)	charge (F/mol)	**6a** yield (%)
**1**	**Gr**	**Gr**	**42**	**3.5**	**93**
2	Gr	Pt	42	3.5	73
3	Gr	GC	42	3.5	70
4	Gr	SS	42	3.5	40
5	Gr	Cu	42	3.5	25

Reaction conditions: **5a** (0.05 M, 1 equiv), $(PhSe)_2$ (0.04 M, 0.8 equiv), $LiClO_4$ (0.02 M), MeCN/TFE (9:1 v/v), Gr anode, X cathode(active surface area 12 cm^2), undivided flow cell, flow rate: 0.15 mL min^{-1}, constant current = 42 mA, charge: 3.5 F /mol at rt. The yield was determined by ^1H NMR using 1,3,5-trimethoxybenzene as internal standard.

Table S3: Optimisation of charge

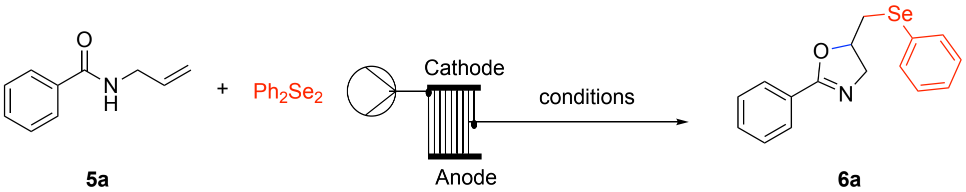

entry	current (mA)	charge (F/mol)	Current density (mA/cm^2)	**6a** yield (%)
1	27	2.25	2.25	70
2	30	2.5	2.5	73
3	36	3	3	89
**4**	**42**	**3.5**	3.5	**93**

Reaction conditions: **5a** (0.05 M, 1 equiv), $(PhSe)_2$ (0.04 M, 0.8 equiv), $LiClO_4$ (0.02 M), MeCN/TFE (9:1 v/v), Gr anode, Gr cathode(active surface area 12 cm^2), undivided flow cell, flow rate: 0.15 mL min^{-1}, constant current = X mA, charge: X F /mol at rt. The yield was determined by ^1H NMR using 1,3,5-trimethoxybenzene as internal standard.

Table S4: Optimisation of concentration

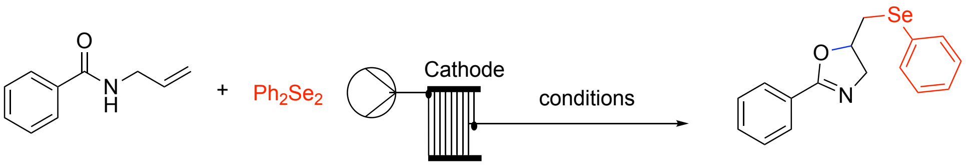

5a 6a

entry	[**5a**] (M)	[(PhSe)2] (M)	current (mA)	charge (F/mol)	**6a** yield (%)
1	0.025	0.02	21	3.5	78
**2**	**0.05**	**0.04**	**42**	**3.5**	**93**
3	0.075	0.06	63	3.5	58
4	0.1	0.08	84	3.5	41

Reaction conditions: **5a** (X M, 1 equiv.), $(PhSe)_2$ (Y M, 0.8 equiv.), $LiClO_4$ (0.02 M, 0.2mmol), MeCN/TFE (9:1 v/v), Gr anode, Gr cathode(active surface area 12 cm^2), undivided flow cell, flow rate: 0.15 mL min^{-1}, constant current = X mA, charge : 3.5 F /mol at rt. The yield was determined by ^{1}H NMR using 1,3,5-trimethoxybenzene as internal standard.

Table S5: Optimisation of Flow rate screening

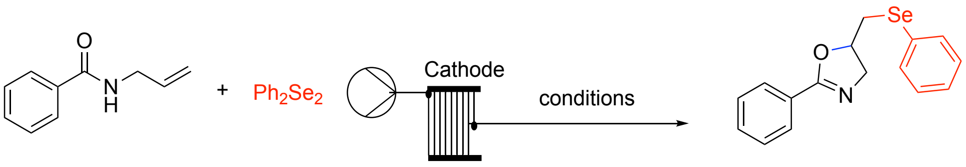

5a 6a

entry	flow rate (mL min^{-1})	current (mA)	Calculated residence time (min)	**6a** yield (%)
1	0.05	14	12	24
2	0.1	28	6	69
**3**	**0.15**	**42**	4	**93**
4	0.2	56	3	70

Reaction conditions: **5a** (0.05 M, 1 equiv.), $(PhSe)_2$ (0.04 M, 0.8 equiv.), $LiClO_4$ (0.02 M, 0.2mmol), MeCN/TFE (9:1 v/v), Gr anode, Gr cathode (active surface area 12 cm^2), undivided flow cell, flow rate: X mL min^{-1}, charge : 3.5 F /mol at rt. The yield was determined by ^{1} H NMR using 1,3,5-trimethoxybenzene as internal standard.

Table S6: Optimisation of electrolyte screening

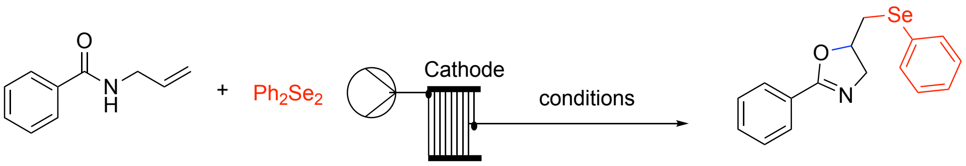

5a **6a**

entry	electrolyte (M)	**6a** yield (%)
1	$LiClO_4$ (0.002 M)	53
2	$LiClO_4$ (0.01 M)	75
**3**	**$LiClO_4$ (0.02 M)**	**93**
4	Et_4NBF_4 (0.02 M)	66
5	$BuNClO_4$(0.02 M)	70
6	-	trace

Reaction conditions: **5a** (0.05 M, 1 equiv.), $(PhSe)_2$ (0.04 M, 0.8 equiv.), $LiClO_4$ (X M), MeCN/TFE (9:1 v/v), Gr anode, Gr cathode (active surface area 12 cm^2), undivided flow cell, flow rate: 0.15 mL min^{-1}, constant current = 42 mA, charge: 3.5 F /mol at rt. The yield was determined by ^1H NMR using 1,3,5-trimethoxybenzene as internal standard.

Characterization of the Products:

2-Phenyl-5-((phenylselanyl)methyl)-4,5-dihydrooxazole (6a)

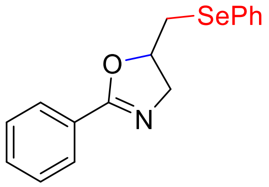

Prepared according to the general procedure GP4 from *N*-allylbenzamide (42 mg, 0.26 mmol) over 35 minutes. The product was purified by column chromatography, applying (petroleum ether: ethyl acetate 7:3) as eluent to get the product as a colorless oil (74 mg, 0.233 mmol, 90%).

^{1}H NMR (300 MHz, $CDCl_3$) δ = 7.85 (d, *J* = 6.9 Hz, 2H), 7.60 – 7.51 (m, 2H), 7.48 – 7.41 (m, 1H), 7.40 – 7.32 (m, 2H), 7.28 – 7.24 (m, 3H), 4.93 – 4.80 (m, 1H), 4.14 (dd, *J* = 15.0, 9.5 Hz, 1H), 3.81 (dd, *J* = 15.0, 6.9 Hz, 1H), 3.27 (dd, *J* = 12.7, 5.4 Hz, 1H), 3.03 (dd, *J* = 12.7, 7.4 Hz, 1H) ppm.

^{13}C {^{1}H} NMR (75 MHz, $CDCl_3$) δ = 163.7, 133.4, 131.3, 129.2, 128.9, 128.3, 128.2, 127.6, 127.5, 78.8, 60.5, 32.1 ppm.

HRMS (ESI-TOF) m/z: $[M+H]^+$ calcd for $C_{16}H_{16}NOSe$ 318.0401; found: 318.0397.

The spectral data are in agreement with the literature.[17]

5-((Phenylselanyl)methyl)-2-(4-(trifluoromethyl)phenyl)-4,5-dihydrooxazole (6b)

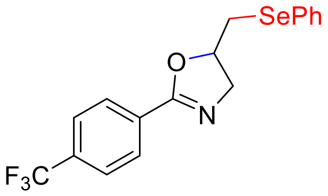

Prepared according to the general procedure GP4 from *N*-allyl-4-(trifluoromethyl)benzamide (60 mg , 0.26 mmol) over 35 minutes. The product was purified by column chromatography, applying (petroleum ether: ethyl acetate 7:3) as eluent to get the product as a colorless oil (86 mg, 0.22 mmol, 85%).

^{1}H NMR (300 MHz, $CDCl_3$) δ = 7.94 (d, *J* = 8.1 Hz, 2H), 7.64 (d, *J* = 8.2 Hz, 2H), 7.56 (dd, *J* = 6.3, 3.3 Hz, 2H), 7.25 (dd, *J* = 4.1, 2.0 Hz, 3H), 5.00 – 4.88 (m, 1H), 4.19 (dd, *J* = 15.2, 9.5 Hz, 1H), 3.86 (dd, *J* = 15.2, 7.0 Hz, 1H), 3.28 (dd, *J* = 12.8, 5.4 Hz, 1H), 3.08 (dd, *J* = 12.8, 7.2 Hz, 1H) ppm.

^{13}C {^{1}H} NMR (75 MHz, $CDCl_3$) δ = 162.6, 133.4, 132.9 (d, *J* = 32.6 Hz), 129.3(d, J = 216.5 Hz),, 128.5, 127.6, 125.3 (d, *J* = 3.8 Hz), 123.2 (d, *J* = 13.2 Hz), 79.4 , 60.3, 31.9 ppm.

^{19}F NMR (471 MHz, $CDCl_3$) δ = -62.95.

The spectral data are in agreement with the literature.[17]

2-(4-Iodophenyl)-5-((phenylselanyl)methyl)-4,5-dihydrooxazole (6c)

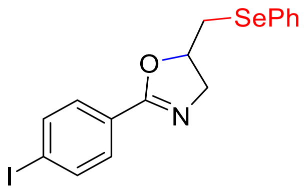

Prepared according to the general procedure GP4 from *N*-allyl-4-iodobenzamide (75 mg, 0.26 mmol) over 35 minutes. The product was purified by column chromatography, applying (petroleum ether: ethyl acetate 7:3) as eluent to get the product as a colorless oil (83 mg, 0.19 mmol, 73%).

^{1}H NMR (300 MHz, $CDCl_3$) δ = 7.71(dd, *J* = 6.9, 2.1Hz, 2H), 7.57 – 7.51 (m, 2H), 7.28 – 7.22 (m, 5H), 4.87 (m, 1H), 4.12 (dd, *J* = 15.1, 9.5 Hz, 1H), 3.79 (dd, *J* = 15.1, 7.0 Hz, 1H), 3.25 (dd, *J* = 12.8, 5.5 Hz, 1H), 3.03 (dd, *J* = 12.9, 7.5 Hz, 1H) ppm.

^{13}C {^{1}H} NMR (75 MHz, $CDCl_3$) δ = 163.1, 137.5, 133.3, 129.7, 129.3, 128.9, 127.5, 127.1, 98.4, 79.1, 60.1, 32.2 ppm.

HRMS (ESI-TOF) m/z: $[M+H]^+$ calcd for $C_{16}H_{15}INOSe$ 443.9366; found: 443.9364.

The spectral data are in agreement with the literature.[19]

2-(4-Bromophenyl)-5-((phenylselanyl)methyl)-4,5-dihydrooxazole (6d)

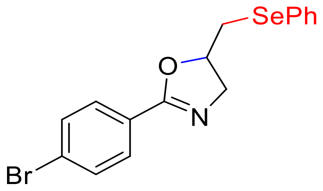

Prepared according to the general procedure GP4 from *N*-allyl-4-iodobenzamide (62 mg, 0.26 mmol) over 35 minutes. The product was purified by column chromatography, applying (petroleum ether: ethyl acetate 7:3) as eluent to get the product as a colorless oil (90 mg, 0.23 mmol, 88%).

^{1}H NMR (300 MHz, $CDCl_3$) δ = 7.67 (d, *J* = 8.7 Hz, 2H), 7.54 (m, 2H), 7.49 (m, 2H), 7.25 (m, 3H), 4.88 (dtd, *J* = 9.5, 7.1, 5.5 Hz, 1H), 4.13 (dd, *J* = 15.1, 9.5 Hz, 1H), 3.79 (dd, *J* = 15.1, 6.9 Hz, 1H), 3.25 (dd, *J* = 12.8, 5.5 Hz, 1H), 3.03 (dd, *J* = 12.8, 7.2 Hz, 1H) ppm.

^{13}C {^{1}H} NMR (75 MHz, $CDCl_3$) δ = 163.1, 133.4, 131.5, 129.7, 129.2, 128.8, 127.5, 126.5, 126.0, 79.3, 60.1, 31.9 ppm.

HRMS (ESI-TOF) m/z: $[M+H]^+$ calcd for $C_{16}H_{15}BrNOSe$ 395.9502; found: 395.9502.

The spectral data are in agreement with the literature.[18]

2-(4-Chlorophenyl)-5-((phenylselanyl)methyl)-4,5-dihydrooxazole (6e)

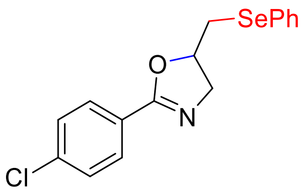

Prepared according to the general procedure GP4 from *N*-allyl-4-chlorobenzamide (51 mg, 0.26 mmol) over 35 minutes. The product was purified by column chromatography, applying (petroleum ether: ethyl acetate 7:3) as eluent to get the product as a colorless oil (80 mg, 0.23 mmol, 88%).

^{1}H NMR (300 MHz, $CDCl_3$) δ = 7.78 – 7.71 (m, 2H), 7.56 – 7.53 (m, 2H), 7.33 (d, *J* = 8.8 Hz, 2H), 7.25 (dt, *J* = 4.1, 1.1 Hz, 3H), 4.88 (dtd, *J* = 9.5, 7.1, 5.5 Hz, 1H), 4.14 (dd, *J* = 15.1, 9.5 Hz, 1H), 3.80 (dd, *J* = 15.1, 6.9 Hz, 1H), 3.29 – 3.21 (m, 1H), 3.09 – 2.99 (m, 1H) ppm.

^{13}C {^{1}H} NMR (75 MHz, $CDCl_3$) δ = 163.0, 137.5, 133.4, 129.5, 129.2, 128.8, 128.6, 127.5, 126.1, 79.1, 60.0, 31.6 ppm.

HRMS (ESI-TOF) m/z: $[M+H]^+$ calcd for $C_{16}H_{15}ClNOSe$ 352.0008; found: 352.0007.

The spectral data are in agreement with the literature.[17]

2-(4-Methoxyphenyl)-5-((phenylselanyl)methyl)-4,5-dihydrooxazole (6f)

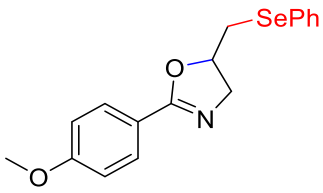

Prepared according to the general procedure GP4 from *N*-allyl-4-methoxybenzamide (50 mg, 0.26 mmol) over 35 minutes. The product was purified by column chromatography, applying (petroleum ether: ethyl acetate 8:2) as eluent to get the product as a colorless oil (80 mg, 0.23 mmol, 88%).

^{1}H NMR (300 MHz, $CDCl_3$) δ = 7.78 (d, *J* = 9.0 Hz, 2H), 7.58 – 7.50 (m, 2H), 7.52 – 7.24 (m, 2H), 6.87 (d, *J* = 9.0 Hz, 2H), 4.92 – 4.79 (m, 1H), 4.12 (dd, *J* = 14.7, 9.3 Hz, 1H), 3.82 (s, 3H), 3.26 (dd, *J* = 12.7, 5.5 Hz, 1H), 3.02 (dd, *J* = 12.7, 7.4 Hz, 1H) ppm.

^{13}C {^{1}H} NMR (75 MHz, $CDCl_3$) δ = 163.8, 162.2, 133.3, 129.9, 129.2, 128.9, 127.5, 120.0, 113.6, 79.0, 60.5, 55.8, 32.2 ppm.

HRMS (ESI-TOF) m/z: $[M+H]^+$ calcd for $C_{17}H_{18}NO_2Se$ 348.0508; found: 348.0503.

The spectral data are in agreement with the literature.[17]

2-(4-Isopropylphenyl)-5-((phenylselanyl)methyl)-4,5-dihydrooxazole (6g)

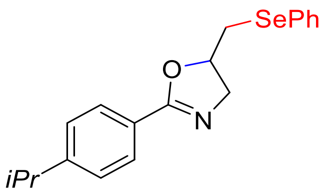

Prepared according to the general procedure GP4 from *N*-allyl-4-isopropylbenzamide (53 mg, 0.26 mmol) over 35 minutes. The product was purified by column chromatography, applying (petroleum ether: ethyl acetate 8:2) as eluent to get the product as a colorless oil (47 mg, 0.13 mmol, 50%).

^{1}H NMR (300 MHz, $CDCl_3$) δ = 7.82 (d, *J* = 8.4 Hz, 2H), 7.60 (dd, *J* = 6.4, 3.6 Hz, 2H), 7.31 (d, *J* = 4.4, 2.1 Hz, 2H), 7.29 – 7.25 (m, 3H), 4.97 – 4.81 (m, 1H), 4.18 (dd, *J* = 14.9, 9.4 Hz, 1H), 3.84 (dd, *J* = 14.9, 6.8 Hz, 1H), 3.31 (dd, *J* = 12.7, 5.4 Hz, 1H), 3.06 (dd, *J* = 12.7, 7.6 Hz, 1H), 2.95 (dq, *J* = 13.9, 7.0 Hz, 1H), 1.29 (d, *J* = 6.9 Hz, 6H) ppm.

^{13}C {^{1}H} NMR (75 MHz, $CDCl_3$) δ = 163.8, 152.8, 133.3, 129.2, 128.9, 128.2, 127.5, 126.4, 125.2, 78.7, 60.5, 34.1, 32.2, 23.8 ppm.

HRES (ESI-TOF) m/z: $[M+H]^+$ calcd for $C_{19}H_{22}NOSe$ 360.0869; found: 360.0867.

IR (neat) $\tilde{\nu}$ = 1647, 1577, 1259, 1091, 723, 690 cm^{-1}.

Methyl 4-(5-((phenylselanyl)methyl)-4,5-dihydrooxazol-2-yl)benzoate (6h)

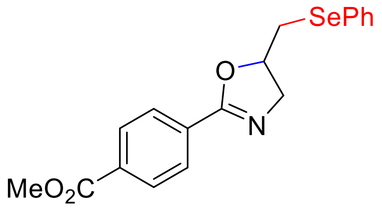

Prepared according to the general procedure GP4 from methyl 4-(allylcarbamoyl)benzoate (57 mg, 0.26 mmol) over 35 minutes. The product was purified by column chromatography, applying (petroleum ether: ethyl acetate 8:2) as eluent to get the product as a colorless oil (70 mg, 0.19 mmol, 73%).

^{1}H NMR (300 MHz, $CDCl_3$) δ = 8.03 (d, *J* = 8.7 Hz, 2H), 7.88 (d, *J* = 8.7 Hz, 2H), 7.58 – 7.51 (m, 2H), 7.26 – 7.21 (m, 3H), 4.97 – 4.85 (m, 1H), 4.17 (dd, *J* = 15.3, 9.5 Hz, 1H), 3.92 (s, 3H), 3.84 (dd, *J* = 15.3, 7.0 Hz, 1H), 3.27 (dd, *J* = 12.8, 5.4 Hz, 1H), 3.06 (dd, *J* = 12.8, 7.2 Hz, 1H) ppm.

^{13}C {^{1}H} NMR (75 MHz, $CDCl_3$) δ = 166.2, 162.6, 133.4, 132.4, 131.6, 129.5, 129.2, 128.8, 128.1, 127.5, 127.0, 79.2, 60.3, 52.3, 31.8 ppm.

HRES (ESI-TOF) m/z: $[M+H]^+$ calcd for $C_{18}H_{18}NO_3Se$ 376.0461; found: 376.0452.

IR (neat) $\tilde{\nu}$ = 1716, 1641, 1276, 1261, 733, 706 cm^{-1}.

5-((Phenylselanyl)methyl)-2-(m-tolyl)-4,5-dihydrooxazole (6i)

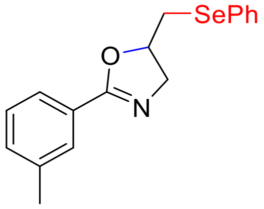

Prepared according to the general procedure GP4 from *N*-allyl-3-methylbenzamide (46 mg, 0.26 mmol) over minutes. The product was purified by column chromatography, applying (petroleum ether: ethyl acetate 8:2) as eluent to get the product as a colorless oil (60 mg, 0.18 mmol, 70%).

^{1}H NMR (300 MHz, $CDCl_3$) δ = 7.66 (s, 1H), 7.60-7.65 (m, 1H), 7.55 (dd, *J* = 6.4, 3.0 Hz, 2H), 7.25 (dd, *J* = 4.0, 2.1 Hz, 5H), 4.81-4.91 (m, 1H), 4.14 (dd, *J* = 14.9, 9.5 Hz, 1H), 3.80 (dd, *J* = 15.0, 6.9 Hz, 1H), 3.27 (dd, *J* = 12.7, 5.4 Hz, 1H), 3.03 (dd, *J* = 12.7, 7.5 Hz, 1H), 2.35 (s, 3H) ppm.

^{13}C {^{1}H} NMR (75 MHz, $CDCl_3$) δ = 163.9, 138.0, 133.4, 132.2, 129.3, 128.9, 128.7, 128.2, 127.5, 127.5, 125.3, 78.9, 60.1, 31.8, 21.1 ppm.

HRMS (ESI-TOF) m/z: $[M+H]^+$ calcd for $C_{17}H_{18}NOSe$ 332.0563; found: 332.0554.

The spectral data are in agreement with the literature.[17]

3-(5-((Phenylselanyl)methyl)-4,5-dihydrooxazol-2-yl)benzonitrile (6j)

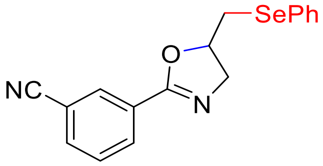

Prepared according to the general procedure GP4 from *N*-allyl-3-cyanobenzamide (48 mg, 0.26 mmol) over 35 minutes. The product was purified by column chromatography, applying (petroleum ether: ethyl acetate 7:3) as eluent to get the product as a colorless oil (60 mg, 0.17 mmol, 68%).

^{1}H NMR (300 MHz, $CDCl_3$) δ = 8.07 – 8.03 (m, 1H), 7.97 (dd, *J* = 1.6, 1.1 Hz, 1H), 7.73 – 7.69 (m, 1H), 7.57 – 7.51 (m, 2H), 7.48 (td, *J* = 7.9, 0.6 Hz, 1H), 7.31 – 7.21 (m, 3H), 4.94 (dtd, *J* = 9.6, 6.9, 5.5 Hz, 1H), 4.17 (dd, *J* = 15.3, 9.6 Hz, 1H), 3.84 (dd, *J* = 15.3, 7.0 Hz, 1H), 3.24 (dd, *J* = 13.0, 5.5 Hz, 1H), 3.09 (dd, *J* = 13.0, 6.7 Hz, 1H) ppm.

^{13}C {^{1}H} NMR (75 MHz, $CDCl_3$) δ = 161.9, 134.4, 133.4, 132.2, 131.7, 129.2, 129.2, 128.9, 128.8, 127.6, 118.0, 112.6, 79.7, 60.5, 31.8 ppm.

HRMS (ESI-TOF) m/z: $[M+H]^+$ calcd for $C_{17}H_{15}N_2OSe$ 343.0357; found: 343.0350.

IR (neat) $\tilde{\nu}$ = 1735, 1377, 1128, 815, 736 cm^{-1}.

2-(3-Nitrophenyl)-5-((phenylselanyl)methyl)-4,5-dihydrooxazole (6k)

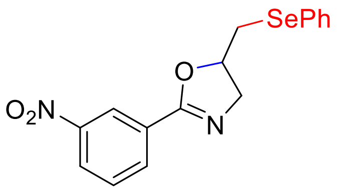

Prepared according to the general procedure GP4 from N-allyl-3-nitrobenzamide (54 mg, 0.26 mmol) over 35 minutes. The product was purified by column chromatography, applying (petroleum ether: ethyl acetate 7:3) as eluent to get the product as a colorless oil (60 mg, 0.17 mmol, 65%).

^{1}H NMR (300 MHz, $CDCl_3$) δ = 8.64 – 8.62 (m, 1H), 8.32 (ddd, *J* = 8.2, 2.3, 1.1 Hz, 1H), 8.20 – 8.15 (m, 1H), 7.62 – 7.53 (m, 3H), 7.27 (d, *J* = 1.5 Hz, 1H), 7.27 – 7.23 (m, 2H), 5.05 – 4.93 (m, 1H), 4.22 (dd, *J* = 15.3, 9.5 Hz, 1H), 3.90 (dd, *J* = 15.3, 7.0 Hz, 1H), 3.29 (dd, *J* = 13.0, 5.2 Hz, 1H), 3.14 (dd, *J* = 12.9, 7.1 Hz, 1H) ppm.

^{13}C {^{1}H} NMR (75 MHz, $CDCl_3$) δ = 161.8, 148.2, 133.8, 133.2, 129.4, 129.2, 127.6, 125.8, 123.1, 79.8, 60.1, 31.8 ppm.

HRES (ESI-TOF) m/z: $[M+H]^+$ calcd for $C_{16}H_{15}N_2O_3{}^{80}Se$ 363.0251; found: 363.0248.

IR (neat) $\tilde{\nu}$ = 1745, 1540, 1249, 745, 698 cm^{-1}.

2-(2-Methyl-5-(trifluoromethyl)phenyl)-5-((phenylselanyl)methyl)-4,5-dihydrooxazole (6l)

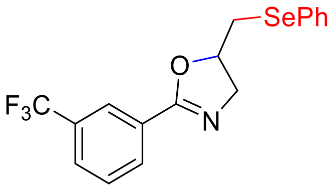

Prepared according to the general procedure GP4 from *N*-allyl-3-(trifluoromethyl)benzamide (60 mg, 0.26 mmol) over 35 minutes. The product was purified by column chromatography, applying (petroleum ether: ethyl acetate 7:3) as eluent to get the product as a colorless oil (65 mg, 0.17 mmol, 65%).

^{1}**H NMR** (300 MHz, $CDCl_3$) δ = 8.11 (s, 1H), 8.05 (d, *J* = 8.0 Hz, 1H), 7.73 (d, *J* = 7.8 Hz, 1H), 7.61 – 7.54 (m, 2H), 7.51 (d, *J* = 7.8 Hz, 1H), 7.28 (d, *J* = 2.0 Hz, 3H), 5.02 – 4.90 (m, 1H), 4.21 (dd, *J* = 15.2, 9.5 Hz, 1H), 3.88 (dd, *J* = 15.2, 7.0 Hz, 1H), 3.30 (dd, *J* = 12.9, 5.2 Hz, 1H), 3.11 (dd, *J* = 12.9, 7.2 Hz, 1H) ppm.

^{13}**C {^{1}H} NMR** (75 MHz, $CDCl_3$) δ = 162.6, 143.6, 139.6, 133.4, 131.2 (d, *J* = 20.3 Hz), 130.4, 129.3, 129.1, 128.9, 128.5, 128.4, 128.1, 127.6, 126.3, 125.1 (d, *J* = 3.8 Hz), 79.4, 60.3, 46.9, 32.0 ppm.

HRMS (ESI-TOF) m/z: $[M+H]^+$ calcd for $C_{17}H_{15}NOF_3{}^{80}Se$ 386.0271; found: 386.0269.

IR (neat) $\tilde{\nu}$ = 1651, 1377, 1161, 815, 695 cm^{-1}.

5-((Phenylselanyl)methyl)-2-(o-tolyl)-4,5-dihydrooxazole (6m)

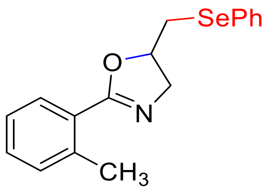

Prepared according to the general procedure GP4 from *N*-allyl-2-methylbenzamide (46 mg, 0.26 mmol) over 35 minutes. The product was purified by column chromatography, applying (petroleum ether: ethyl acetate 7:3) as eluent to get the product as a colorless oil (60 mg, 0.18 mmol, 70%).

^{1}**H NMR** (300 MHz, $CDCl_3$) δ = 7.69 (dd, *J* = 7.7, 1.4 Hz, 1H), 7.58 – 7.51 (m, 2H), 7.35 – 7.27 (m, 1H), 7.26 – 7.10 (m, 5H), 4.87 – 4.74 (m, 1H), 4.16 (dd, *J* = 15.0, 9.5 Hz, 1H), 3.88 – 3.79 (m, 1H), 3.26 (dd, *J* = 12.6, 5.4 Hz, 1H), 3.03 (dd, *J* = 12.6, 7.5 Hz, 1H), 2.56 (s, 3H) ppm.

^{13}**C {^{1}H} NMR** (75 MHz, $CDCl_3$) δ = 164.1, 138.8, 133.4, 131.2, 130.6, 129.9, 129.3, 128.9, 127.5, 126.9, 125.5, 77.8, 60.6, 31.8, 22.0 ppm.

HRMS (ESI-TOF) m/z: $[M+H]^+$ calcd for $C_{17}H_{18}NOSe$ 332.0560; found: 332.0554.

The spectral data are in agreement with the literature.[19]

2-(2-Bromophenyl)-5-((phenylselanyl)methyl)-4,5-dihydrooxazole (6n)

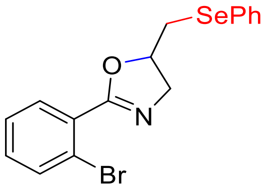

Prepared according to the general procedure GP4 from *N*-allyl-2-bromobenzamide (62 mg, 0.26 mmol) over 35 minutes. The product was purified by column chromatography, applying (petroleum ether: ethyl acetate 7:3) as eluent to get the product as a colorless oil (67 mg, 0.17 mmol, 65%).

^{1}H NMR (300 MHz, $CDCl_3$) δ = 7.64 – 7.61 (m, 1H), 7.60-7.55 (m, 3H), 7.32 (dd, *J* = 7.4, 1.6 Hz, 1H), 7.29 – 7.26 (m, 3H), 7.24 (t, *J* = 1.6 Hz, 1H), 4.94 – 4.82 (m, 1H), 4.19 (dd, *J* = 15.0, 9.5 Hz, 1H), 3.88 (dd, *J* = 15.0, 6.7 Hz, 1H), 3.32 (dd, *J* = 12.7, 5.4 Hz, 1H), 3.07 (dd, *J* = 12.7, 7.8 Hz, 1H) ppm.

^{13}C {^{1}H} NMR (75 MHz, $CDCl_3$) δ = 163.0, 133.9, 133.4, 131.7, 131.4, 129.4, 129.3, 128.7, 127.6, 127.1, 122.1, 79.1, 60.5, 31.8 ppm.

HRES (ESI-TOF) m/z: $[M+H]^+$ calcd for $C_{16}H_{15}BrNOSe$ 395.9506; found: 395.9502.

IR (neat) $\tilde{\nu}$ = 1647, 1328, 1242, 763, 690 cm^{-1}.

2-(2-Chlorophenyl)-5-((phenylselanyl)methyl)-4,5-dihydrooxazole (6o)

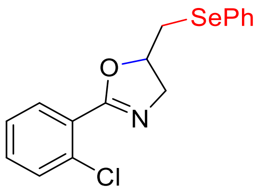

Prepared according to the general procedure GP4 from *N*-allyl-2-chlorobenzamide (51 mg, 0.26 mmol) over 35 minutes. The product was purified by column chromatography, applying (petroleum ether: ethyl acetate 7:3) as eluent to get the product as a colorless oil (70 mg, 0.2 mmol, 77%).

^{1}H NMR (300 MHz, $CDCl_3$) δ = 7.63 (d, *J* = 7.7 Hz, 1H), 7.58 – 7.50 (m, 2H), 7.41 (d, *J* = 6.6 Hz, 1H), 7.33 (td, *J* = 7.6, 1.8 Hz, 1H), 7.27 – 7.24 (m, 1H), 7.23 (ddd, *J* = 5.9, 2.6, 1.2 Hz, 3H), 4.91 – 4.79 (m, 1H), 4.18 (dd, *J* = 15.1, 9.5 Hz, 1H), 3.86 (dd, *J* = 15.1, 6.7 Hz, 1H), 3.28 (dd, *J* = 12.7, 5.4 Hz, 1H), 3.04 (dd, *J* = 12.7, 7.7 Hz, 1H) ppm.

^{13}C {^{1}H} NMR (75 MHz, $CDCl_3$) δ = 162.2, 133.5, 131.6, 131.3, 130.7, 129.3, 128.8, 128.3, 127.6, 127.2, 126.5, 79.1, 60.8, 31.9 ppm.

HRES (ESI-TOF) m/z: $[M+H]^+$ calcd for $C_{16}H_{15}ClNOSe$ 352.0005; found: 352.0007.

IR (neat) $\tilde{\nu}$ = 1647, 1477, 1327, 1031, 871,765 cm^{-1}.

2-(2-Iodophenyl)-5-((phenylselanyl)methyl)-4,5-dihydrooxazole (6p)

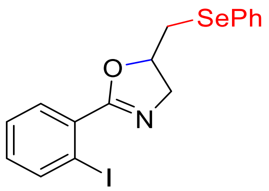

Prepared according to the general procedure GP4 from *N*-allyl-2-iodobenzamide (75 mg, 0.26 mmol) over 35 minutes. The product was purified by column chromatography, applying (petroleum ether: ethyl acetate 8:2) as eluent to get the product as a colorless oil (90 mg, 0.20 mmol, 78%).

^{1}H NMR (300 MHz, $CDCl_3$) δ = 7.92 (d, *J* = 9.1 Hz, 1H), 7.60 -7.53(m, 3H), 7.34 (t, *J* = 7.0 Hz, 1H), 7.30 – 7.25 (m, 3H), 7.09 (td, *J* = 7.8, 1.8 Hz, 1H), 4.93-4.83 (m, 1H), 4.19 (dd, *J* = 15.0, 9.5 Hz, 1H), 3.87 (dd, *J* = 15.0, 6.8 Hz, 1H), 3.34 (dd, *J* = 12.7, 5.4 Hz, 1H), 3.08 (dd, *J* = 12.7, 7.8 Hz, 1H) ppm.

^{13}C {^{1}H} NMR (75 MHz, $CDCl_3$) δ = 163.8, 140.6, 133.4, 133.2, 131.7, 130.8, 129.3, 128.7, 127.85, 127.6, 94.7, 79.3, 60.1, 31.8 ppm.

HRMS (ESI-TOF) m/z: $[M+H]^+$ calcd for $C_{16}H_{15}INOSe$ 443.9366; found: 443.9364.

The spectral data are in agreement with the literature.[20]

2-([1,1'-Biphenyl]-2-yl)-5-((phenylselanyl)methyl)-4,5-dihydrooxazole (6q)

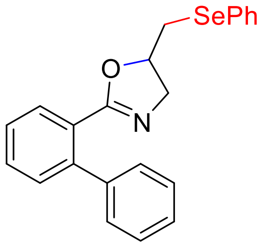

Prepared according to the general procedure GP4 from *N*-allyl-[1,1'-biphenyl]-2-carboxamide (62 mg , 0.26 mmol) over 35 minutes. The product was purified by column chromatography, applying (petroleum ether: ethyl acetate 7:3) as eluent to get the product as a colorless oil (73 mg, 0.19 mmol, 73%).

^{1}H NMR (300 MHz, $CDCl_3$) δ = 7.61 – 7.57 (m, 1H), 7.38 (dd, *J* = 7.8, 1.7 Hz, 1H), 7.36 – 7.29 (m, 2H), 7.28-7.25 (m, 5H), 7.23 (t, *J* = 1.4 Hz, 2H), 7.15 – 7.10 (m, 3H), 4.50 – 4.38 (m, 1H), 3.88 (dd, *J* = 14.8, 9.5 Hz, 1H), 3.56 (dd, *J* = 14.8, 6.7 Hz, 1H), 2.72 (dd, *J* = 12.6, 5.4 Hz, 1H), 2.58 (dd, *J* = 12.6, 8.1 Hz, 1H) ppm.

^{13}C {^{1}H} NMR (75 MHz, $CDCl_3$) δ = 162.67, 133.4, 131.6, 131.3, 130.7, 129.3, 128.8, 127.5, 127.2, 126.5, 78.7, 60.9, 31.8 ppm.

HRMS (ESI-TOF) m/z: $[M+H]^+$ calcd for $C_{22}H_{20}NOSe$ 394.0705; found: 394.0710.

IR (neat) $\tilde{\nu}$ = 1649, 1477, 1072, 1039, 821, 671, 615 cm^{-1}.

2-(Furan-2-yl)-5-((phenylselanyl)methyl)-4,5-dihydrooxazole (6r)

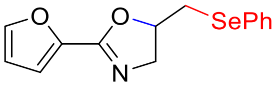

Prepared according to the general procedure GP4 from *N*-allylfuran-2-carboxamide (39 mg, 0.26 mmol) over 35 minutes. The product was purified by column chromatography, applying (petroleum ether: ethyl acetate 7:3) as eluent to get the product as a colorless oil (60 mg, 0.19 mmol, 73%).

^{1}H NMR (300 MHz, $CDCl_3$) δ = 7.63 – 7.44 (m, 3H), 7.30 – 7.21 (m, 3H), 6.85 (d, *J* = 3.2 Hz, 1H), 6.46 (dd, *J* = 3.4, 1.8 Hz, 1H), 4.92 – 4.77 (m, 1H), 4.15 (dd, *J* = 15.0, 9.4 Hz, 1H), 3.82 (dd, *J* = 15.0, 6.9 Hz, 1H), 3.27 (dd, *J* = 12.7, 5.1 Hz, 1H), 3.01 (dd, *J* = 12.7, 7.8 Hz, 1H) ppm.

^{13}C {^{1}H} NMR (75 MHz, $CDCl_3$) δ = 156.4, 145.2, 142.9, 133.4, 129.3, 128.7, 127.6, 114.8, 111.6, 79.4, 60.1, 31.9 ppm.

The spectral data are in agreement with the literature.[18]

5-((Phenylselanyl)methyl)-2-(thiophen-2-yl)-4,5-dihydrooxazole (6s)

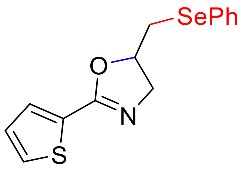

Prepared according to the general procedure GP4 from *N*-allylthiophene-2-carboxamide (43 mg, 0.26 mmol) over 35 minutes. The product was purified by column chromatography, applying (petroleum ether: ethyl acetate 7:3) as eluent to get the product as a colorless oil (54 mg, 0.19 mmol, 73%).

^{1}H NMR (300 MHz, $CDCl_3$) δ = 7.47 (d, *J* = 4.9 Hz, 1H), 7.42 (d, *J* = 6.2 Hz, 1H), 7.06 – 7.00 (m, 1H), 4.86 (m, 1H), 4.12 (dd, *J* = 14.9, 9.4 Hz, 1H), 3.78 (dd, *J* = 14.9, 6.8 Hz, 1H), 3.27 (dd, *J* = 12.7, 5.2 Hz, 1H), 3.02 (dd, *J* = 12.7, 7.8 Hz, 1H) ppm.

^{13}C {^{1}H} NMR (75 MHz, $CDCl_3$) δ = 159.5, 133.4, 130.3, 129.9, 129.2, 128.7, 127.5, 127.5, 79.4, 60.2, 31.7 ppm.
The spectral data are in agreement with the literature.[18]

5-((Phenylselanyl)methyl)-2-(pyridin-3-yl)-4,5-dihydrooxazole (6t)

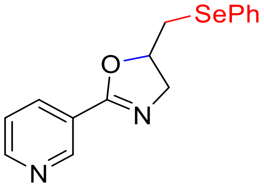

Prepared according to the general procedure GP4 from *N*-allylnicotinamide (42 mg, 0.26 mmol) over 35 minutes. The product was purified by column chromatography, applying (petroleum ether: ethyl acetate 7:3) as eluent to get the product as a colorless oil (60 mg, 0.19 mmol, 73%).

^{1}H NMR (300 MHz, $CDCl_3$) δ = 9.01 (d, *J* = 3.0 Hz, 1H), 8.67 (dd, *J* = 4.9, 1.7 Hz, 1H), 8.07 (d, *J* = 8.0 Hz, 1H), 7.55 (dd, *J* = 6.6, 2.9 Hz, 2H), 7.34 – 7.20 (m, 4H), 4.98 – 4.85 (m, 1H), 4.16 (dd, *J* = 15.2, 9.5 Hz, 1H), 3.84 (dd, *J* = 15.2, 7.0 Hz, 1H), 3.26 (dd, *J* = 12.8, 5.3 Hz, 1H), 3.07 (dd, *J* = 12.8, 7.2 Hz, 1H) ppm.

^{13}C {^{1}H} NMR (75 MHz, $CDCl_3$) δ = 161.7, 152.0, 149.4, 135.4, 133.4, 129.2, 128.8, 127.6, 123.8, 123.1, 79.3, 60.5, 31.8 ppm.

HRES (ESI-TOF) m/z: $[M+H]^+$ calcd for $C_{15}H_{15}N_2OSe$ 319.0351; found: 319.0350.

The spectral data are in agreement with the literature.[19]

2-Phenyl-6-((phenylselanyl)methyl)-5,6-dihydro-4H-1,3-oxazine (6u)

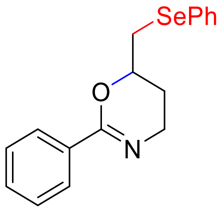

Prepared according to the general procedure GP4 from *N*-(but-3-en-1-yl) benzamide (46 mg, 0.26 mmol) over 35 minutes. The product was purified by column chromatography, applying (petroleum ether: ethyl acetate 7:3) as eluent to get the product as a colorless oil (70 mg, 0.21mmol, 81%).

^{1}H NMR (300 MHz, $CDCl_3$) δ = 7.75 (dd, *J* = 24.7, 7.7 Hz, 2H), 7.57 – 7.51 (m, 2H), 7.42 – 7.35 (m, 2H), 7.35 – 7.28 (m, 2H), 7.28 – 7.16 (m, 2H), 4.46 – 4.35 (m, 1H), 3.66 (ddd, *J* = 16.7, 5.4, 3.0 Hz, 1H), 3.60 – 3.47 (m, 1H), 3.26 (dd, *J* = 12.9, 6.4 Hz, 1H), 3.10 (dd, *J* = 12.9, 6.3 Hz, 1H), 2.16 – 2.05 (m, 1H), 1.84 – 1.70 (m, 1H) ppm.

^{13}C {^{1}H} NMR (75 MHz, $CDCl_3$) δ = 155.2, 133.7, 133.0, 130.4, 129.8, 129.3, 128.0, 127.3, 127.0, 74.1, 43.1, 32.7, 27.0 ppm.

The spectral data are in agreement with the literature.[21]

2-(4-Chlorophenyl)-6-((phenylselanyl)methyl)-5,6-dihydro-4H-1,3-oxazine (6v)

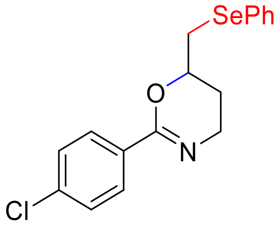

Prepared according to the general procedure GP4 from *N*-(but-3-en-1-yl)-4-chlorobenzamide (54 mg, 0.26 mmol) over 35 minutes. The product was purified by column chromatography, applying (petroleum ether: ethyl acetate 7:3) as eluent to get the product as a colorless oil (61 mg, 0.17 mmol, 65%).

^{1}H NMR (300 MHz, $CDCl_3$) δ = 7.71 (d, *J* = 8.7 Hz, 2H), 7.58 – 7.50 (m, 2H), 7.28 (d, *J* = 4.9 Hz, 2H), 7.27 – 7.22 (m, 3H), 4.46 – 4.30 (m, 1H), 3.71 – 3.61 (m, 1H), 3.53 (ddd, *J* = 12.8, 10.3, 5.0 Hz, 1H), 3.25 (dd, *J* = 12.9, 6.5 Hz, 1H), 3.09 (dd, *J* = 12.9, 6.2 Hz, 1H), 2.16 – 2.04 (m, 1H), 1.85 – 1.67 (m, 1H) ppm.

^{13}C {^{1}H} NMR (75 MHz, $CDCl_3$) δ = 154.7, 136.6, 133.0, 131.9, 129.7, 129.3, 128.4, 128.3, 127.4, 74.9, 43.1, 32.3, 26.9 ppm.

HRES (ESI-TOF) m/z: $[M+H]^+$ calcd for $C_{17}H_{17}ClNO^{80}Se$ 366.0162; found: 366.0164.

IR (neat) $\tilde{\nu}$ = 1716, 1651, 1348, 1089, 758, 663, 644 cm^{-1}.

2-Cyclohexyl-5-((phenylselanyl)methyl)-4,5-dihydrooxazole (6w)

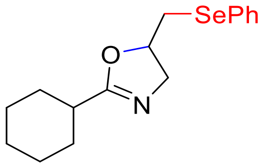

Prepared according to the general procedure GP4 from *N*-allylcyclohexanecarboxamide (43 mg, 0.26 mmol) over 35 minutes. The product was purified by column chromatography, applying (petroleum ether: ethyl acetate 7:3) as eluent to get the product as a colorless oil (50 mg, 0.15 mmol, 58%).

^{1}H NMR (300 MHz, $CDCl_3$) δ = 7.57 – 7.51 (m, 2H), 7.28 (t, *J* = 3.6,Hz, 3H), 4.60 (dq, *J* = 9.5, 6.6 Hz, 1H), 3.91 (dd, *J* = 14.3, 9.4 Hz, 1H), 3.58 (dd, *J* = 14.3, 9.4 Hz, 1H), 3.15 (dd, *J* = 12.6, 5.4 Hz, 1H), 2.94 (dd, *J* = 12.6, 7.4 Hz, 1H), 2.28 – 2.17 (m, 1H),1.91 – 1.85 (m, 2H), 1.78 – 1.63 (m, 2H), 1.47 – 1.11 (m, 6H) ppm.

^{13}C {^{1}H} NMR (75 MHz, $CDCl_3$) δ = 170.8, 133.2, 129.2, 129.0, 127.4, 77.9, 59.6, 37.6, 32.0, 29.7, 25.8, 25.6 ppm.

HRES (ESI-TOF) m/z: $[M+H]^+$ calcd for $C_{16}H_{22}NOSe$ 324.0865; found: 324.0867.

The spectral data are in agreement with the literature.[19]

2-Cyclobutyl-5-((phenylselanyl)methyl)-4,5-dihydrooxazole (6x)

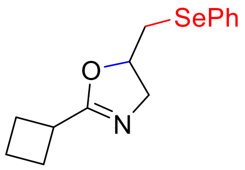

Prepared according to the general procedure GP4 from *N*-allylcyclobutanecarboxamide (36 mg, 0.26 mmol) over 35 minutes. The product was purified by column chromatography, applying (petroleum ether: ethyl acetate 8:2) as eluent to get the product as a colorless oil (50 mg, 0.17 mmol, 65%).

^{1}H NMR (300 MHz, $CDCl_3$) δ = 7.57 – 7.51 (m, 2H), 7.29 – 7.25 (m, 3H), 4.69 (dq, *J* = 9.4, 6.6 Hz, 1H), 3.93 (dd, *J* = 13.9, 9.0 Hz, 1H), 3.59 (dd, *J* = 13.5, 6.7 Hz, 1H), 3.17 (dd, *J* = 12.6, 5.3 Hz, 1H), 2.96 (dd, *J* = 12.6, 7.5 Hz, 1H), 2.34 – 2.24 (m, 1H), 2.22 – 2.10 (m, 4H), 2.05 – 1.81 (m, 2H) ppm.

^{13}C {^{1}H} NMR (75 MHz, $CDCl_3$) δ = 170.0, 133.2, 129.2, 128.9, 127.3, 78.4, 59.6, 32.8, 32.0, 25.8, 18.6 ppm.

HRES (ESI-TOF) m/z: $[M+H]^+$ calcd for $C_{14}H_{18}NOSe$ 296.0555; found: 296.0554.

IR (neat) $\tilde{\upsilon}$ = 1728, 1608, 1244, 740, 689 cm^{-1}.

2-Cyclopropyl-5-((phenylselanyl)methyl)-4,5-dihydrooxazole (6y)

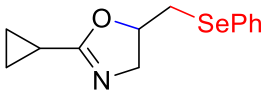

Prepared according to the general procedure GP4 from *N*-allylcyclopropanecarboxamide (33 mg, 0.26 mmol) over 35 minutes. The product was purified by column chromatography, applying (petroleum ether: ethyl acetate 7:3) as eluent to get the product as a colorless oil (40 mg, 0.14 mmol, 54%).

^{1}H NMR (300 MHz, $CDCl_3$) δ = 7.56 – 7.48 (m, 2H), 7.29 – 7.23 (m, 3H), 4.70 – 4.56 (m, 1H), 3.88 (dd, *J* = 14.2, 9.4 Hz, 1H), 3.54 (dd, *J* = 14.2, 6.7 Hz, 1H), 3.13 (dd, *J* = 12.6, 5.5 Hz, 1H), 2.92 (dd, *J* = 12.6, 7.4 Hz, 1H), 1.62 – 1.53 (m, 1H), 0.95 – 0.85 (m, 1H), 0.85 – 0.73 (m, 3H) ppm.

^{13}C {^{1}H} NMR (75 MHz, $CDCl_3$) δ = 168.4, 133.2, 129.2, 128.9, 127.4, 78.3, 59.6, 31.9, 8.6, 6.8, 6.7 ppm.

The spectral data are in agreement with the literature.[19]

2-(*tert*-Butyl)-5-((phenylselanyl)methyl)-4,5-dihydrooxazole (6z)

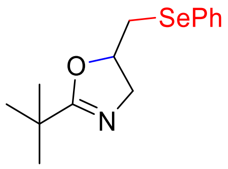

Prepared according to the general procedure GP4 from *N*-allylpivalamide (37 mg, 0.26 mmol) over 35 minutes. The product was purified by column chromatography, applying (petroleum ether: ethyl acetate 7:3) as eluent to get the product as a colorless oil (50 mg, 0.17 mmol, 65%).

^{1}H NMR (300 MHz, $CDCl_3$) δ = 7.57 – 7.51 (m, 2H), 7.28 – 7.24 (m, 3H), 4.73 – 4.57 (m, 1H), 3.90 (dd, *J* = 14.4, 9.4 Hz, 1H), 3.58 (dd, *J* = 14.4, 6.5 Hz, 1H), 3.15 (dd, *J* = 12.5, 5.2 Hz, 1H), 2.91 (dd, *J* = 12.5, 7.7 Hz, 1H), 1.17 (s, 9H) ppm.

^{13}C {^{1}H} NMR (75 MHz, $CDCl_3$) δ = 174.0, 133.2, 129.2, 127.4, 78.6, 59.7, 33.0, 31.4, 27.9 ppm.

HRES (ESI-TOF) m/z: $[M+H]^+$ calcd for $C_{14}H_{20}NO^{80}Se$ 298.0711; found: 298.0710.

IR (neat) $\tilde{\nu}$ = 1631, 1477, 1070, 734, 690 cm^{-1}.

2-(Naphthalen-2-yl)-5-((phenylselanyl)methyl)-4,5-dihydrooxazole (6aa)

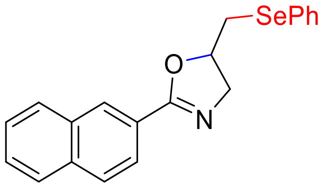

Prepared according to the general procedure GP4 from *N*-allyl-2-naphthamide (55 mg, 0.26 mmol) over 35 minutes. The product was purified by column chromatography, applying (petroleum ether: ethyl acetate 7:3) as eluent to get the product as a colorless oil (50 mg, 0.14 mmol, 54%).

^{1}H NMR (300 MHz, $CDCl_3$) δ = 9.12 (s, 1H), 7.95 (dd, *J* = 7.3, 1.3 Hz, 1H), 7.91- 7.83 (m, 2H), 7.63 – 7.39 (m, 5H), 7.29 – 7.21 (m, 3H), 4.94 – 4.82 (m, 1H), 4.30 (dd, *J* = 15.1, 9.5 Hz, 1H), 3.97 (dd, *J* = 15.1, 6.8 Hz, 1H), 3.32 (dd, *J* = 12.6, 5.4 Hz, 1H), 3.10 (dd, *J* = 12.6, 7.4 Hz, 1H) ppm.

^{13}C {^{1}H} NMR (75 MHz, $CDCl_3$) δ = 163.5, 133.7, 133.4, 132.0, 131.1, 129.3, 129.1, 128.9, 128.5, 127.6, 127.4, 126.4, 126.1, 124.6, 124.3, 77.6, 61.0, 32.1 ppm.

HRMS (ESI-TOF) m/z: $[M+H]^+$ calcd for $C_{20}H_{18}NOSe$ 368.0556; found: 368.0554.

The spectral data are in agreement with the literature.[19]

5-Methyl-2-phenyl-5-((phenylselanyl)methyl)-4,5-dihydrooxazole (6ab)

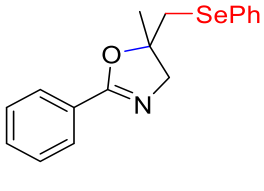

Prepared according to the general procedure GP4 from *N*-(2-methylallyl) benzamide (46 mg, 0.26 mmol) over 35 minutes. The product was purified by column chromatography, applying (petroleum ether: ethyl acetate 7:3) as eluent to get the product as a colorless oil (65 mg, 0.19 mmol, 73%).

^{1}H NMR (300 MHz, $CDCl_3$) δ = 7.88 – 7.83 (m, 2H), 7.59 – 7.54 (m, 2H), 7.51 – 7.45 (m, 1H), 7.42 – 7.36 (m, 2H), 7.28 – 7.22 (m, 3H), 4.05 (d, *J* = 14.8 Hz, 1H), 3.83 (d, *J* = 14.8 Hz, 1H), 3.31 (s, 2H), 1.61 (s, 3H) ppm.

^{13}C {^{1}H} NMR (75 MHz, $CDCl_3$) δ = 163.1, 133.0, 131.2, 130.3, 129.1, 128.2, 128.1, 127.9, 127.2, 85.7, 65.6, 38.5, 26.3 ppm.

The spectral data are in agreement with the literature.[19]

2-(4-Chlorophenyl)-5-methyl-5-((phenylselanyl)methyl)-4,5-dihydrooxazole (6ac)

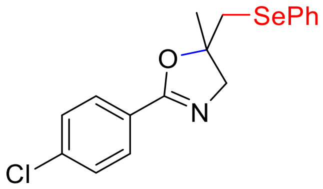

Prepared according to the general procedure GP4 from 4-chloro-*N*-(2-methylallyl) benzamide (54 mg, 0.26 mmol) over 35 minutes. The product was purified by column chromatography, applying (petroleum ether: ethyl acetate 7:3) as eluent to get the product as a colorless oil (60 mg, 0.17 mmol, 65%).

^{1}H NMR (300 MHz, $CDCl_3$) δ = 7.71 (d, *J* = 8.8 Hz, 2H), 7.55 – 7.50 (m, 2H), 7.32 (d, *J* = 8.8 Hz, 2H), 7.22 – 7.19 (m, 3H), 4.01 (dd, *J* = 14.9, 2.8 Hz, 1H), 3.79 (dd, *J* = 14.9, 3.1 Hz, 1H), 3.26 (s, 2H), 1.57 (s, 3H) ppm.

^{13}C {^{1}H} NMR (75 MHz, $CDCl_3$) δ = 162.1, 137.4, 133.1, 130.2, 129.4, 129.1, 128.5, 127.2, 126.3, 86.2, 65.6, 38.4, 26.1 ppm.

HRES (ESI-TOF) m/z: $[M+H]^+$ calcd for $C_{17}H_{17}ClNOSe$ 366.0160; found: 366.0164.

IR (neat) $\tilde{\nu}$ = 1647, 1577, 1259, 1091, 723, 690 cm^{-1}.

2-Phenyl-5-((phenylselanyl)methylene)-4,5-dihydrooxazole (6ad)

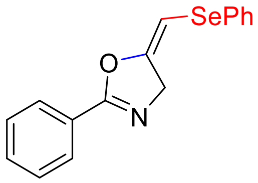

Prepared according to the general procedure GP4 from *N*-(prop-2-yn-1-yl) benzamide (41 mg, 0.26 mmol) over 35 minutes. The product was purified by column chromatography, applying (petroleum ether: ethyl acetate 7:3) as eluent to get the product as a yellow oil (70 mg, 0.18 mmol, 70%).

^{1}H NMR (300 MHz, $CDCl_3$) δ = 8.02 – 7.92 (m, 2H), 7.52 (dd, *J* = 8.0, 2.1 Hz, 1H), 7.48 – 7.38 (m, 4H), 7.30 – 7.17 (m, 3H), 6.20 (t, *J* = 3.0 Hz, 1H), 4.76 (d, *J* = 3.0 Hz, 2H) ppm.

^{13}C {^{1}H} NMR (75 MHz, $CDCl_3$) δ = 163.7, 161.8, 139.3, 132.0, 129.6, 129.3, 128.6, 128.0, 127.7, 126.4, 85.2, 58.9 ppm.

The spectral data are in agreement with the literature.[20]

5-(((4-Methoxyphenyl)selanyl)methyl)-2-phenyl-4,5-dihydrooxazole (6ae)

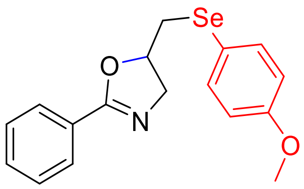

Prepared according to the general procedure GP4 from *N*-allylbenzamide (42 mg, 0.26 mmol) over 35 minutes. The product was purified by column chromatography, applying (petroleum ether: ethyl acetate 7:3) as eluent to get the product as a colorless oil (70 mg, 0.20 mmol, 77%).

^{1}H NMR (300 MHz, $CDCl_3$) δ = 7.85 (d, *J* = 6.9 Hz, 2H), 7.52 (d, *J* = 8.9 Hz, 2H), 7.45 (d, *J* = 7.2 Hz, 1H), 7.44 – 7.34 (m, 2H), 6.80 (d, *J* = 8.9 Hz, 2H), 4.91 – 4.77 (m, 1H), 4.14 (dd, *J* = 15.0, 9.5 Hz, 1H), 3.89 – 3.75 (m, 4H), 3.18 (dd, *J* = 12.6, 5.4 Hz, 1H), 2.95 (dd, *J* = 12.6, 7.6 Hz, 1H) ppm.

^{13}C {^{1}H} NMR (75 MHz, $CDCl_3$) δ = 163.9, 159.7, 136.3, 131.3, 128.3, 128.1, 127.7, 118.6, 114.9, 79.0, 60.5, 55.0, 32.7 ppm.

The spectral data are in agreement with the literature.[20]

2-Phenyl-5-((p-tolylselanyl)methyl)-4,5-dihydrooxazole (6af)

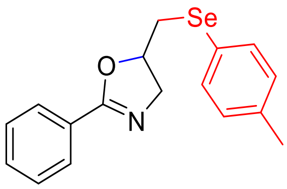

Prepared according to the general procedure GP4 from *N*-allylbenzamide (42 mg, 0.26 mmol) over 35 minutes. The product was purified by column chromatography, applying (petroleum ether: ethyl acetate 7:3) as eluent to get the product as a colorless oil (65 mg, 0.19 mmol, 73%).

^{1}H NMR (300 MHz, $CDCl_3$) δ = 7.89 – 7.82 (m, 2H), 7.51 – 7.43 (m, 3H), 7.38 (t, *J* = 7.3 Hz, 2H), 7.08 (d, *J* = 7.8 Hz, 2H), 4.92 – 4.79 (m, 1H), 4.14 (dd, *J* = 15.0, 9.5 Hz, 1H), 3.81 (dd, *J* = 15.0, 6.9 Hz, 1H), 3.24 (dd, *J* = 12.6, 5.4 Hz, 1H), 2.99 (dd, *J* = 12.6, 7.6 Hz, 1H), 2.32 (s, 3H) ppm.

^{13}C {^{1}H} NMR (75 MHz, $CDCl_3$) δ = 163.5, 137.7, 133.9, 131.3, 130.1, 128.3, 128.2, 127.6, 124.9, 79.1, 60.2, 32.2, 21.1 ppm.

The spectral data are in agreement with the literature.[20]

5-((Benzylselanyl)methyl)-2-phenyl-4,5-dihydrooxazole (6ag)

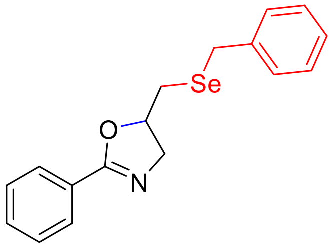

Prepared according to the general procedure GP4 from *N*-allylbenzamide (42 mg, 0.26 mmol) over 35 minutes. The product was purified by column chromatography, applying (petroleum ether: ethyl acetate 7:3) as eluent to get the product as a colorless oil (65 mg, 0.18 mmol, 69%).

^{1}H NMR (300 MHz, $CDCl_3$) δ = 7.92 (d, *J* = 6.9 Hz, 2H), 7.50 – 7.34 (m, 3H), 7.31 – 7.15 (m, 5H), 4.81 (ddd, *J* = 12.8, 9.5, 6.9 Hz, 1H), 4.11 (dd, *J* = 14.9, 9.5 Hz, 1H), 3.85 (s, 2H), 3.72 (dd, *J* = 14.9, 7.2 Hz, 1H), 2.80 (dd, *J* = 12.9, 5.7 Hz, 1H), 2.69 (dd, *J* = 13.0, 6.7 Hz, 1H) ppm.

^{13}C {^{1}H} NMR (75 MHz, $CDCl_3$) δ = 163.9, 138.6, 131.4, 128.9, 128.6, 128.4, 128.2, 127.6, 127.0, 79.8, 60.8, 28.2 ppm.

HRES (ESI-TOF) m/z: $[M+H]^+$ calcd for $C_{17}H_{18}NO^{80}Se$ 332.0560; found: 332.0554.

IR (neat) $\tilde{\nu}$ = 1645, 1577, 1267, 760, 696 cm^{-1}.

3-Phenyl-5-((phenylselanyl)methyl)-4,5-dihydroisoxazole (8a)

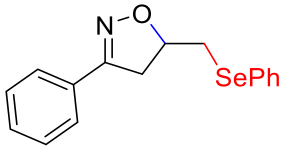

Prepared according to the general procedure GP5 from (*E*)-1-phenylbut-3-en-1-one oxime (42 mg, 0.26 mmol) over 35 minutes. The product was purified by column chromatography, applying (petroleum ether: ethyl acetate 7:3) as eluent to get the product as a colorless oil (70 mg, 0.22 mmol, 85%).

^{1}H NMR (300 MHz, $CDCl_3$) δ 7.64 (dd, *J* = 6.9, 2.9 Hz, 2H), 7.56 (dd, *J* = 6.5, 3.0 Hz, 2H), 7.40 (dd, *J* = 5.1, 2.0 Hz, 3H), 7.32 – 7.26 (m, 3H), 4.96– 4.85 (m, 1H), 3.45 (dd, *J* = 16.8, 10.2 Hz, 1H), 3.31 (dd, *J* = 12.6, 4.5 Hz, 1H), 3.20 (dd, *J* = 16.8, 6.8 Hz, 1H), 2.99 (dd, *J* = 12.6, 9.1 Hz, 1H) ppm.

^{13}C {^{1}H} NMR (75 MHz, $CDCl_3$) δ 156.4, 133.3, 130.2, 129.4, 129.4, 128.7, 128.7, 127.6, 126.7, 80.4, 40.1, 31.5 ppm.

The spectral data are in agreement with the literature.[22]

3-(4-Methoxyphenyl)-5-((phenylselanyl)methyl)-4,5-dihydroisoxazole (8b)

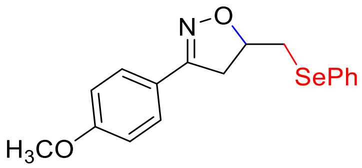

Prepared according to the general procedure GP5 from (*E*)-1-(4-methoxyphenyl)but-3-en-1-one oxime (50 mg, 0.26 mmol) over 35 minutes. The product was purified by column chromatography, applying (petroleum ether: ethyl acetate 7:3) as eluent to get the product as a colorless oil (75 mg, 0.21 mmol, 81%).

^{1}H NMR (500 MHz, $CDCl_3$) δ 7.59 – 7.53 (m, 4H), 7.31 – 7.24 (m, 3H), 6.94 – 6.88 (m, 2H), 4.90 – 4.82 (m, 1H), 3.84 (s, 3H), 3.41 (dd, *J* = 16.7, 10.2 Hz, 1H), 3.30 (dd, *J* = 12.6, 4.5 Hz, 1H), 3.17 (dd, *J* = 17.4, 7.4 Hz, 1H), 2.98 (dd, *J* = 12.6, 9.1 Hz, 1H) ppm.

^{13}C {^{1}H} NMR (126 MHz, $CDCl_3$) δ 161.1, 155.9, 133.6, 129.3, 128.8, 128.3, 127.6, 122.2, 114.3, 80.2, 55.4, 40.1, 31.5 ppm.

The spectral data are in agreement with the literature.[20]

5-((phenylselanyl)methyl)-3-(*p*-tolyl)-4,5-dihydroisoxazole (8c)

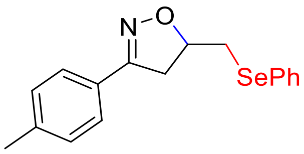

Prepared according to the general procedure GP5 from (*E*)-1-(p-tolyl)but-3-en-1-one oxime (46 mg, 0.26 mmol) over 35 minutes. The product was purified by column chromatography, applying (petroleum ether: ethyl acetate 7:3) as eluent to get the product as a colorless oil (71 mg, 0.21 mmol, 81%).

^{1}H NMR (300 MHz, CDCl$_3$) δ 7.60 – 7.49 (m, 4H), 7.33 – 7.24 (m, 3H), 7.20 (d, *J* = 8.0 Hz, 2H), 4.94 – 4.82 (m, 1H), 3.42 (dd, *J* = 16.8, 10.2 Hz, 1H), 3.31 (dd, *J* = 12.5, 4.5 Hz, 1H), 3.18 (dd, *J* = 16.8, 6.8 Hz, 1H), 2.98 (dd, *J* = 12.5, 9.1 Hz, 1H), 2.38 (s, 3H) ppm.

^{13}C {^{1}H} NMR (75 MHz, CDCl$_3$) δ 155.9, 140.3, 133.2, 129.4, 129.3, 128.7, 127.5, 126.6, 126.61, 80.5, 40.5, 31.4, 21.6 ppm.

The spectral data are in agreement with the literature.[22]

3-(4-Fluorophenyl)-5-((phenylselanyl)methyl)-4,5-dihydroisoxazole (8d)

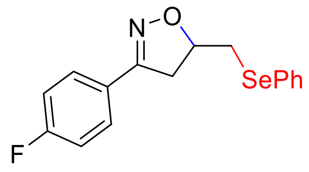

Prepared according to the general procedure GP5 from (*E*)-1-(4-fluorophenyl)but-3-en-1-one oxime (47 mg, 0.26 mmol) over 35 minutes. The product was purified by column chromatography, applying (petroleum ether: ethyl acetate 7:3) as eluent to get the product as a colorless oil (60 mg, 0.18 mmol, 70%).

^{1}H NMR (300 MHz, CDCl$_3$) δ 7.65 – 7.59 (m, 2H), 7.56 (dd, *J* = 6.5, 2.9 Hz, 2H), 7.29 (dd, *J* = 5.5, 2.3 Hz, 3H), 7.08 (dd, *J* = 7.8, 5.3 Hz, 2H), 4. 96– 4.85 (m, 1H), 3.42 (dd, *J* = 16.8, 10.2 Hz, 1H), 3.31 (dd, *J* = 12.6, 4.5 Hz, 1H), 3.17 (dd, *J* = 16.8, 6.9 Hz, 1H), 2.99 (dd, *J* = 12.6, 9.1 Hz, 1H) ppm.

^{13}C {^{1}H} NMR (75 MHz, CDCl$_3$) δ 163.8 (d, *J* = 250.9 Hz), 155.2 , 133.3, 129.4, 128.7, 128.6, 127.6, 125.7, 115.9 (d, *J* = 22.0 Hz), 80.5, 40.1, 31.3 ppm.

The spectral data are in agreement with the literature.[23]

3-(4-Bromophenyl)-5-((phenylselanyl)methyl)-4,5-dihydroisoxazole (8e)

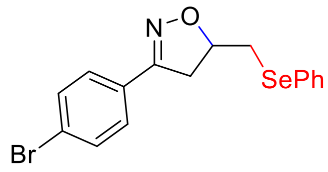

Prepared according to the general procedure GP5 from (*E*)-1-(4-bromophenyl)but-3-en-1-one oxime (62 mg, 0.26 mmol) over 35 minutes. The product was purified by column chromatography, applying (petroleum ether: ethyl acetate 7:3) as eluent to get the product as a colorless oil (80 mg, 0.20 mmol, 77%).

^{1}H NMR (300 MHz, $CDCl_3$) δ 7.58 – 7.53 (m, 2H), 7.49 (dd, *J* = 9.8, 3.3 Hz, 4H), 7.28 (dd, *J* = 6.1, 2.6 Hz, 3H), 4.96-4.86 (m, 1H), 3.41 (dd, *J* = 16.9, 10.3 Hz, 1H), 3.31 (dd, *J* = 12.6, 4.5 Hz, 1H), 3.16 (dd, *J* = 16.8, 6.9 Hz, 1H), 2.99 (dd, *J* = 12.6, 9.1 Hz, 1H) ppm.

^{13}C {^{1}H} NMR (75 MHz, $CDCl_3$) δ 155.6, 133.3, 132.0, 129.4, 128.6, 128.4, 128.2, 127.7, 124.4, 80.7, 40.2, 31.1 ppm.

The spectral data are in agreement with the literature.[23]

3-(3-Methoxyphenyl)-5-((phenylselanyl)methyl)-4,5-dihydroisoxazole (8f)

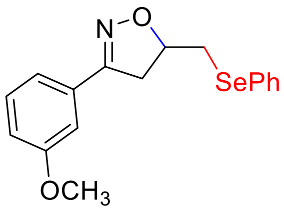

Prepared according to the general procedure GP5 from (*E*)-1-(3-methoxyphenyl)but-3-en-1-one oxime (50 mg, 0.26 mmol) over 35 minutes. The product was purified by column chromatography, applying (petroleum ether: ethyl acetate 7:3) as eluent to get the product as a colorless oil (80 mg, 0.23 mmol, 88%).

^{1}H NMR (300 MHz, $CDCl_3$) δ 7.61 – 7.52 (m, 2H), 7.35 – 7.23 (m, 5H), 7.14 (dd, *J* = 7.6, 2.5 Hz,1H), 6.97 – 6.93 (m, 1H), 4.98 – 4.83 (m, 1H), 3.83 (s, 3H), 3.43 (dd, *J* = 16.9, 10.3 Hz, 1H), 3.31 (dd, *J* = 12.6, 4.5 Hz, 1H), 3.18 (dd, *J* = 16.9, 6.8 Hz, 1H), 2.99 (dd, *J* = 12.6, 9.1 Hz, 1H) ppm.

^{13}C {^{1}H} NMR (75 MHz, $CDCl_3$) δ 159.9, 155.9, 133.2, 130.6, 129.7, 129.3, 128.7, 127.6, 119.4, 116.7, 111.2, 80.5, 55.3, 40.5, 31.5 ppm.

The spectral data are in agreement with the literature.[20]

2-Phenyl-5-((phenylthio)methyl)-4,5-dihydrooxazole (9a)

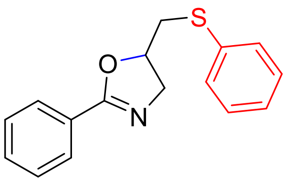

Prepared according to the general procedure GP4 from *N*-allylbenzamide (42 mg, 0.26 mmol) and using diphenyl disulfide (87 mg, 0.4 mmol) instead of diphenyl diselenide over 35 minutes. The product was purified by column chromatography, applying (petroleum ether: ethyl acetate 7:3) as eluent to get the product as a colorless oil (46 mg, 0.168 mmol, 65%).

^{1}H NMR (300 MHz, $CDCl_3$) δ = 7.83 (dd, *J* = 8.4, 1.4 Hz, 2H), 7.47 – 7.32 (m, 5H), 7.30 – 7.22 (m, 2H), 7.21 (t, *J* = 1.4 Hz, 1H), 4.82 (dtd, *J* = 9.5, 6.9, 5.5 Hz, 1H), 4.13 (dd, *J* = 15.0, 9.5 Hz, 1H), 3.86 (dd, *J* = 15.0, 6.8 Hz, 1H), 3.30 (dd, *J* = 13.7, 5.5 Hz, 1H), 3.04 (dd, *J* = 13.7, 7.1 Hz, 1H) ppm.

^{13}C {^{1}H} NMR (75 MHz, $CDCl_3$) δ = 163.7, 134.9, 131.3, 130.3, 129.1, 128.3, 128.1, 127.5, 126.8, 78.1, 59.6, 38.5 ppm.

The spectral data are in agreement with the literature.[20]

^{1}H NMR (300 MHz, $CDCl_3$) of *N*-allylbenzamide (5a)

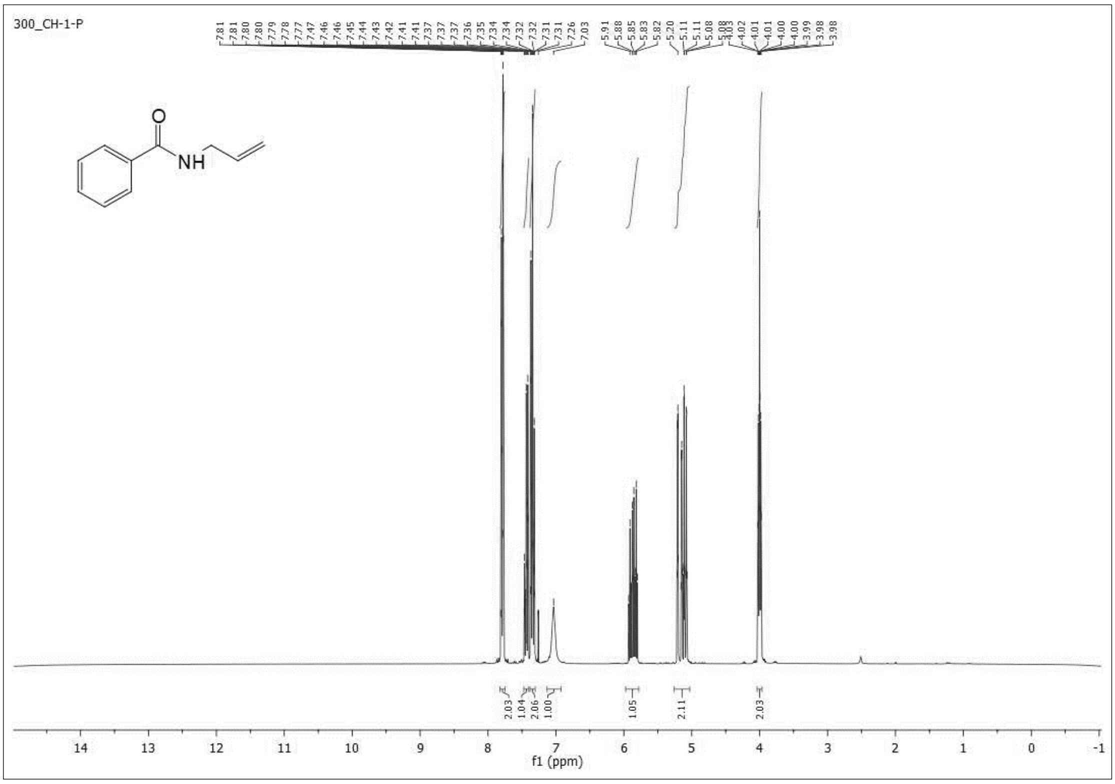

^{13}C {^{1}H} NMR (75 MHz, $CDCl_3$) of *N*-allylbenzamide (5a)

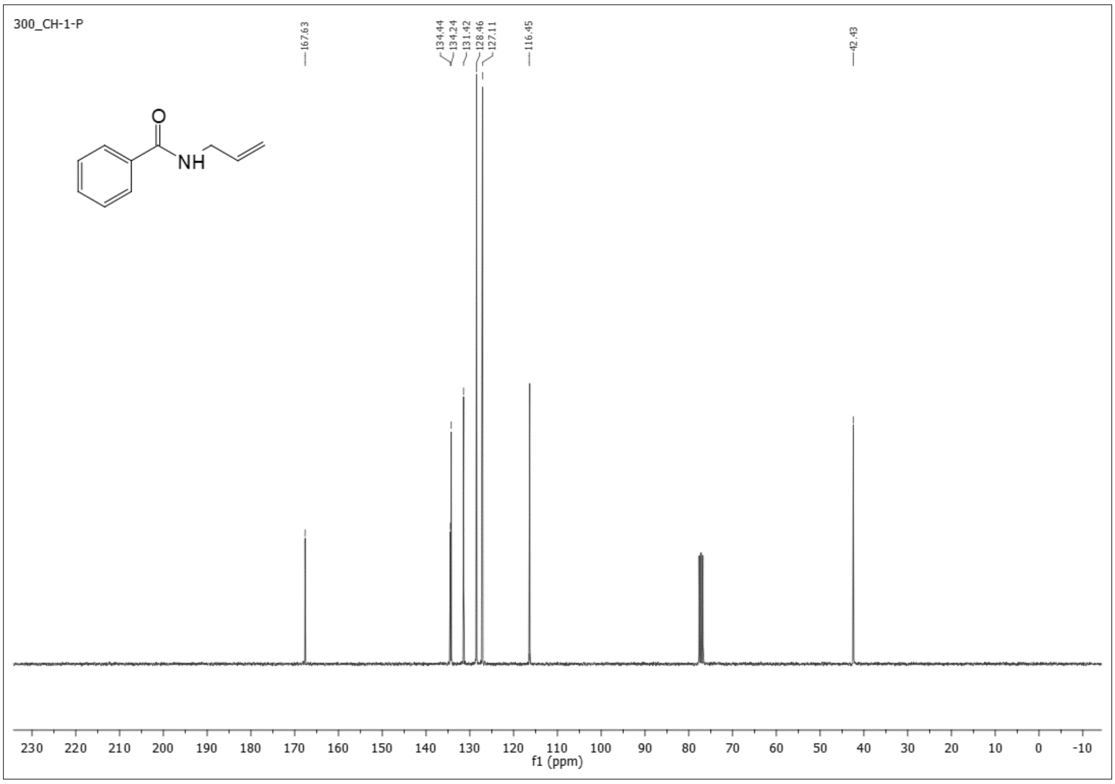

^{1}H NMR (300 MHz, CDCl$_3$) of *N*-allyl-4-(trifluoromethyl)benzamide (5b)

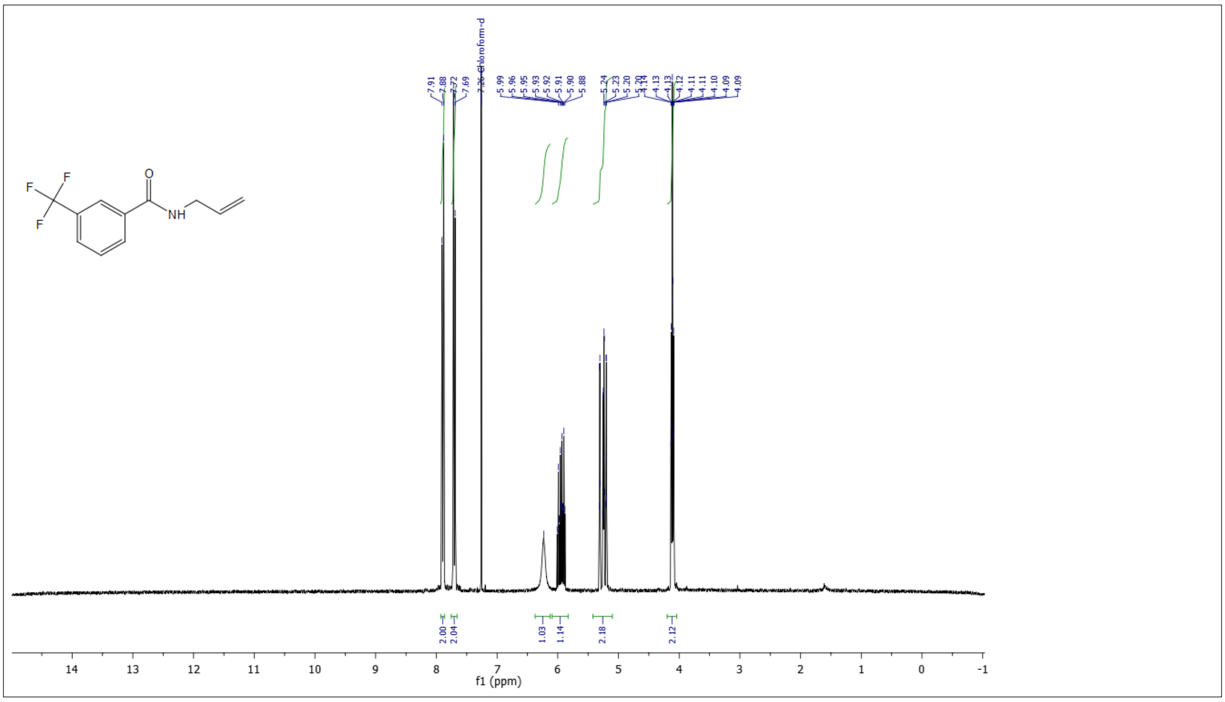

^{13}C {^{1}H} NMR (75 MHz, CDCl$_3$) of *N*-allyl-4-(trifluoromethyl)benzamide (5b)

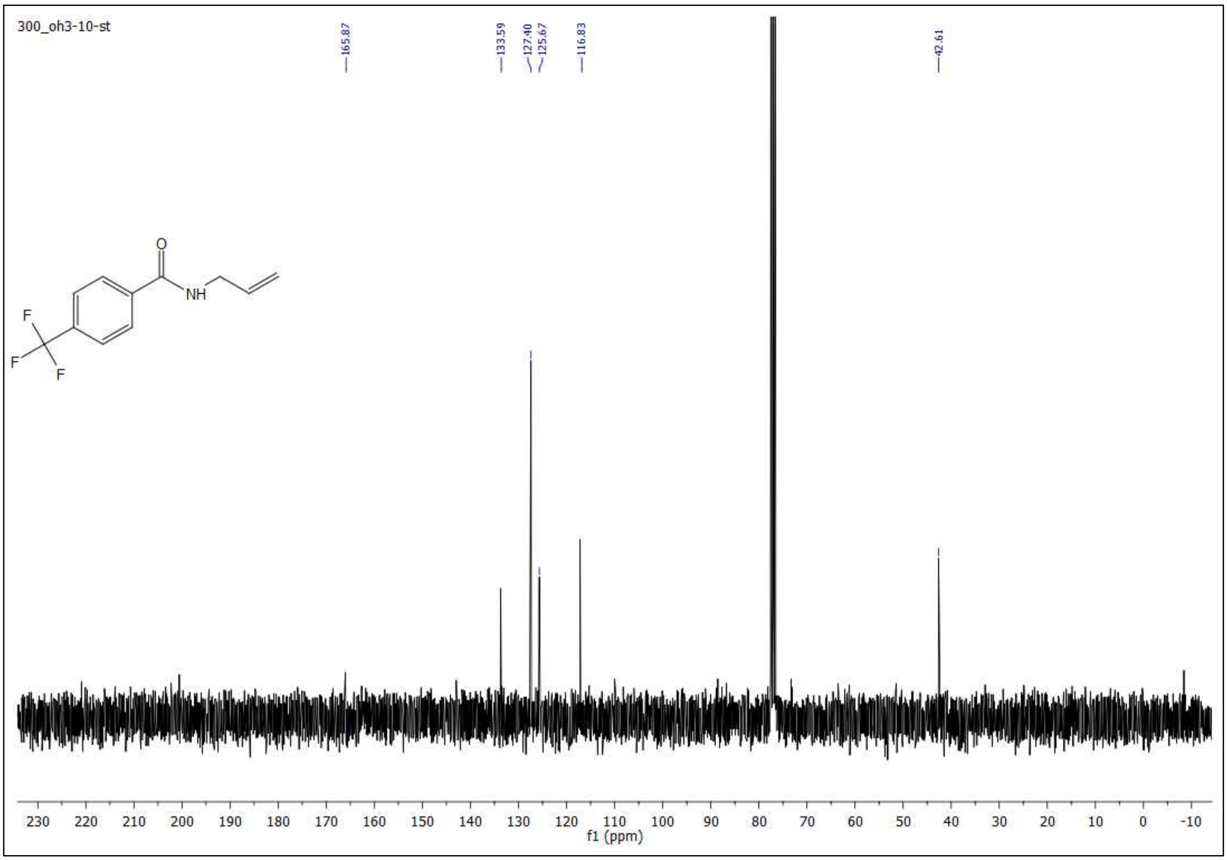

^{1}H NMR (300 MHz, $CDCl_3$) of *N*-allyl-4-iodobenzamide (5c)

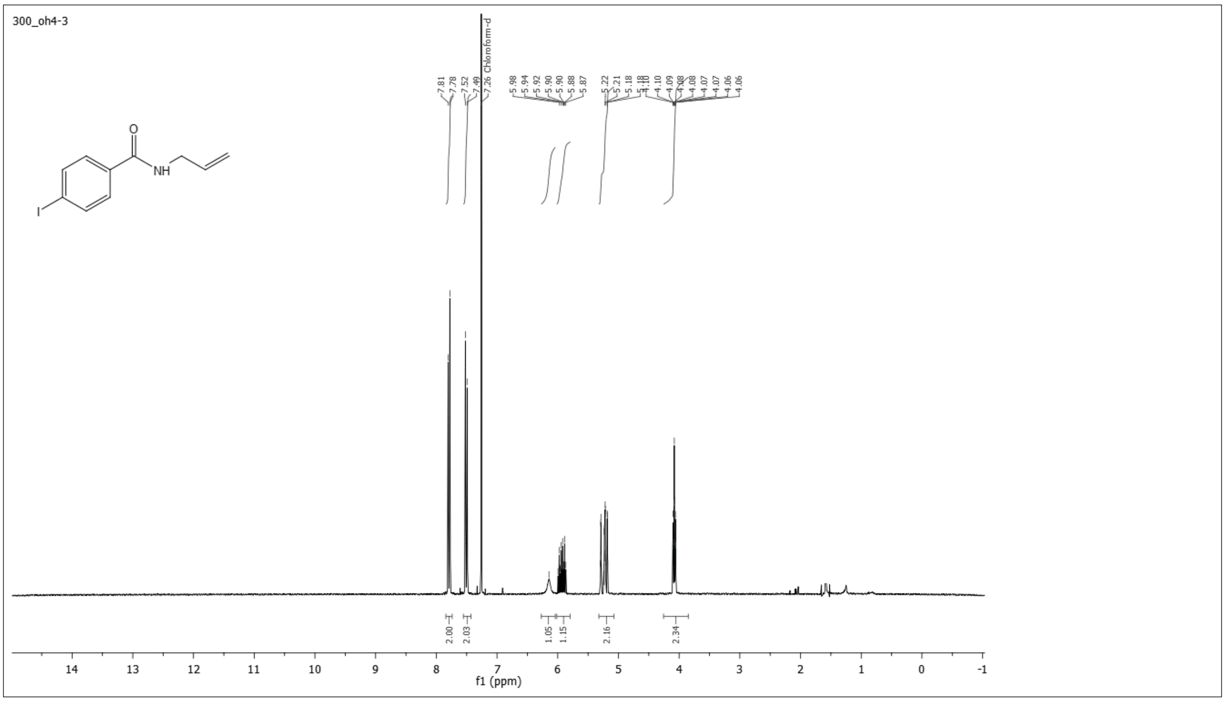

$^{13}C\ \{^1H\}$ NMR (75 MHz, $CDCl_3$) of *N*-allyl-4-iodobenzamide (5c)

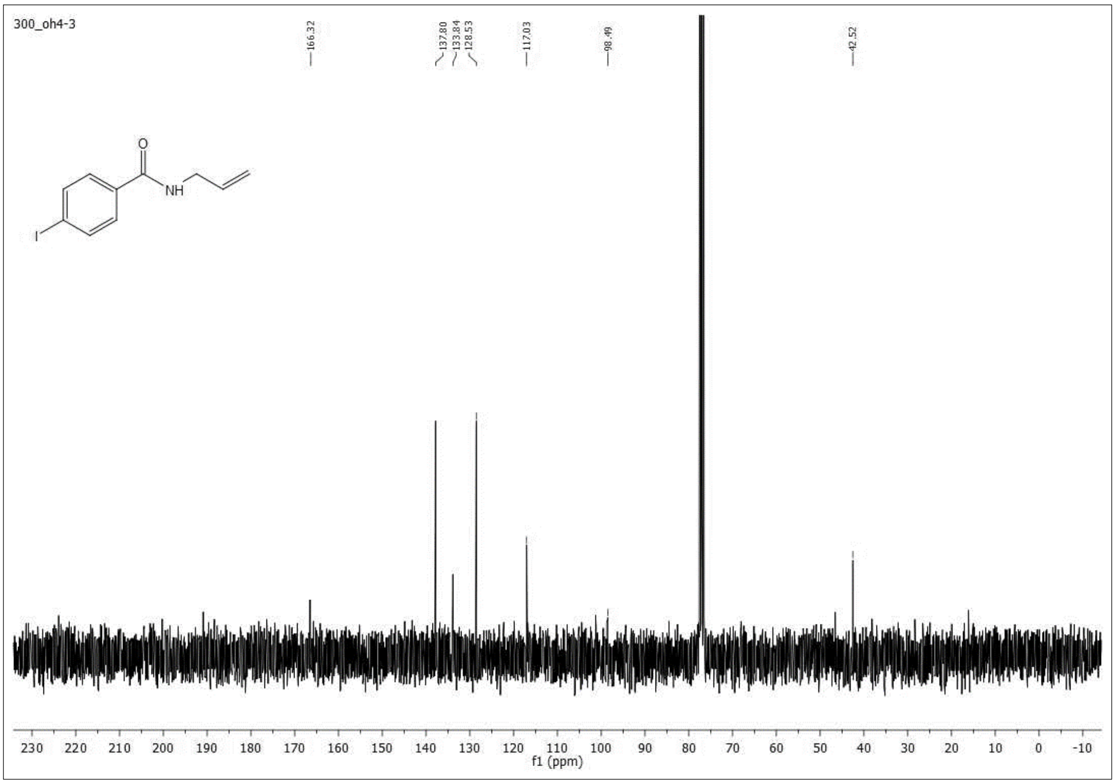

^{1}H NMR (300 MHz, $CDCl_3$) of *N*-allyl-4-bromobenzamide (5d)

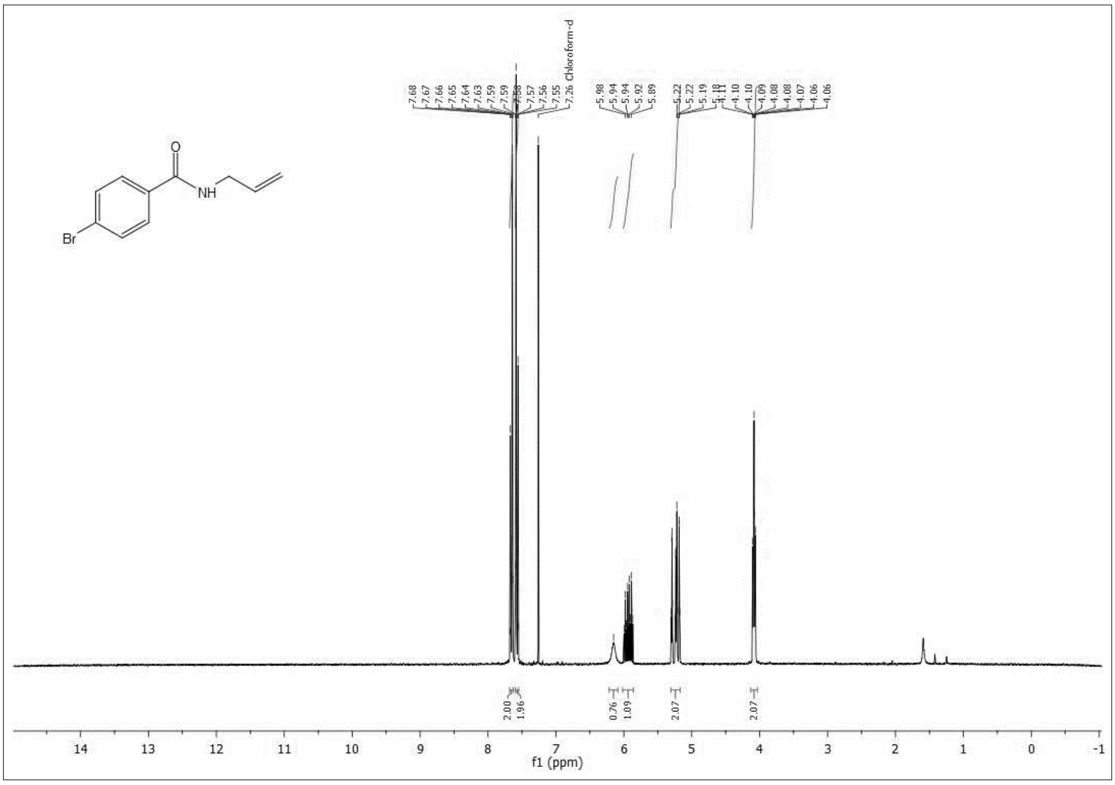

^{13}C {^{1}H} NMR (75 MHz, $CDCl_3$) of *N*-allyl-4-bromobenzamide (5d)

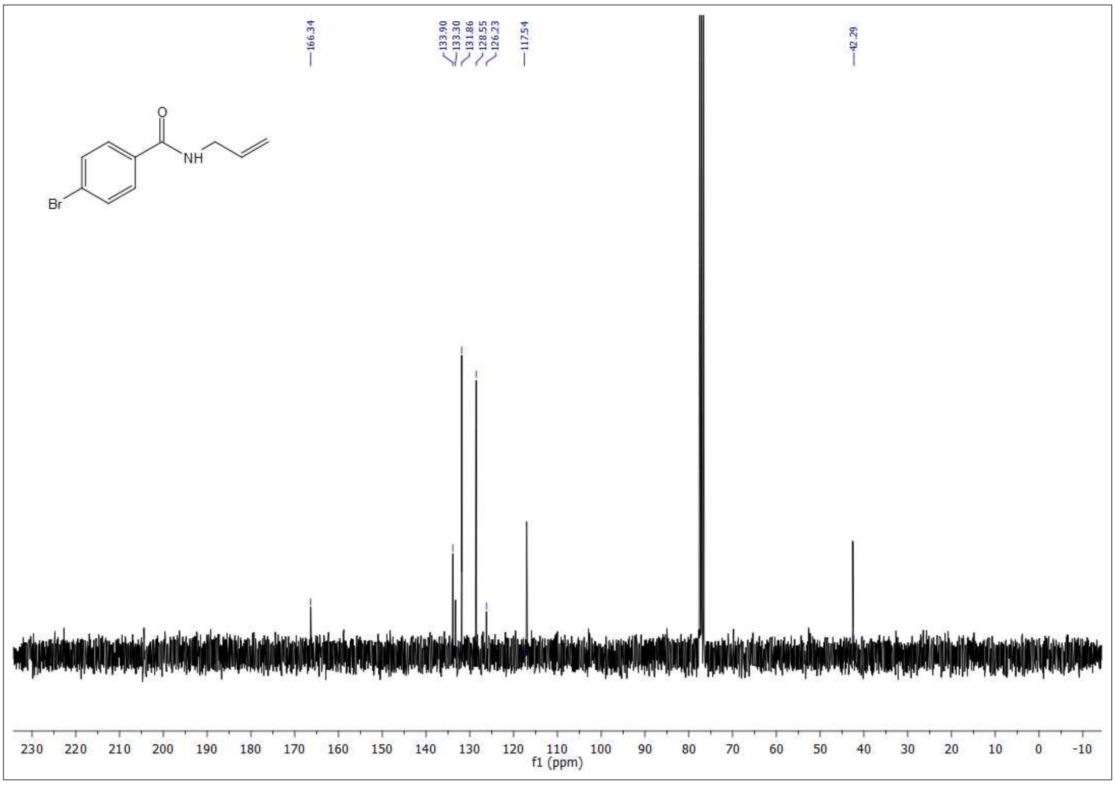

^{1}H NMR (300 MHz, $CDCl_3$) of *N*-allyl-4-chlorobenzamide (5e)

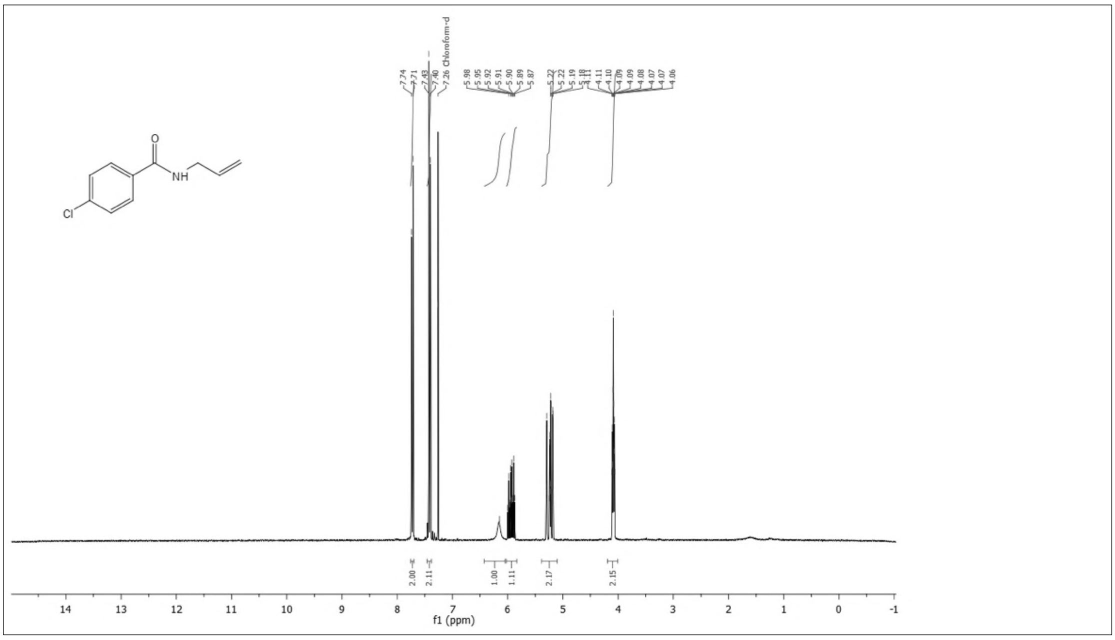

^{13}C {^{1}H} NMR (75 MHz, $CDCl_3$) of *N*-allyl-4-chlorobenzamide (5e)

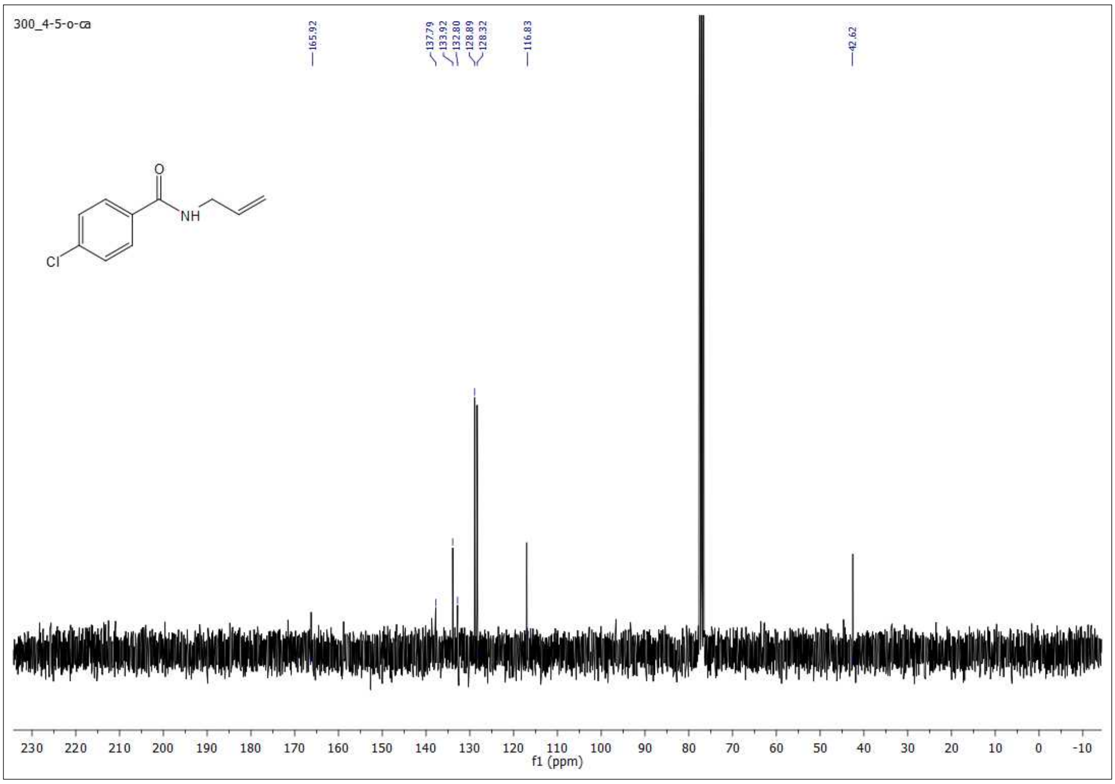

^{1}H NMR (300 MHz, $CDCl_3$) of *N*-allyl-4-methoxybenzamide (5f)

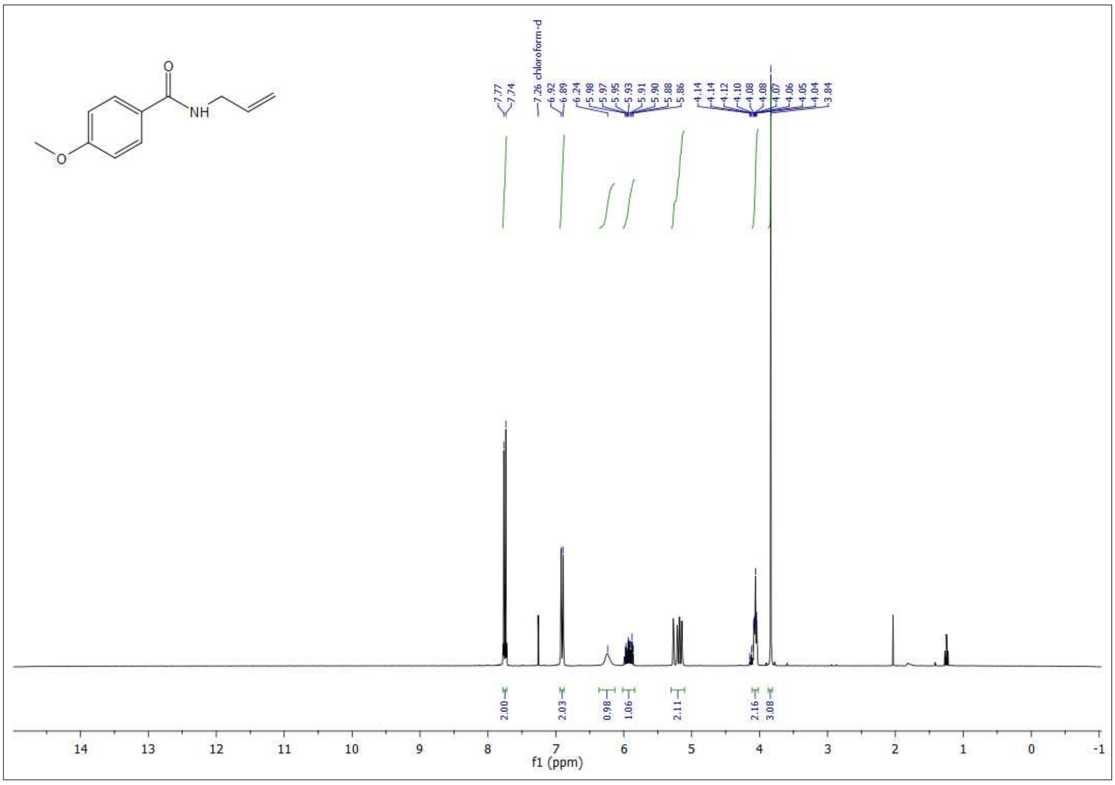

^{13}C {^{1}H} NMR (75 MHz, $CDCl_3$) of *N*-allyl-4-methoxybenzamide (5f)

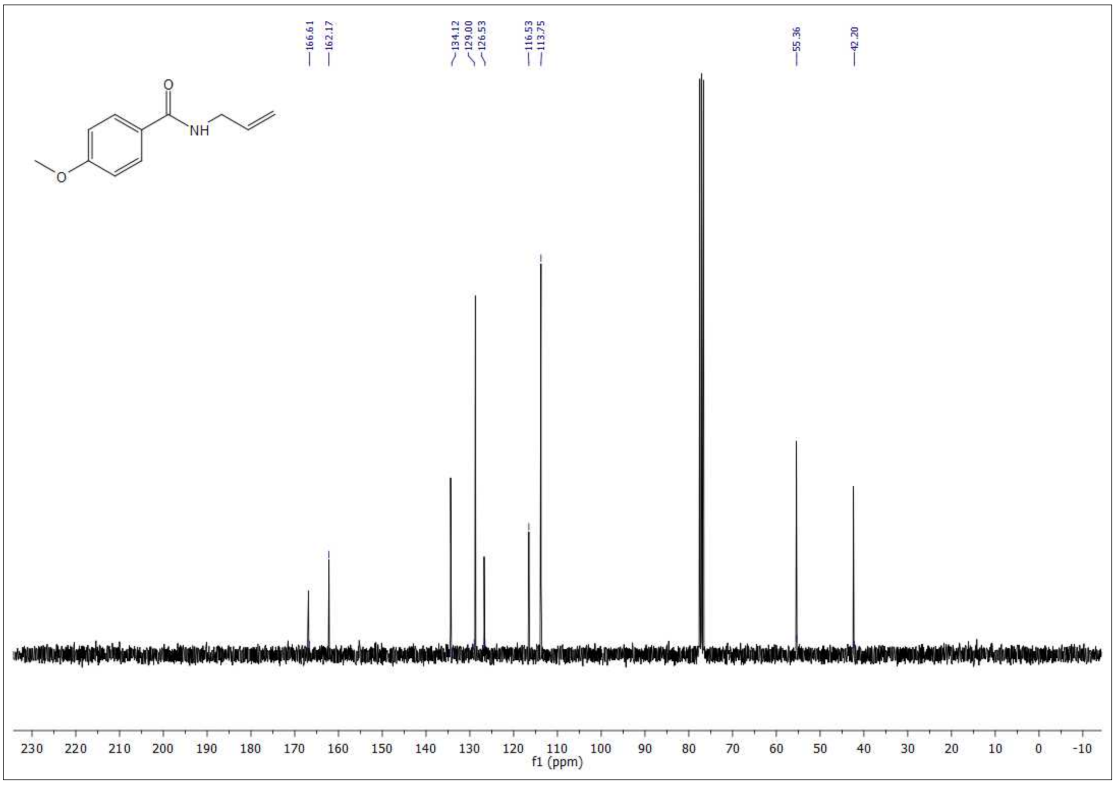

^{1}H NMR (300 MHz, $CDCl_3$) of *N*-allyl-4-isopropylbenzamide (5g)

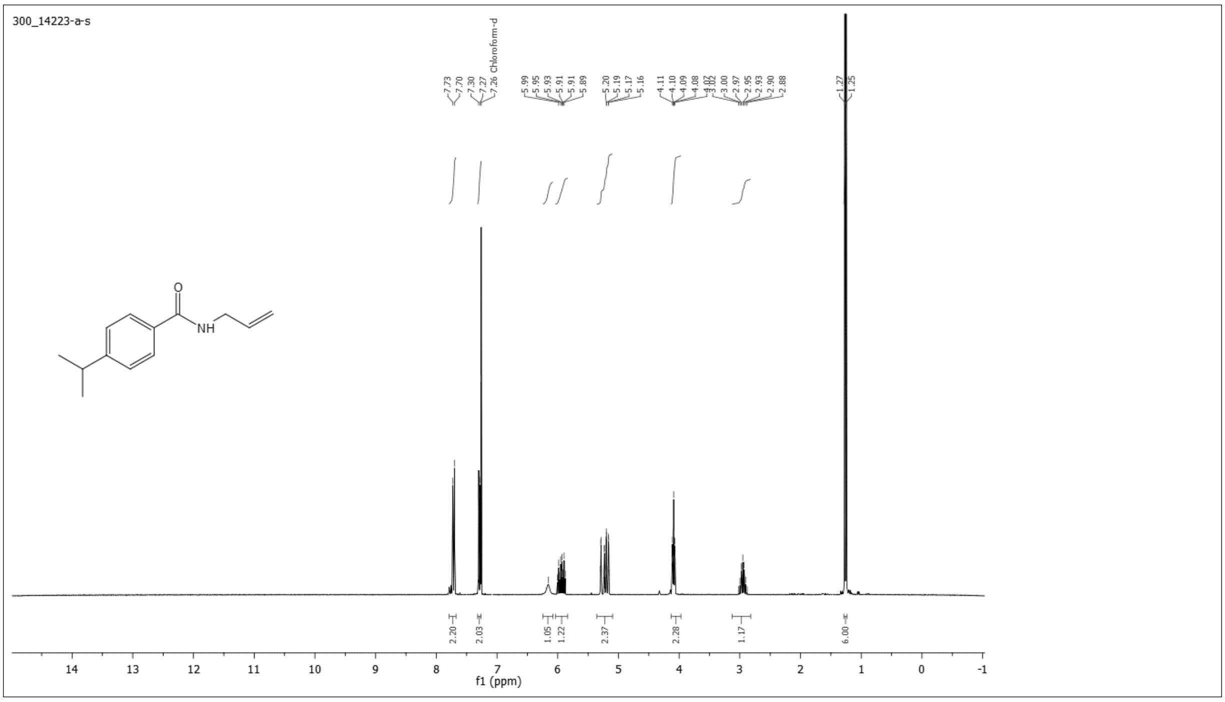

^{13}C {^{1}H} NMR (75 MHz, $CDCl_3$) of *N*-allyl-4-isopropylbenzamide (5g)

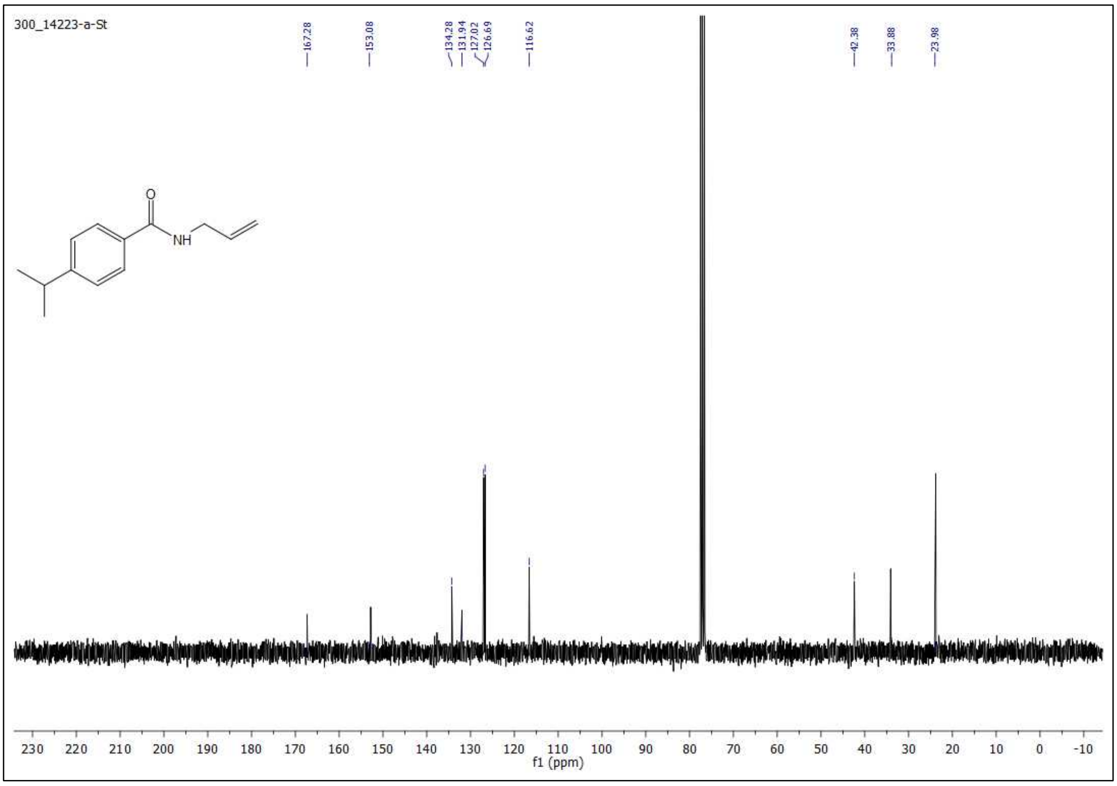

^{1}H NMR (300 MHz, $CDCl_3$) of methyl 4-(allylcarbamoyl)benzoate (5h)

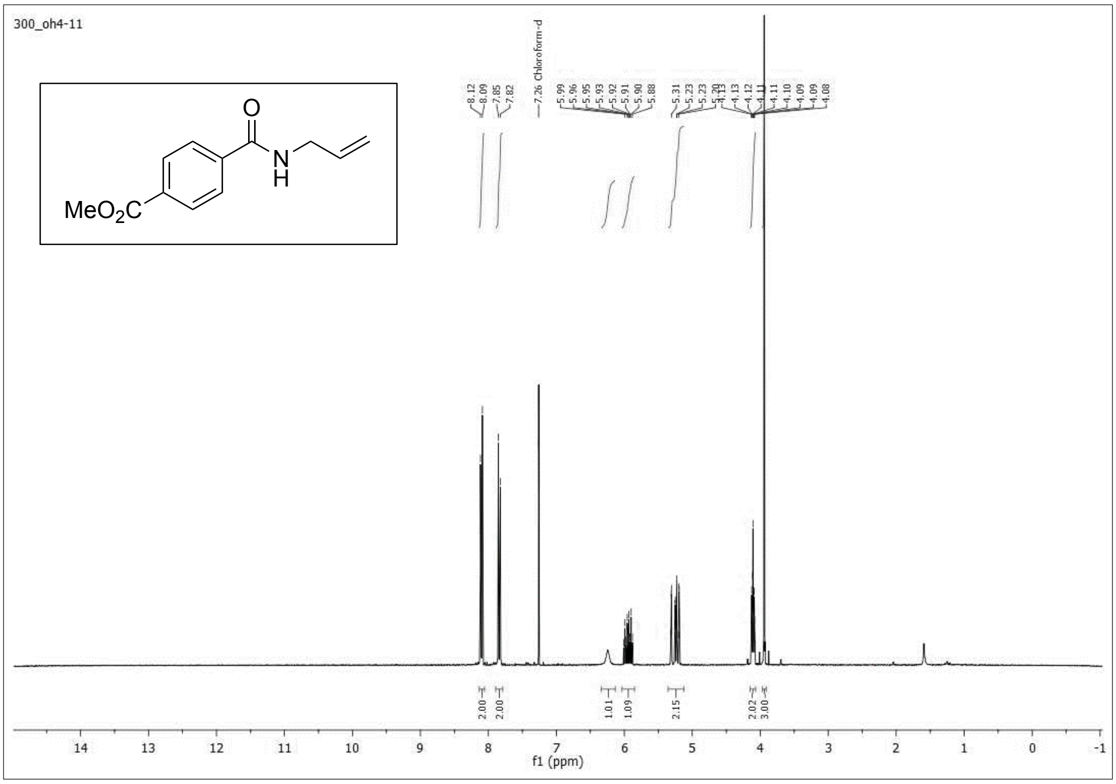

^{13}C {^{1}H} NMR (75 MHz, $CDCl_3$) of methyl 4-(allylcarbamoyl)benzoate (5h)

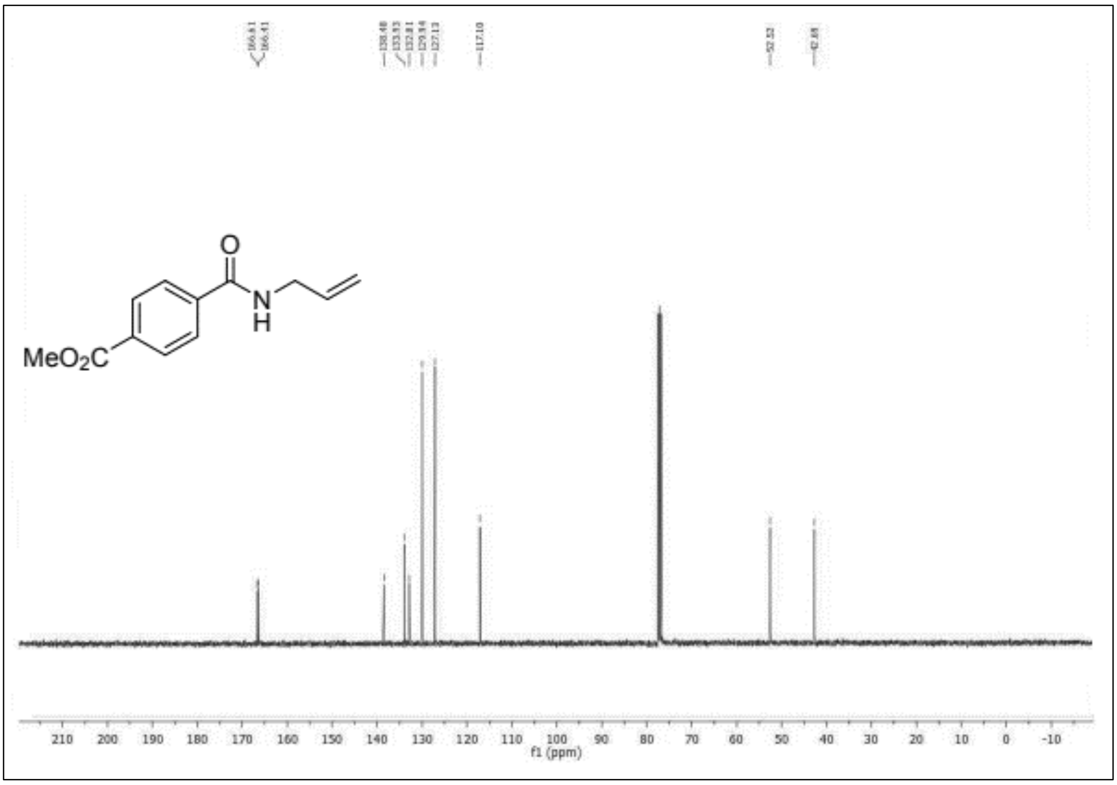

^{1}H NMR (300 MHz, $CDCl_3$) of *N*-allyl-3-methylbenzamide (5i)

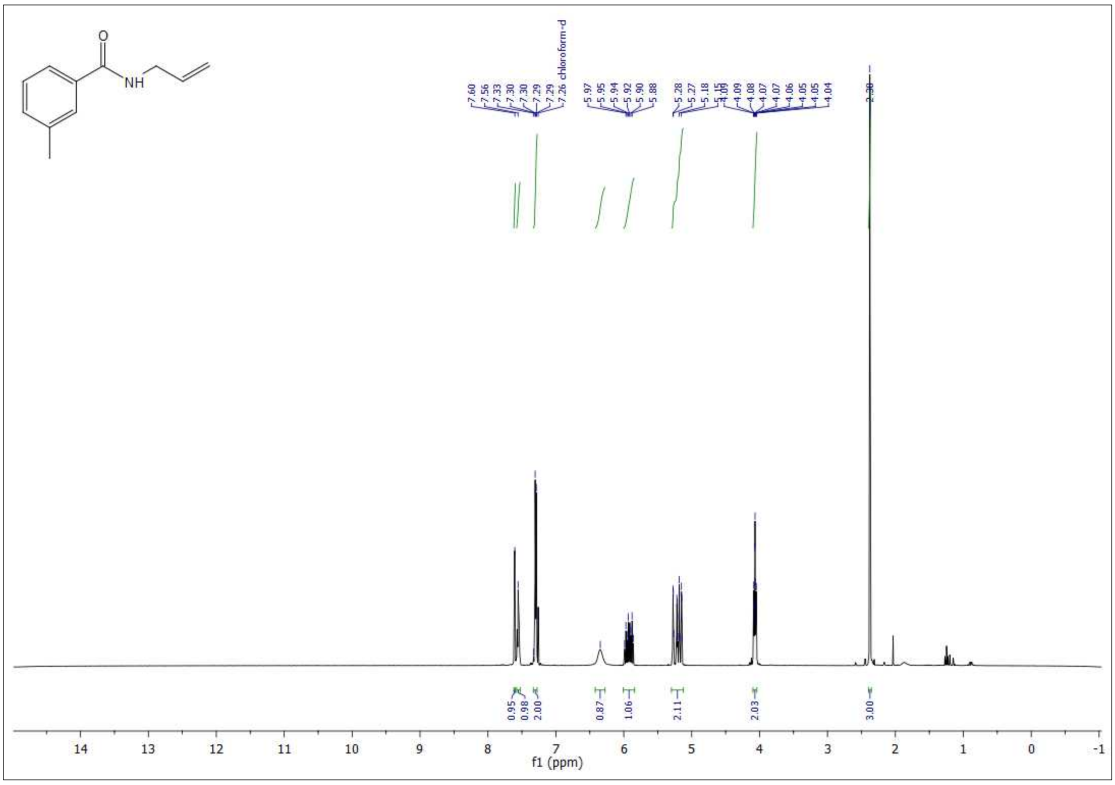

^{13}C {^{1}H} NMR (75 MHz, $CDCl_3$) of *N*-allyl-3-methylbenzamide (5i)

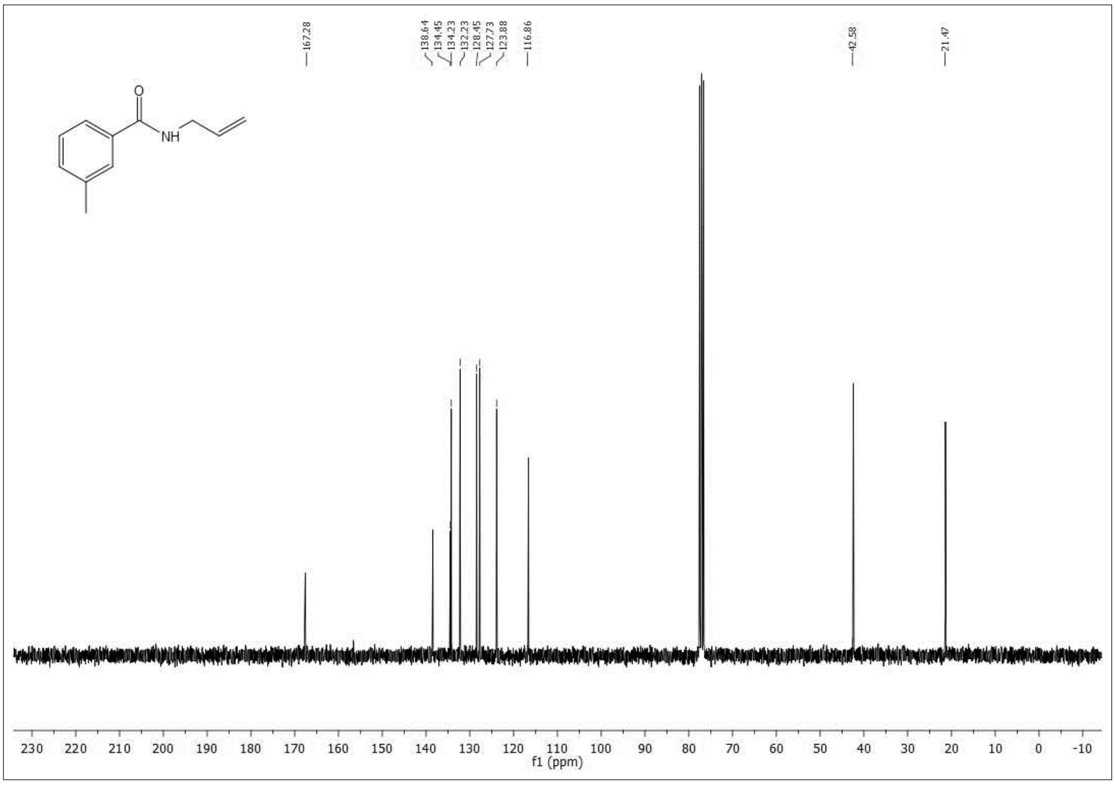

^{1}H NMR (300 MHz, $CDCl_3$) of *N*-allyl-3-cyanobenzamide (5j)

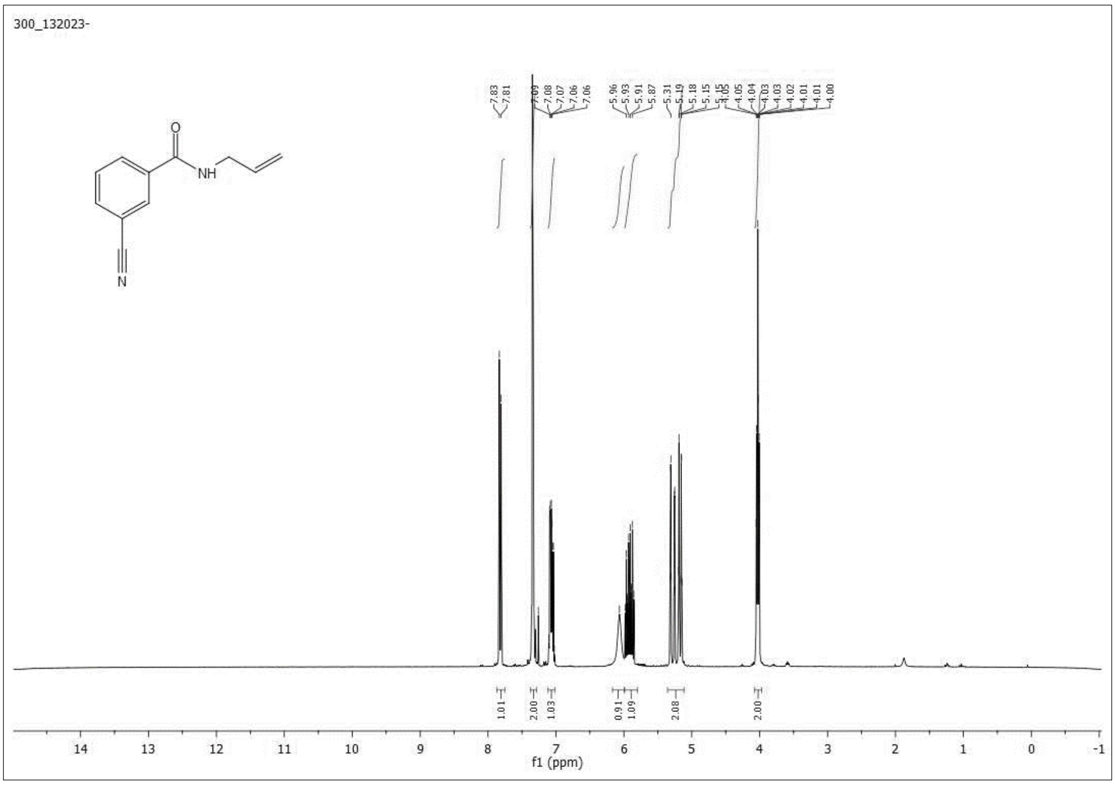

^{13}C {^{1}H} NMR (75 MHz, $CDCl_3$) of N-allyl-3-cyanobenzamide (5j)

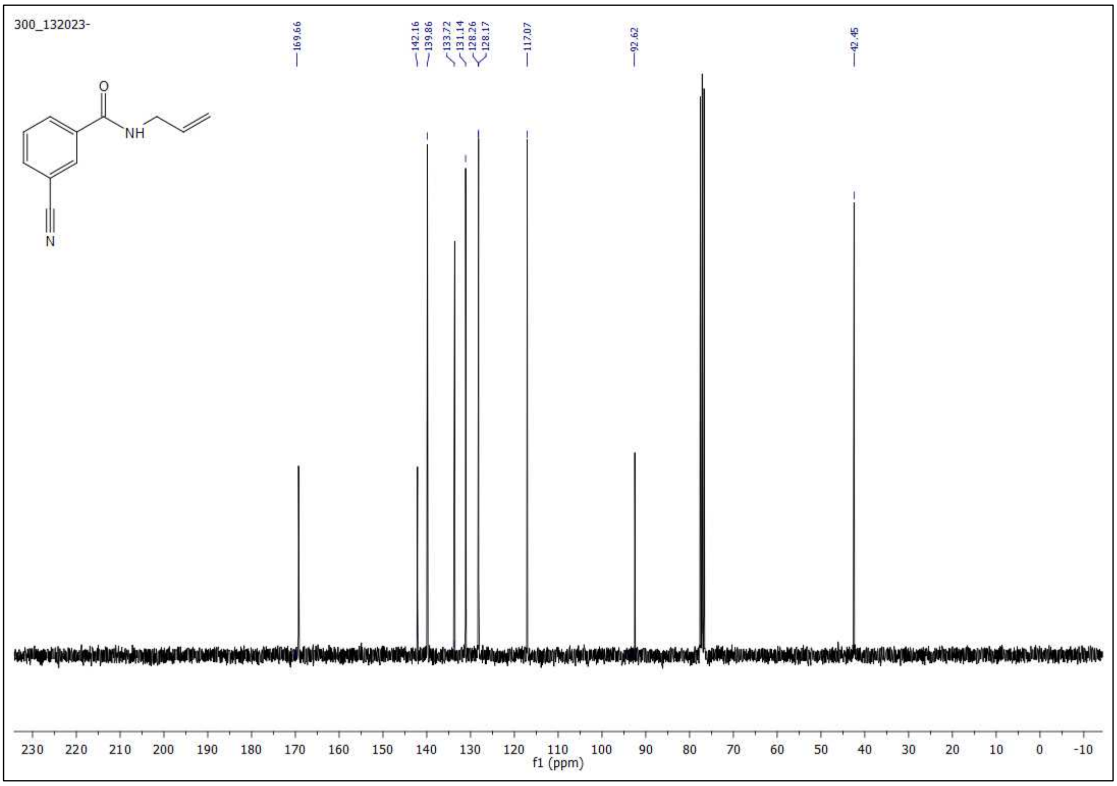

^{1}H NMR (300 MHz, $CDCl_3$) of *N*-allyl-3-nitrobenzamide (5k)

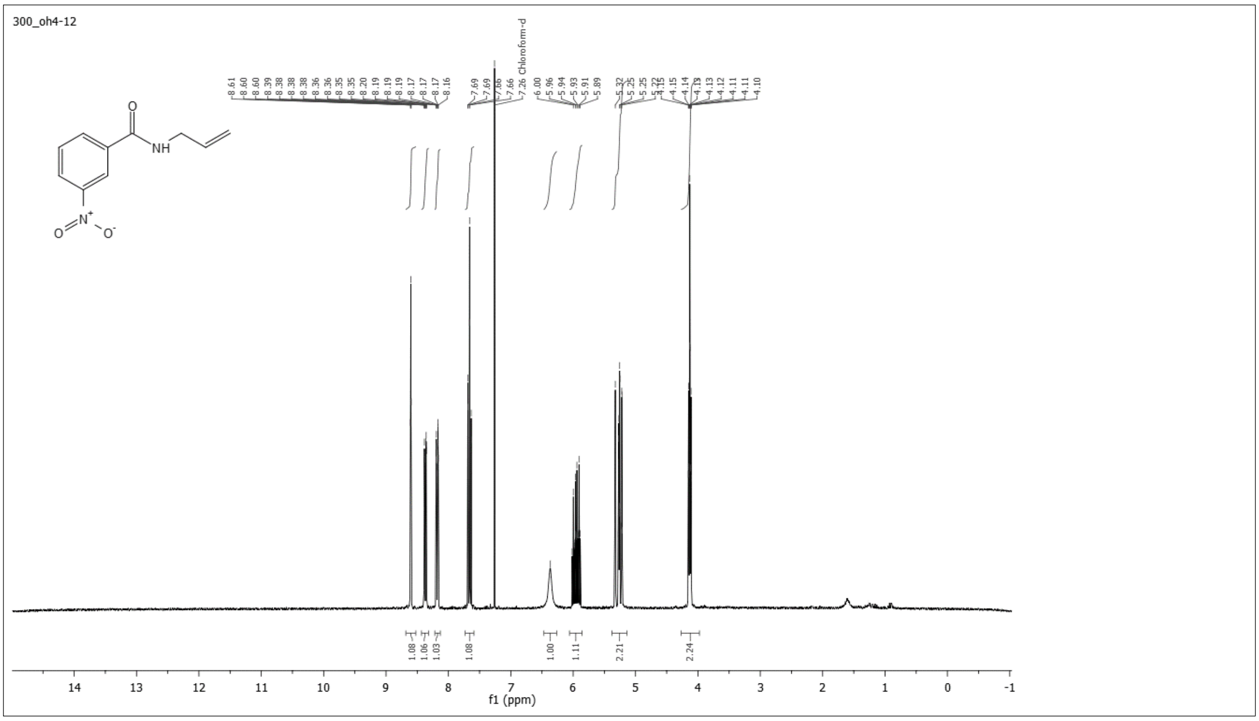

^{13}C {^{1}H} NMR (75 MHz, $CDCl_3$) of *N*-allyl-3-nitrobenzamide (5k)

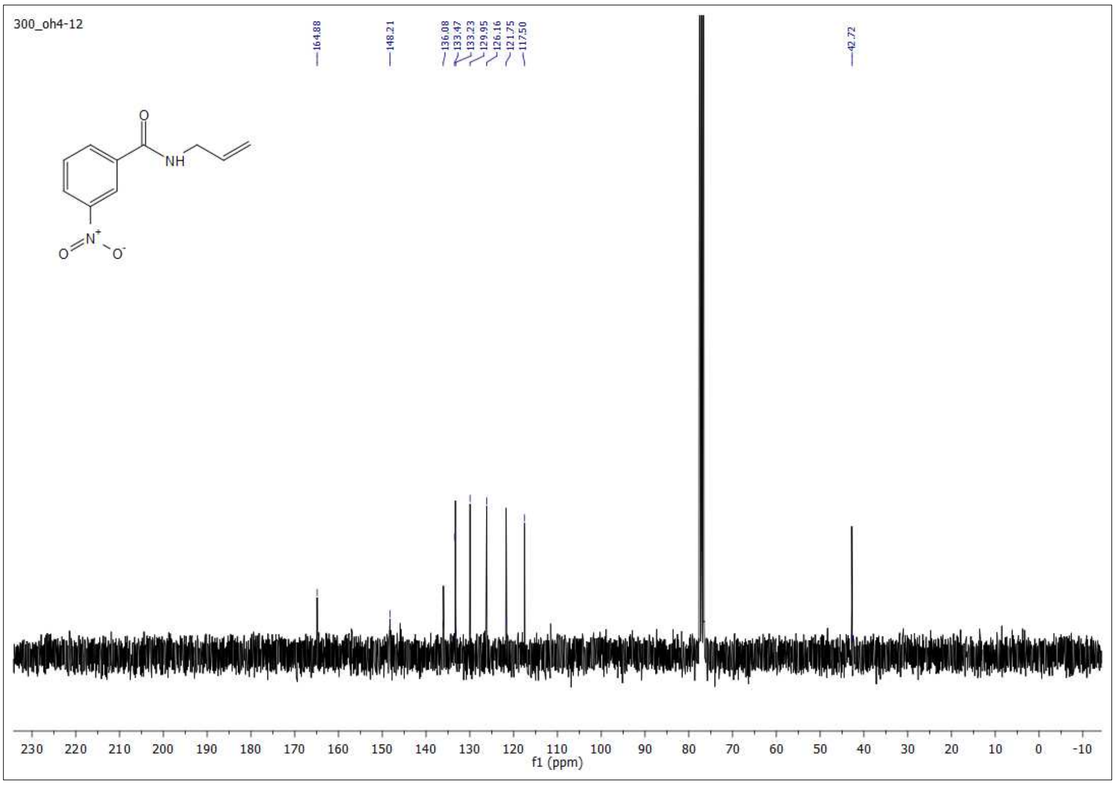

^{1}H NMR (300 MHz, $CDCl_3$) of *N*-allyl-3-(trifluoromethyl)benzamide (5l)

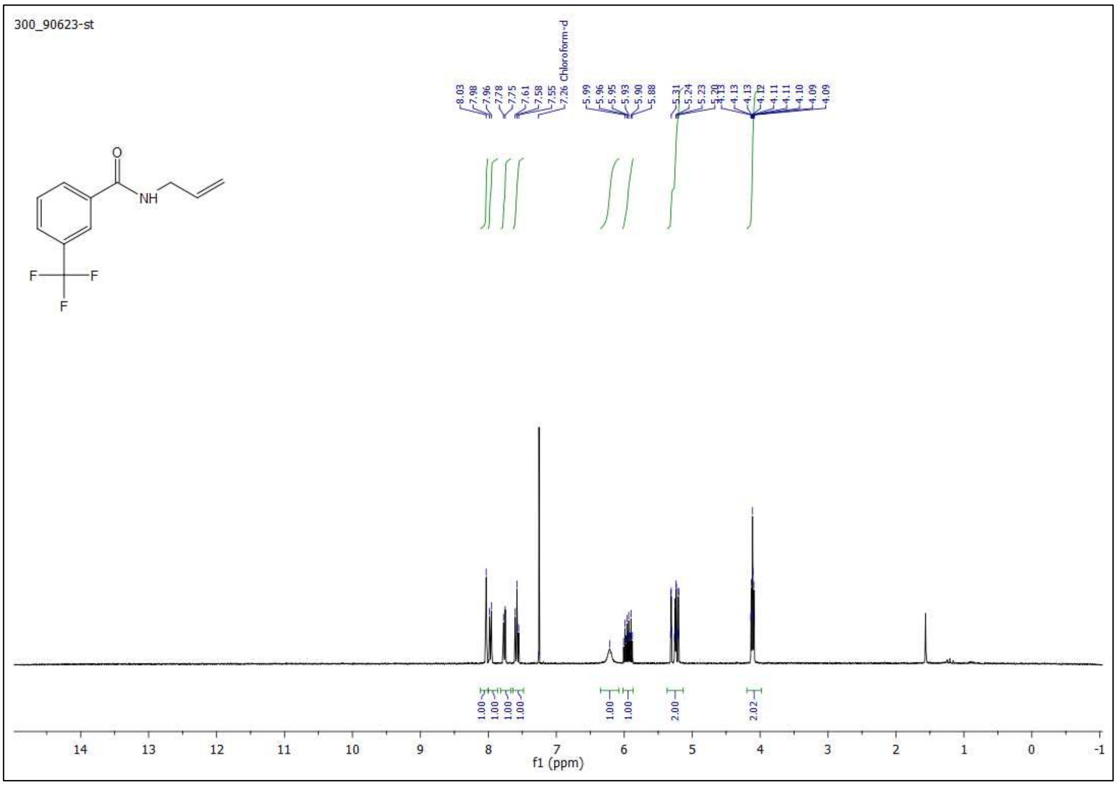

^{13}C {^{1}H} NMR (75 MHz, $CDCl_3$) of *N*-allyl-3-(trifluoromethyl)benzamide (5l)

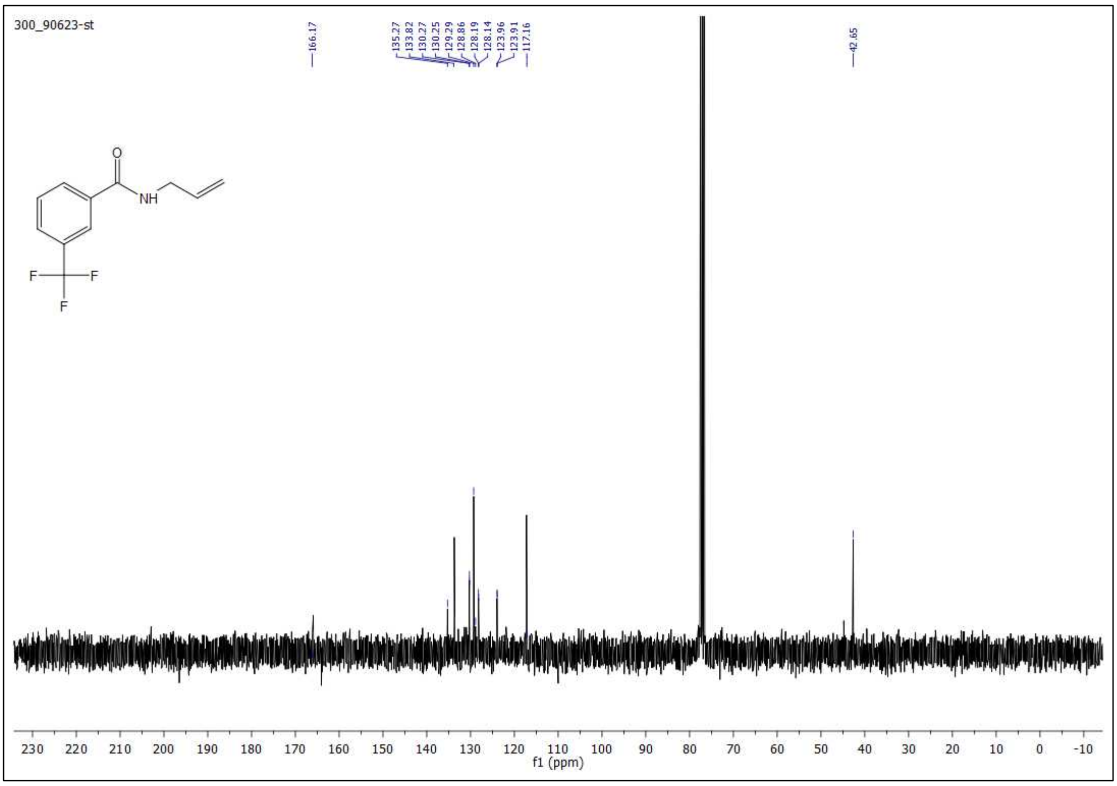

^{1}H NMR (300 MHz, $CDCl_3$) of *N*-allyl-2-methylbenzamide (5m)

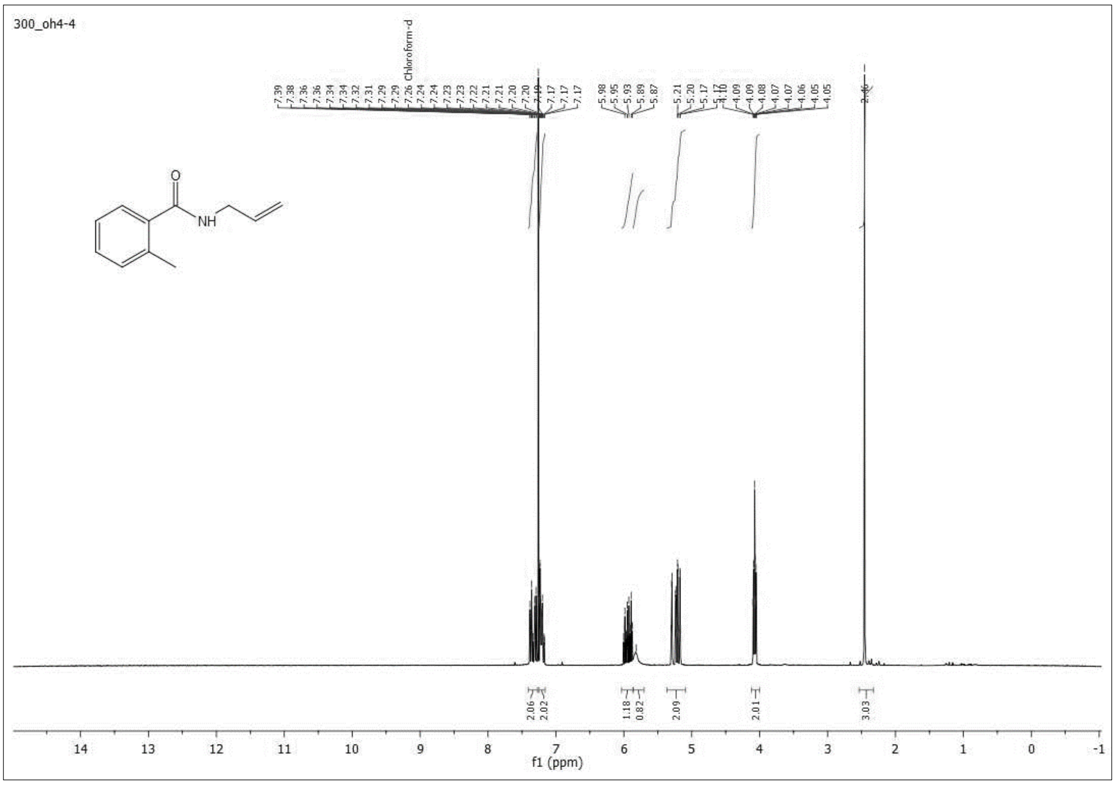

^{13}C {^{1}H} NMR (75 MHz, $CDCl_3$) of *N*-allyl-2-methylbenzamide (5m)

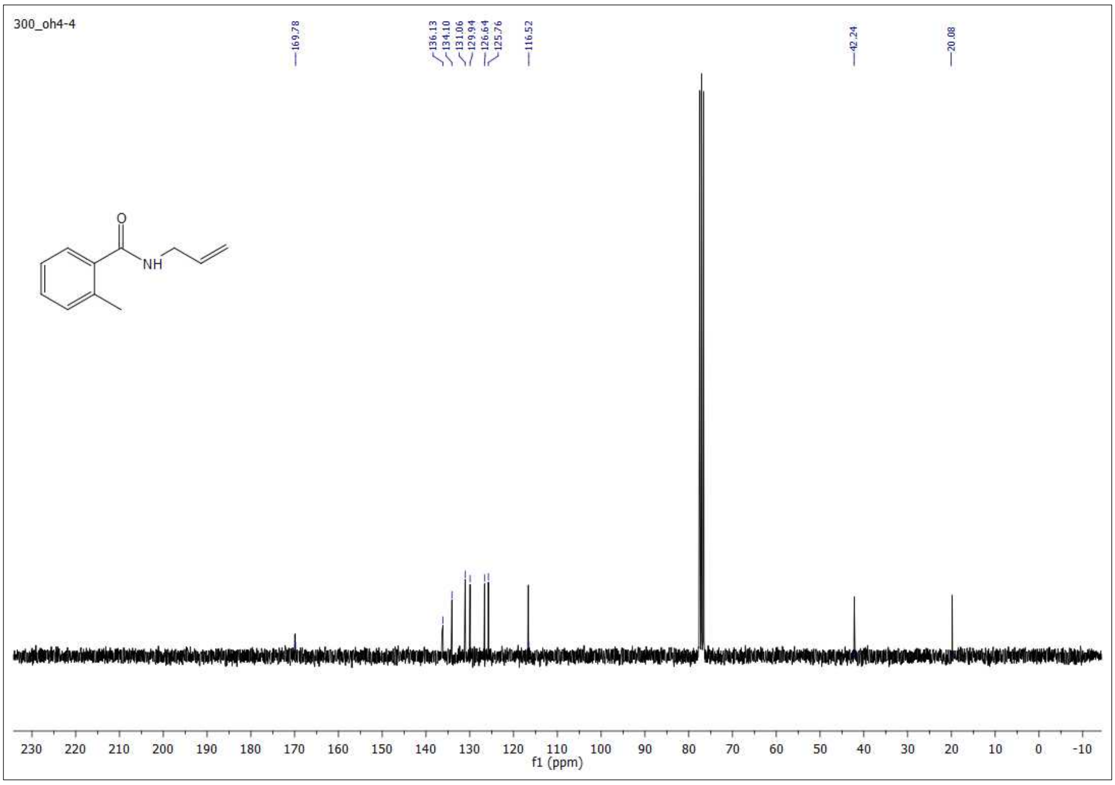

^{1}H NMR (300 MHz, $CDCl_3$) of *N*-allyl-2-bromobenzamide (5n)

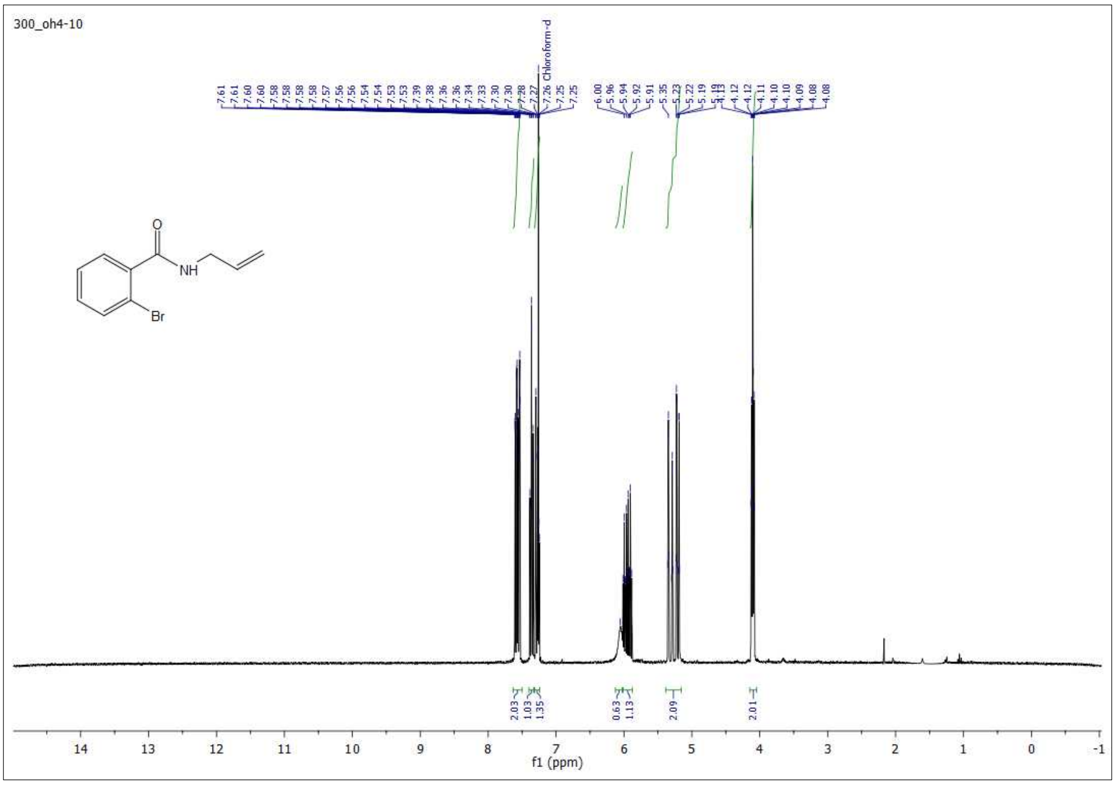

^{13}C {^{1}H} NMR (75 MHz, $CDCl_3$) of *N*-allyl-2-bromobenzamide (5n)

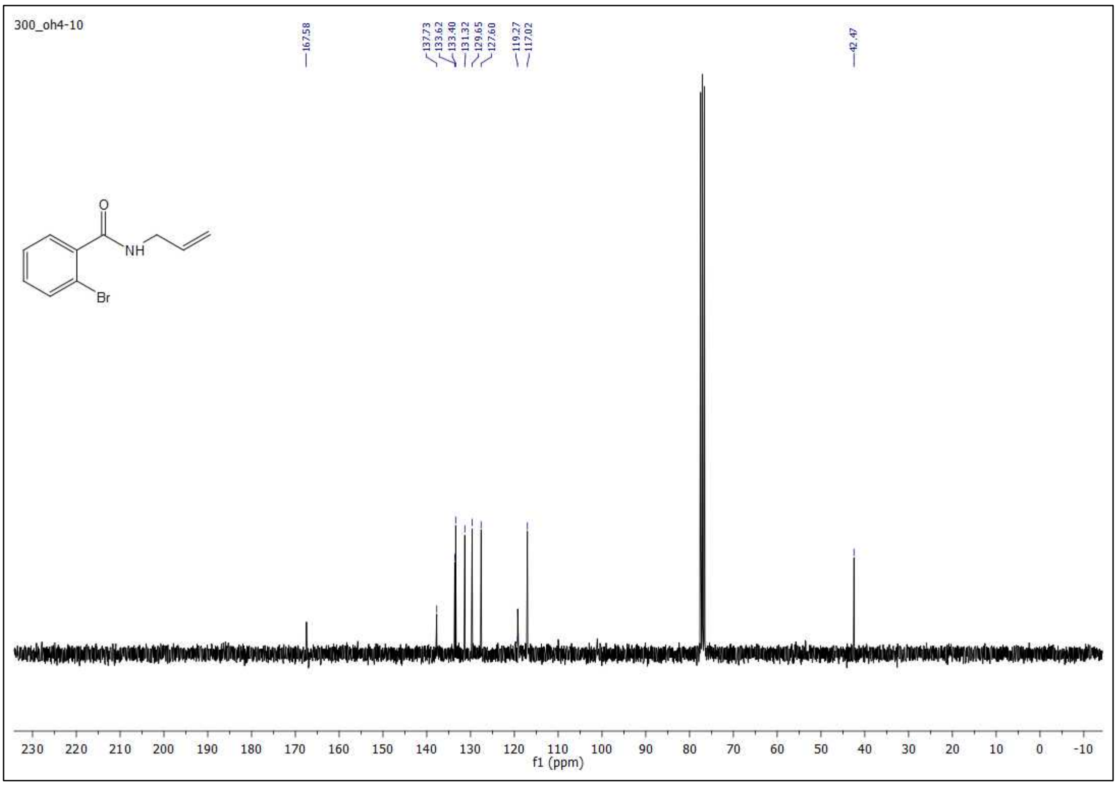

^{1}H NMR (300 MHz, $CDCl_3$) of *N*-allyl-2-chlorobenzamide(5o)

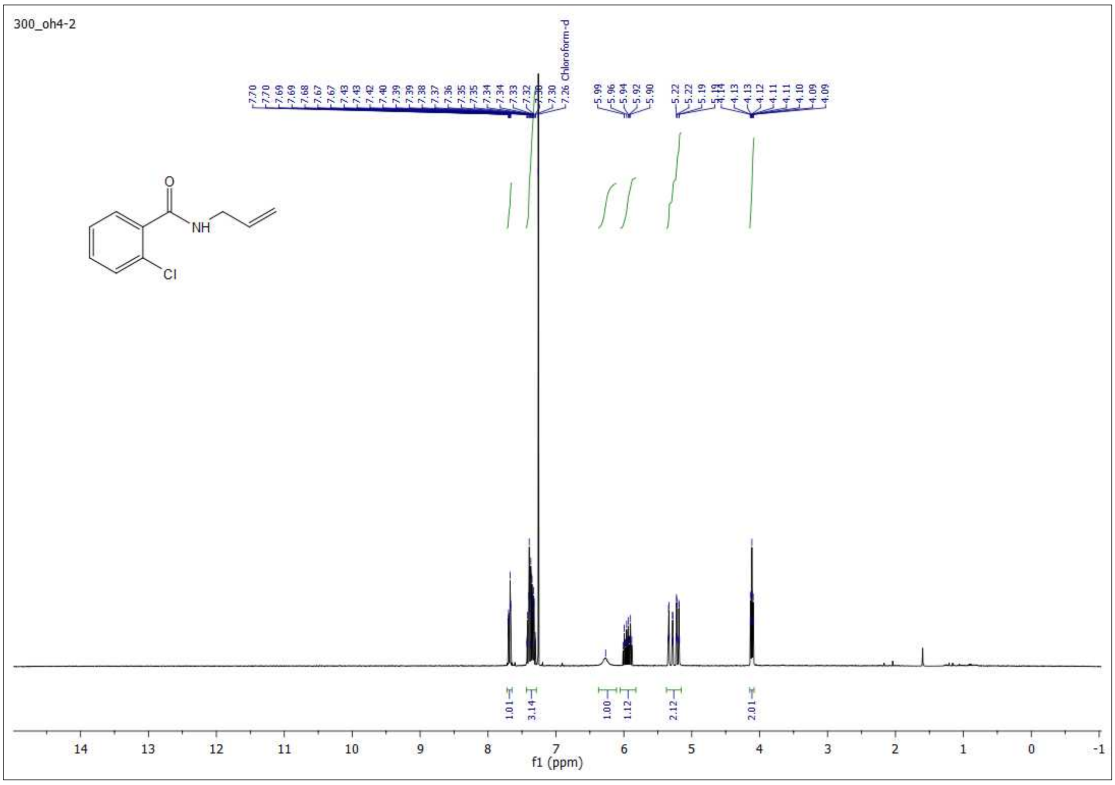

^{13}C {^{1}H} NMR (75 MHz, $CDCl_3$) of N-allyl-2-chlorobenzamide (5o)

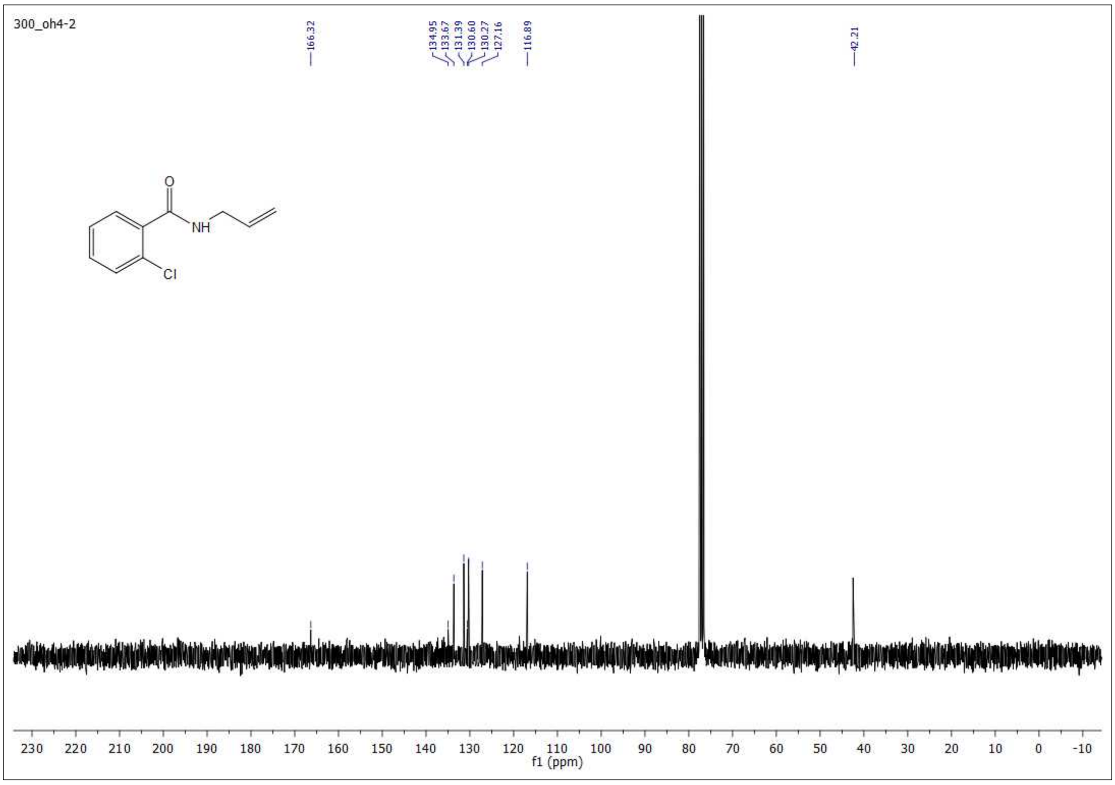

^{1}H NMR (300 MHz, $CDCl_3$) of *N*-allyl-2-iodobenzamide (5p)

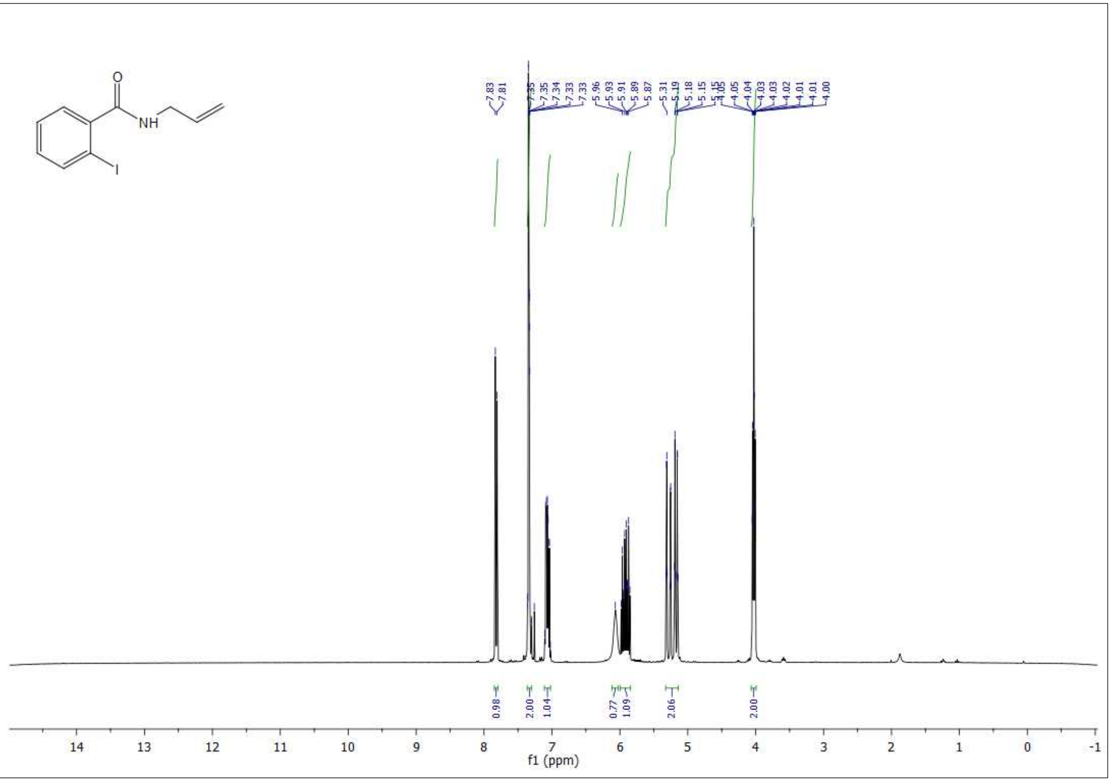

^{13}C {^{1}H} NMR (75 MHz, $CDCl_3$) of *N*-allyl-2-iodobenzamide (5p)

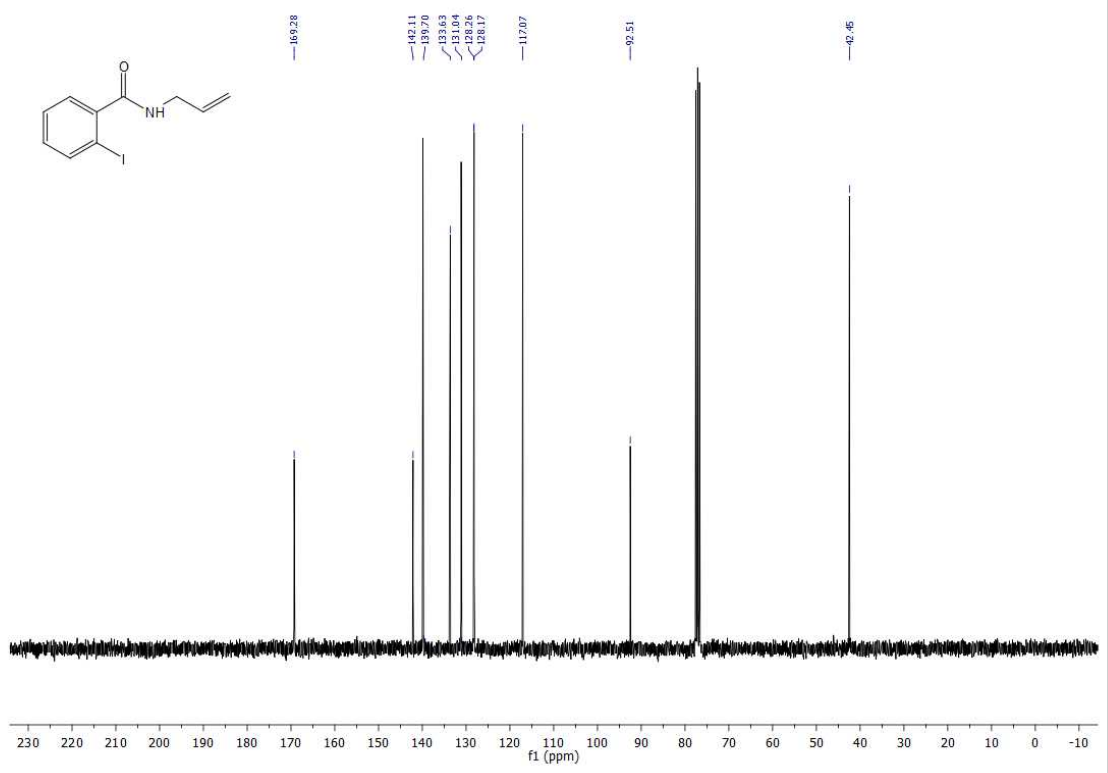

^{1}H NMR (300 MHz, $CDCl_3$) of *N*-allyl-[1,1'-biphenyl]-2-carboxamide (5q)

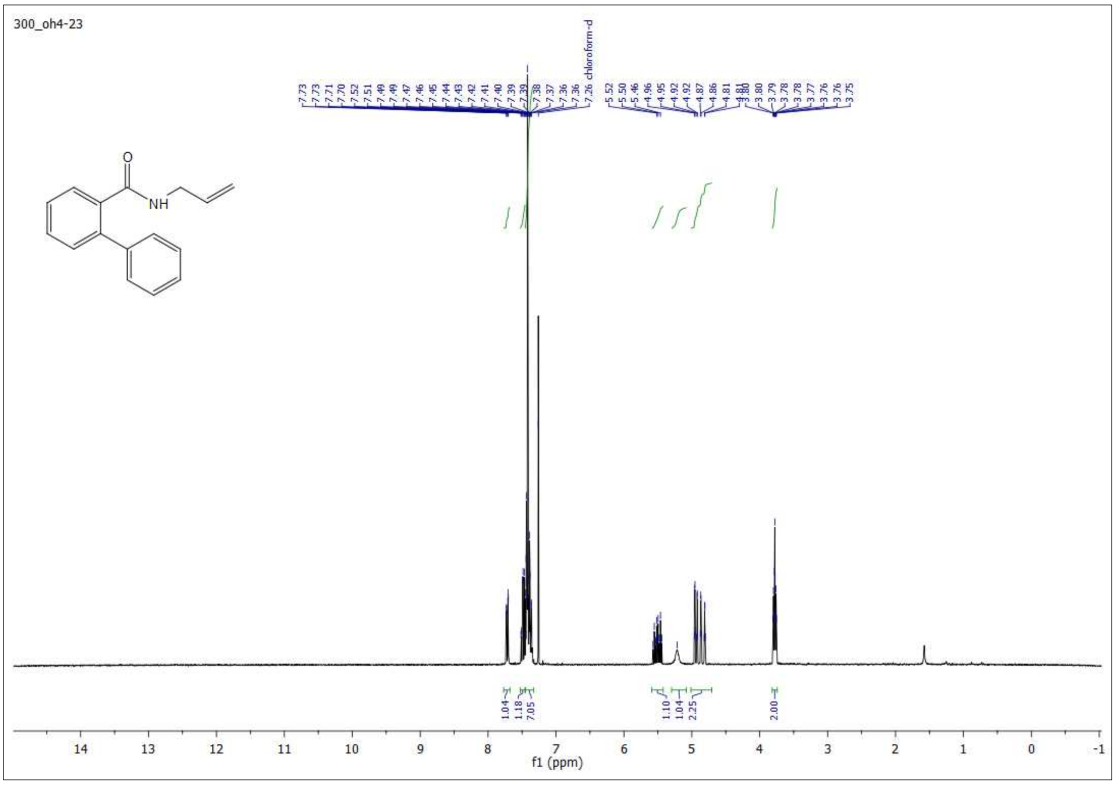

^{13}C {^{1}H} NMR (75 MHz, $CDCl_3$) of *N*-allyl-[1,1'-biphenyl]-2-carboxamide(5q)

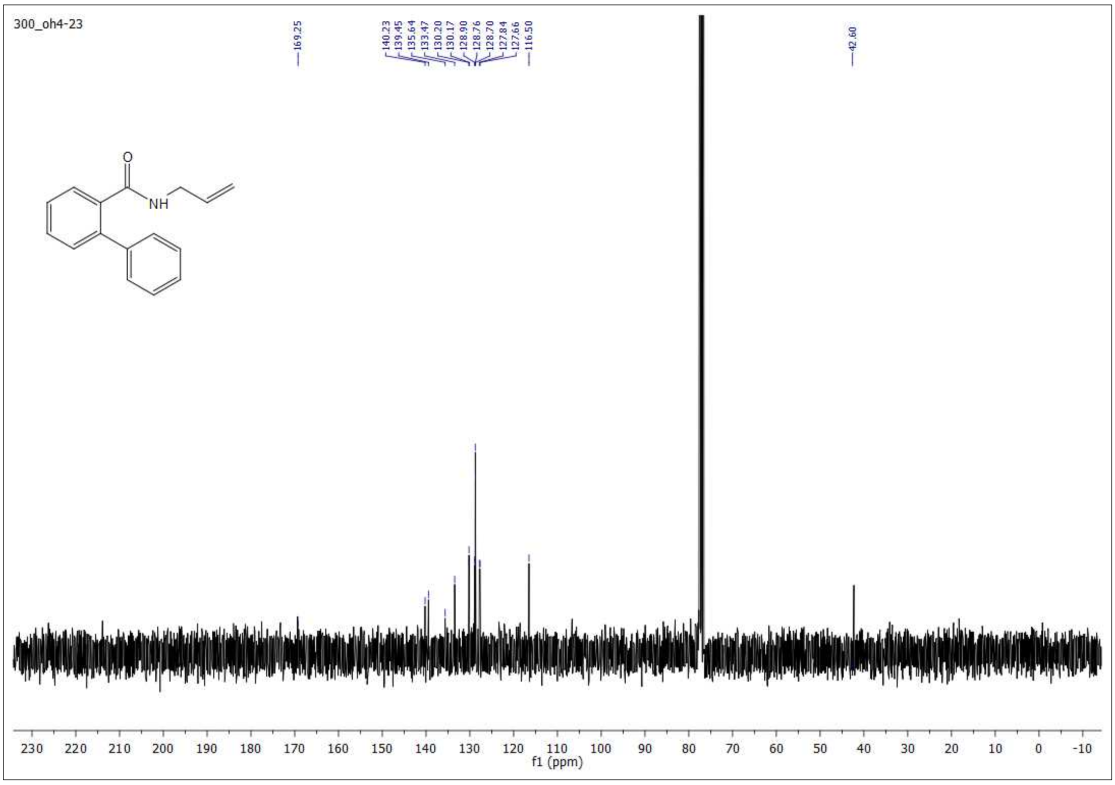

^{1}H NMR (300 MHz, $CDCl_3$) of *N*-allylfuran-2-carboxamide (5r)

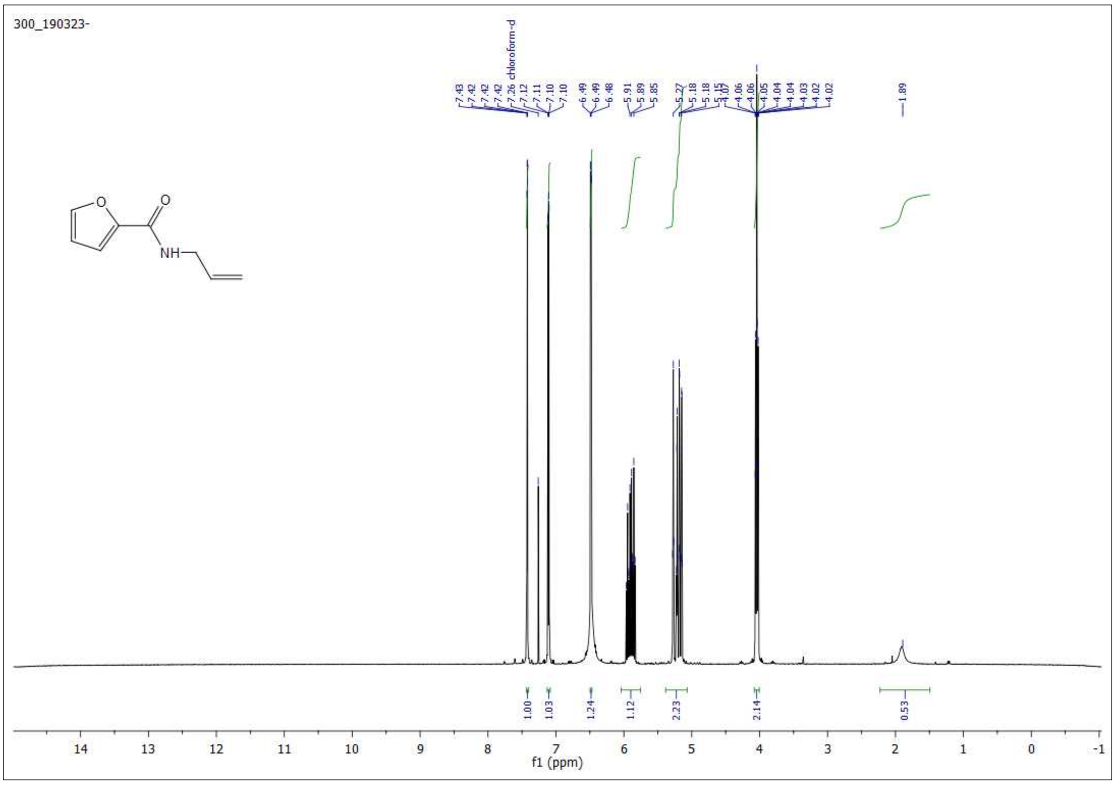

^{13}C {^{1}H} NMR (75 MHz, $CDCl_3$) of *N*-allylfuran-2-carboxamide(5r)

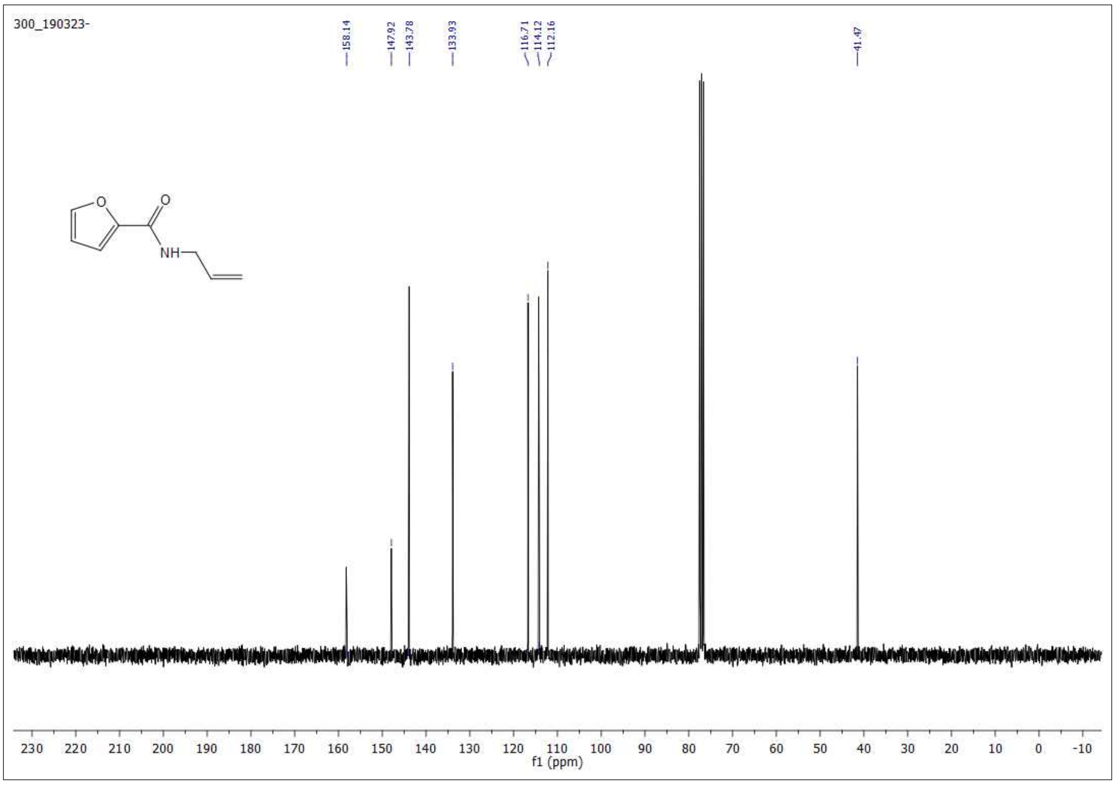

^{1}H NMR (300 MHz, $CDCl_3$) of ***N*-allylthiophene-2-carboxamide (5s)**

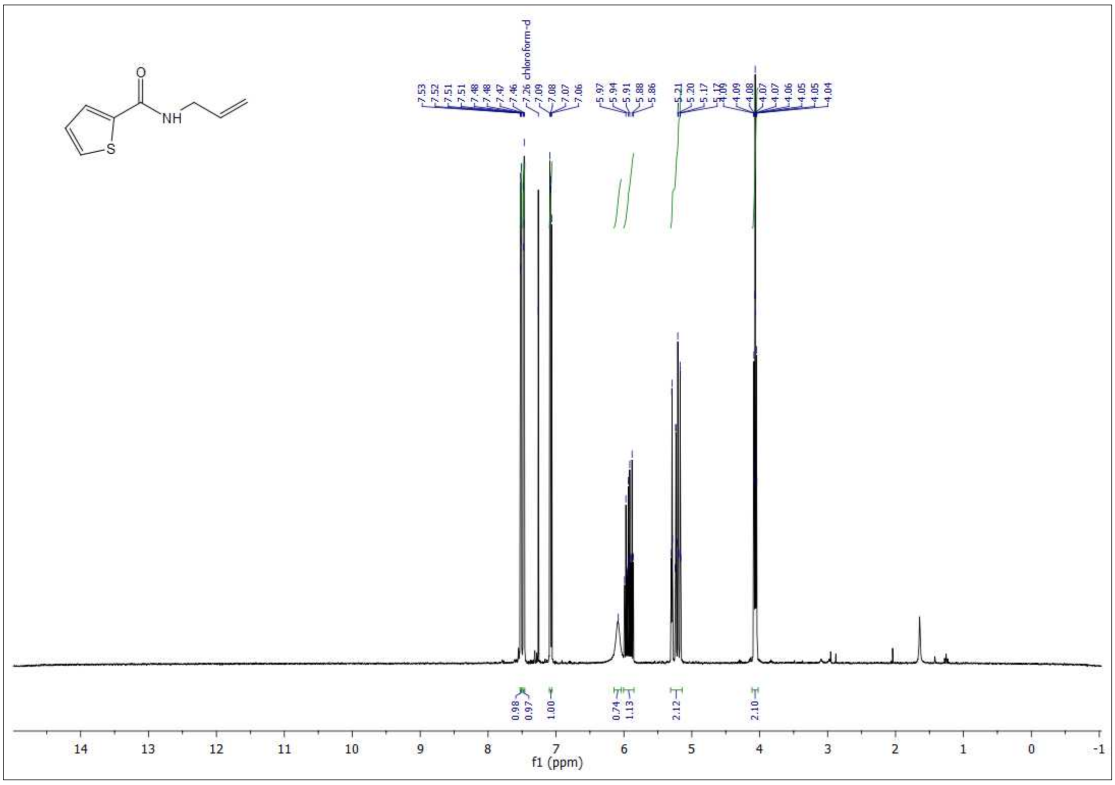

^{13}C {^{1}H} NMR (75 MHz, $CDCl_3$) of ***N*-allylthiophene-2-carboxamide (5s)**

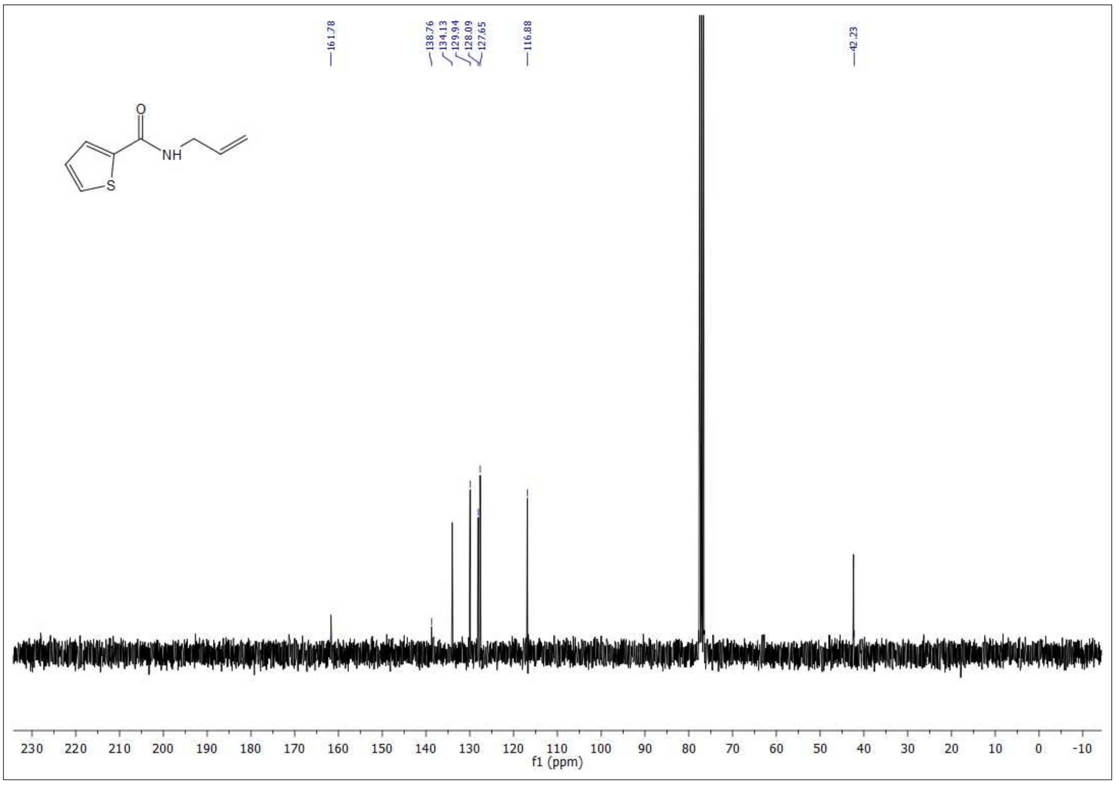

^{1}H NMR (300 MHz, $CDCl_3$) of *N*-allylnicotinamide (5t)

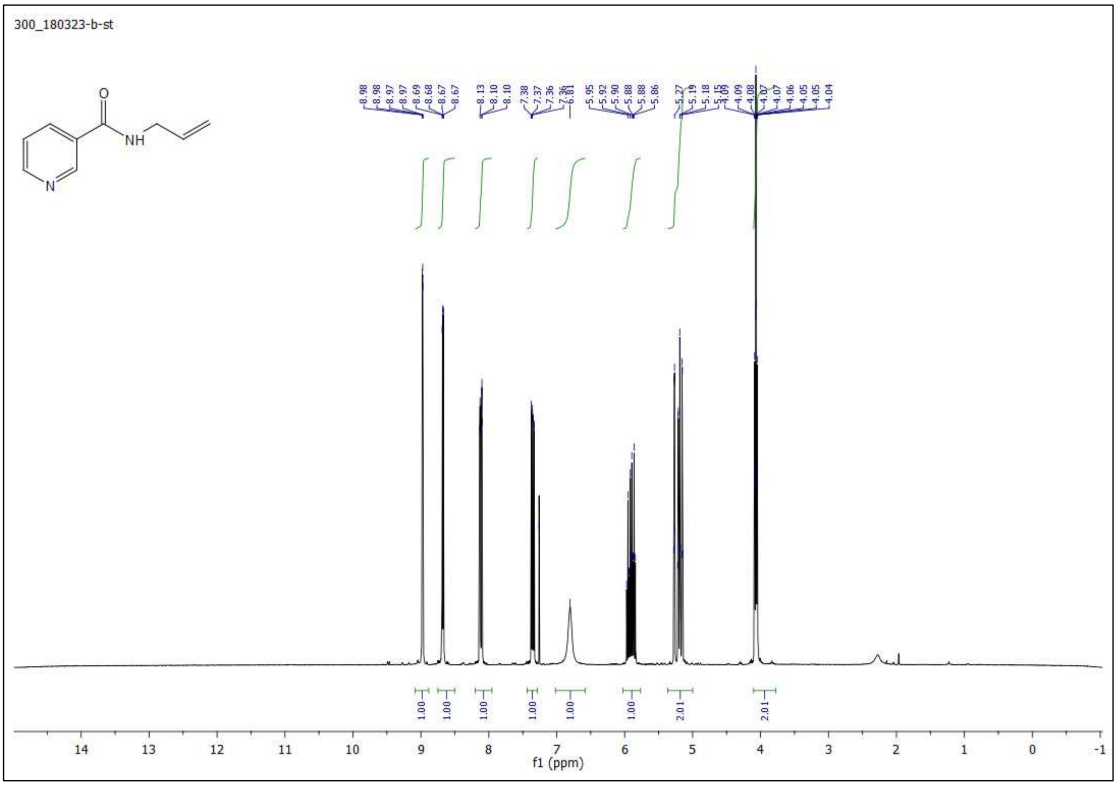

^{13}C {^{1}H} NMR (75 MHz, $CDCl_3$) of *N*-allylnicotinamide (5t)

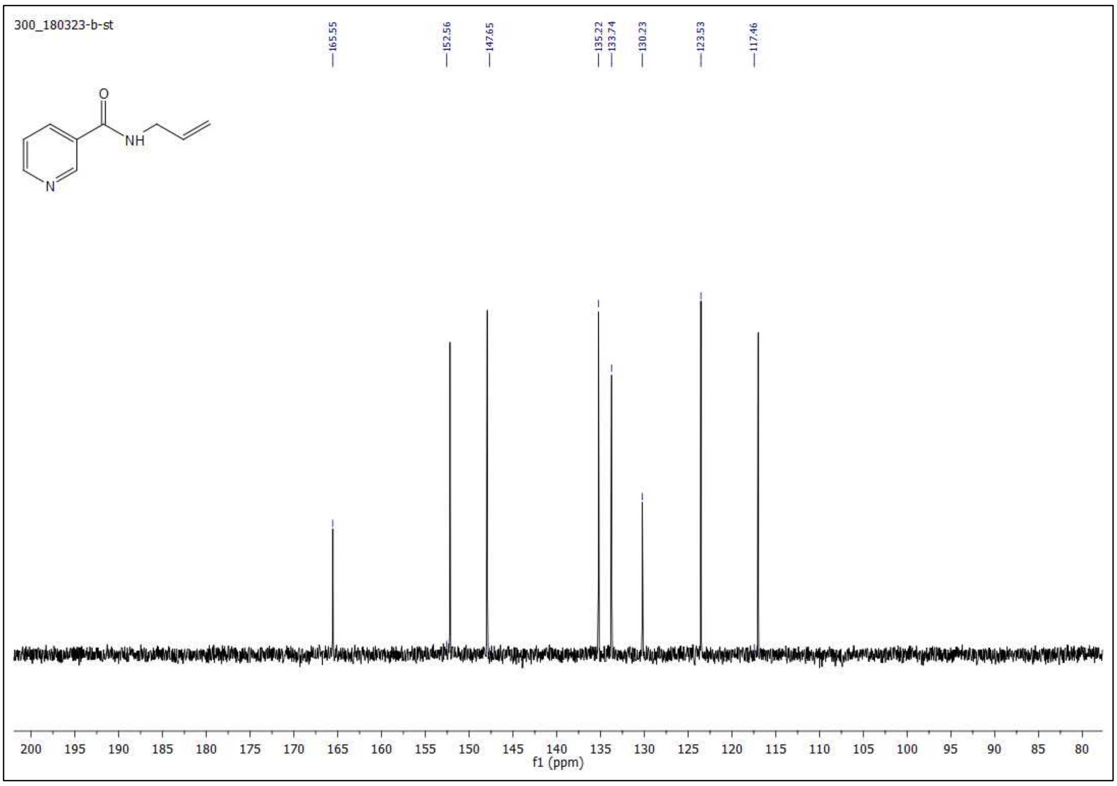

^{1}H NMR (300 MHz, $CDCl_3$) of *N*-(but-3-en-1-yl)benzamide (5u)

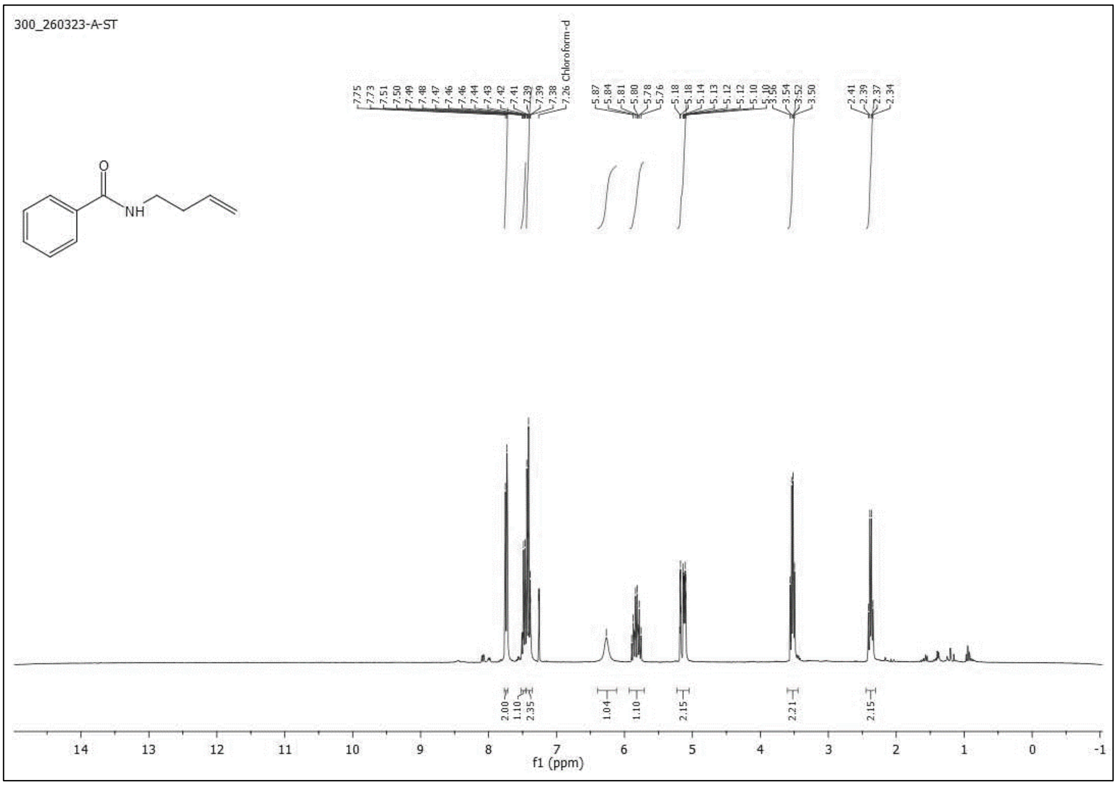

^{13}C {^{1}H} NMR (75 MHz, $CDCl_3$) of *N*-(but-3-en-1-yl)benzamide (5u)

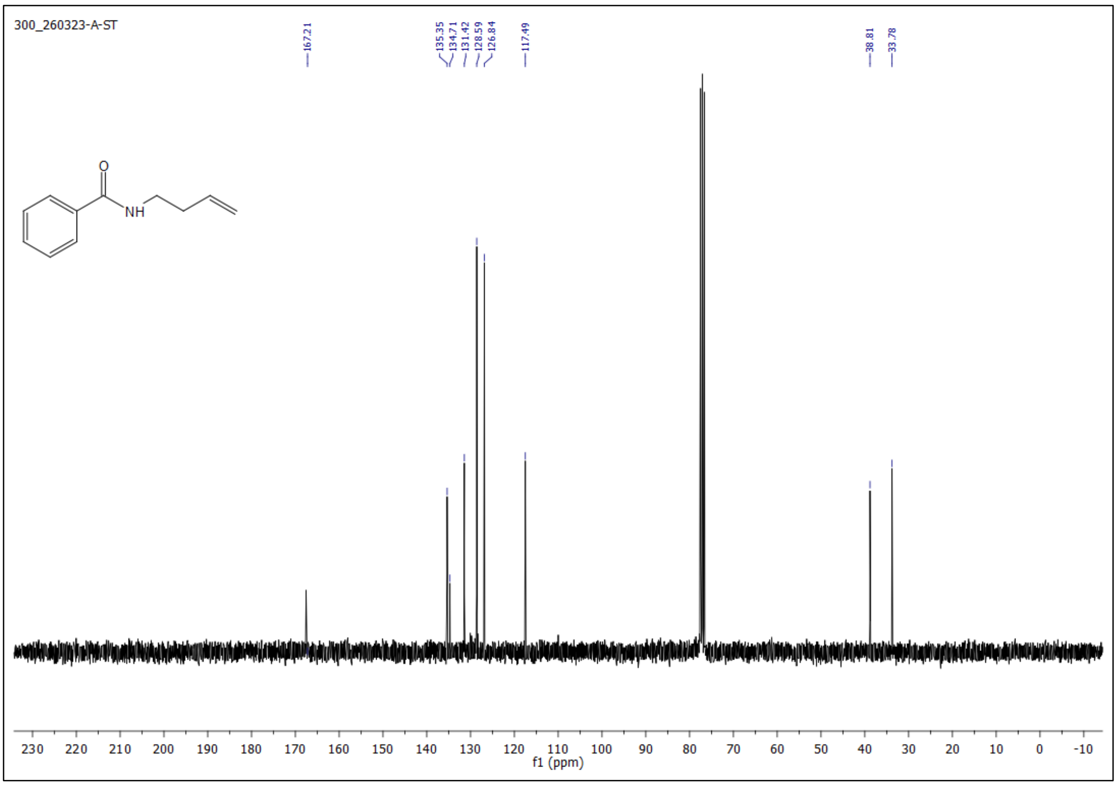

^{1}H NMR (300 MHz, $CDCl_3$) of *N*-(but-3-en-1-yl)-4-chlorobenzamide (5v)

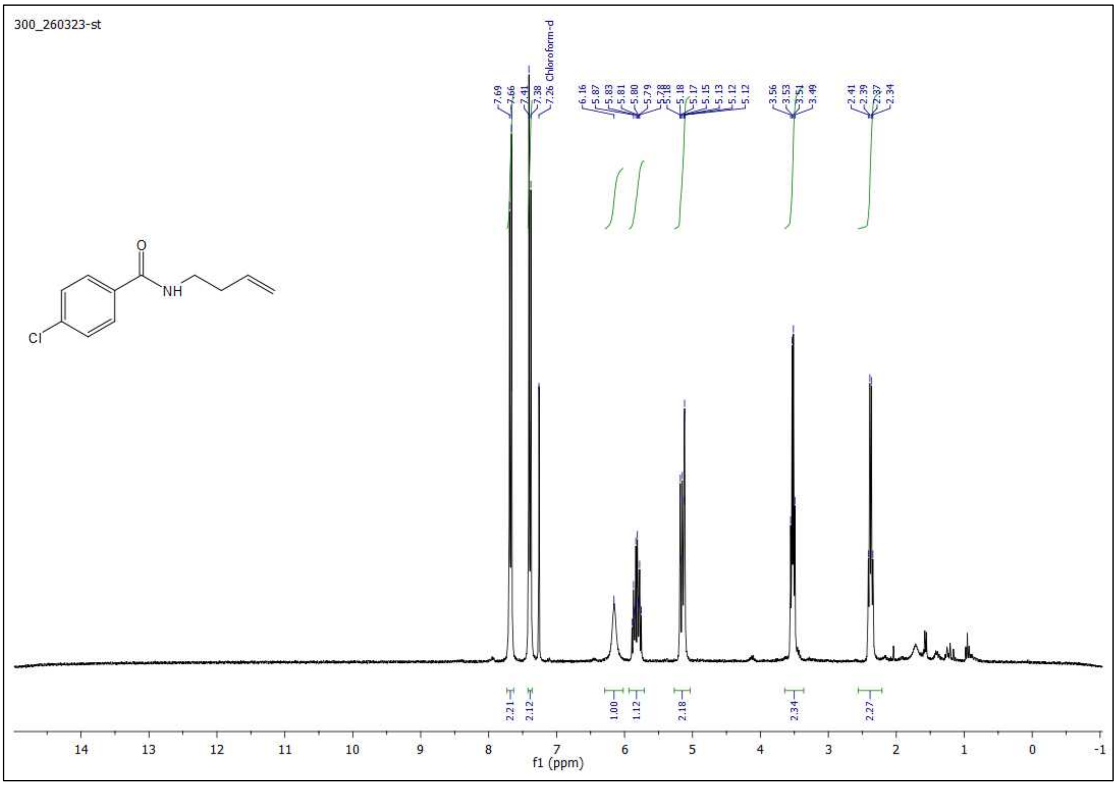

^{13}C {^{1}H} NMR (75 MHz, $CDCl_3$) of *N*-(but-3-en-1-yl)-4-chlorobenzamide (5v)

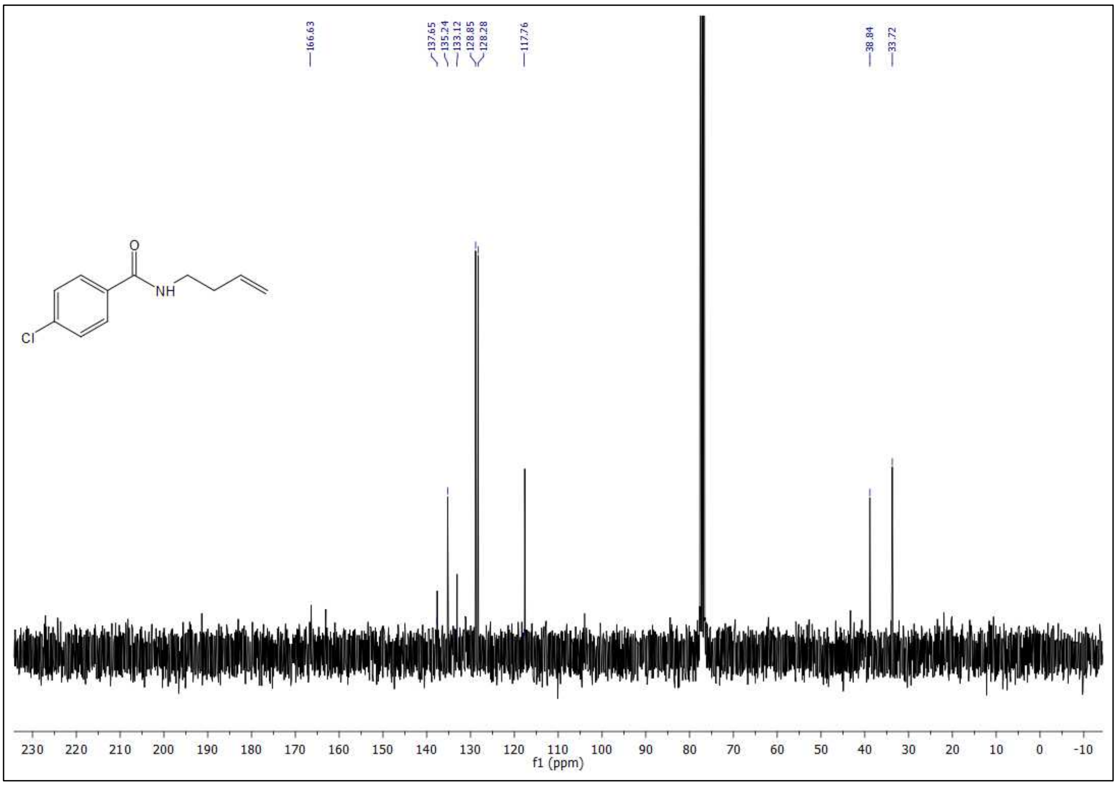

^{1}H NMR (300 MHz, $CDCl_3$) of *N*-allylcyclohexanecarboxamide (5w)

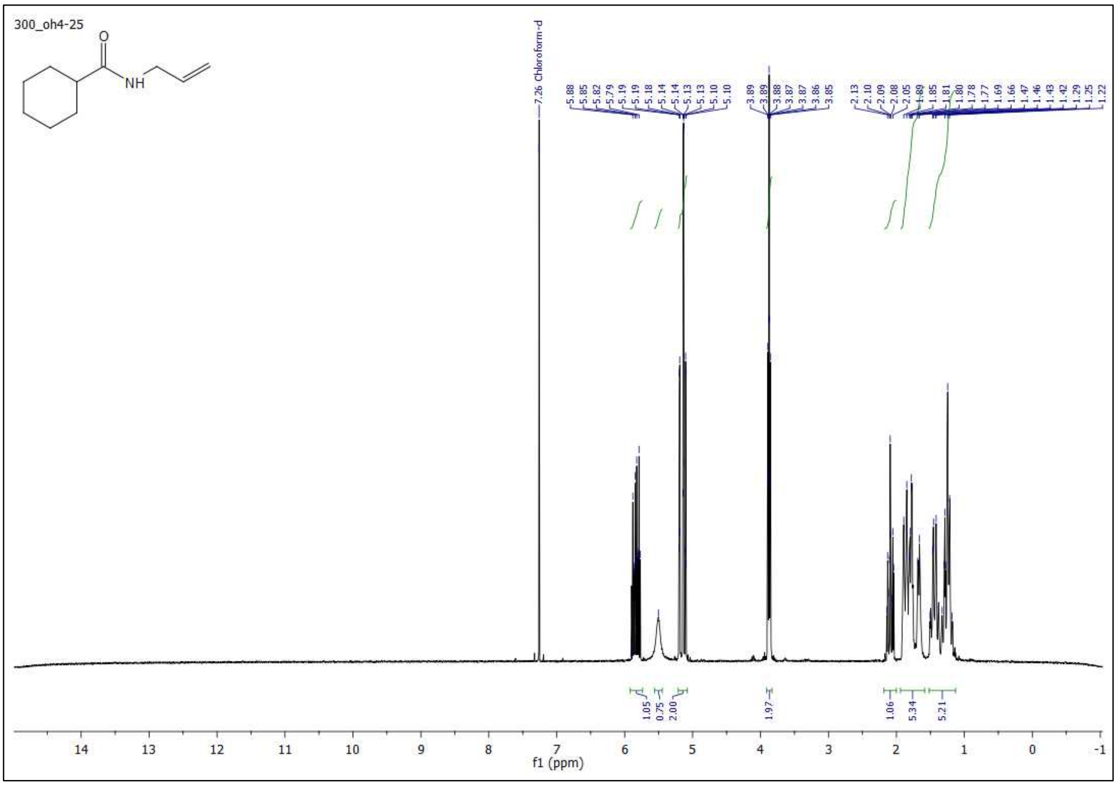

^{13}C {^{1}H} NMR (75 MHz, $CDCl_3$) of *N*-allylcyclohexanecarboxamide (5w)

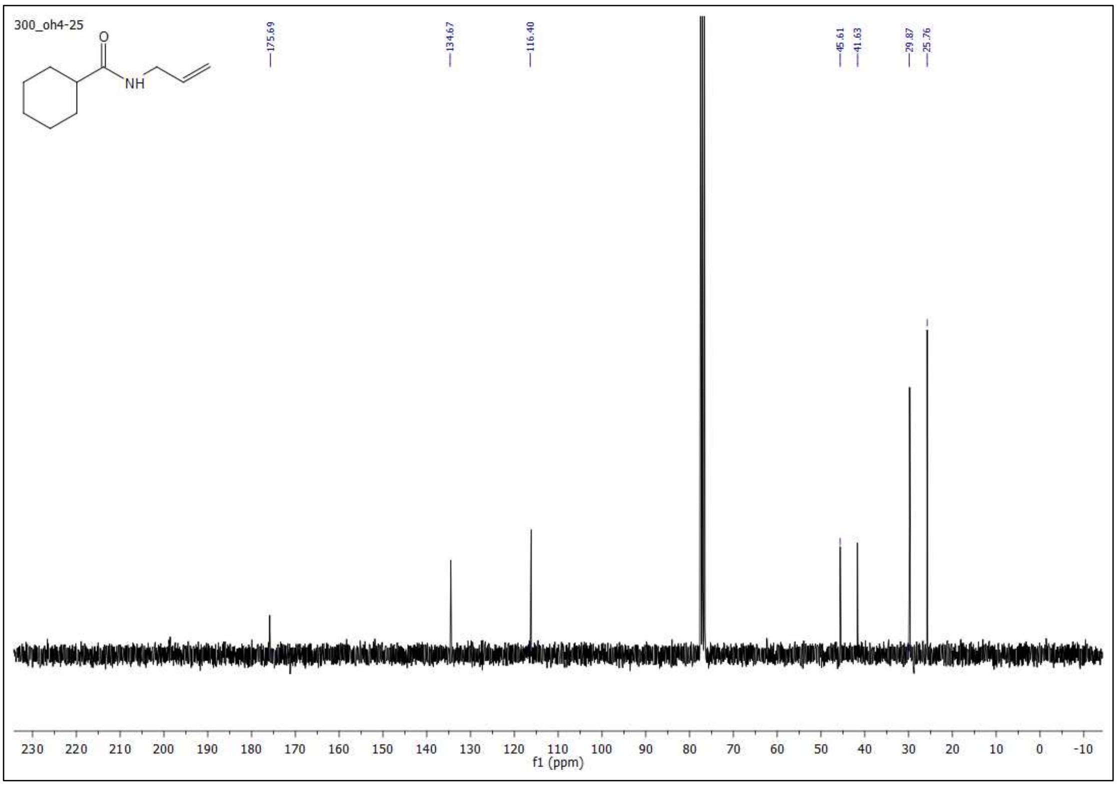

^{1}H NMR (300 MHz, $CDCl_3$) of *N*-allylcyclobutanecarboxamide (5x)

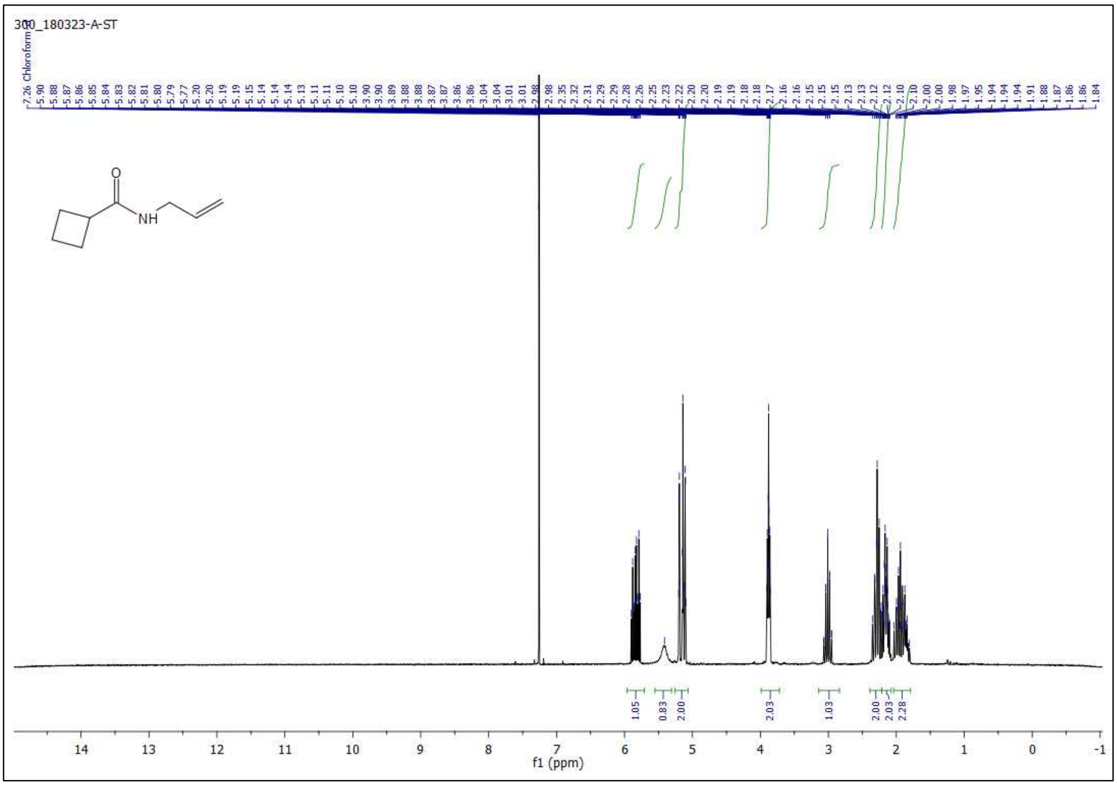

^{13}C {^{1}H} NMR (75 MHz, $CDCl_3$) of *N*-allylcyclobutanecarboxamide (5x)

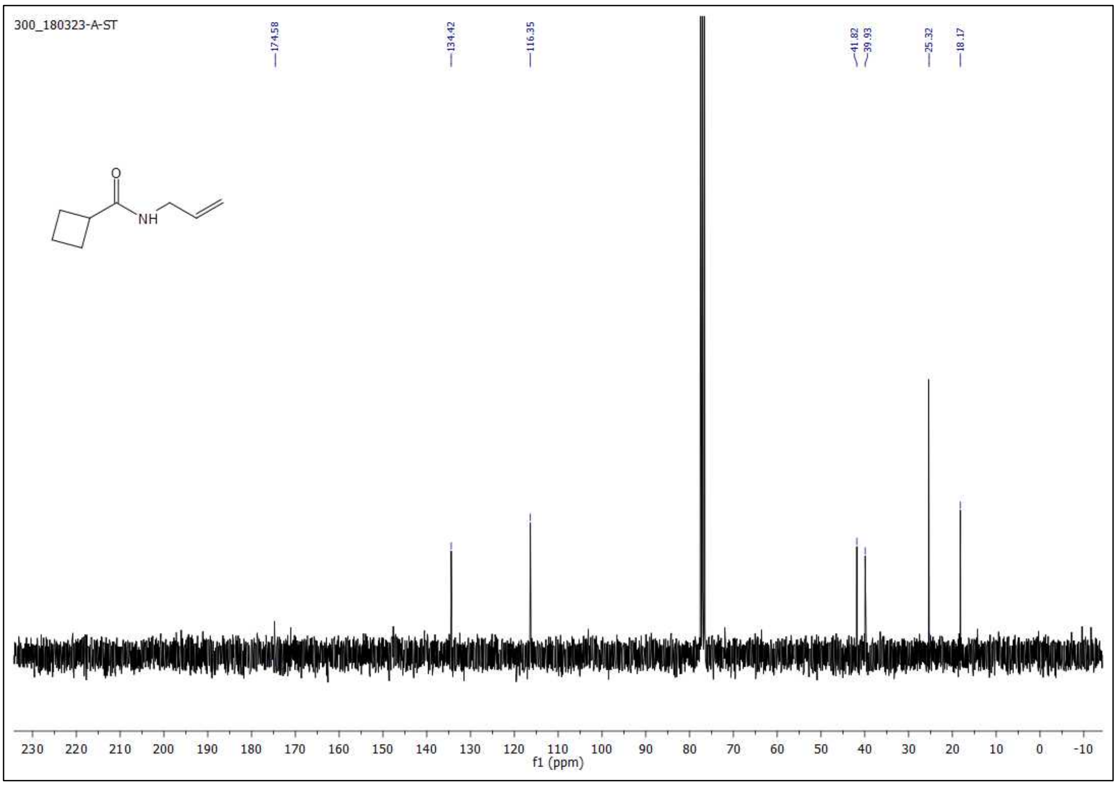

^{1}H NMR (300 MHz, $CDCl_3$) of *N*-allylcyclopropanecarboxamide (5y)

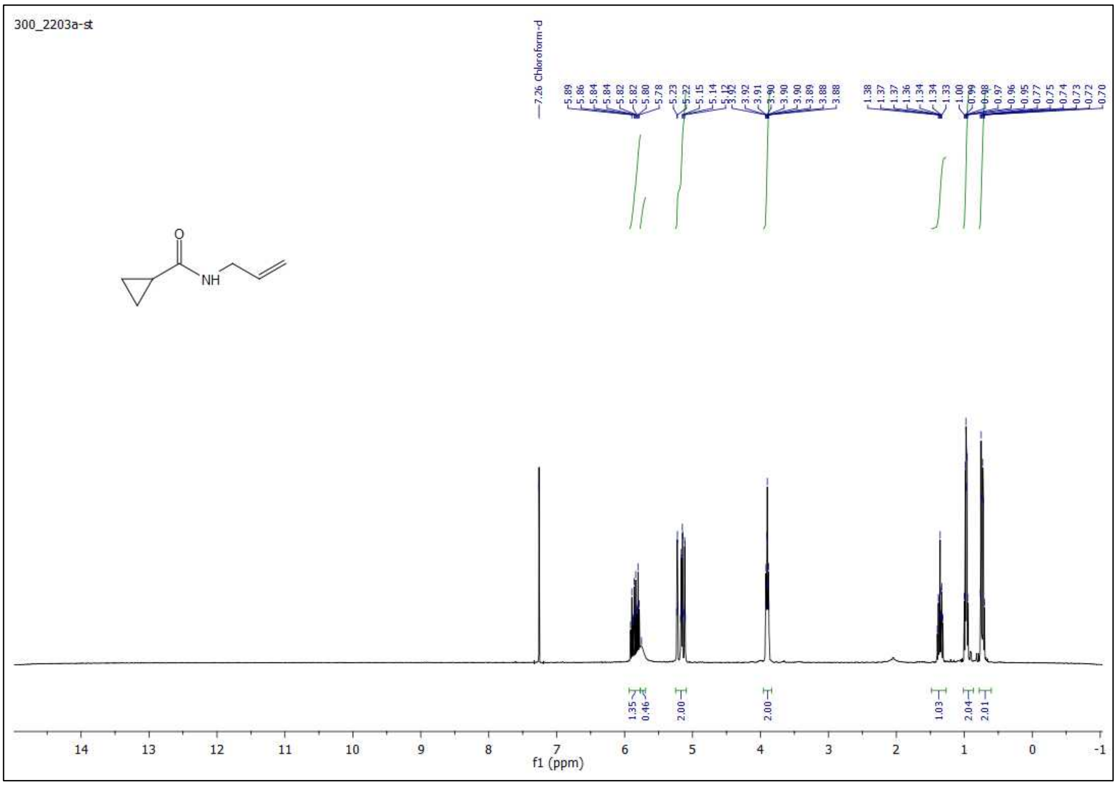

^{13}C {^{1}H} NMR (75 MHz, $CDCl_3$) of *N*-allylcyclopropanecarboxamide (5y)

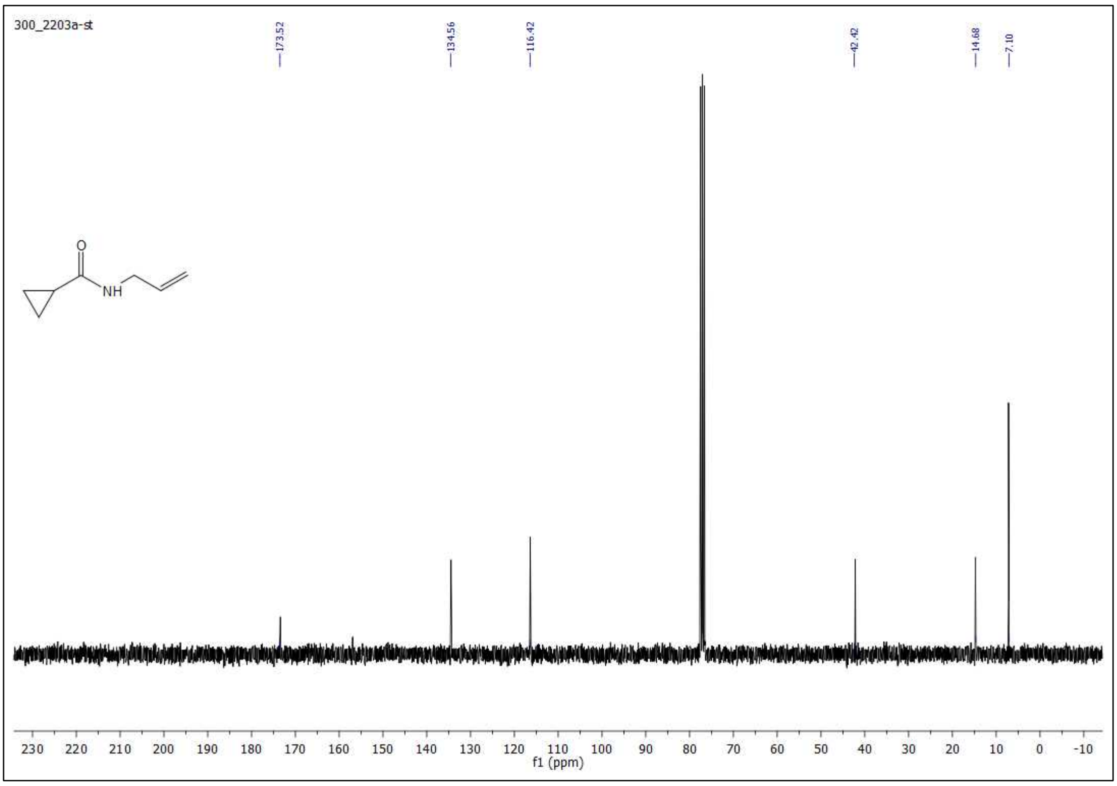

^{1}H NMR (300 MHz, $CDCl_3$) of *N*-allylpivalamide (5z)

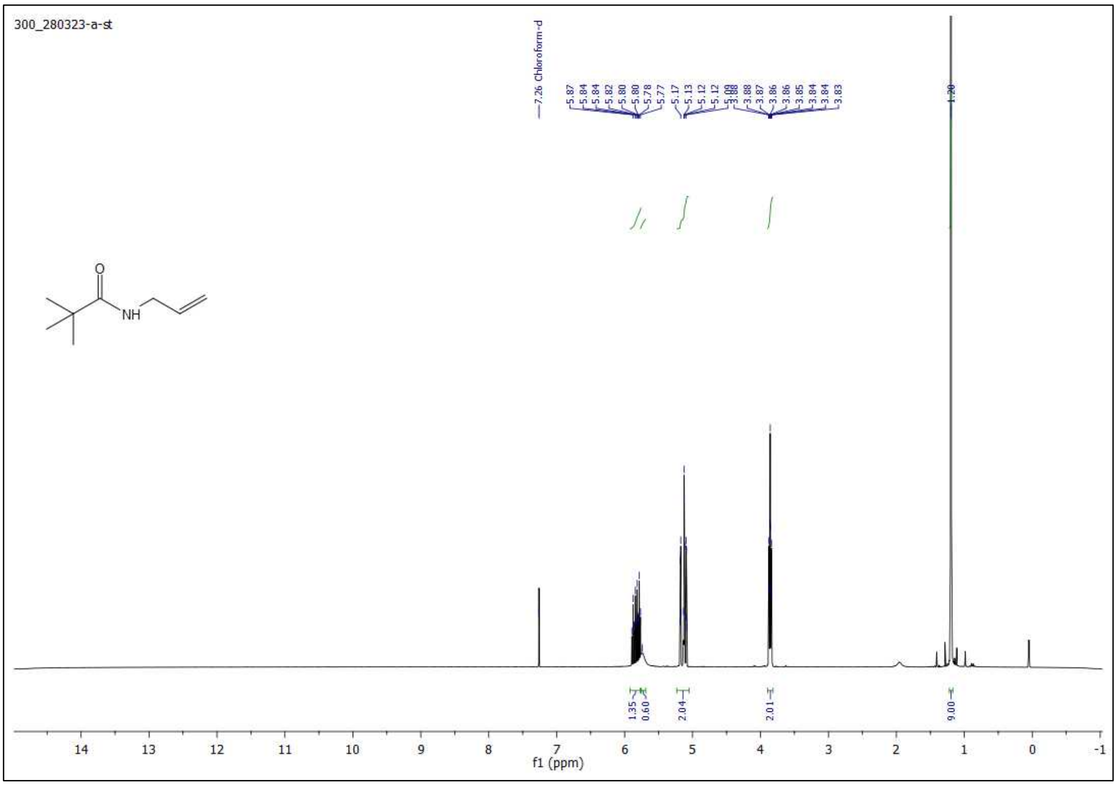

^{13}C {^{1}H} NMR (75 MHz, $CDCl_3$) of *N*-allylpivalamide (5z)

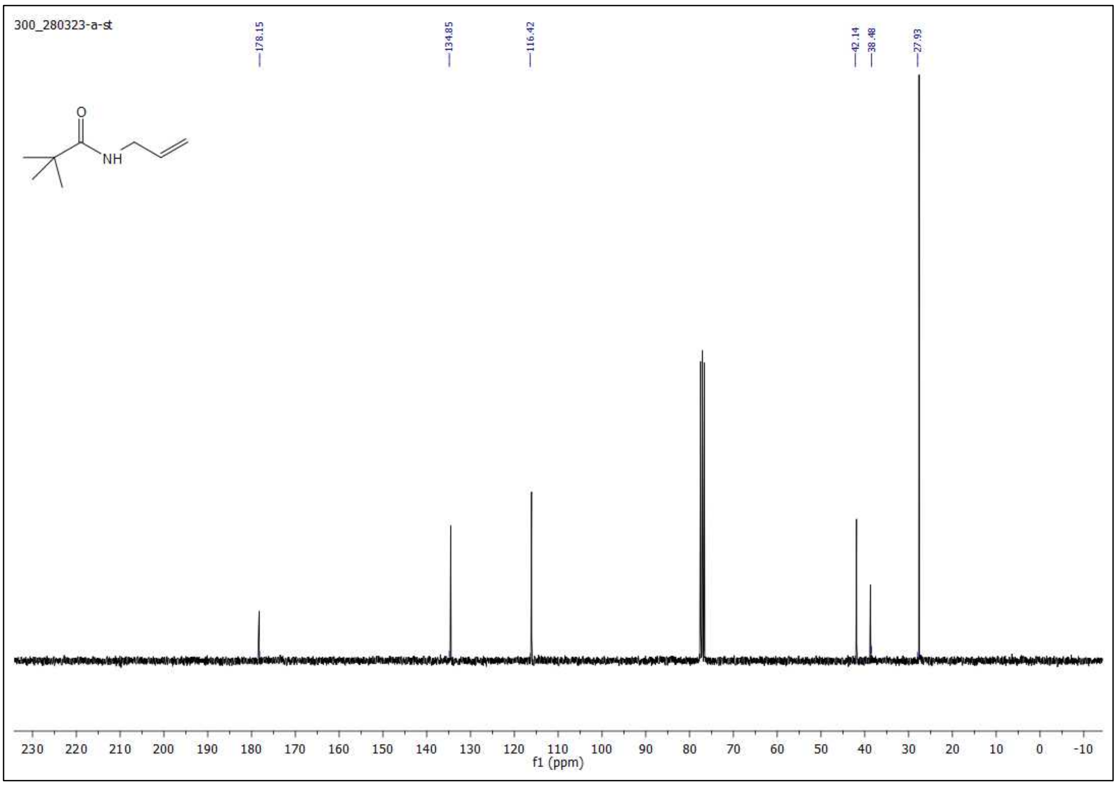

^{1}H NMR (300 MHz, $CDCl_3$) of *N*-allyl-2-naphthamide (5aa)

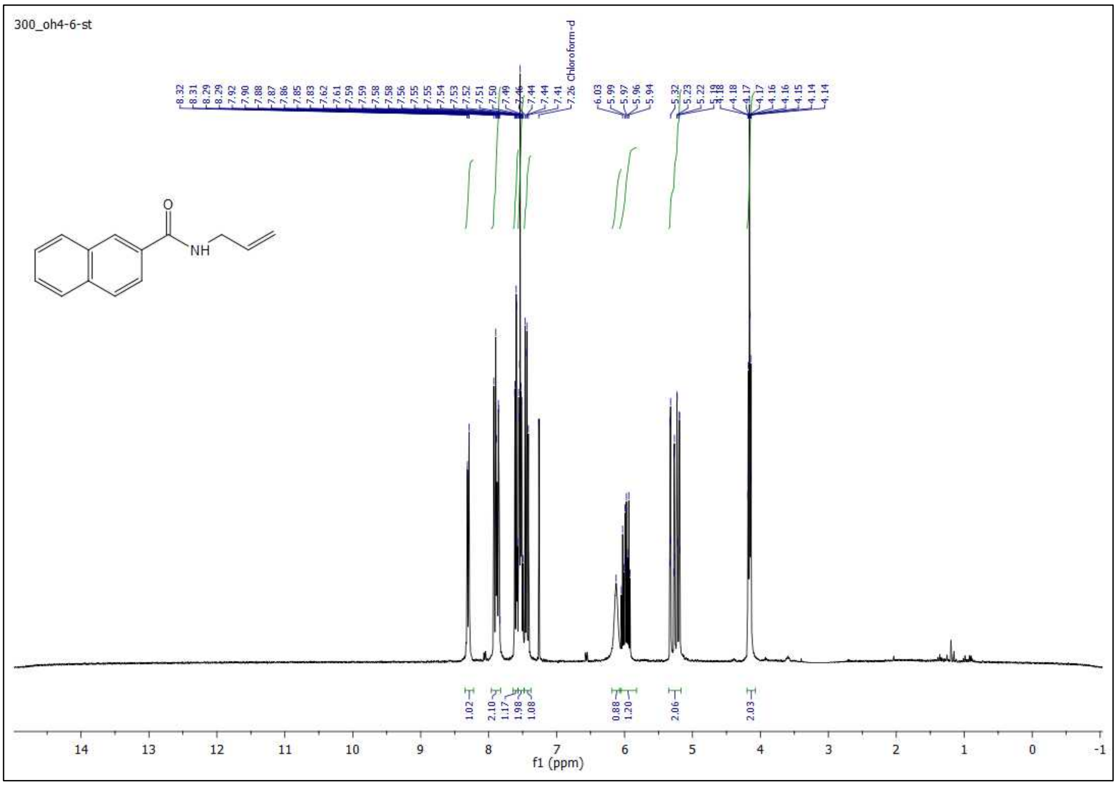

^{13}C {^{1}H} NMR (75 MHz, $CDCl_3$) of *N*-allyl-2-naphthamide (5aa)

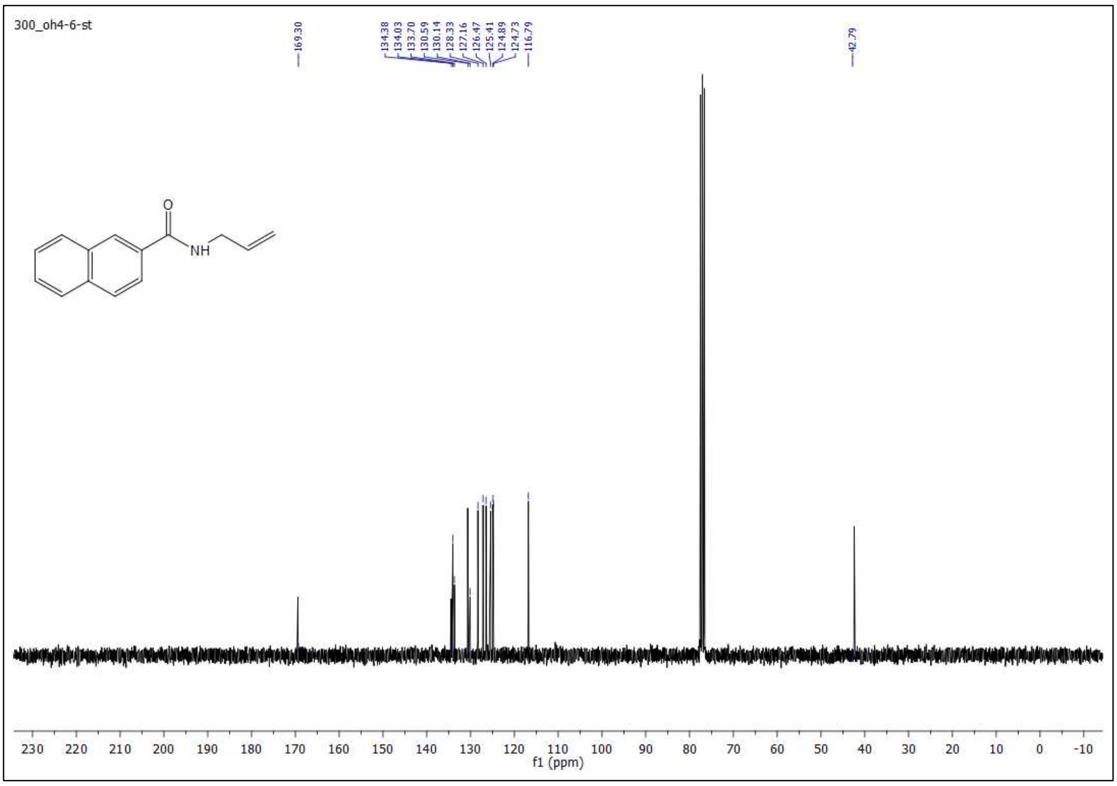

^{1}H NMR (300 MHz, $CDCl_3$) of *N*-(2-methylallyl)benzamide (5ab)

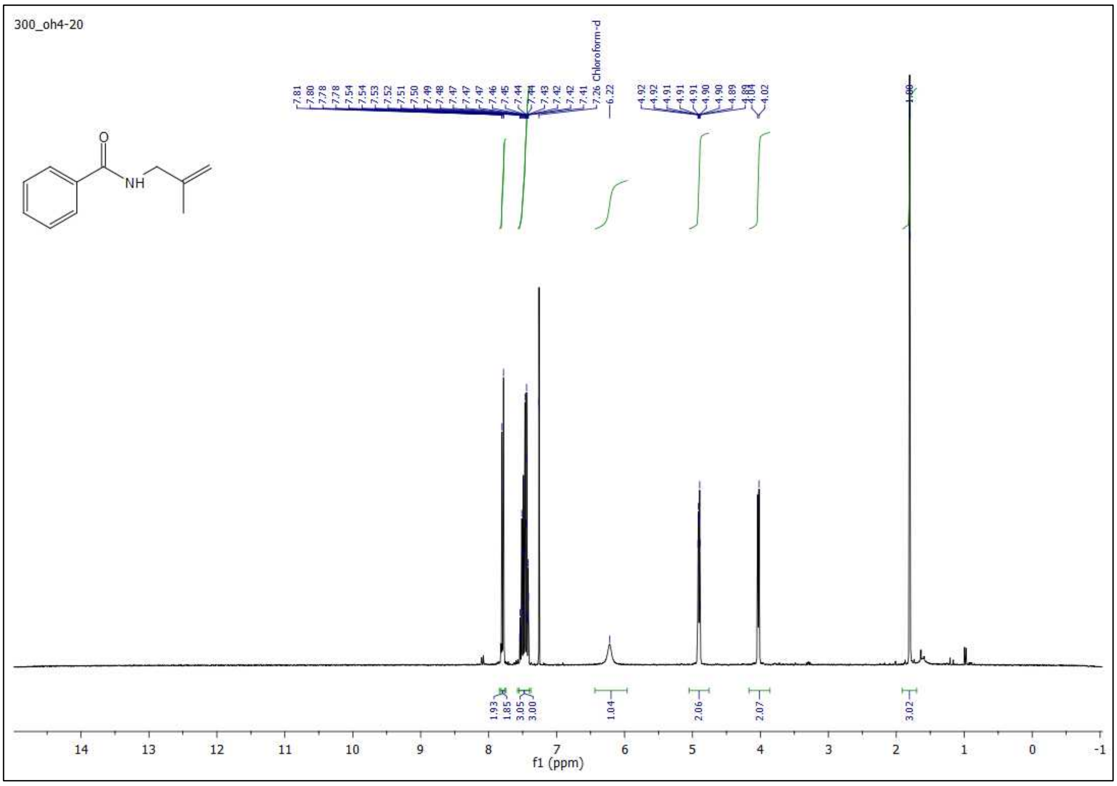

^{13}C {^{1}H} NMR (75 MHz, $CDCl_3$) of *N*-(2-methylallyl)benzamide (5ab)

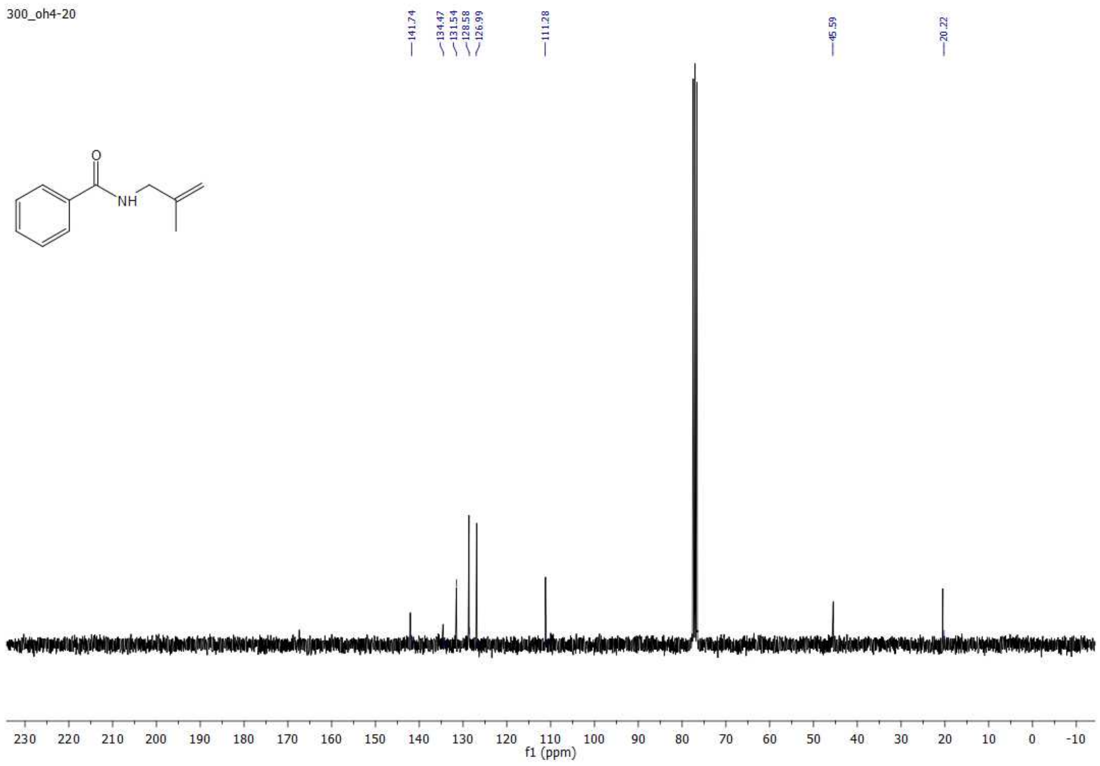

^{1}H NMR (300 MHz, $CDCl_3$) of 4-chloro-*N*-(2-methylallyl)benzamide (5ac)

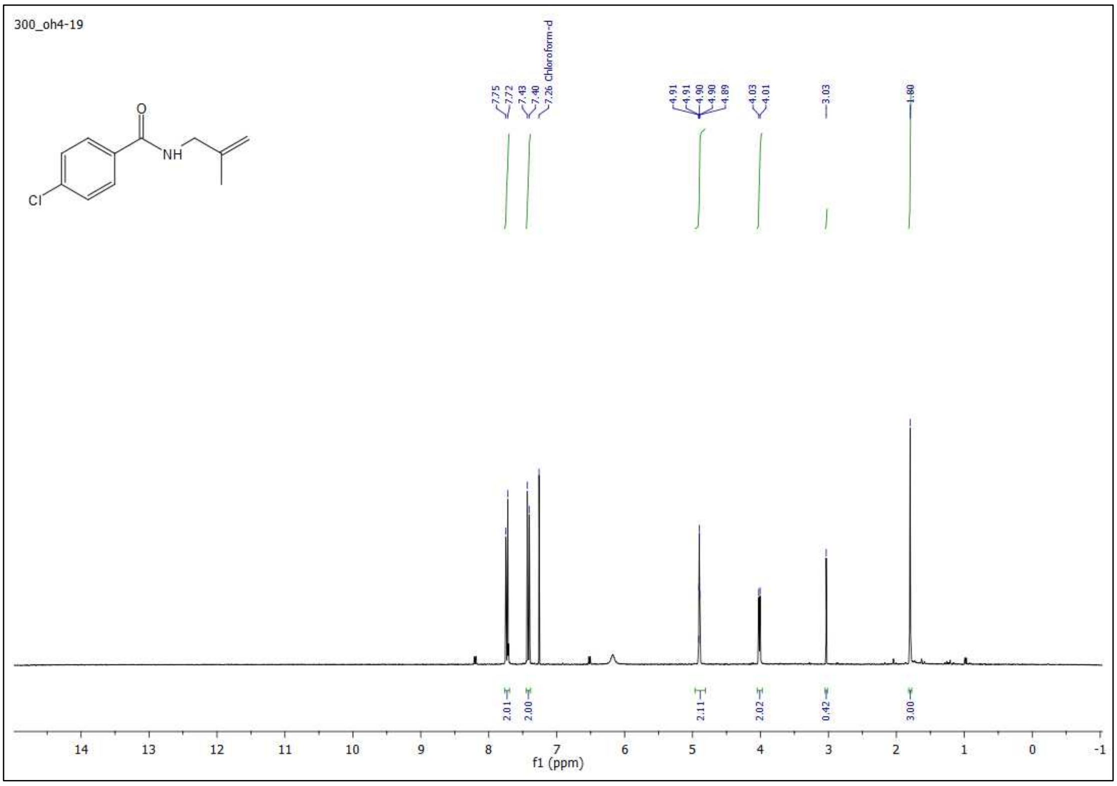

^{13}C {^{1}H} NMR (75 MHz, $CDCl_3$) of 4-chloro-*N*-(2-methylallyl)benzamide (5ac)

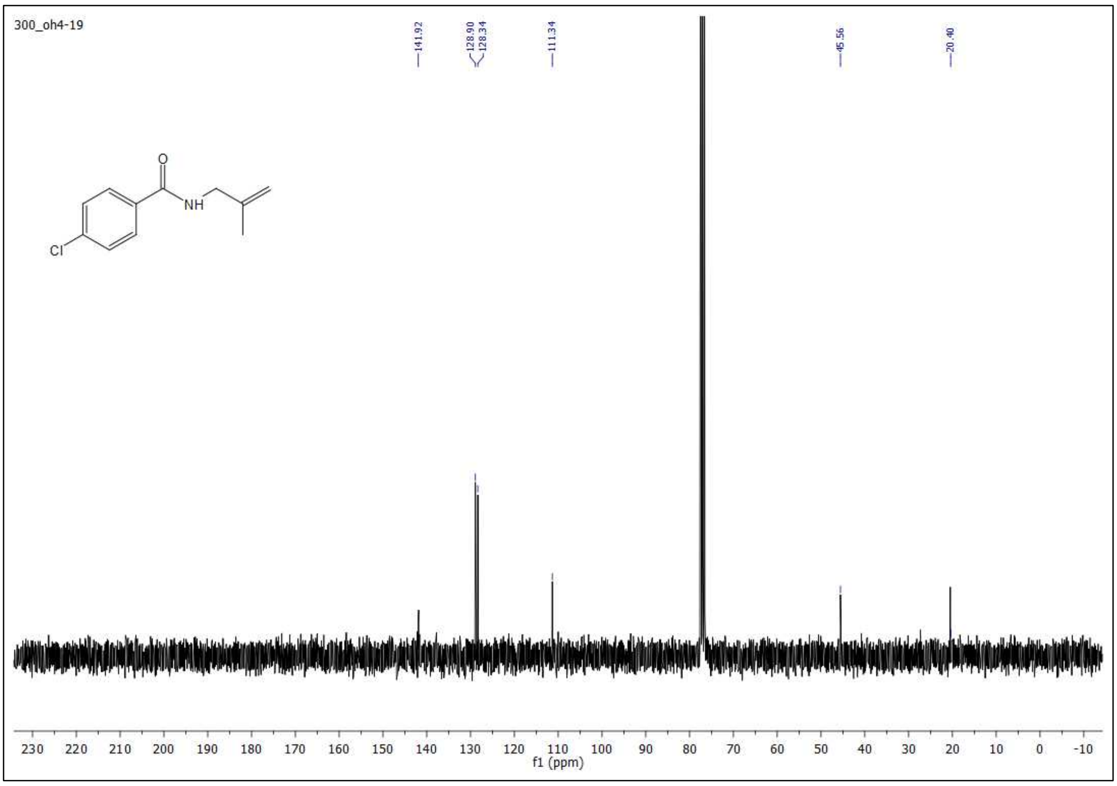

^{1}H NMR (300 MHz, $CDCl_3$) of *N*-(prop-2-yn-1-yl)benzamide (5ad)

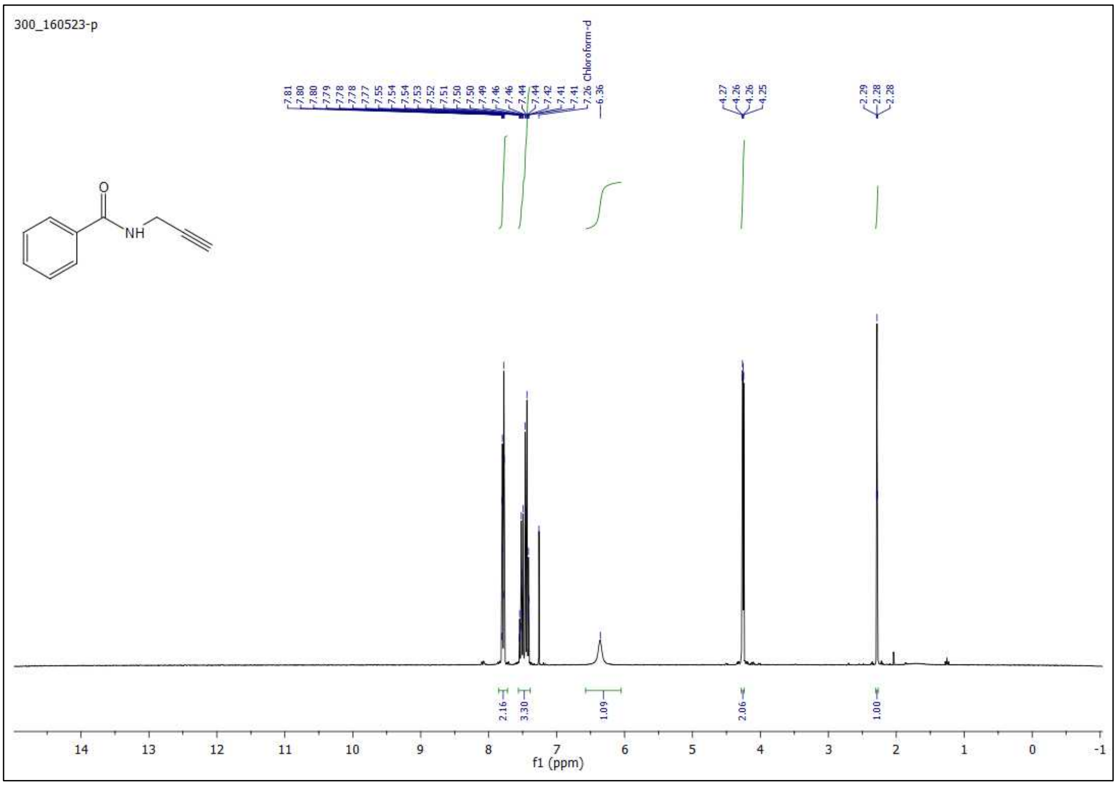

^{13}C {^{1}H} NMR (75 MHz, $CDCl_3$) of *N*-(prop-2-yn-1-yl)benzamide (5ad)

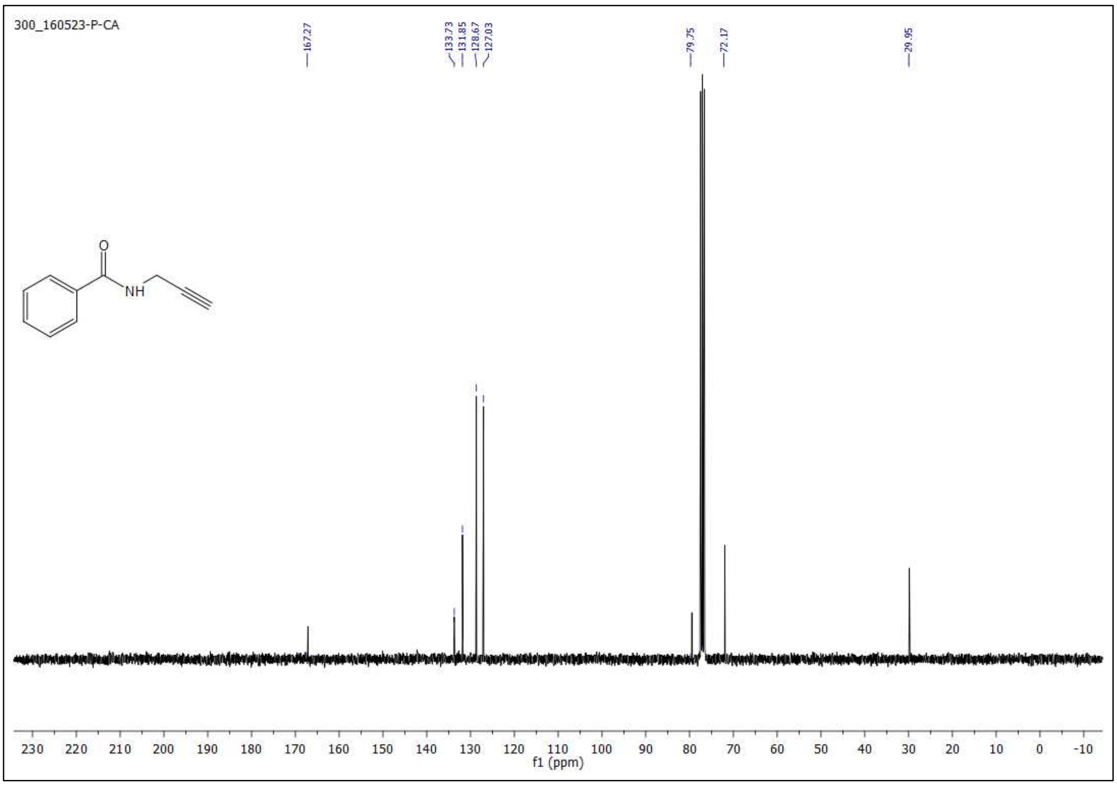

^{1}H NMR (300 MHz, $CDCl_3$) of 2-phenyl-5-((phenylselanyl)methyl)-4,5-dihydrooxazole (6a)

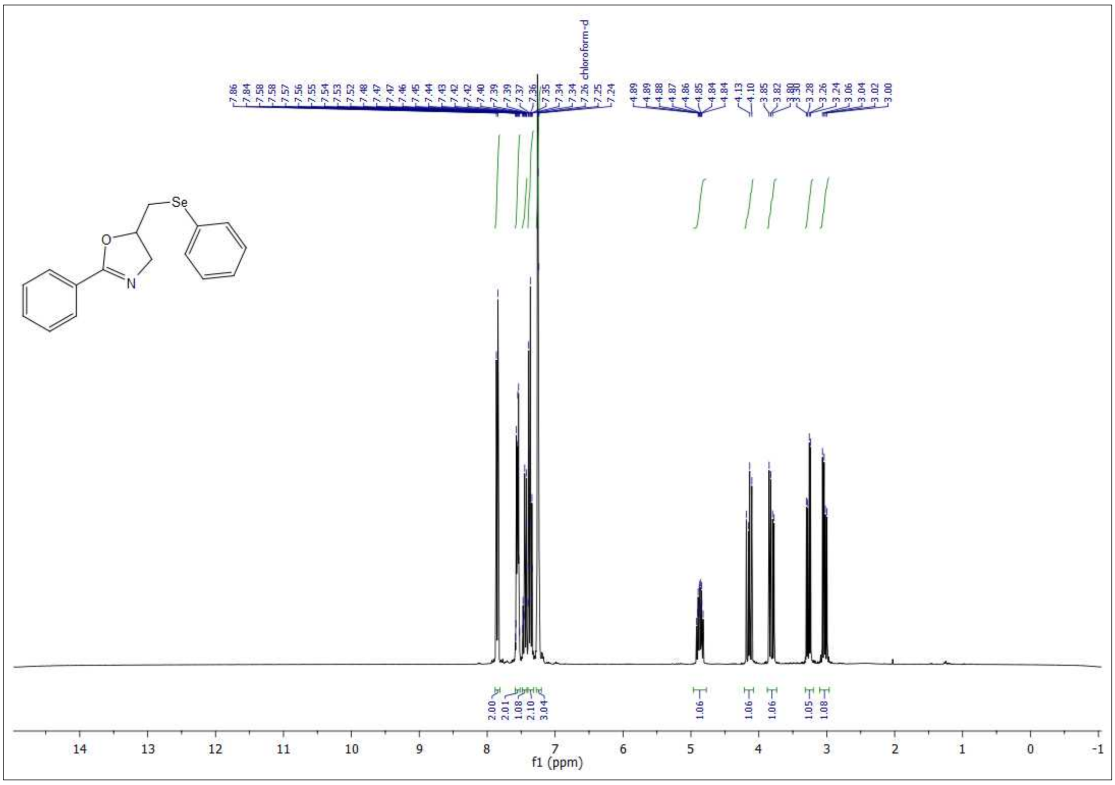

$^{13}C\{^{1}H\}$ NMR (75 MHz, $CDCl_3$) of 2-phenyl-5-((phenylselanyl)methyl)-4,5-dihydrooxazole (6a)

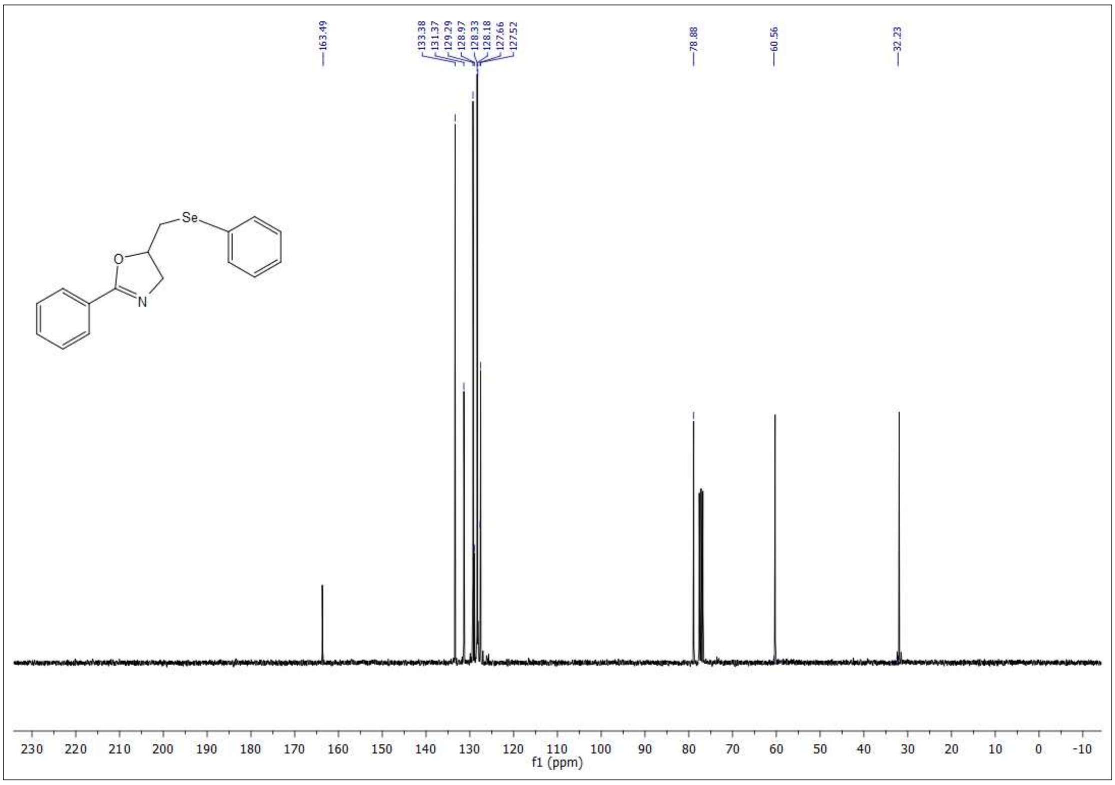

^{1}H NMR (300 MHz, $CDCl_3$) of 5-((phenylselanyl)methyl)-2-(4-(trifluoromethyl)phenyl)-4,5-dihydrooxazole (6b)

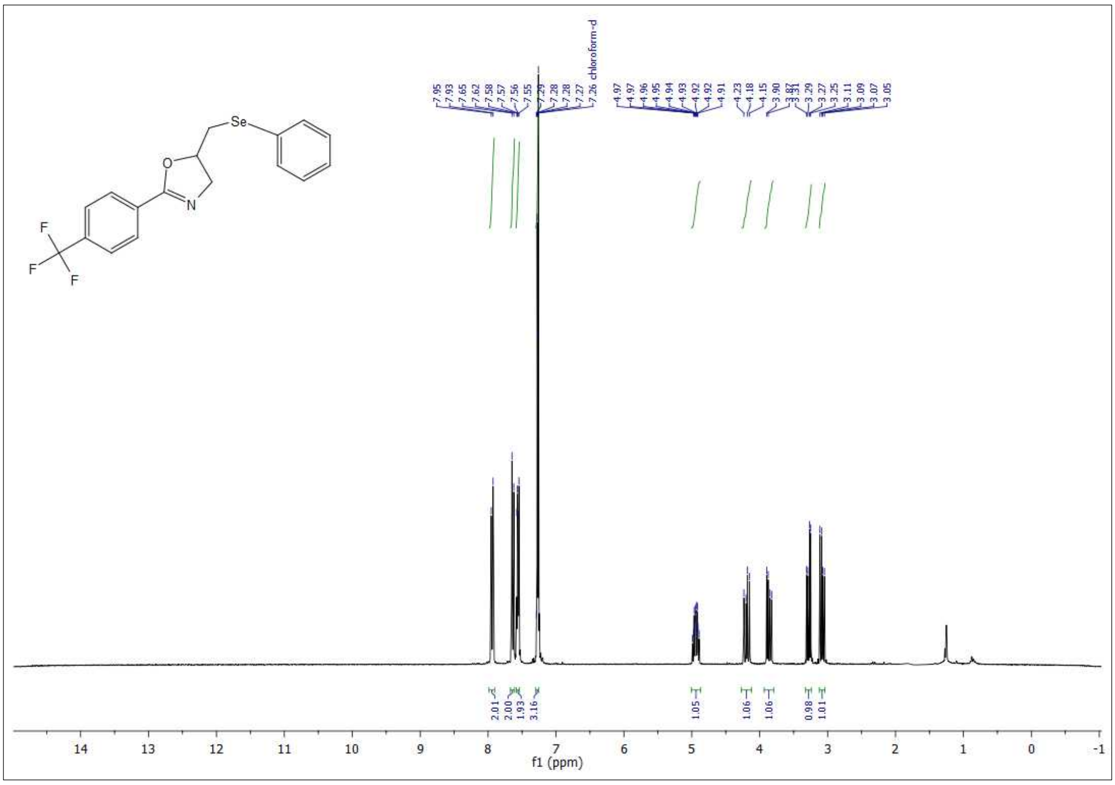

^{13}C {^{1}H} NMR (75 MHz, $CDCl_3$) of 5-((phenylselanyl)methyl)-2-(4-(trifluoromethyl)phenyl)-4,5-dihydrooxazole (6b)

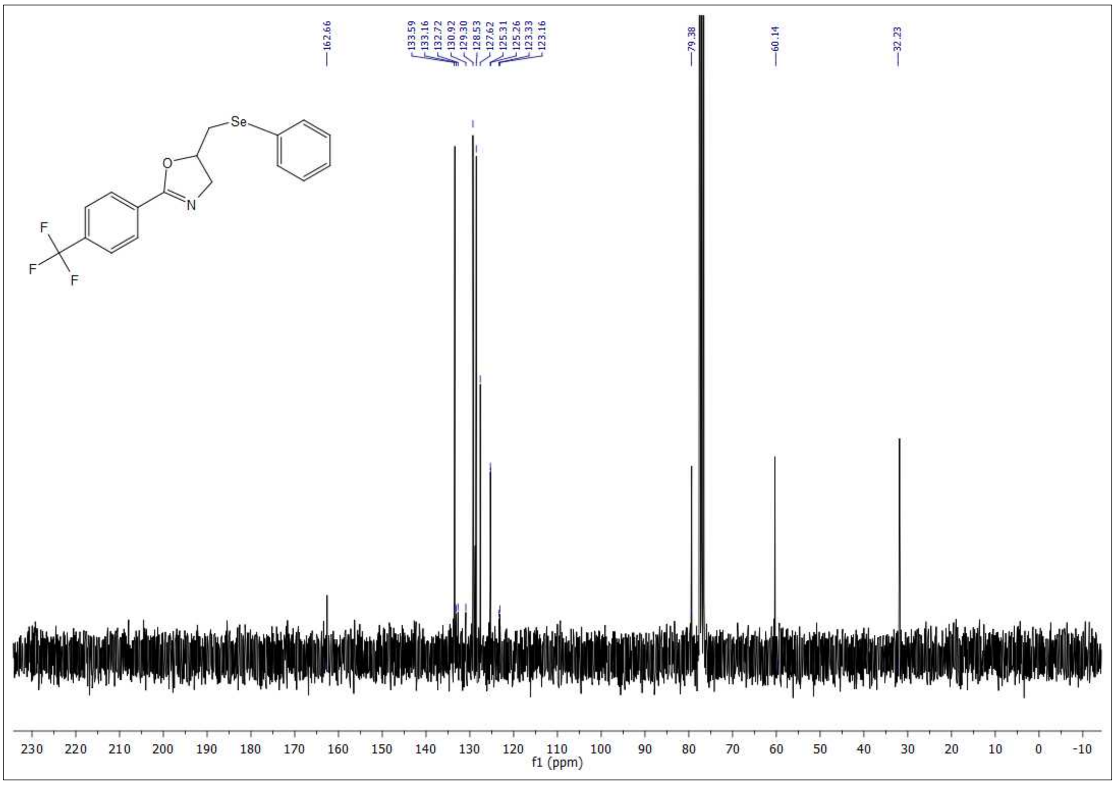

^{19}F NMR (471 MHz, $CDCl_3$) of 5-((phenylselanyl)methyl)-2-(4-(trifluoromethyl)phenyl)-4,5-dihydrooxazole (6b)

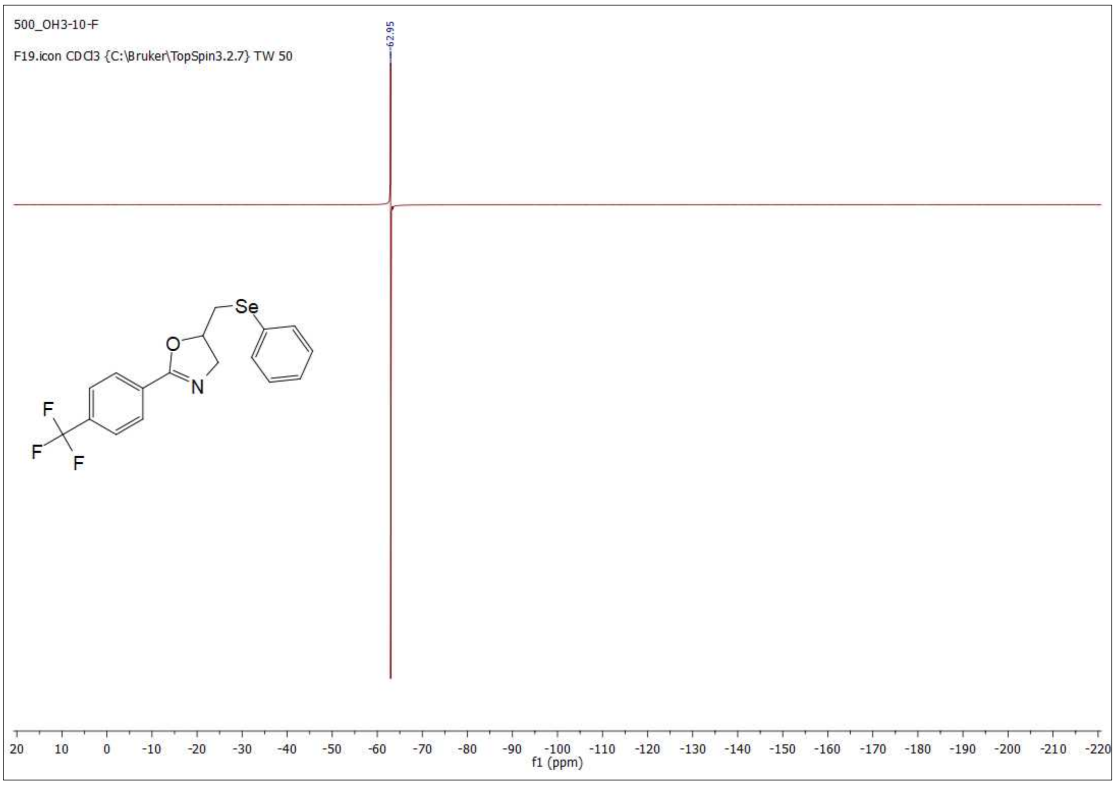

^{1}H NMR (300 MHz, $CDCl_3$) of 2-(4-iodophenyl)-5-((phenylselanyl)methyl)-4,5-dihydrooxazole (6c)

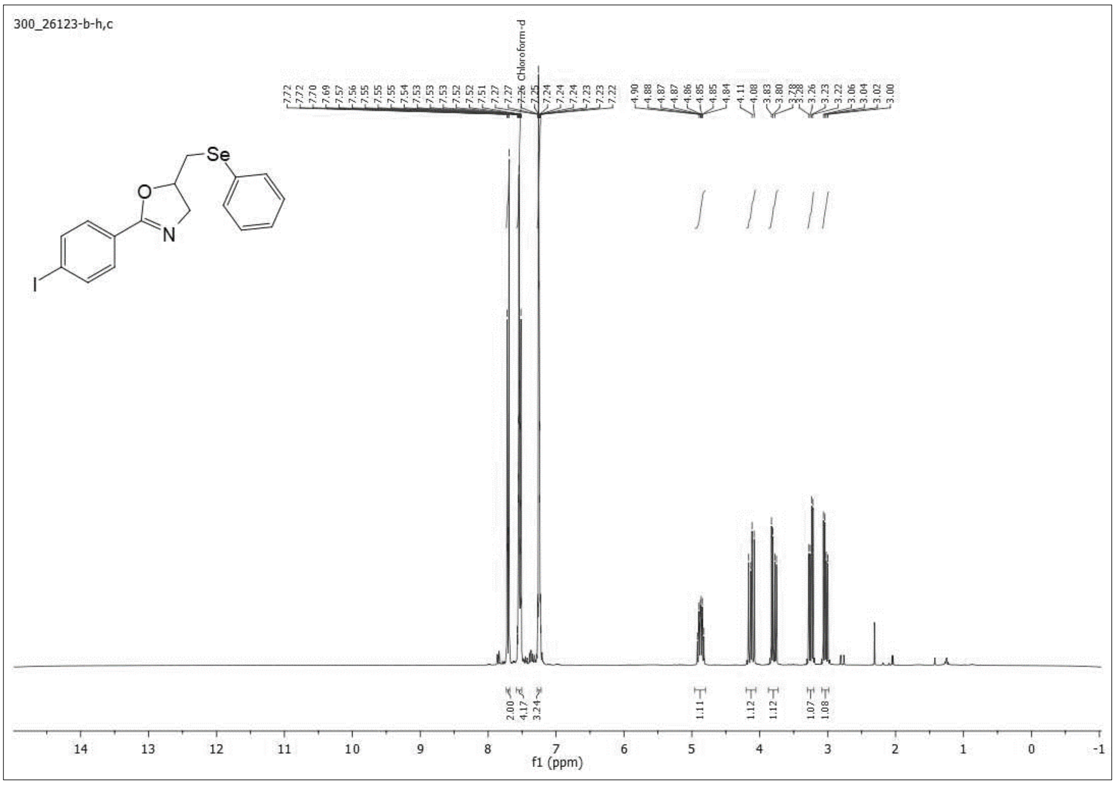

^{13}C {^{1}H} NMR (75 MHz, $CDCl_3$) of 2-(4-iodophenyl)-5-((phenylselanyl)methyl)-4,5-dihydrooxazole (6c)

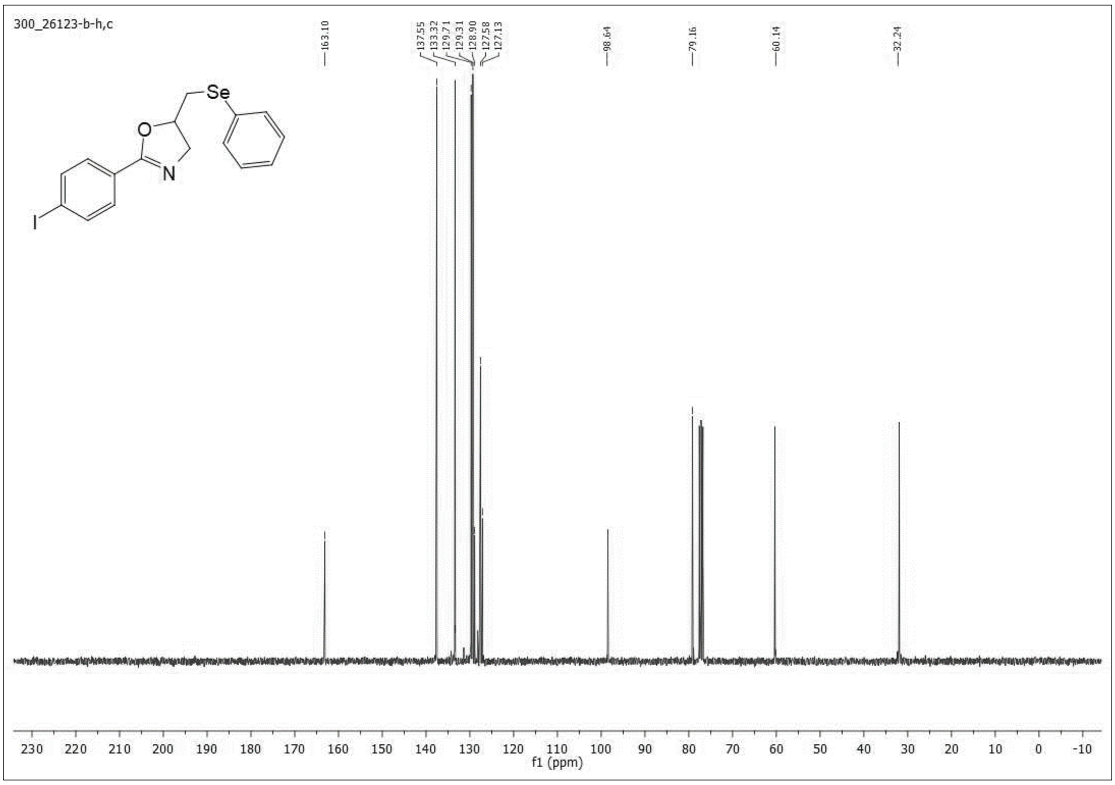

^{1}H NMR (300 MHz, $CDCl_3$) of 2-(4-bromophenyl)-5-((phenylselanyl)methyl)-4,5-dihydrooxazole (6d)

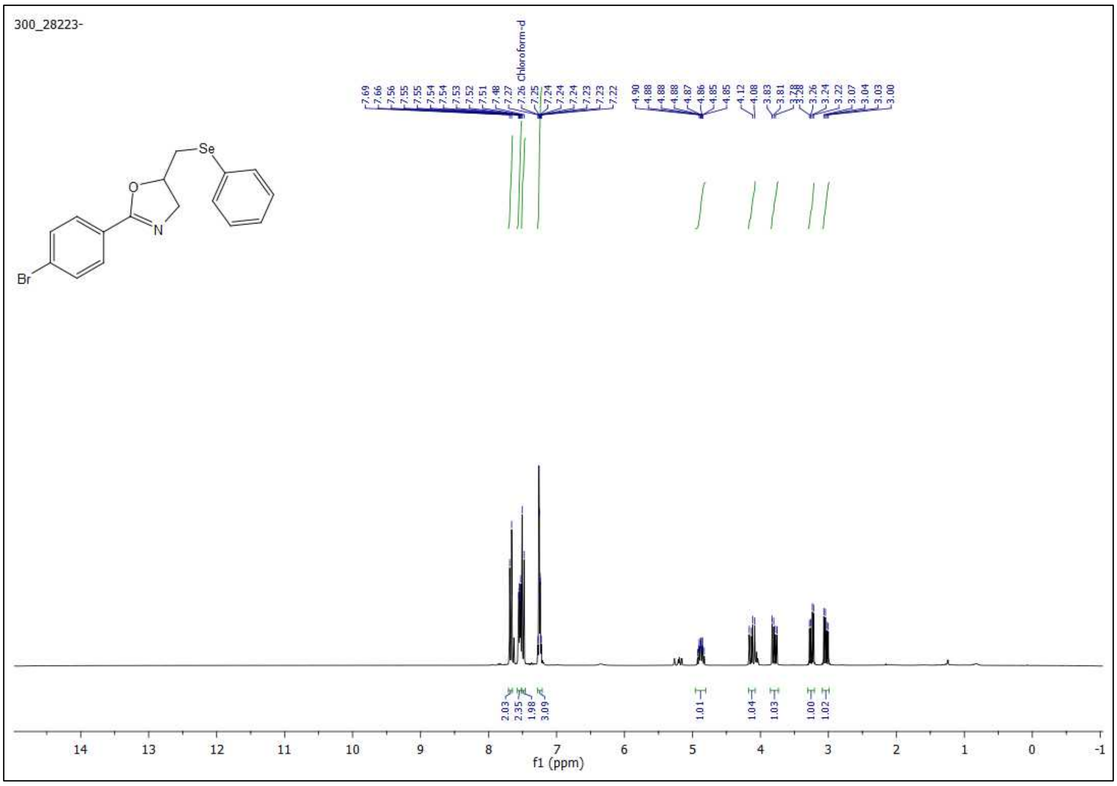

^{13}C {^{1}H} NMR (75 MHz, $CDCl_3$) of 2-(4-bromophenyl)-5-((phenylselanyl)methyl)-4,5-dihydrooxazole (6d)

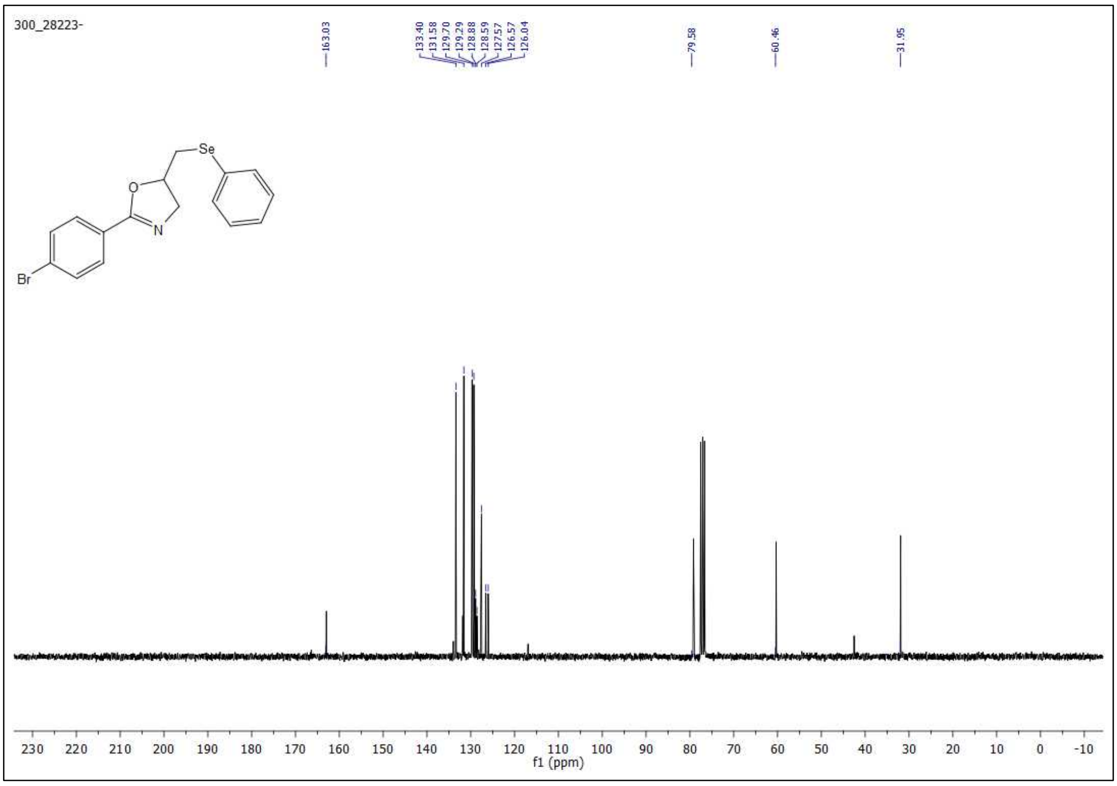

^{1}H NMR (300 MHz, $CDCl_3$) of 2-(4-chlorophenyl)-5-((phenylselanyl)methyl)-4,5-dihydrooxazole (6e)

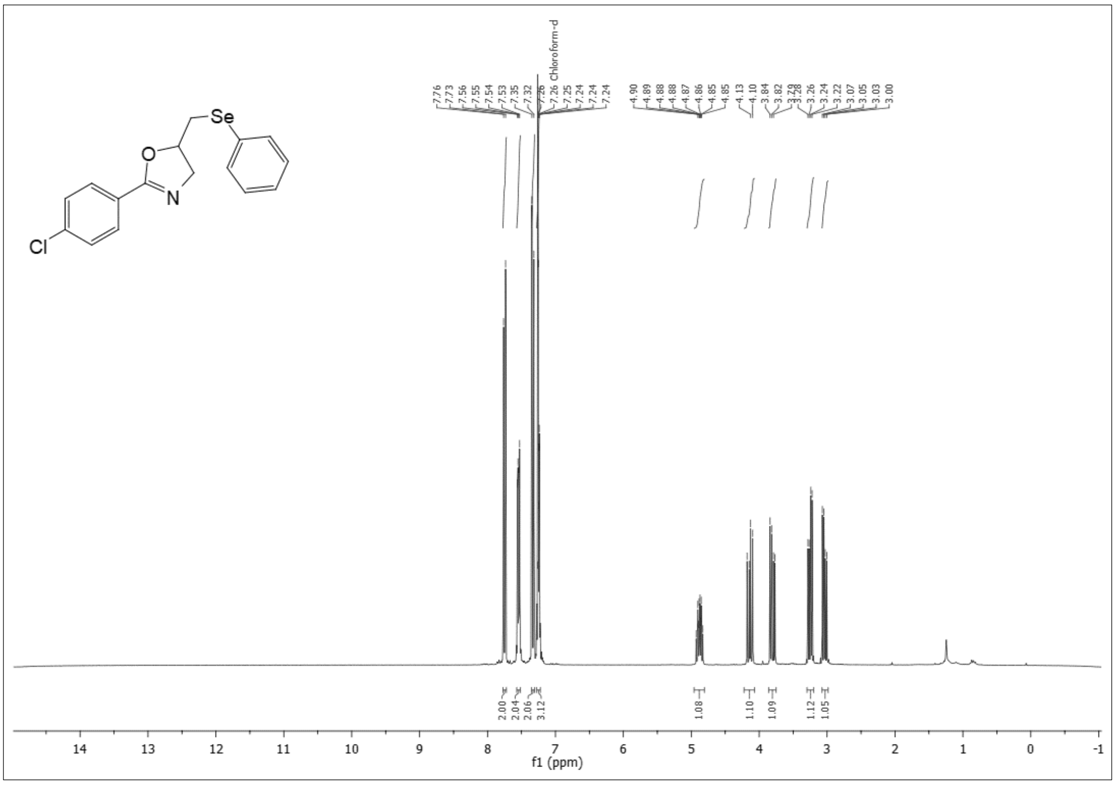

^{13}C {^{1}H} NMR (75 MHz, $CDCl_3$) of 2-(4-chlorophenyl)-5-((phenylselanyl)methyl)-4,5-dihydrooxazole (6e)

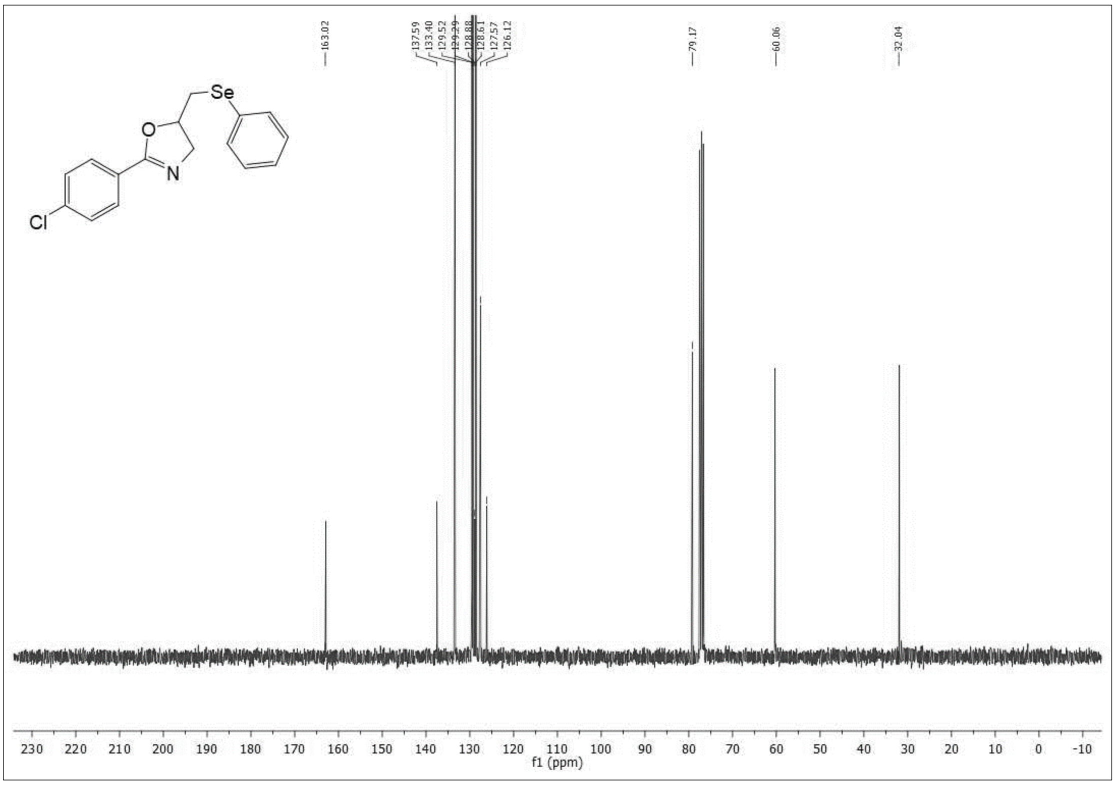

^{1}H NMR (300 MHz, $CDCl_3$) of 2-(4-methoxyphenyl)-5-((phenylselanyl)methyl)-4,5-dihydrooxazole (6f)

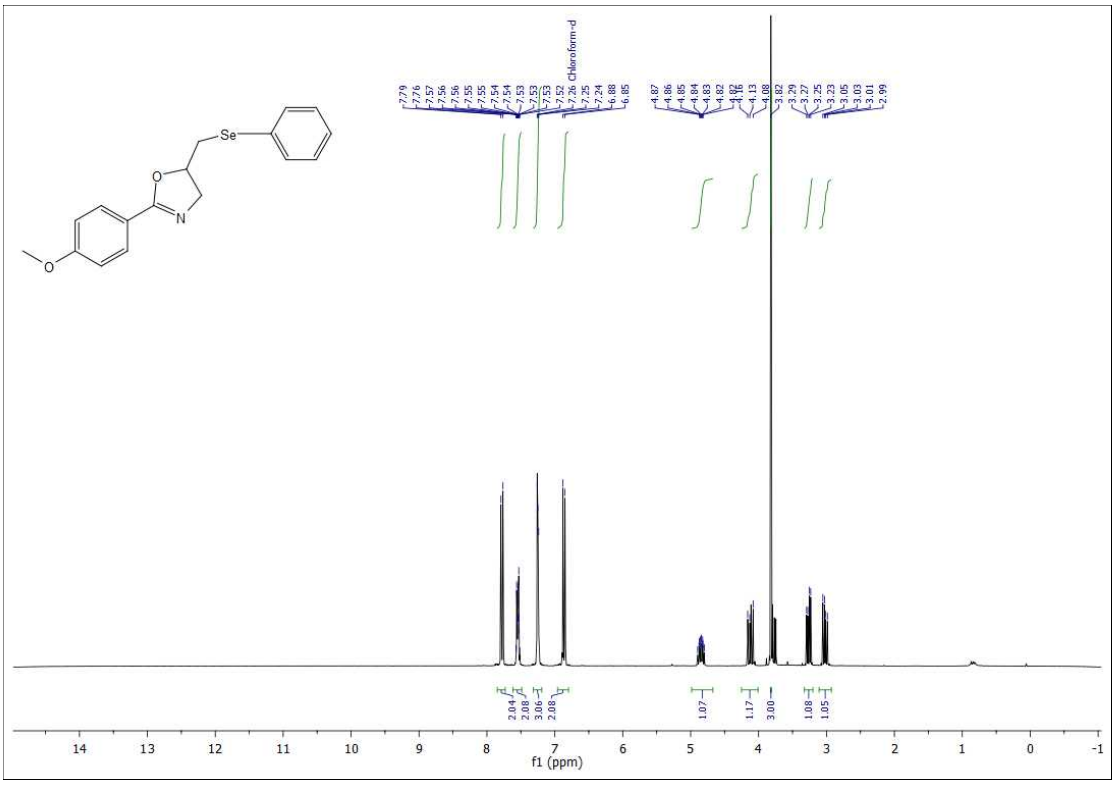

^{13}C {^{1}H} NMR (75 MHz, $CDCl_3$) of 2-(4-methoxyphenyl)-5-((phenylselanyl)methyl)-4,5-dihydrooxazole (6f)

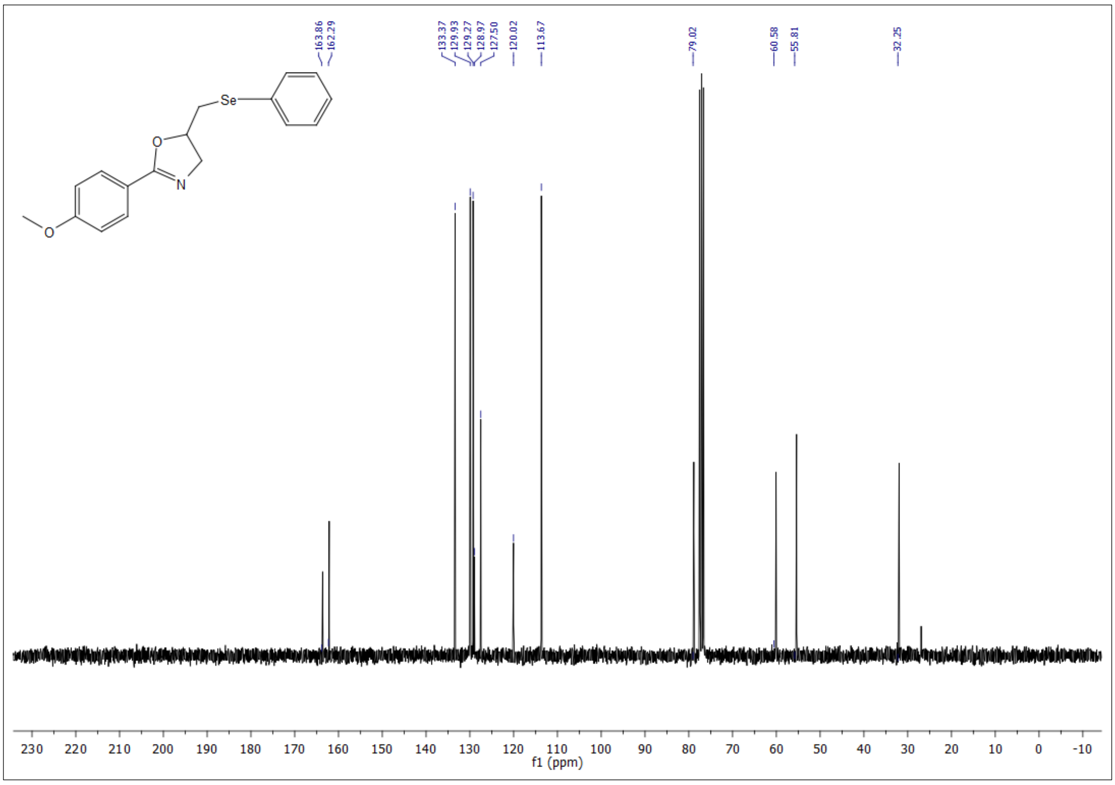

^{1}H NMR (300 MHz, $CDCl_3$) of 2-(4-isopropylphenyl)-5-((phenylselanyl)methyl)-4,5-dihydrooxazole (6g)

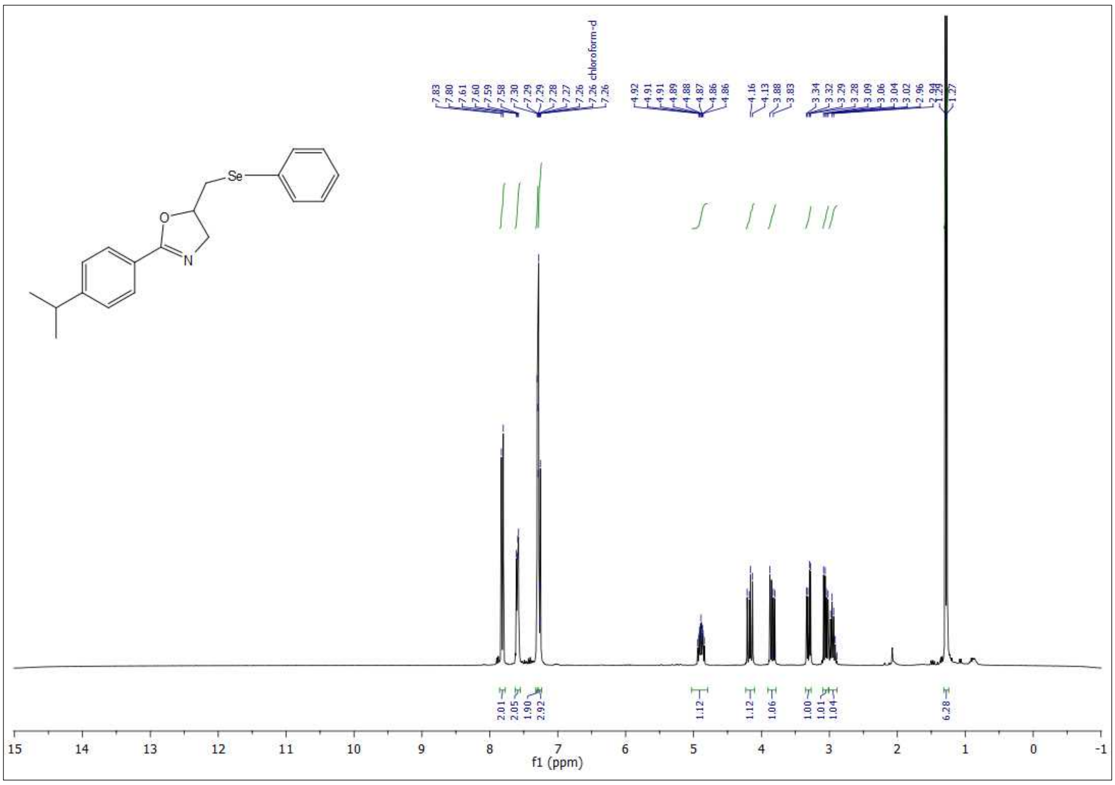

^{13}C {^{1}H} NMR (75 MHz, $CDCl_3$) of 2-(4-isopropylphenyl)-5-((phenylselanyl)methyl)-4,5-dihydrooxazole (6g)

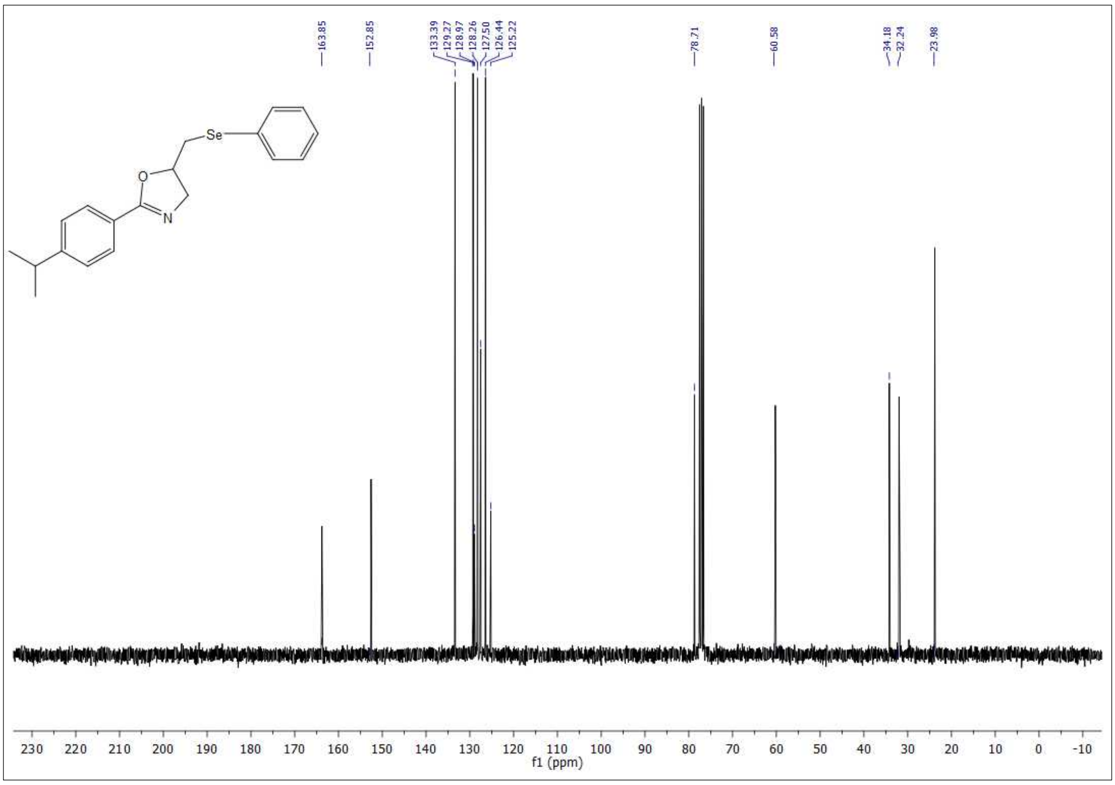

^{1}H NMR (300 MHz, $CDCl_3$) of methyl 4-(5-((phenylselanyl)methyl)-4,5-dihydrooxazol-2-yl)benzoate (6h)

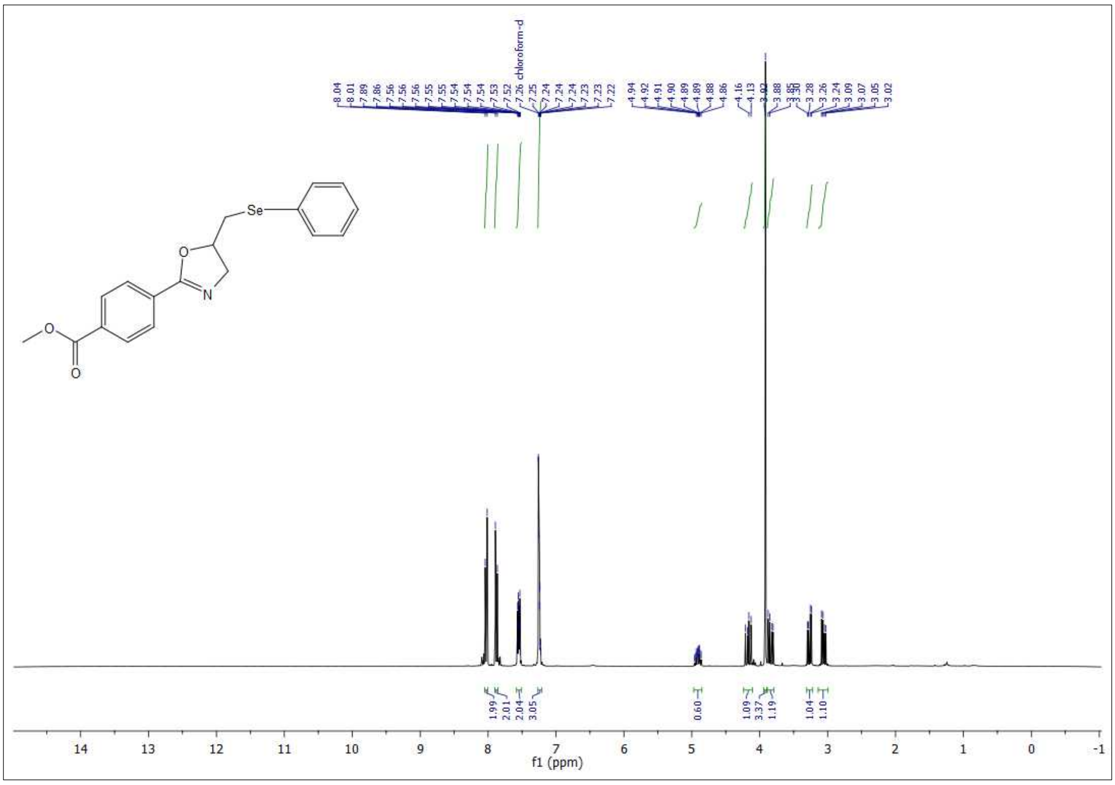

^{13}C {^{1}H} NMR (75 MHz, $CDCl_3$) of methyl 4-(5-((phenylselanyl)methyl)-4,5-dihydrooxazol-2-yl)benzoate (6h)

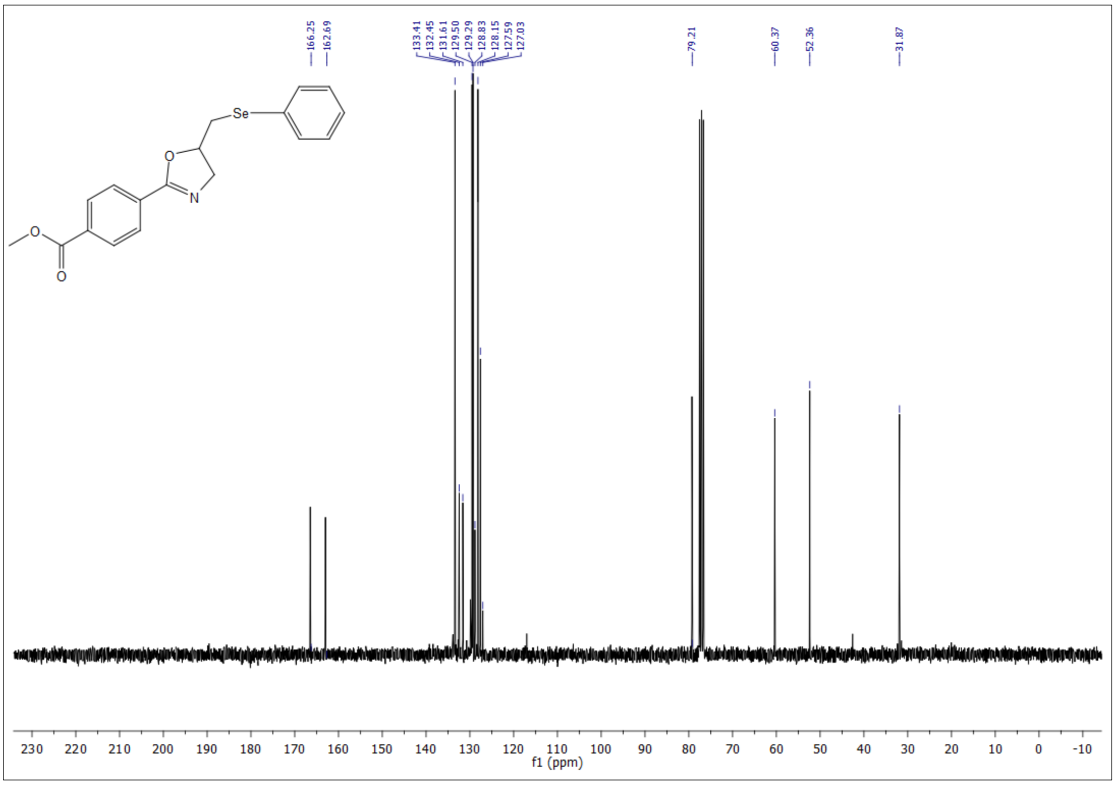

^{1}H NMR (300 MHz, $CDCl_3$) of 5-((phenylselanyl)methyl)-2-(m-tolyl)-4,5-dihydrooxazole (6i)

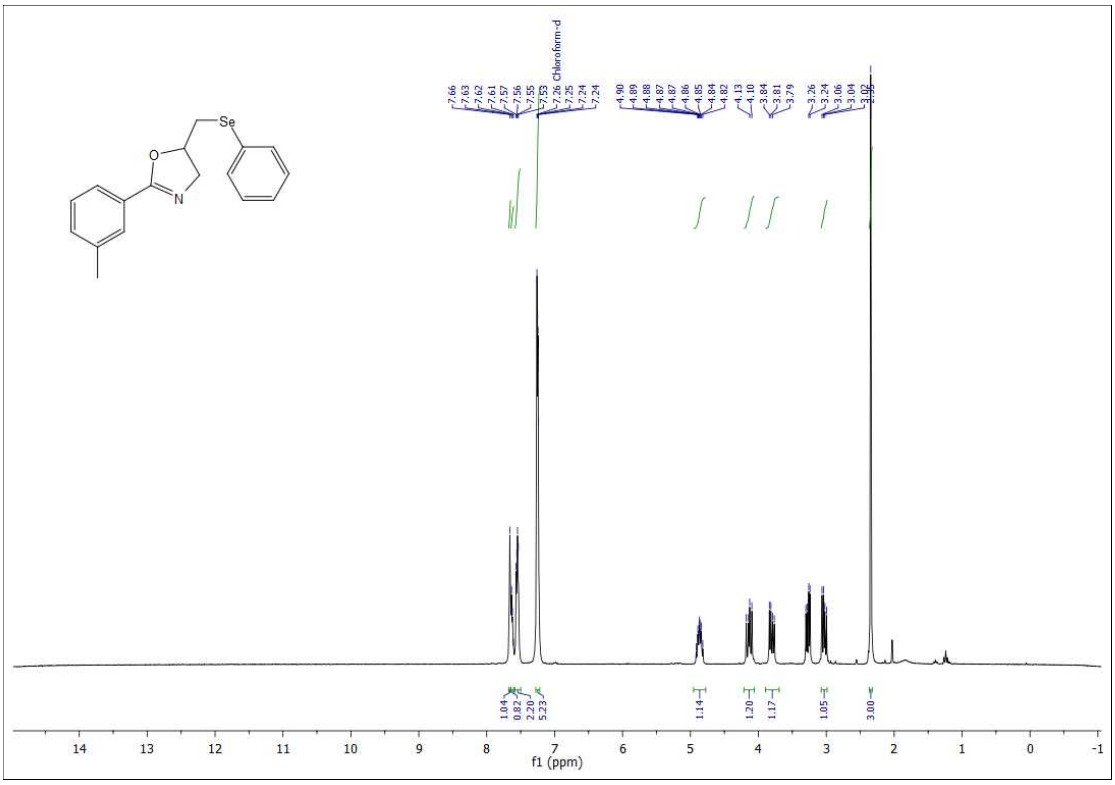

$^{13}C\ \{^{1}H\}$ NMR (75 MHz, $CDCl_3$) of 5-((phenylselanyl)methyl)-2-(m-tolyl)-4,5-dihydrooxazole (6i)

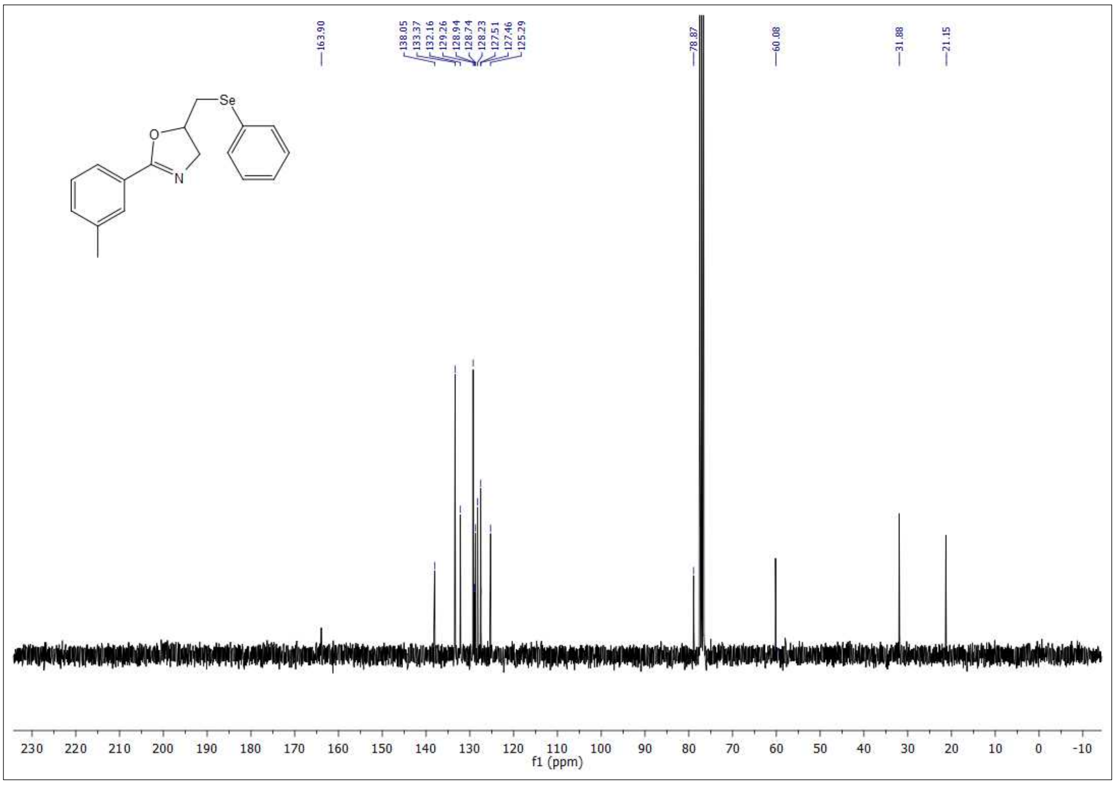

^{1}H NMR (300 MHz, $CDCl_3$) of 3-(5-((phenylselanyl)methyl)-4,5-dihydrooxazol-2-yl)benzonitrile (6j)

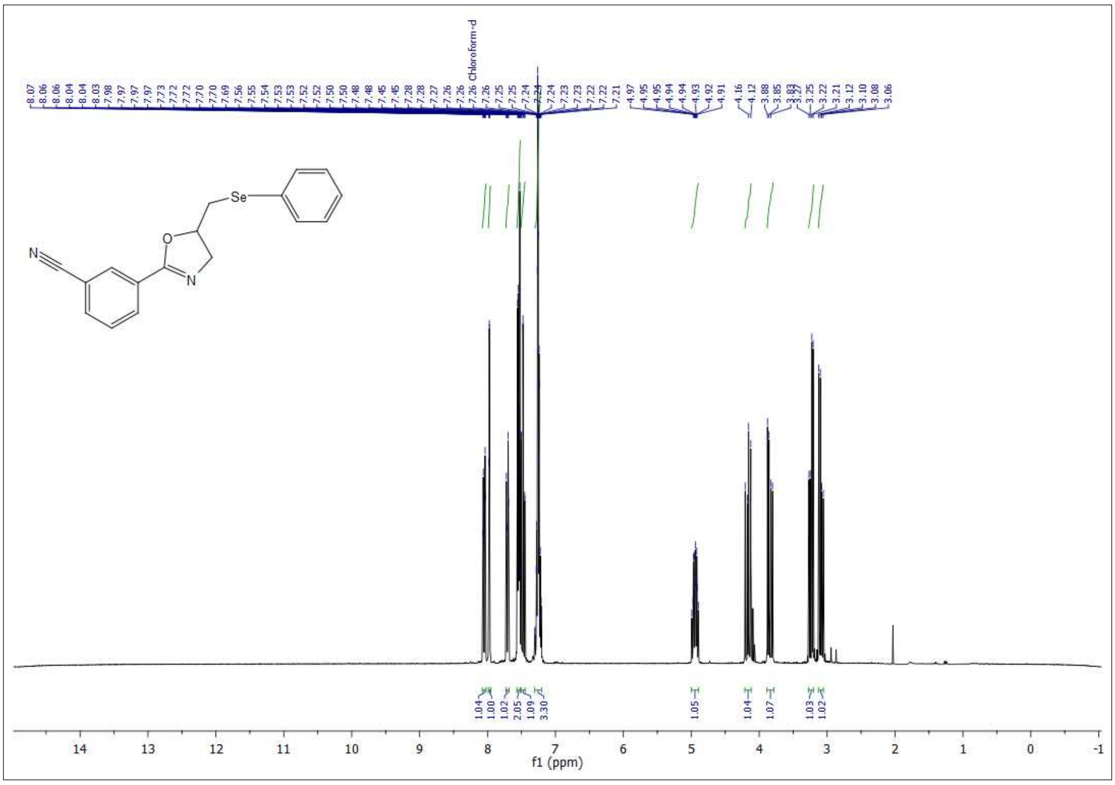

^{13}C {^{1}H} NMR (75 MHz, $CDCl_3$) of 3-(5-((phenylselanyl)methyl)-4,5-dihydrooxazol-2-yl)benzonitrile (6j)

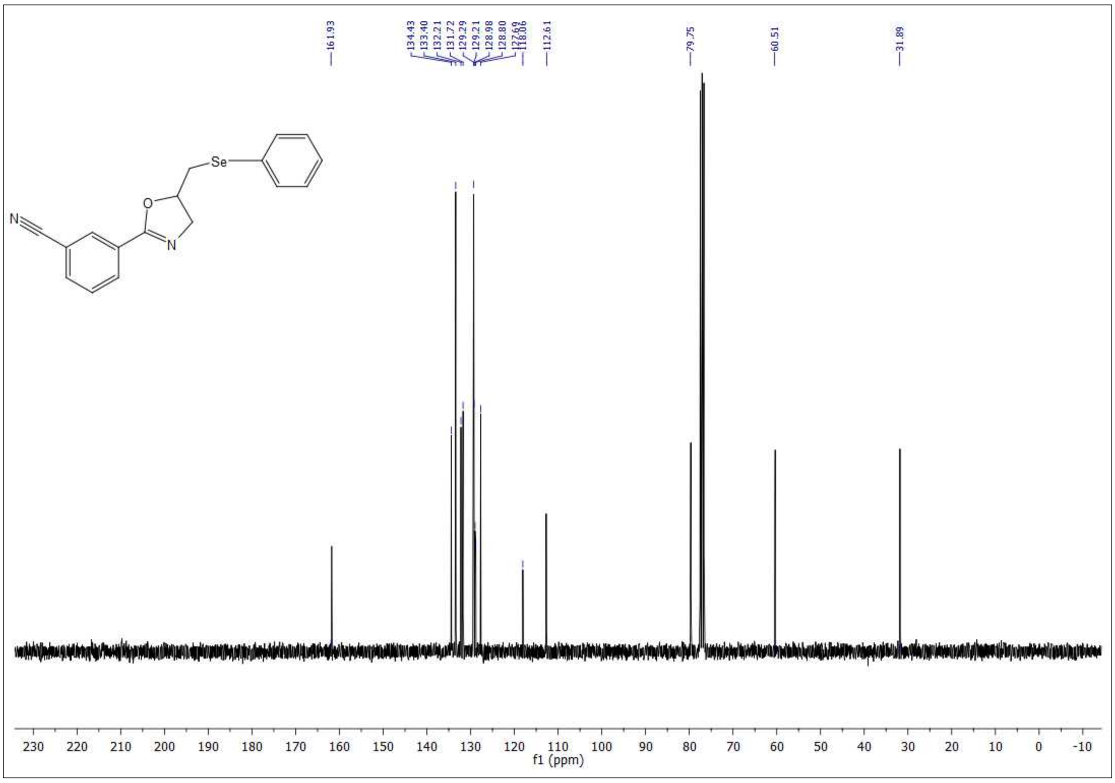

^{1}H NMR (300 MHz, $CDCl_3$) of 2-(3-nitrophenyl)-5-((phenylselanyl)methyl)-4,5-dihydrooxazole (6k)

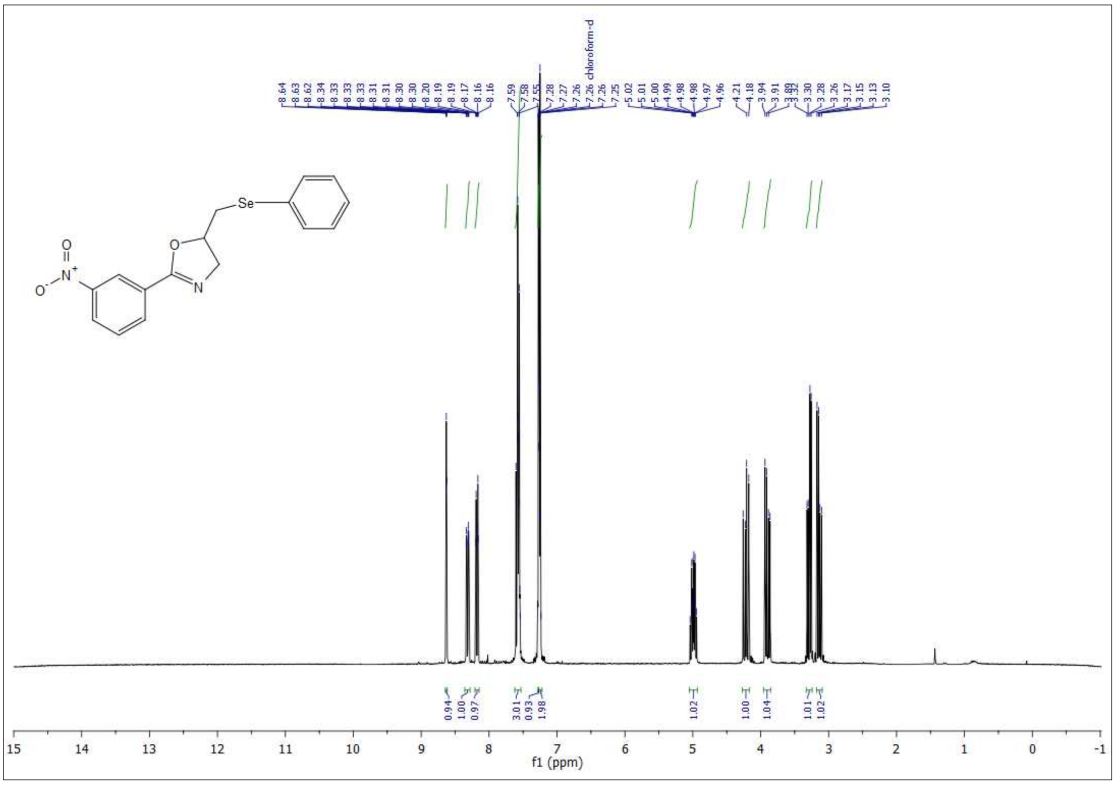

^{13}C {^{1}H} NMR (75 MHz, $CDCl_3$) of 2-(3-nitrophenyl)-5-((phenylselanyl)methyl)-4,5-dihydrooxazole (6k)

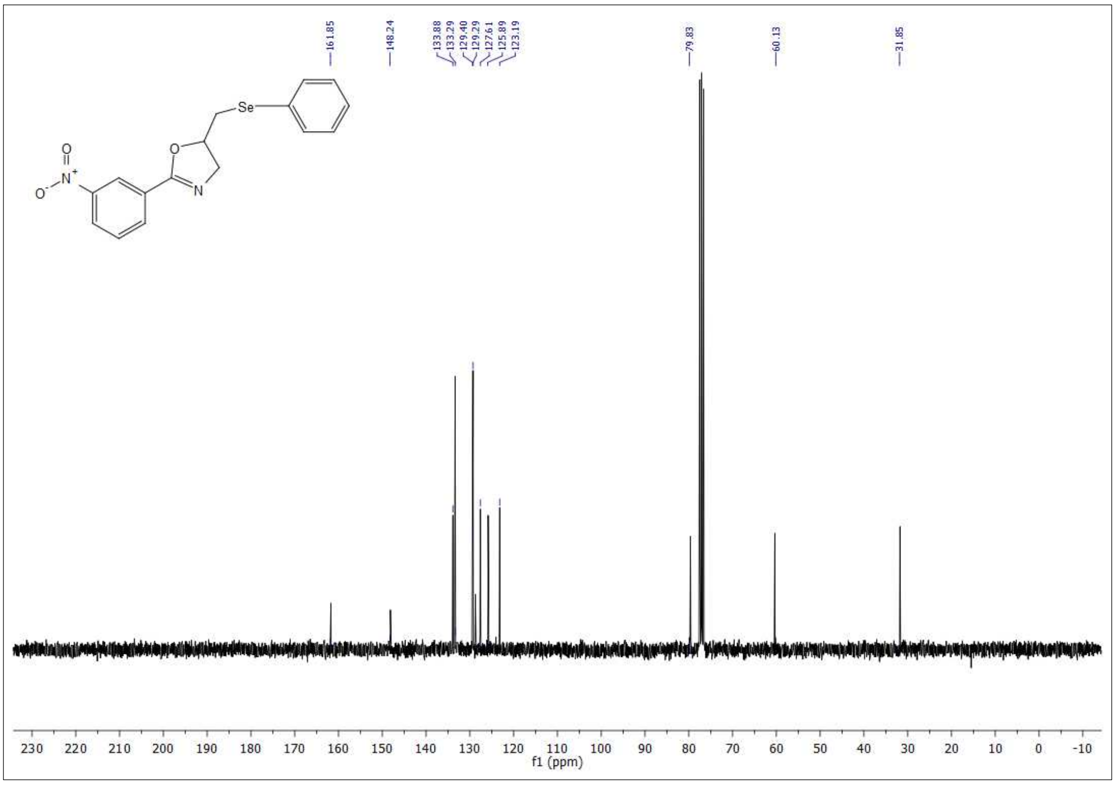

^{1}H NMR (300 MHz, $CDCl_3$) of 2-(6-methyl-5-(trifluoromethyl)phenyl)-5-((phenylselanyl)methyl)-4,5-dihydrooxazole (6l)

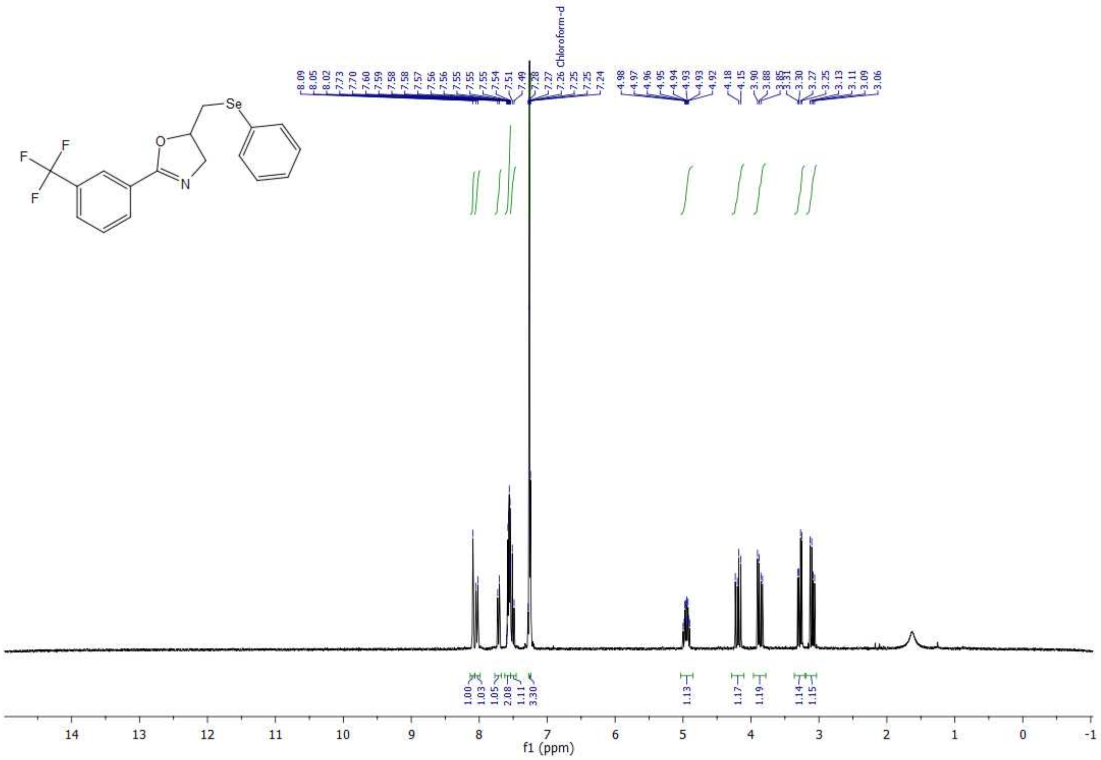

^{13}C {^{1}H} NMR (75 MHz, $CDCl_3$) of 2-(2-methyl-5-(trifluoromethyl)phenyl)-5-((phenylselanyl)methyl)-4,5-dihydrooxazole (6l)

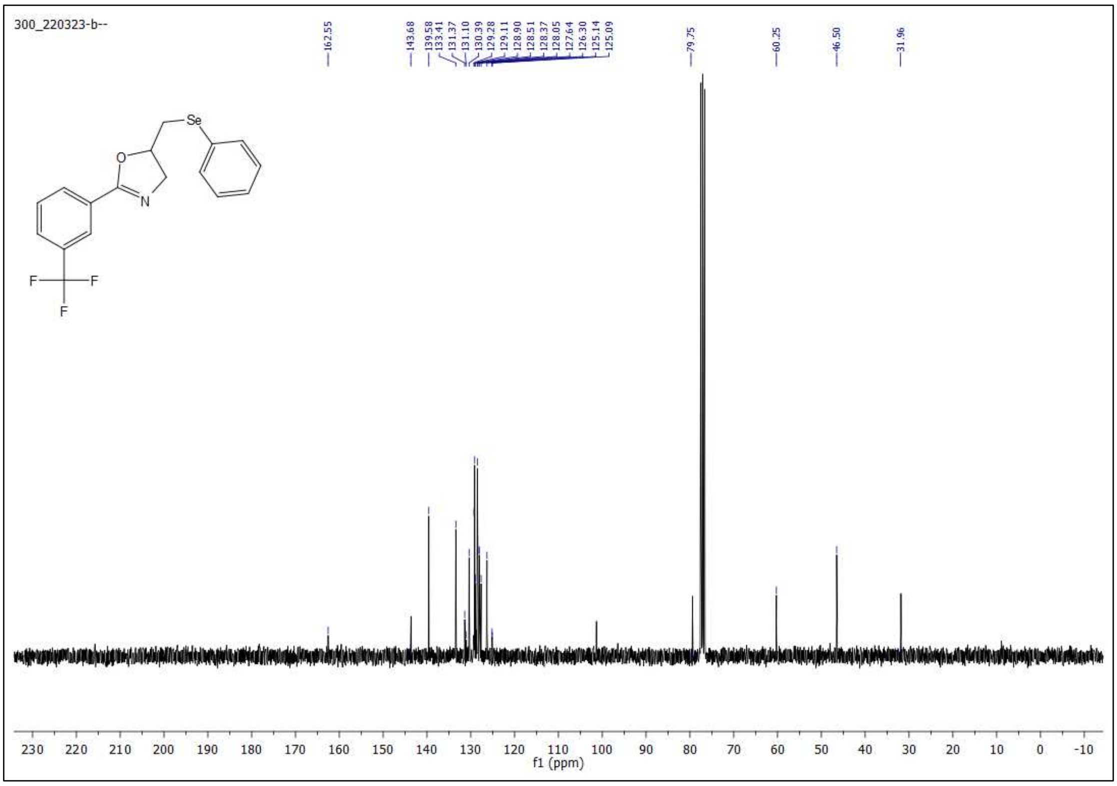

^{1}H NMR (300 MHz, $CDCl_3$) of 5-((phenylselanyl)methyl)-2-(o-tolyl)-4,5-dihydrooxazole (6m)

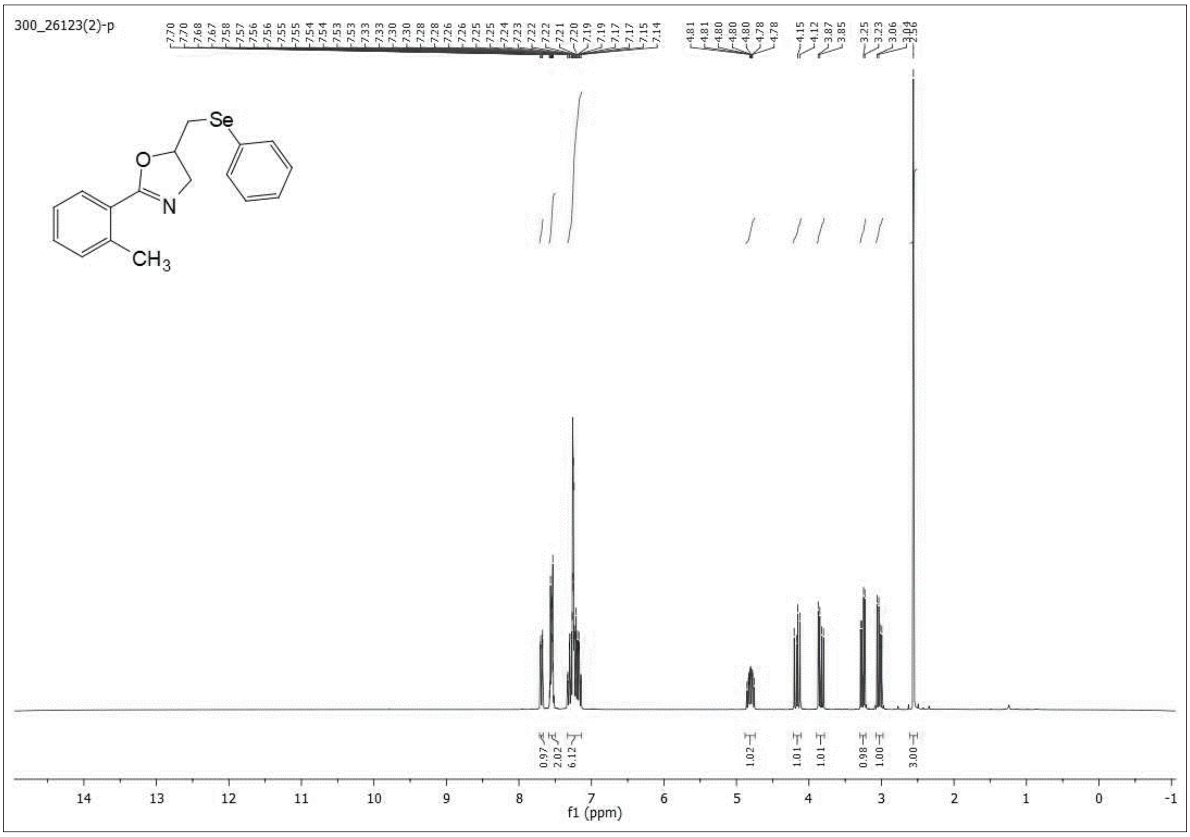

^{13}C {^{1}H} NMR (75 MHz, $CDCl_3$) of 5-((phenylselanyl)methyl)-2-(o-tolyl)-4,5-dihydrooxazole (6m)

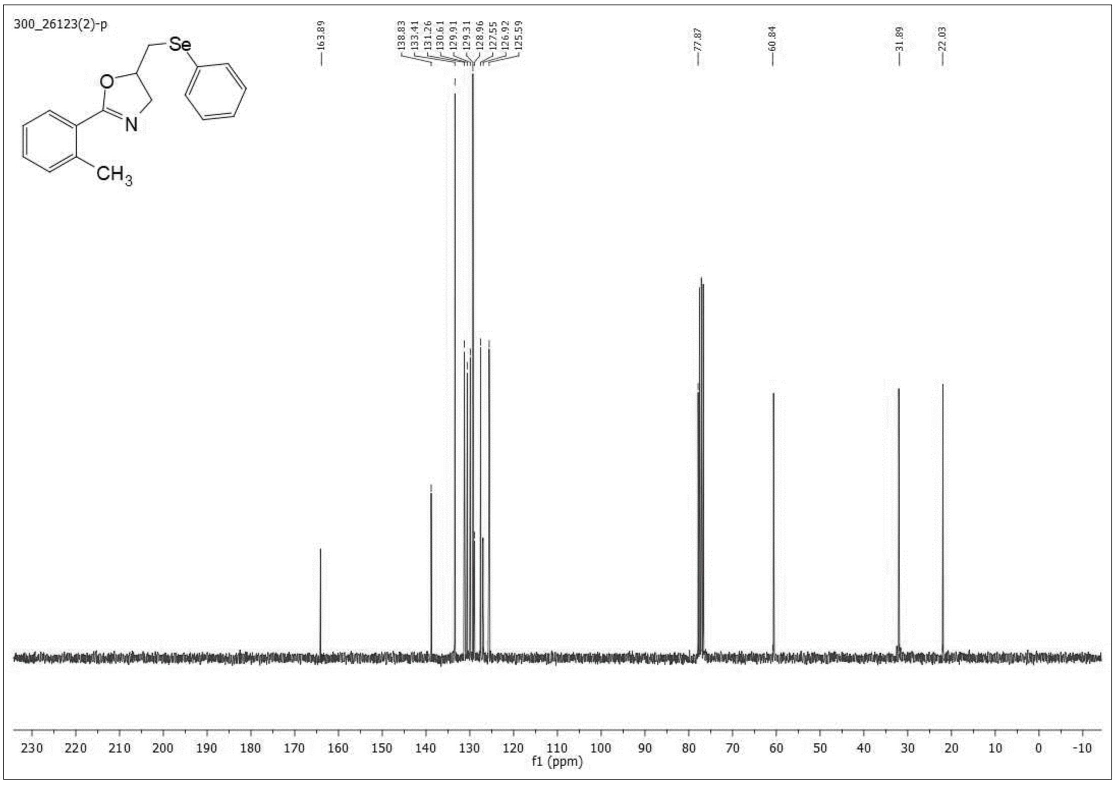

^{1}H NMR (300 MHz, $CDCl_3$) of 2-(6-bromophenyl)-5-((phenylselanyl)methyl)-4,5-dihydrooxazole (6n)

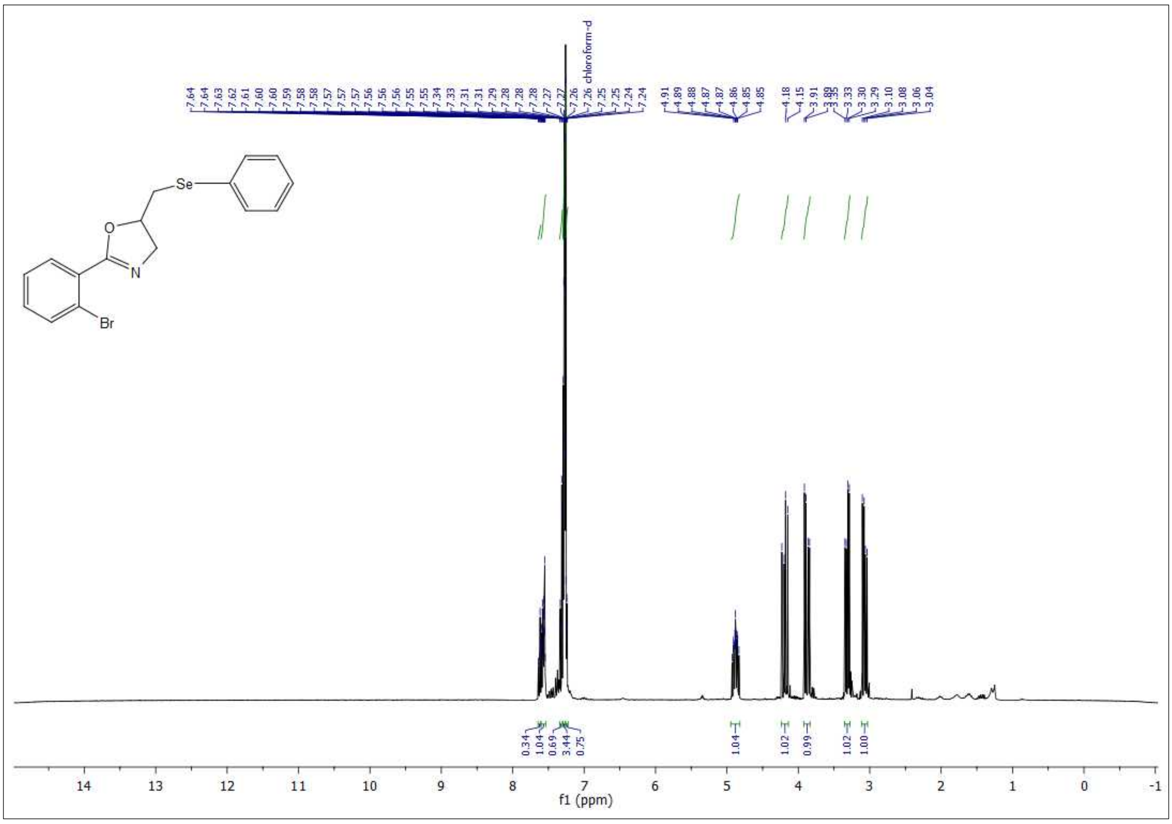

^{13}C {^{1}H} NMR (75 MHz, $CDCl_3$) of 2-(2-bromophenyl)-5-((phenylselanyl)methyl)-4,5-dihydrooxazole (6n)

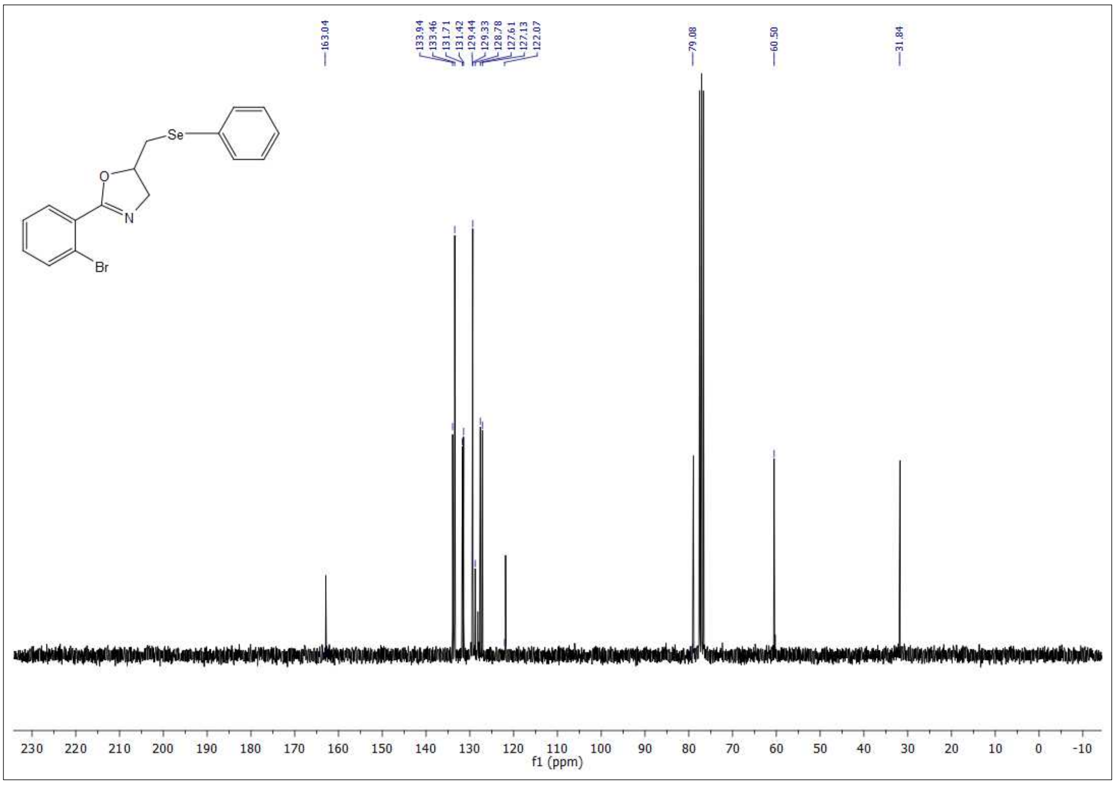

^{1}H NMR (300 MHz, $CDCl_3$) of 2-(2-chlorophenyl)-5-((phenylselanyl)methyl)-4,5-dihydrooxazole (6o)

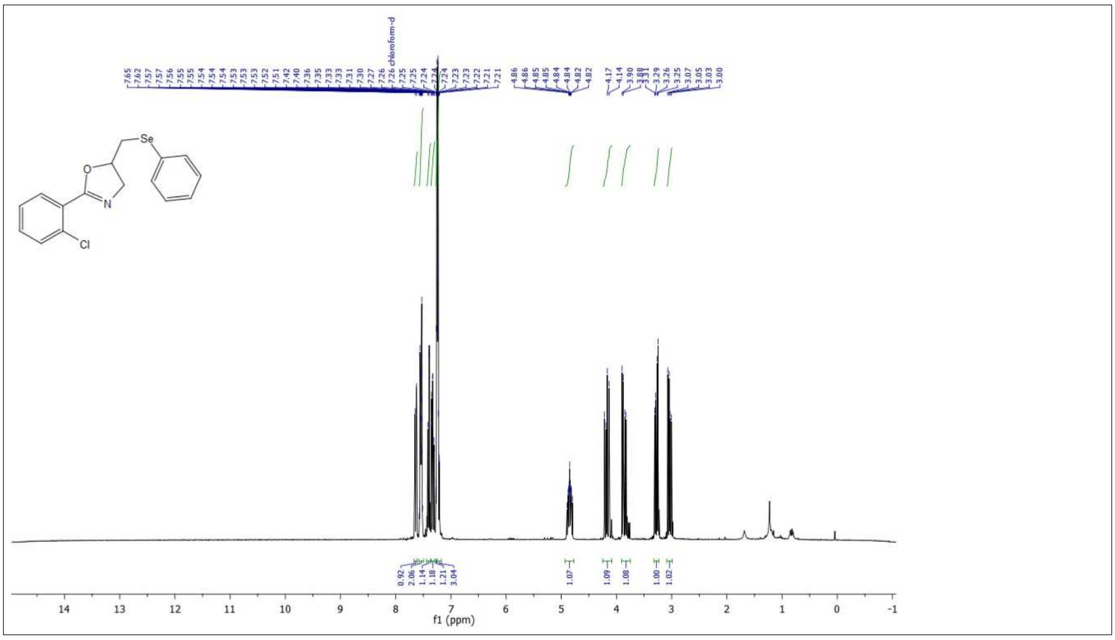

^{13}C {^{1}H} NMR (75 MHz, $CDCl_3$) of 2-(2-chlorophenyl)-5-((phenylselanyl)methyl)-4,5-dihydrooxazole (6o)

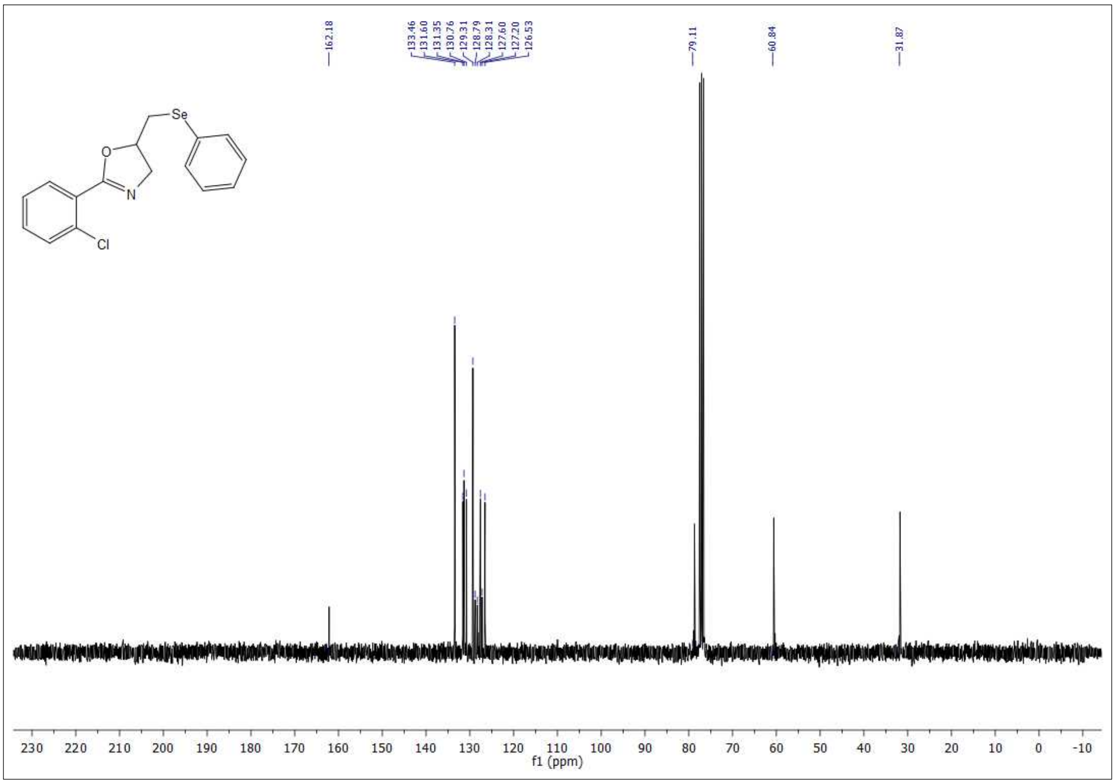

^{1}H NMR (300 MHz, $CDCl_3$) of 2-(2-iodophenyl)-5-((phenylselanyl)methyl)-4,5-dihydrooxazole (6p)

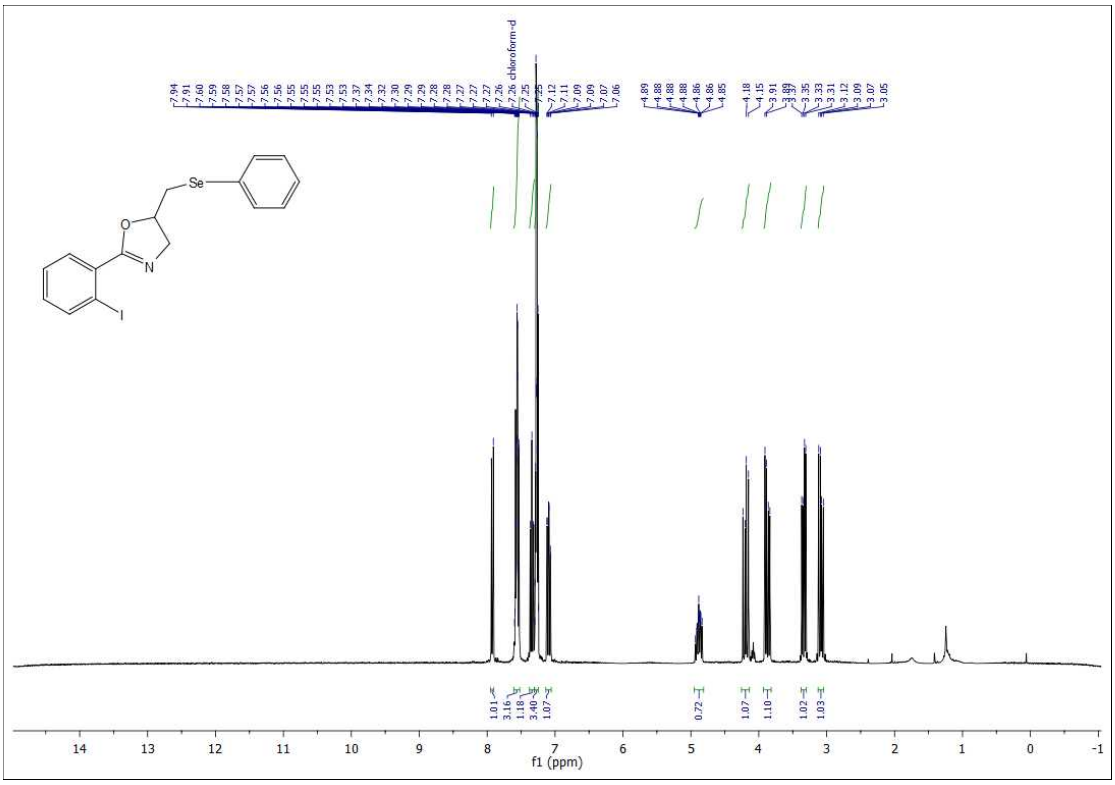

^{13}C {^{1}H} NMR (75 MHz, $CDCl_3$) of 2-(2-iodophenyl)-5-((phenylselanyl)methyl)-4,5-dihydrooxazole (6p)

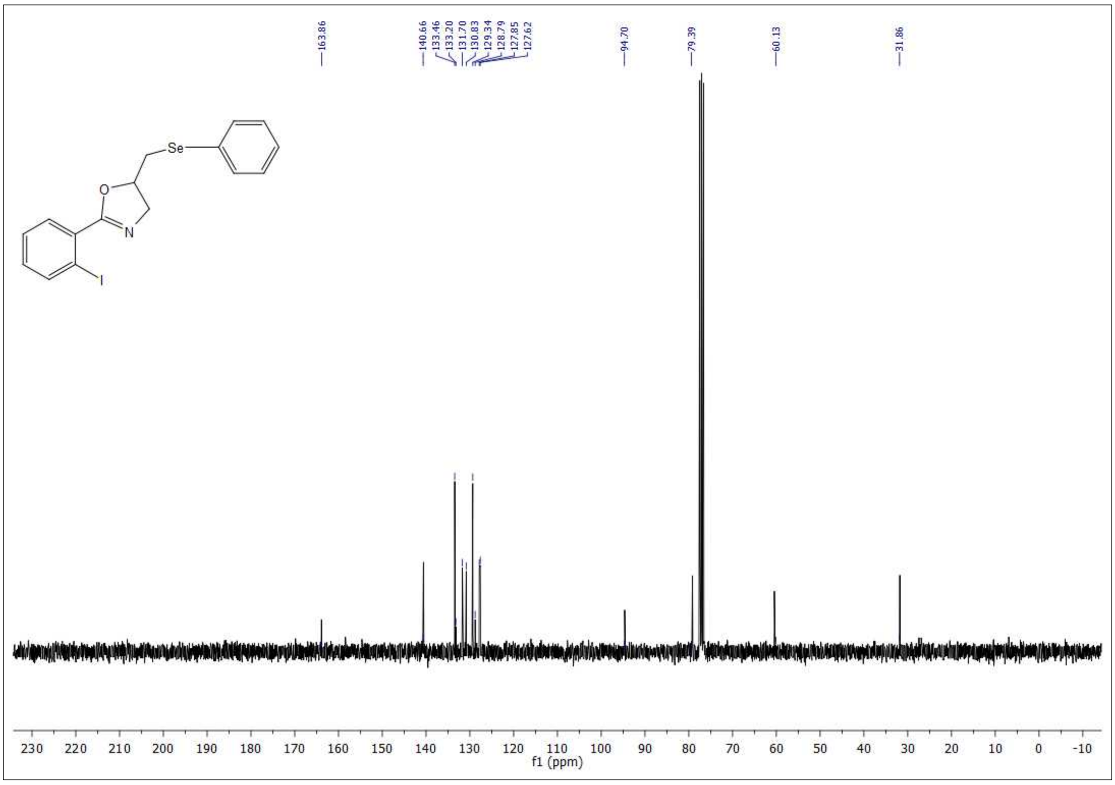

^{1}H NMR (300 MHz, $CDCl_3$) of 2-([1,1'-biphenyl]-2-yl)-5-((phenylselanyl)methyl)-4,5-dihydrooxazole (6q)

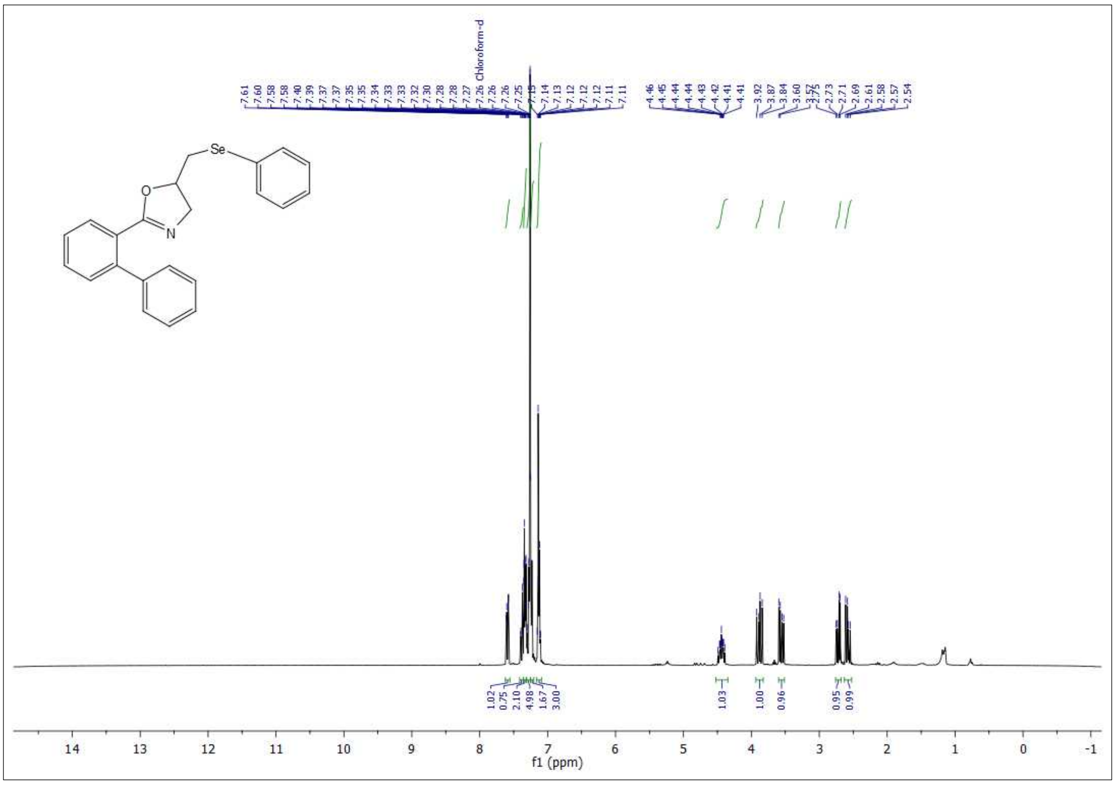

^{13}C {^{1}H} NMR (75 MHz, $CDCl_3$) of 2-([1,1'-biphenyl]-2-yl)-5-((phenylselanyl)methyl)-4,5-dihydrooxazole (6q)

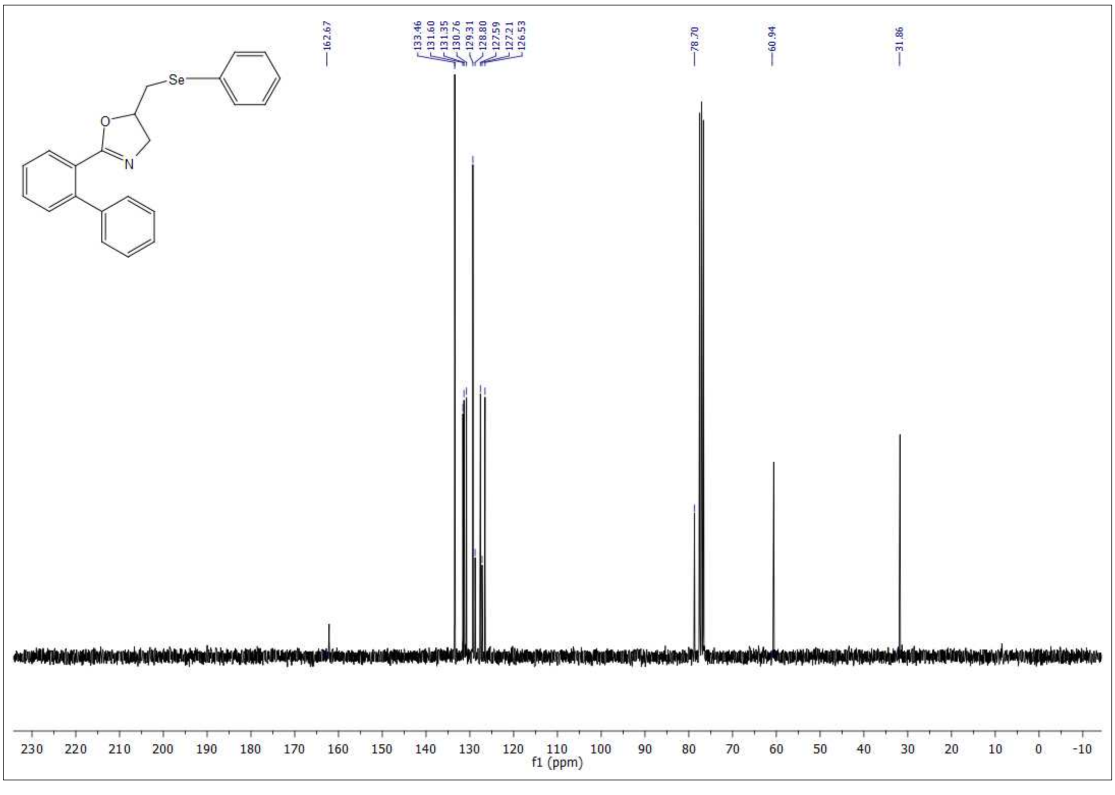

^{1}H NMR (300 MHz, $CDCl_3$) of 2-(furan-2-yl)-5-((phenylselanyl)methyl)-4,5-dihydrooxazole (6r)

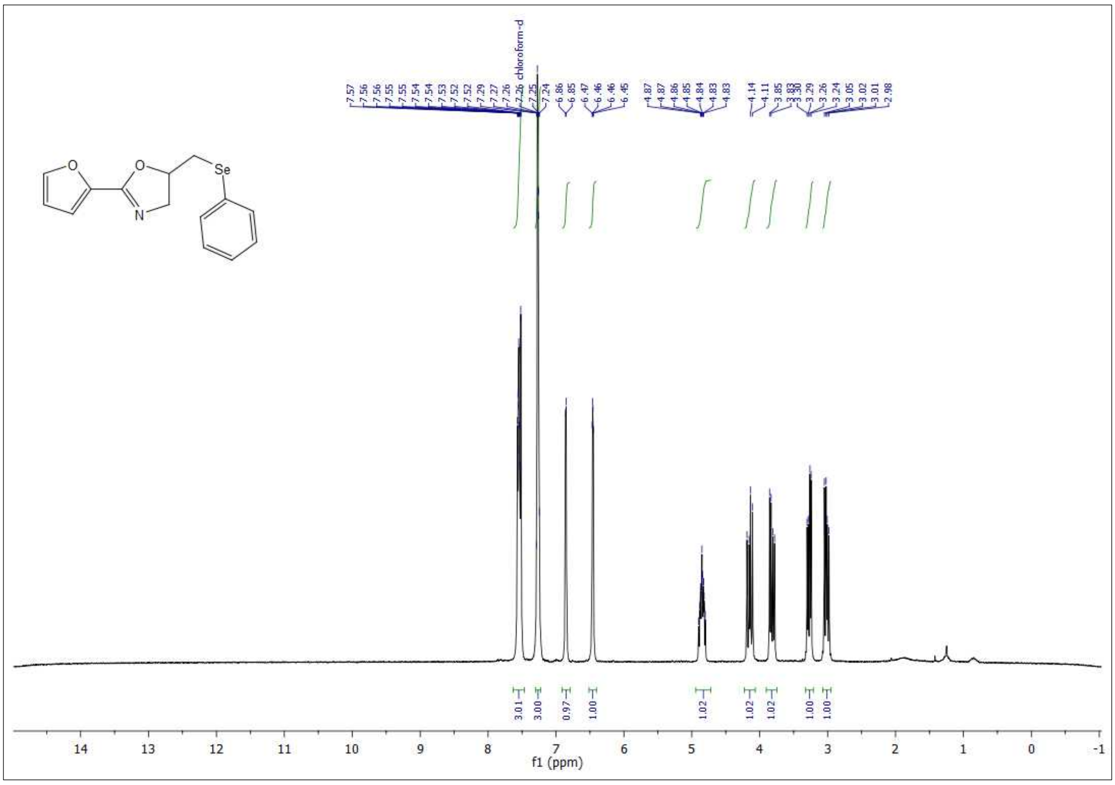

^{13}C {^{1}H} NMR (75 MHz, $CDCl_3$)of 2-(furan-2-yl)-5-((phenylselanyl)methyl)-4,5-dihydrooxazole (6r)

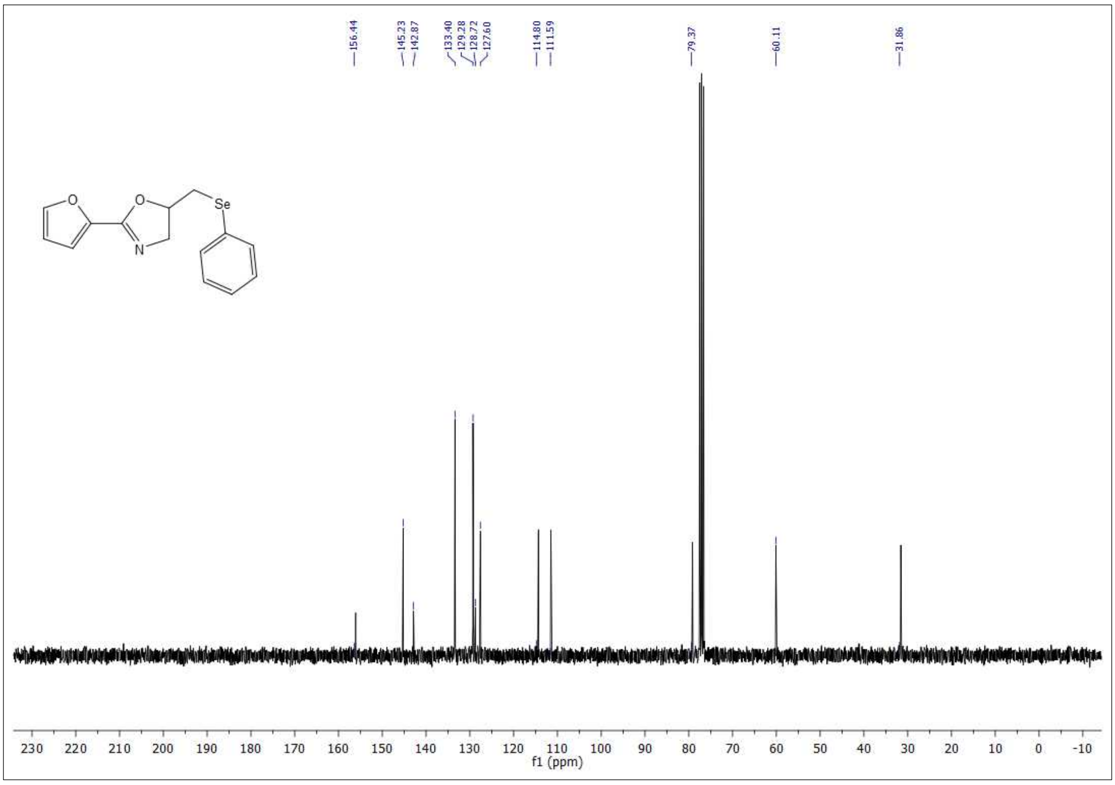

^{1}H NMR (300 MHz, $CDCl_3$) of 5-((phenylselanyl)methyl)-2-(thiophen-2-yl)-4,5-dihydrooxazole (6s)

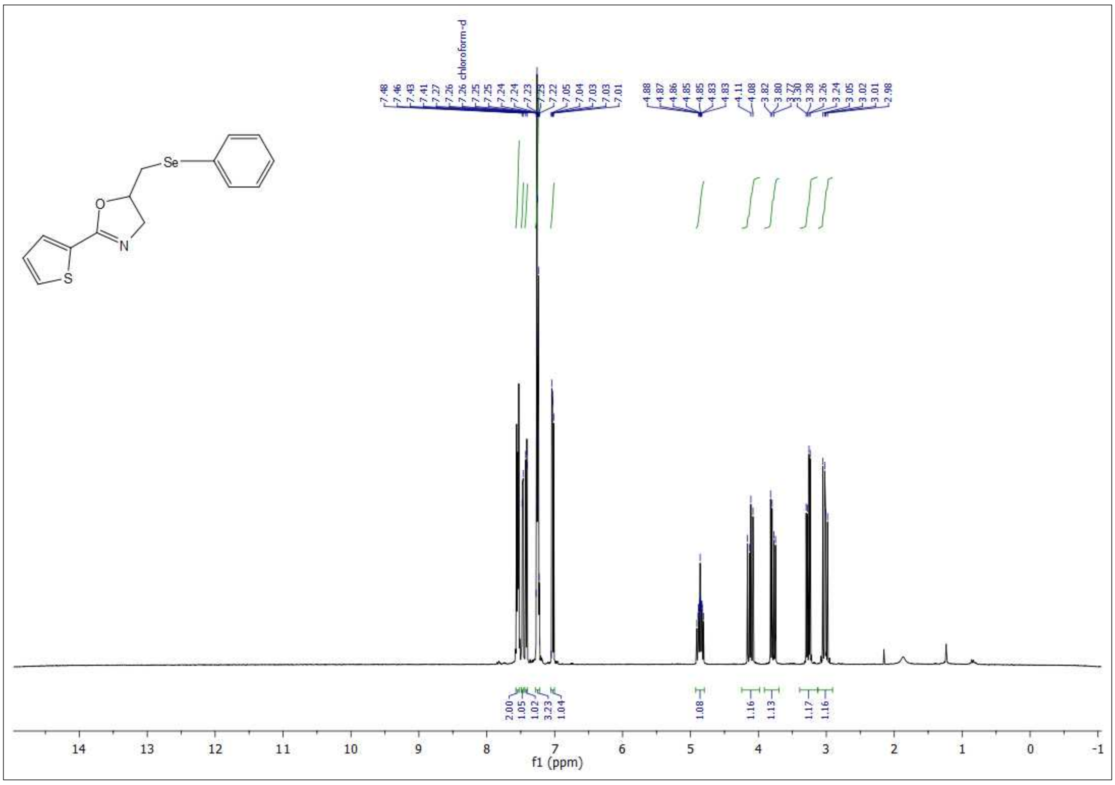

^{13}C {^{1}H} NMR (75 MHz, $CDCl_3$) of 5-((phenylselanyl)methyl)-2-(thiophen-2-yl)-4,5-dihydrooxazole (6s)

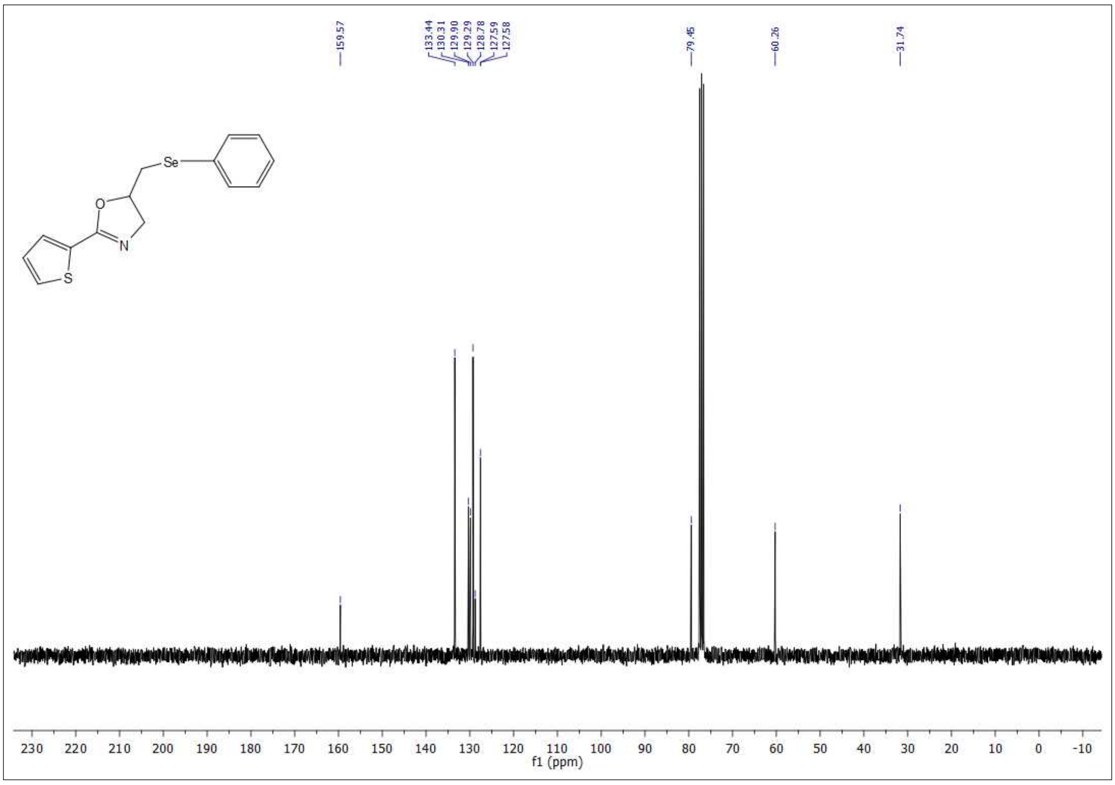

^{1}H NMR (300 MHz, $CDCl_3$) of 5-((phenylselanyl)methyl)-2-(pyridin-3-yl)-4,5-dihydrooxazole (6t)

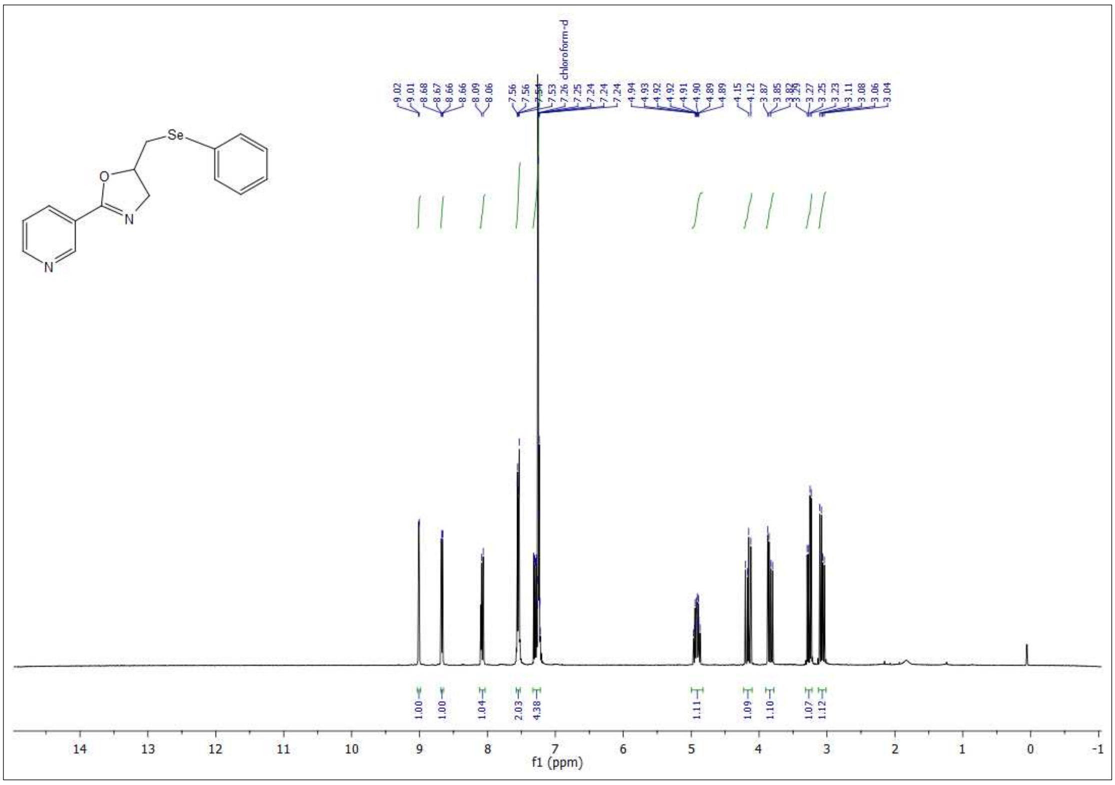

^{13}C {^{1}H} NMR (75 MHz, $CDCl_3$) of 5-((phenylselanyl)methyl)-2-(pyridin-3-yl)-4,5-dihydrooxazole (6t)

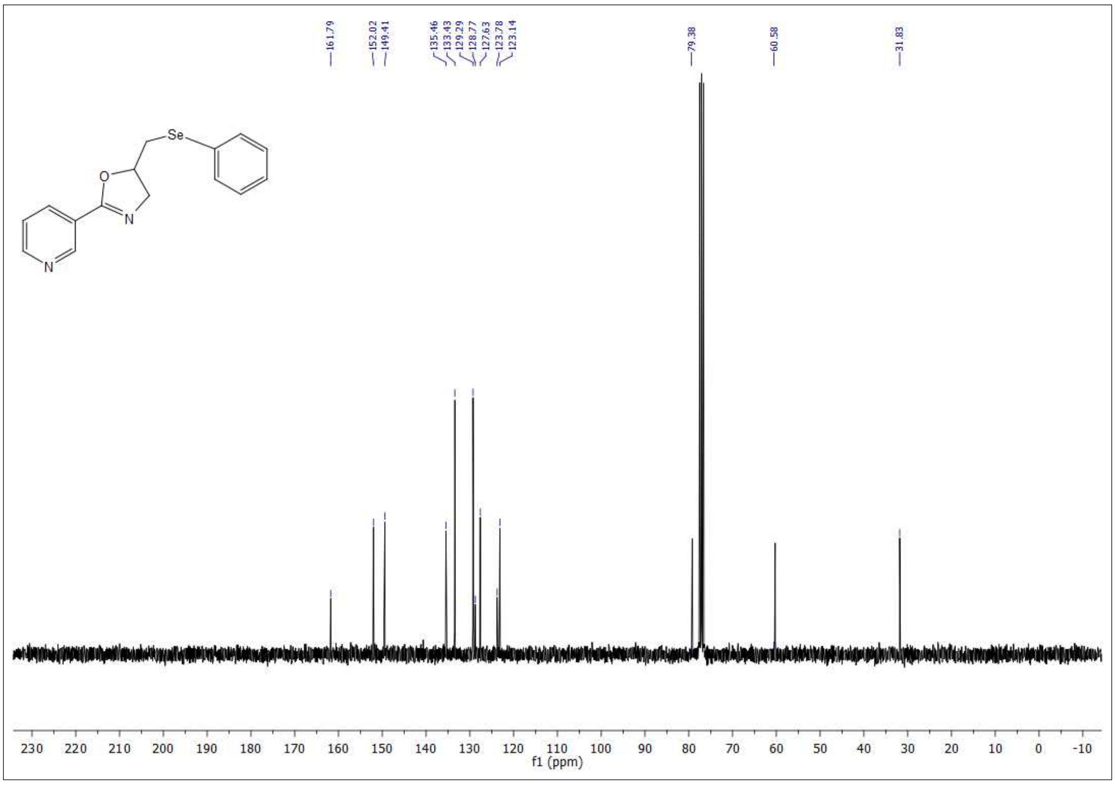

^{1}H NMR (300 MHz, $CDCl_3$) of 2-phenyl-6-((phenylselanyl)methyl)-5,6-dihydro-4H-1,3-oxazine (6u)

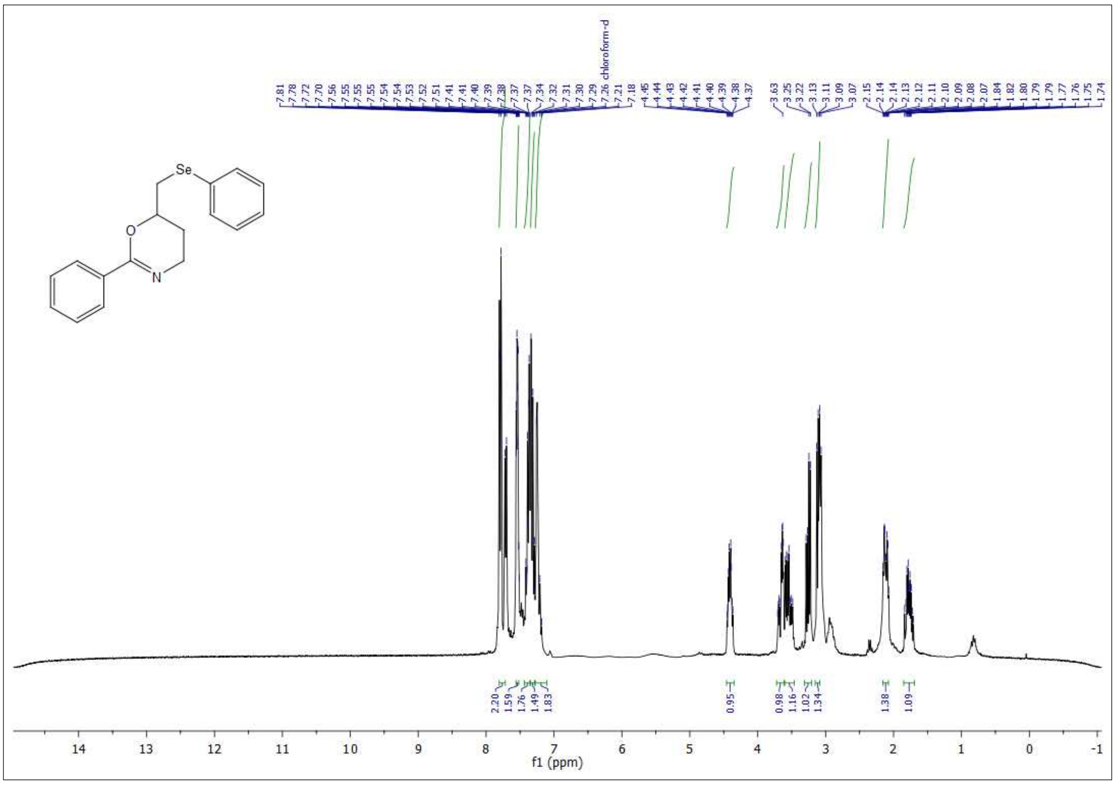

^{13}C {^{1}H} NMR (75 MHz, $CDCl_3$) of 2-phenyl-6-((phenylselanyl)methyl)-5,6-dihydro-4H-1,3-oxazine (6u)

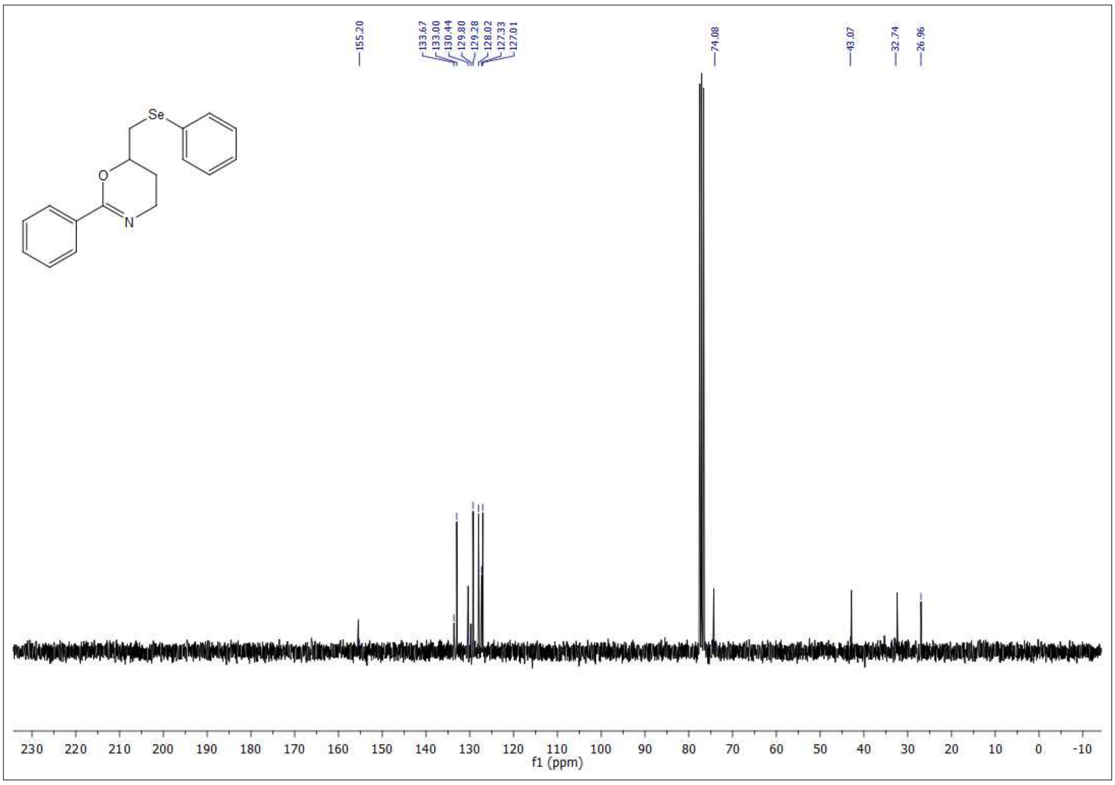

^{1}H NMR (300 MHz, CDCl$_3$) of 2-(4-chlorophenyl)-6-((phenylselanyl)methyl)-5,6-dihydro-4*H*-1,3-oxazine (6v)

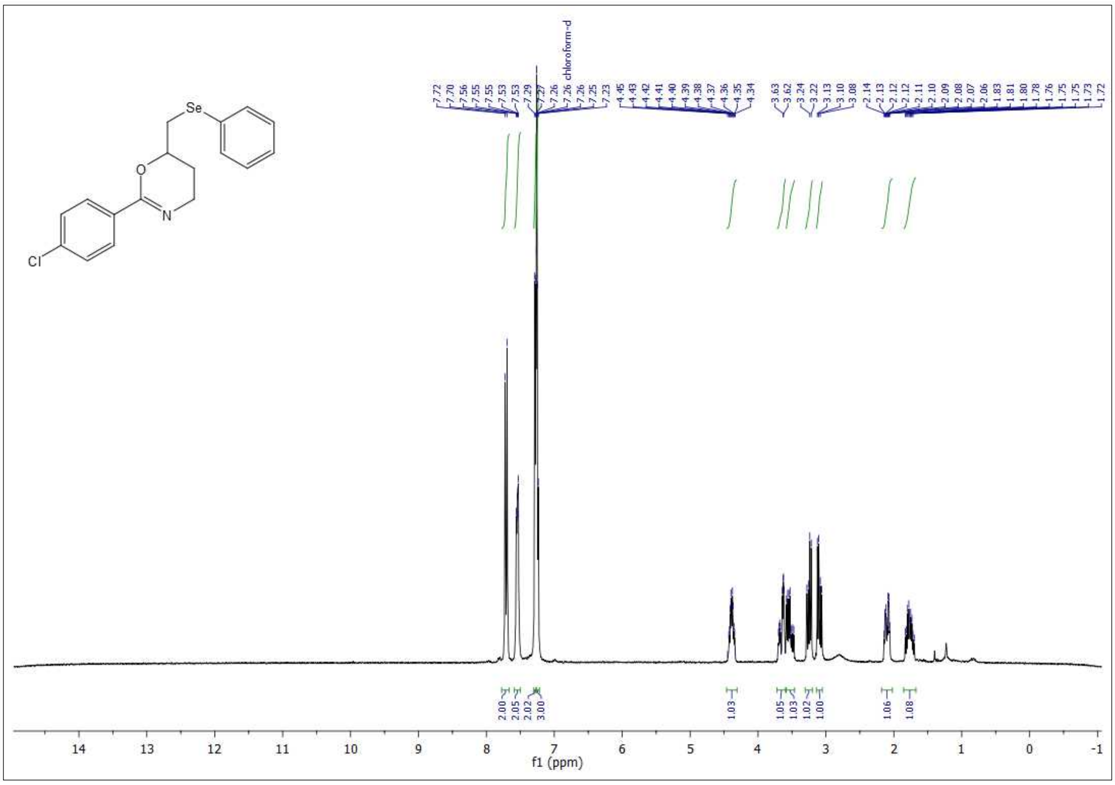

^{13}C {^{1}H} NMR (75 MHz, CDCl$_3$) of 2-(4-chlorophenyl)-6-((phenylselanyl)methyl)-5,6-dihydro-4H-1,3-oxazine (6v)

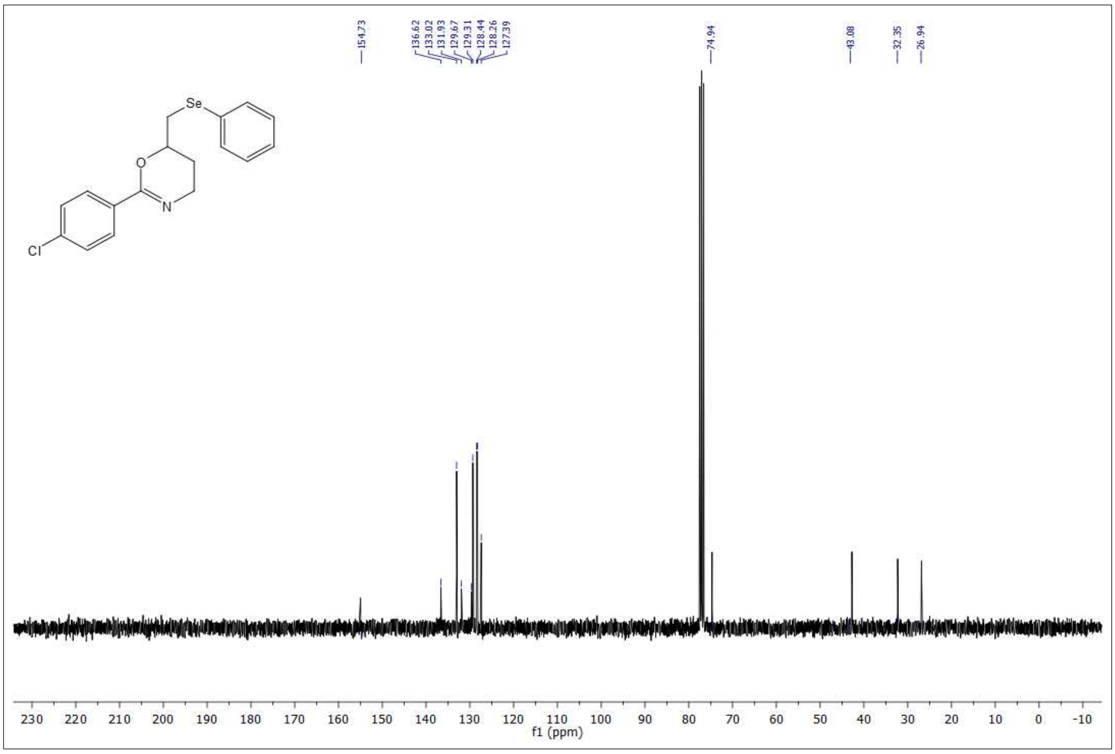

^{1}H NMR (300 MHz, $CDCl_3$) of 2-cyclohexyl-5-((phenylselanyl)methyl)-4,5-dihydrooxazole (6w)

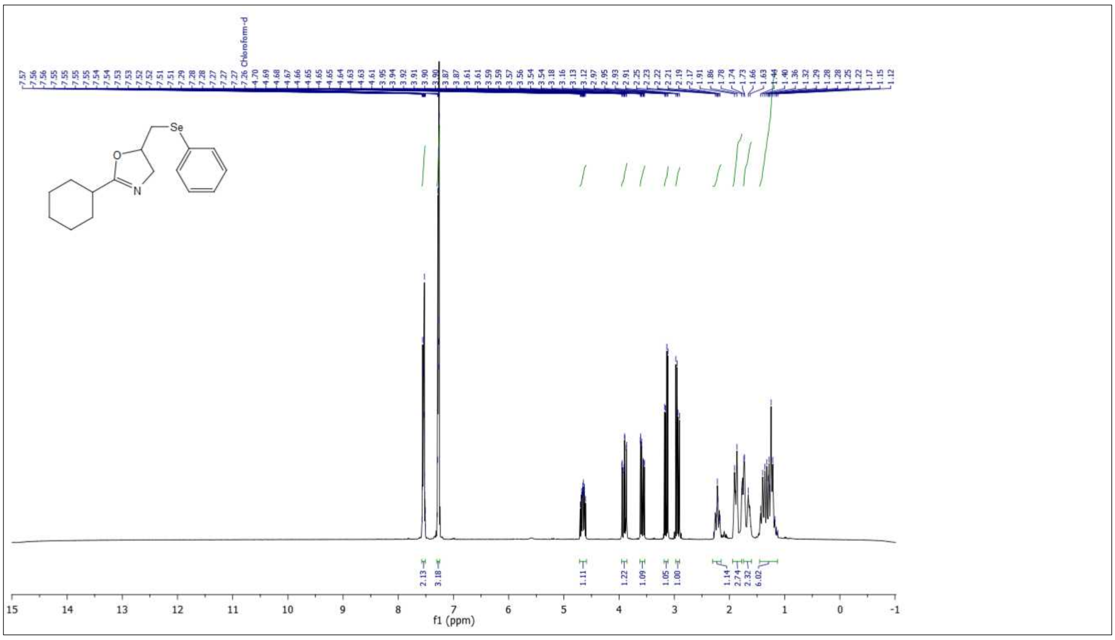

^{13}C {^{1}H} NMR (75 MHz, $CDCl_3$) of 2-cyclohexyl-5-((phenylselanyl)methyl)-4,5-dihydrooxazole (6w)

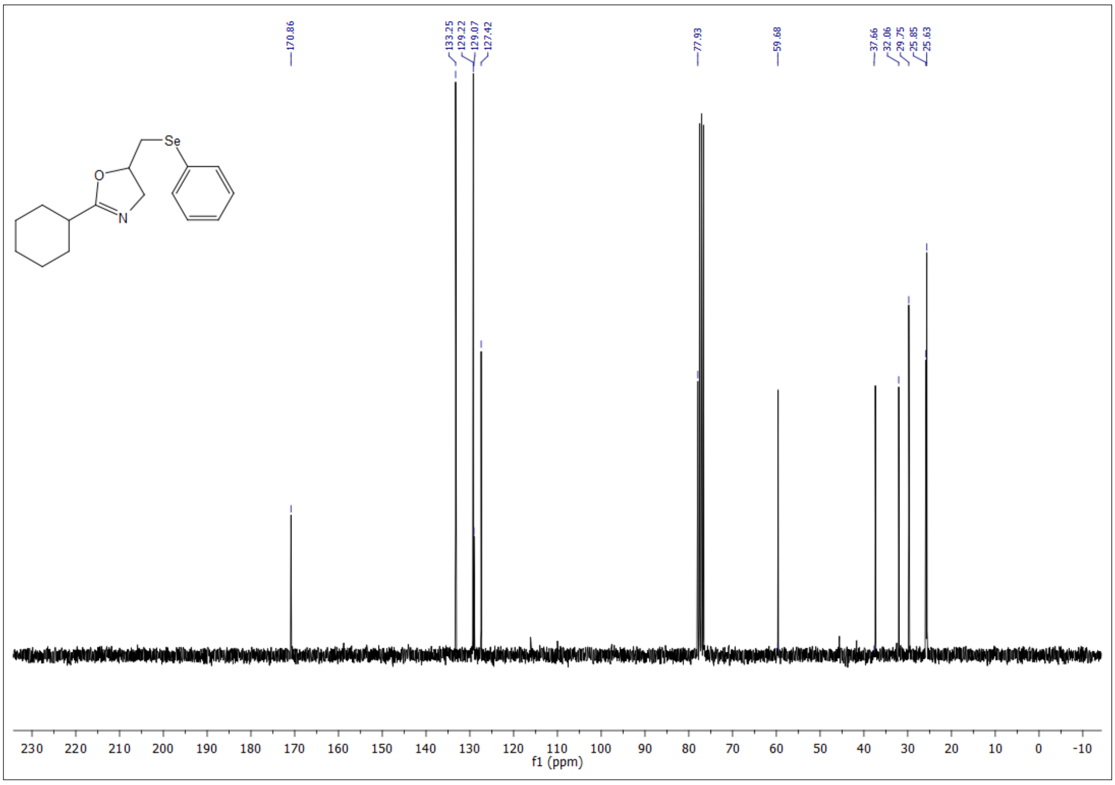

^{1}H NMR (300 MHz, $CDCl_3$) of 2-cyclobutyl-5-((phenylselanyl)methyl)-4,5-dihydrooxazole (6x)

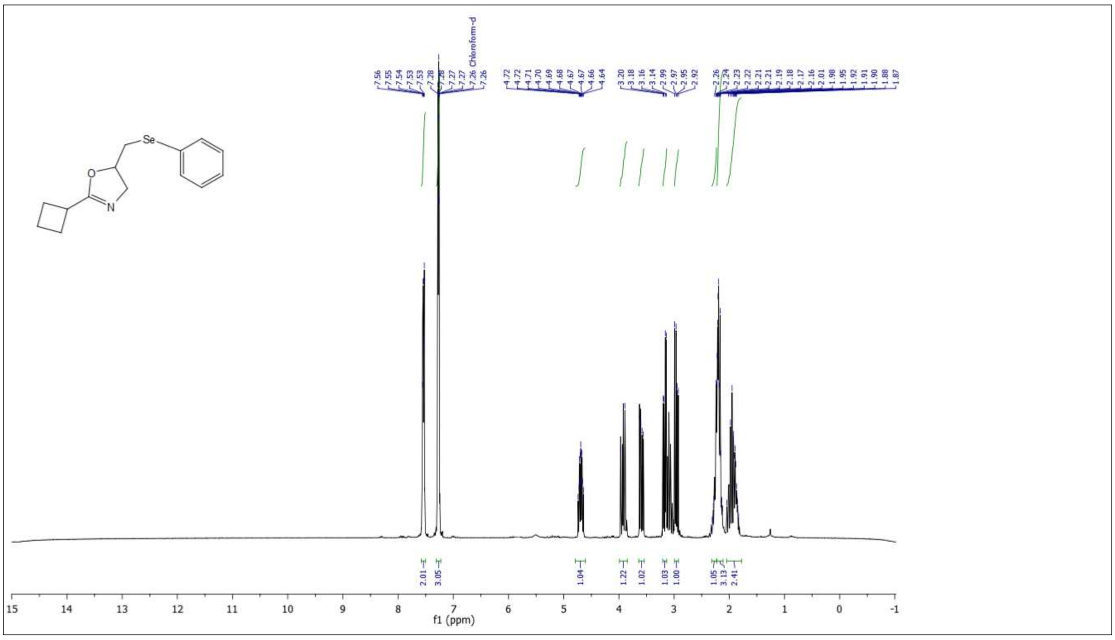

^{13}C {^{1}H} NMR (75 MHz, $CDCl_3$) of 2-cyclobutyl-5-((phenylselanyl)methyl)-4,5-dihydrooxazole (6x)

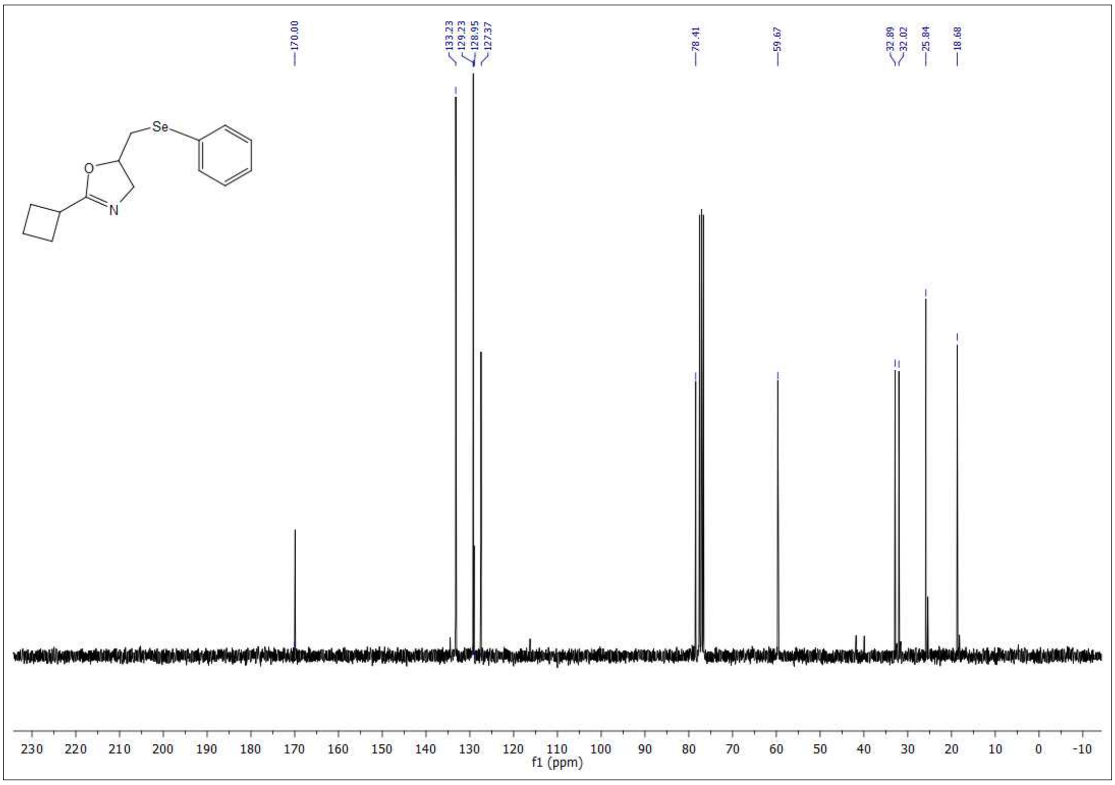

^{1}H NMR (300 MHz, $CDCl_3$) of 2-cyclopropyl-5-((phenylselanyl)methyl)-4,5-dihydrooxazole (6y)

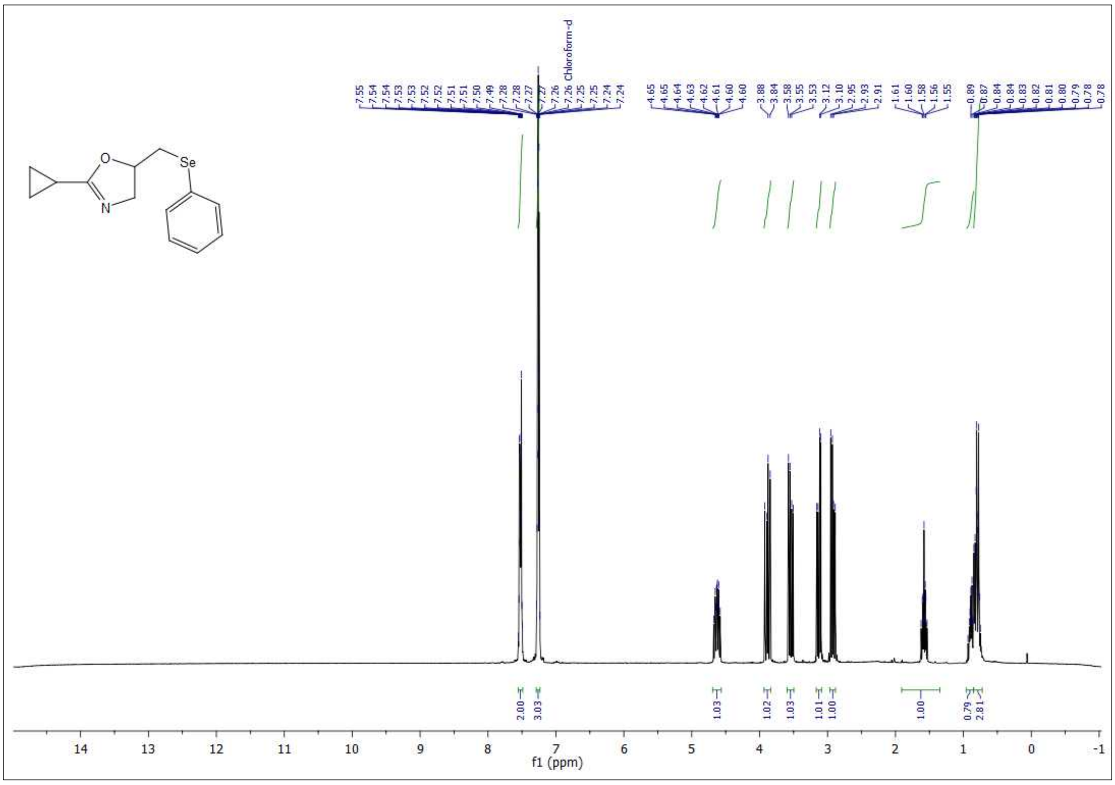

^{13}C {^{1}H} NMR (75 MHz, $CDCl_3$) of 2-cyclopropyl-5-((phenylselanyl)methyl)-4,5-dihydrooxazole (6y)

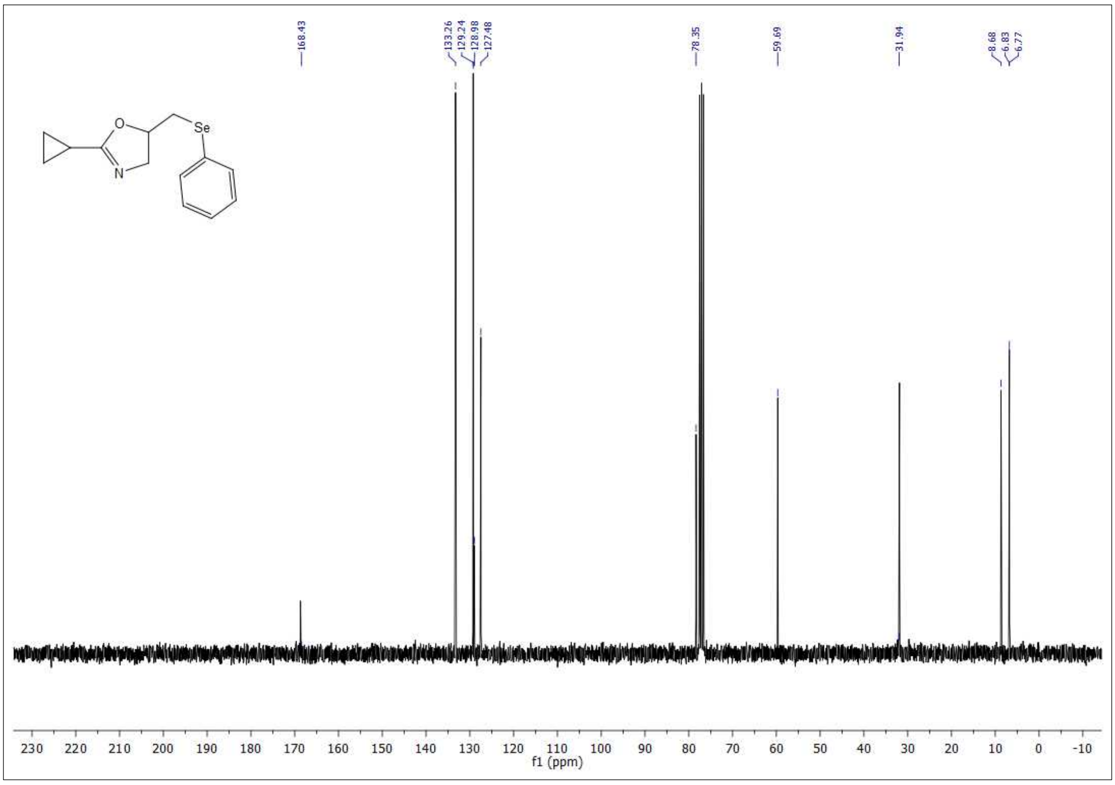

^{1}H NMR (300 MHz, $CDCl_3$) of 2-(tert-butyl)-5-((phenylselanyl)methyl)-4,5-dihydrooxazole (6z)

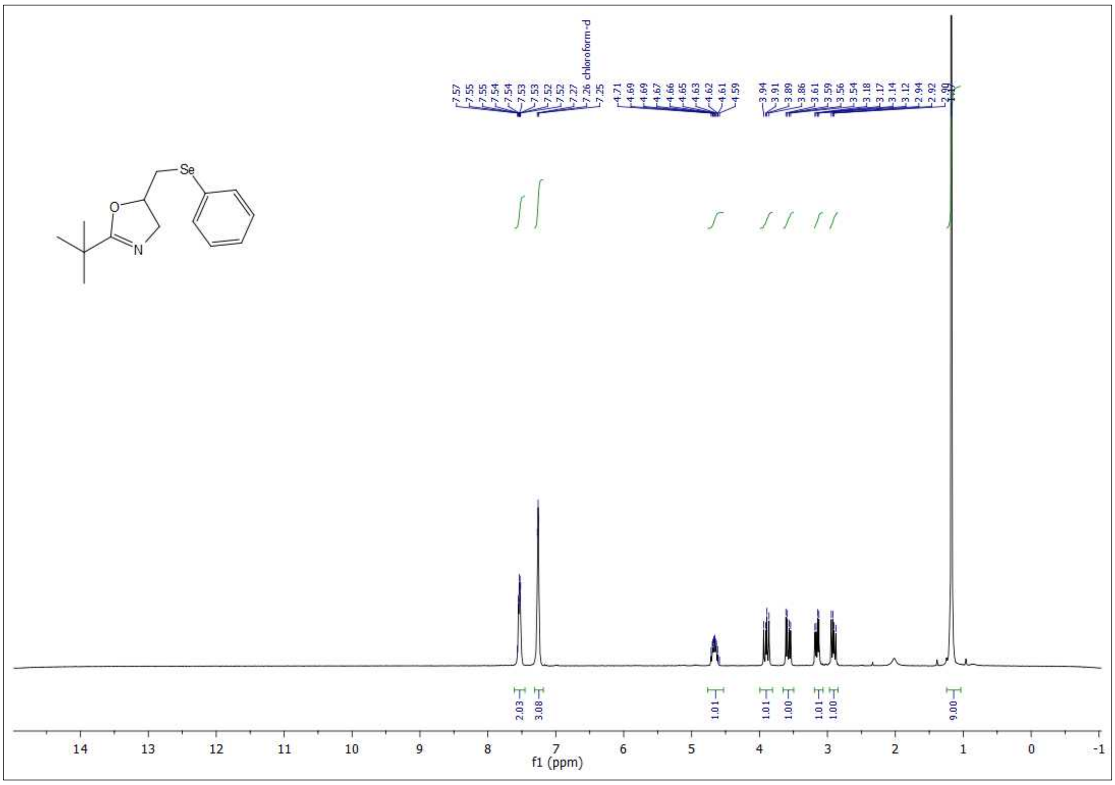

^{13}C {^{1}H} NMR (75 MHz, $CDCl_3$) of 2-(tert-butyl)-5-((phenylselanyl)methyl)-4,5-dihydrooxazole (6z)

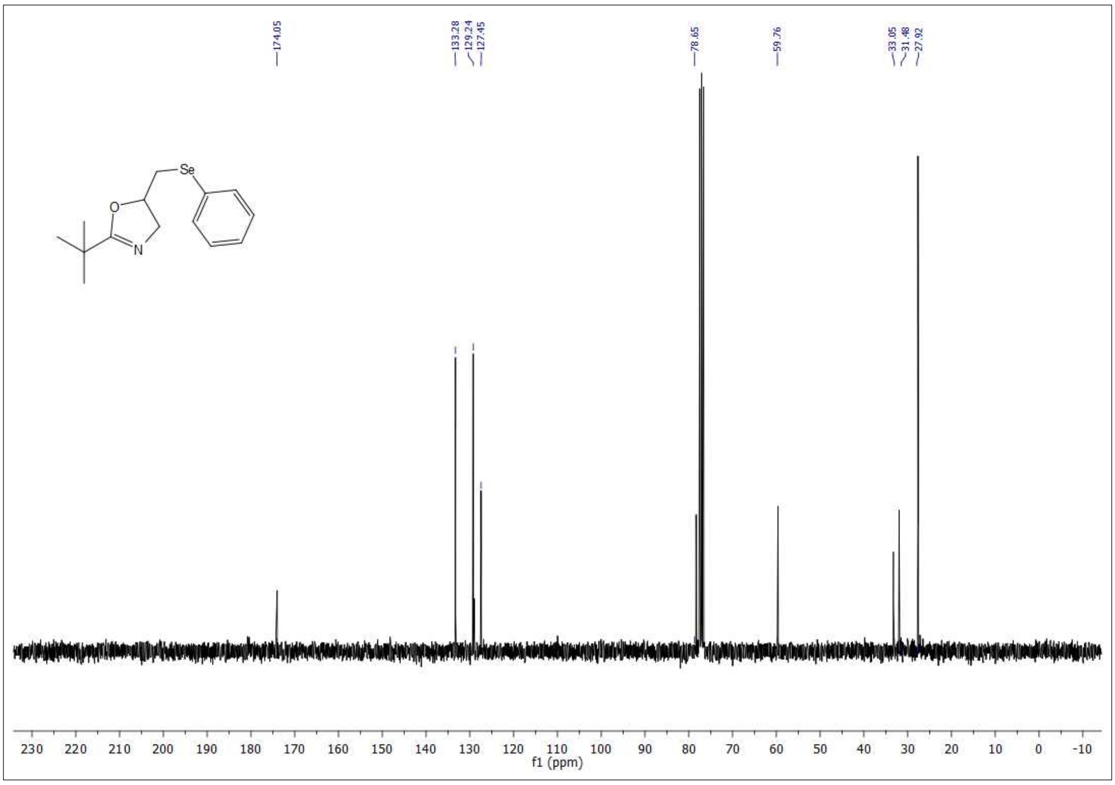

^{1}H NMR (300 MHz, $CDCl_3$) of 2-(naphthalen-2-yl)-5-((phenylselanyl)methyl)-4,5-dihydrooxazole (6aa)

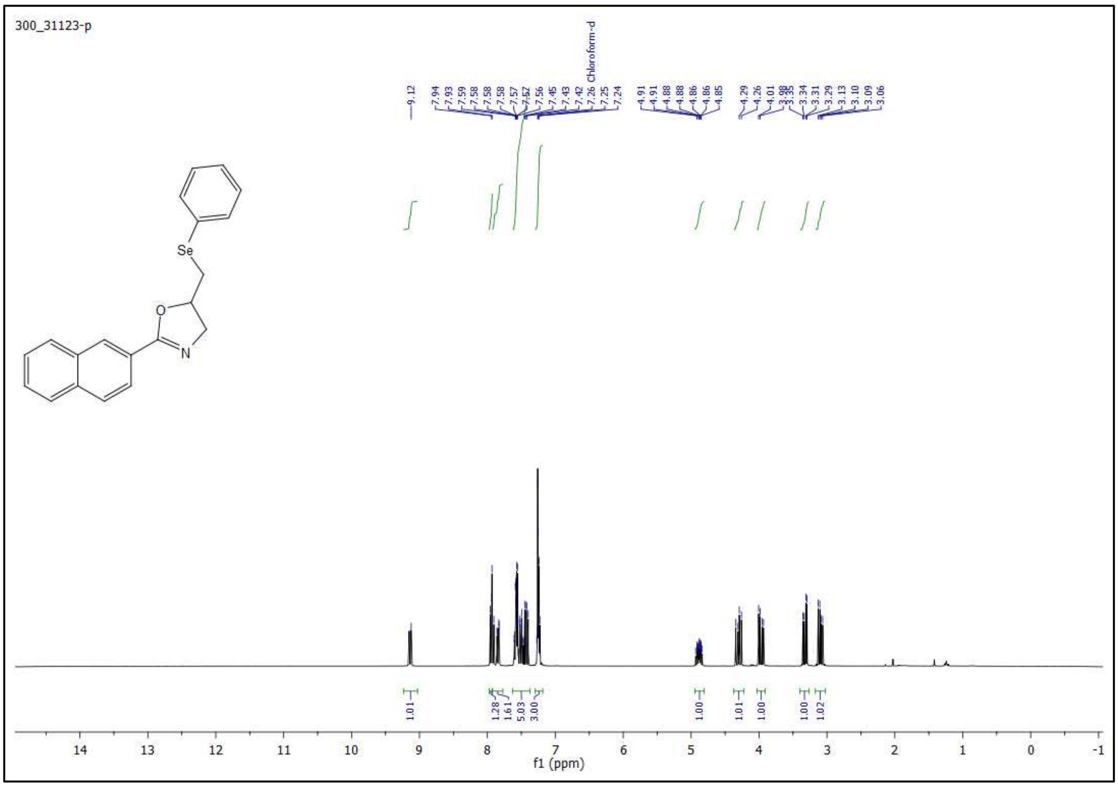

^{13}C {^{1}H} NMR (75 MHz, $CDCl_3$) of 2-(naphthalen-2-yl)-5-((phenylselanyl)methyl)-4,5-dihydrooxazole (6aa)

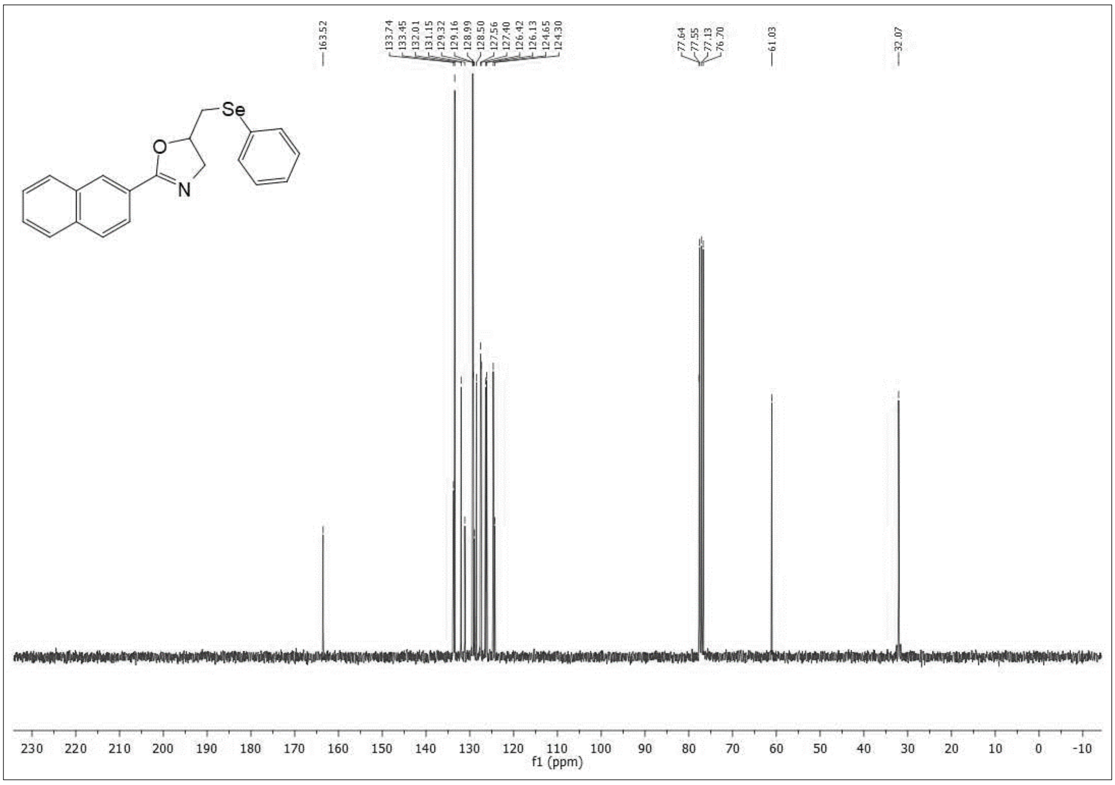

^{1}H NMR (300 MHz, $CDCl_3$) of 5-methyl-2-phenyl-5-((phenylselanyl)methyl)-4,5-dihydrooxazole (6ab)

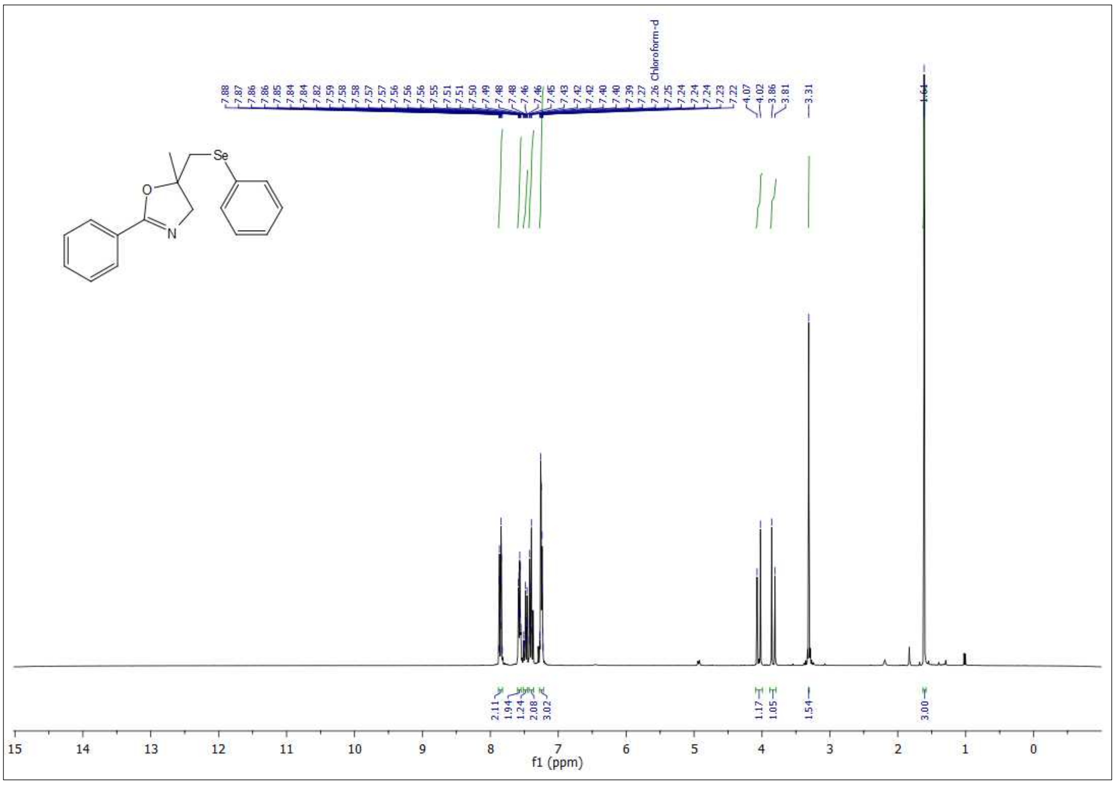

^{13}C {^{1}H} NMR (75 MHz, $CDCl_3$) of 5-methyl-2-phenyl-5-((phenylselanyl)methyl)-4,5-dihydrooxazole (6ab)

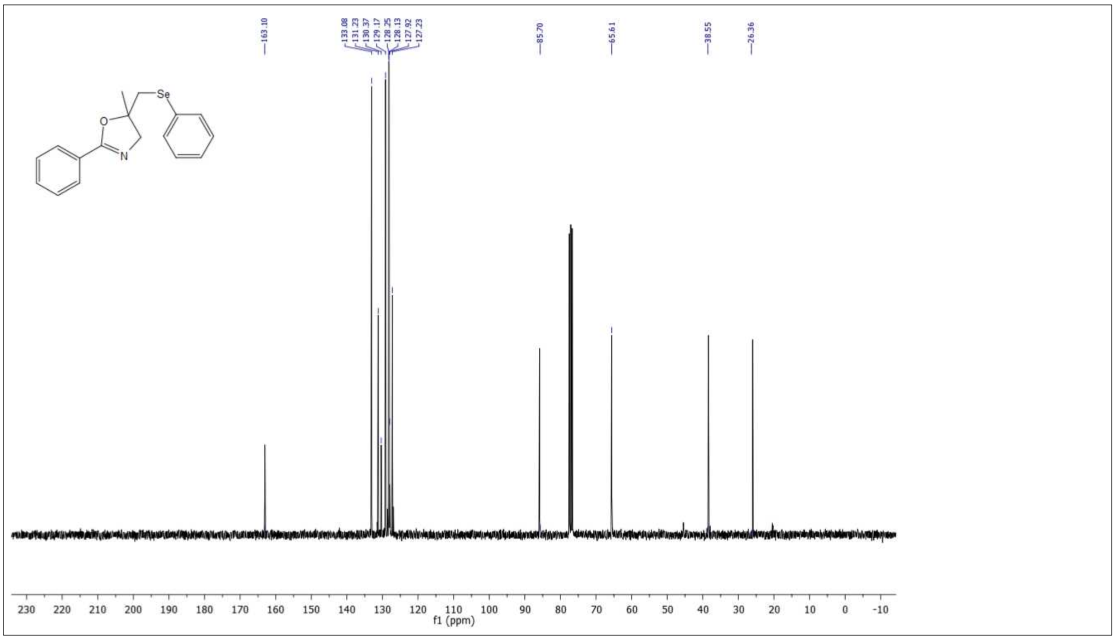

^{1}H NMR (300 MHz, $CDCl_3$) of 2-(4-chlorophenyl)-5-methyl-5-((phenylselanyl)methyl)-4,5-dihydrooxazole (6ac)

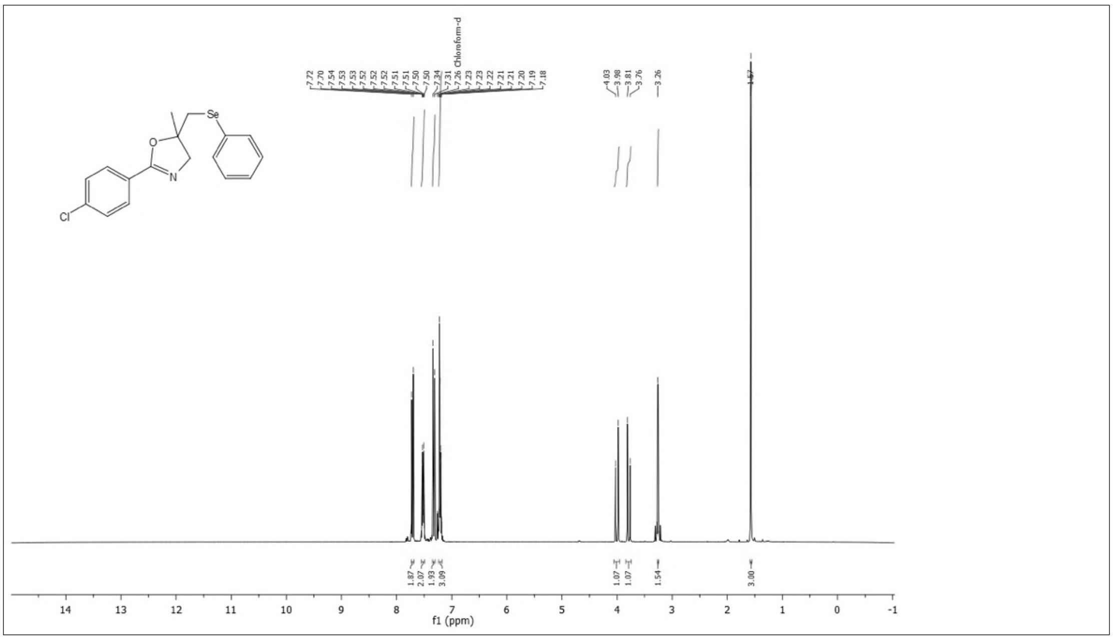

^{13}C {^{1}H} NMR (75 MHz, $CDCl_3$) of 2-(4-chlorophenyl)-5-methyl-5-((phenylselanyl)methyl)-4,5-dihydrooxazole (6ac)

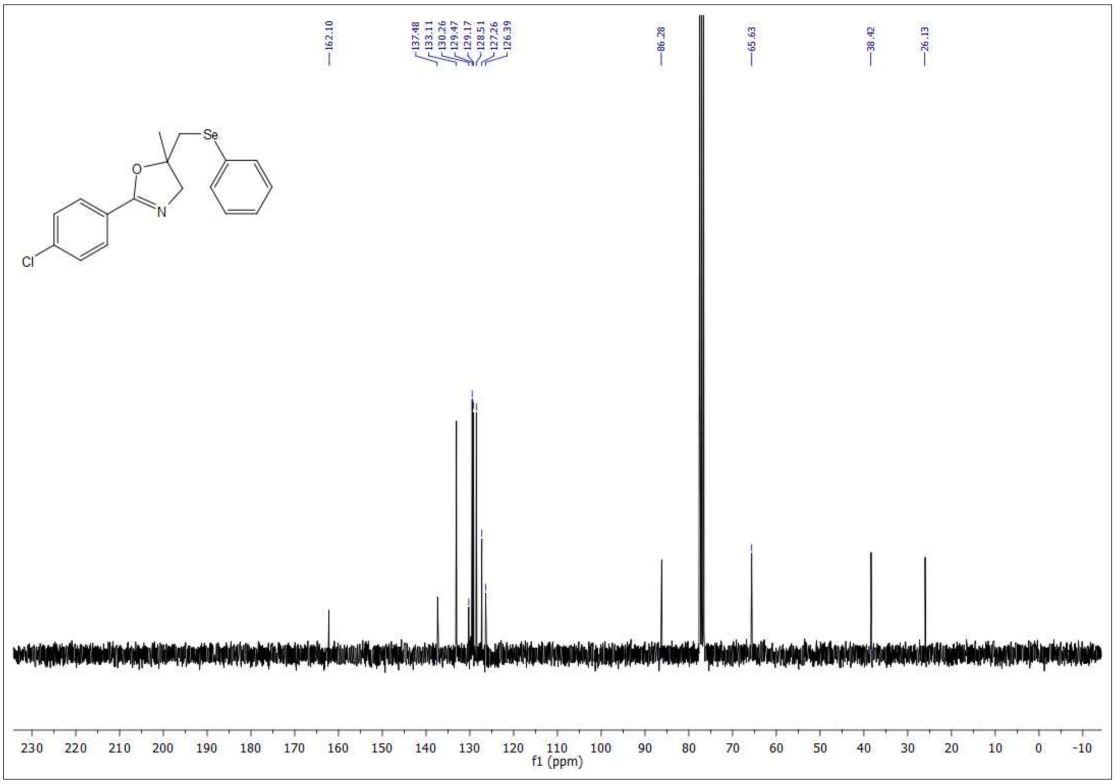

^{1}H NMR (300 MHz, $CDCl_3$) of 2-phenyl-5-((phenylselanyl)methylene)-4,5-dihydrooxazole (6ad)

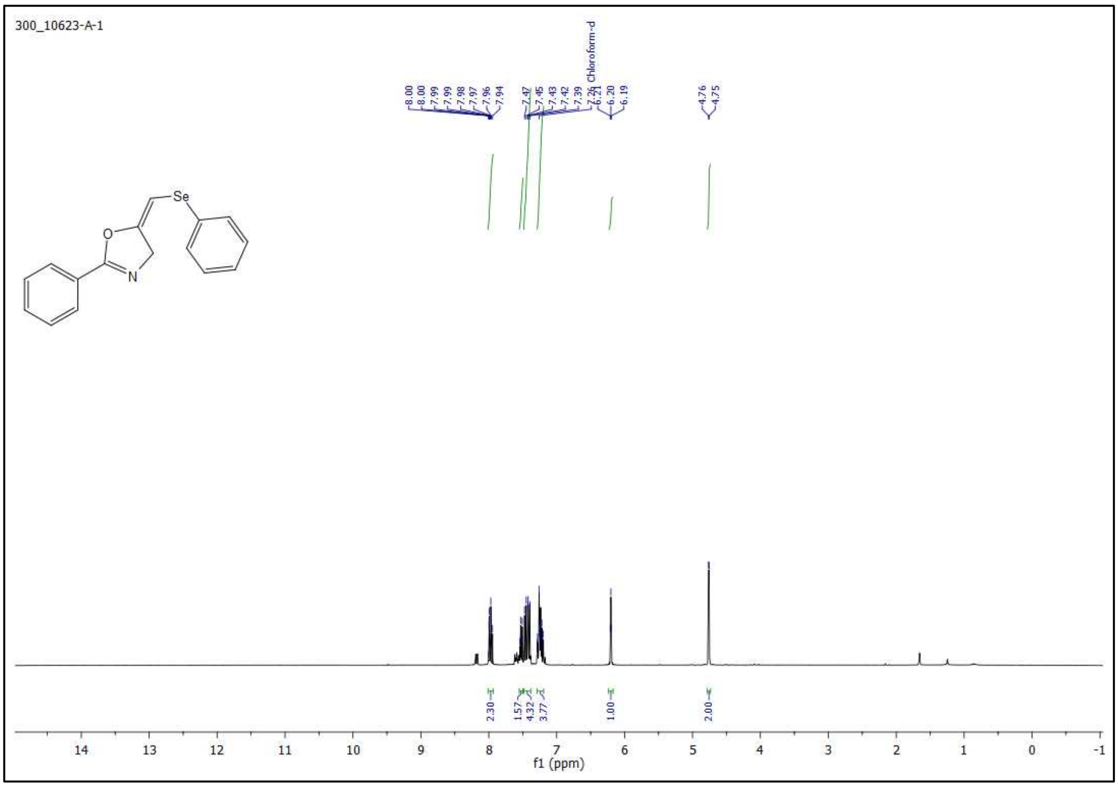

^{13}C {^{1}H} NMR (75 MHz, $CDCl_3$) of 2-phenyl-5-((phenylselanyl)methylene)-4,5-dihydrooxazole (6ad)

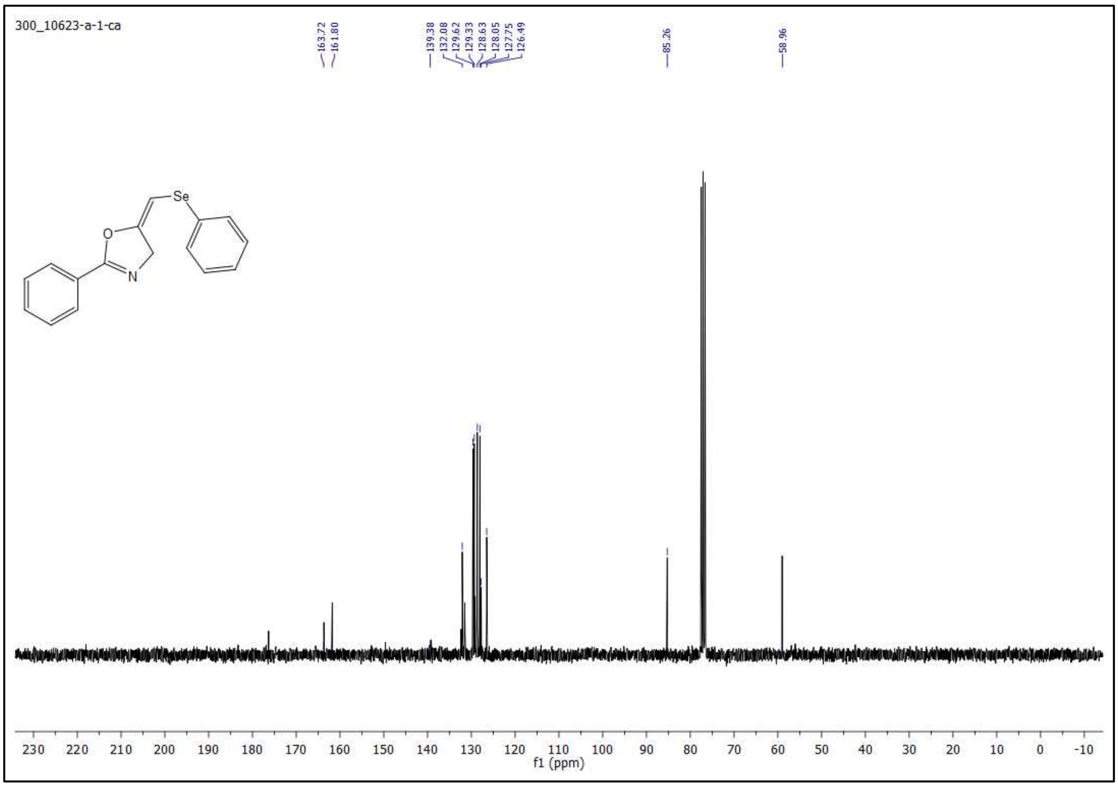

^{1}H NMR (300 MHz, $CDCl_3$) of 5-(((4-methoxyphenyl)selanyl)methyl)-2-phenyl-4,5-dihydrooxazole (6ae)

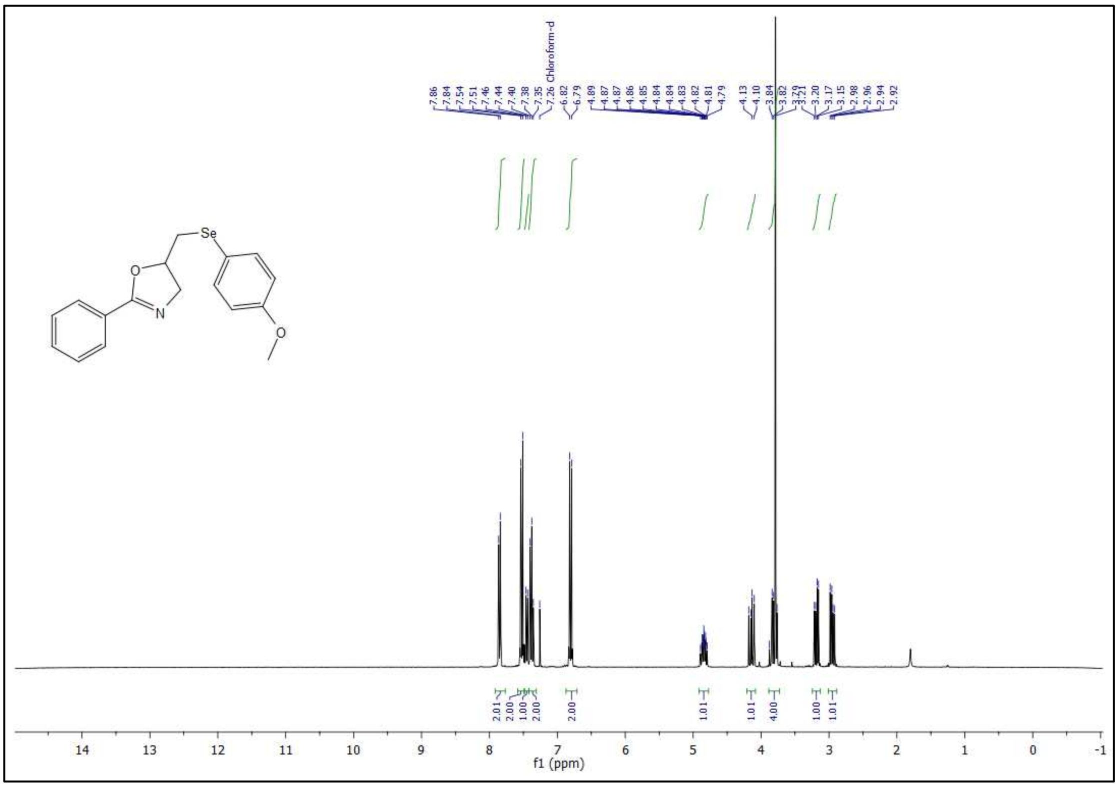

^{13}C {^{1}H} NMR (75 MHz, $CDCl_3$) of 5-(((4-methoxyphenyl)selanyl)methyl)-2-phenyl-4,5-dihydrooxazole (6ae)

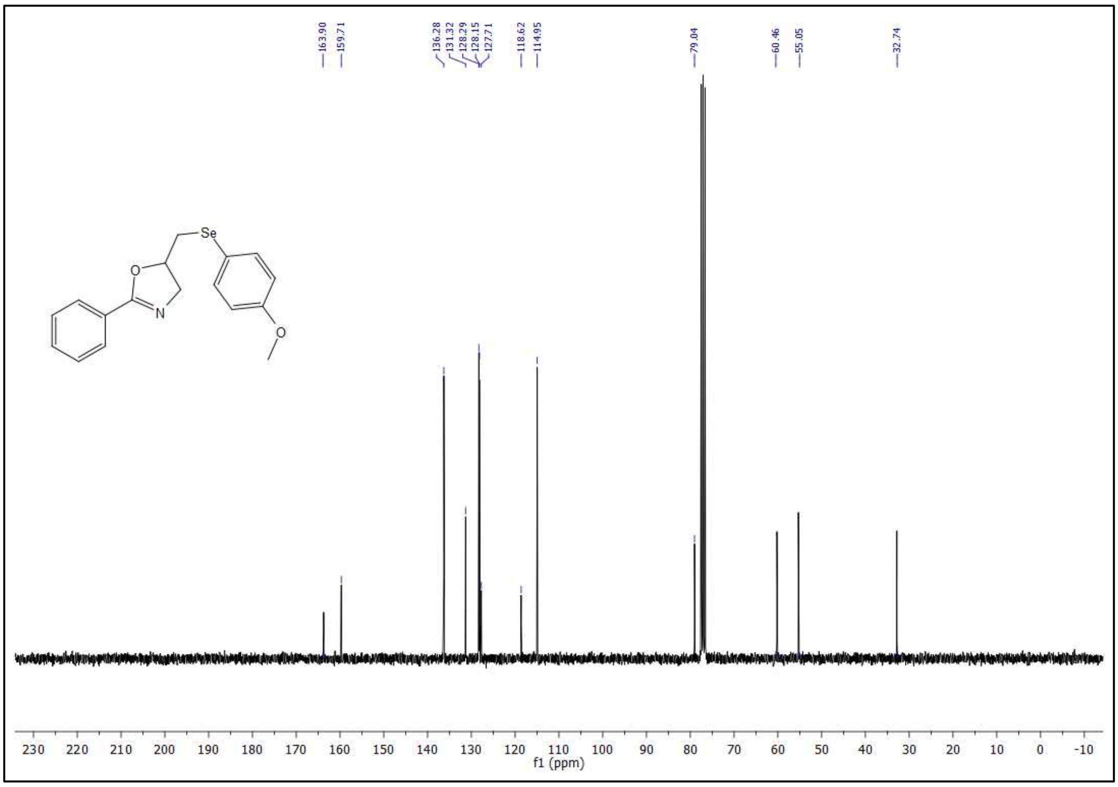

^{1}H NMR (300 MHz, $CDCl_3$) of 2-phenyl-5-((p-tolylselanyl)methyl)-4,5-dihydrooxazole (6af)

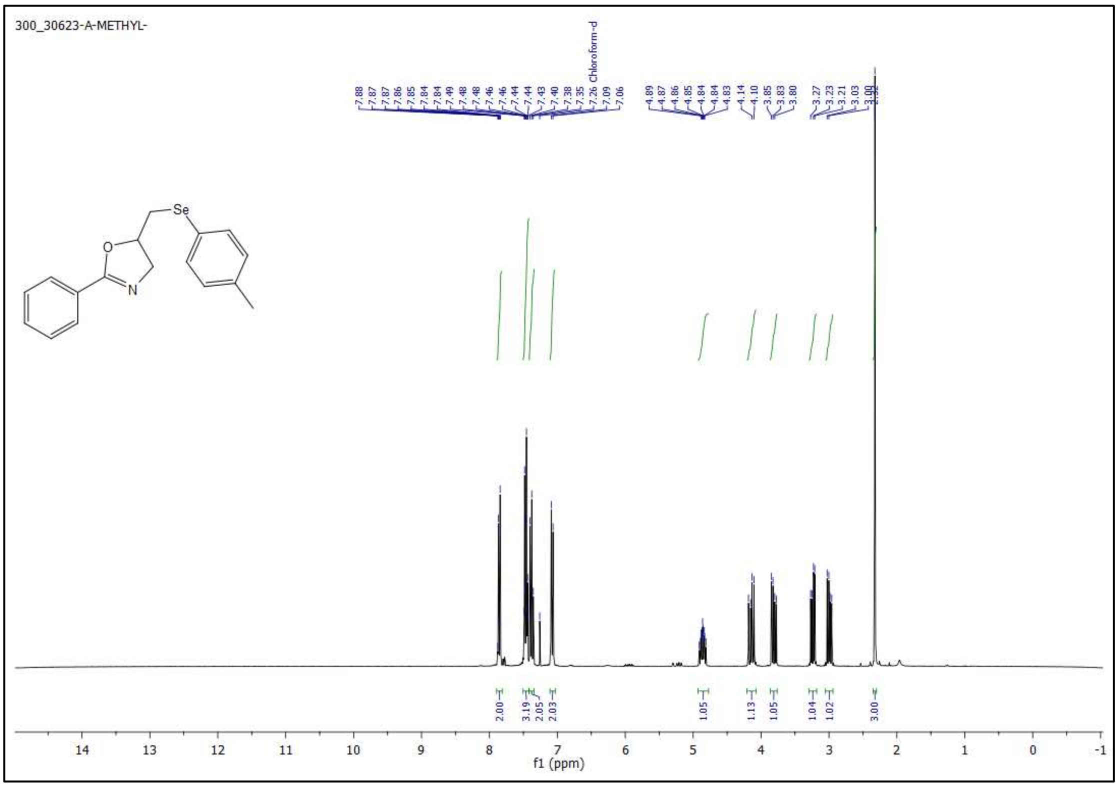

^{13}C {^{1}H} NMR (75 MHz, $CDCl_3$) of 2-phenyl-5-((p-tolylselanyl)methyl)-4,5-dihydrooxazole (6af)

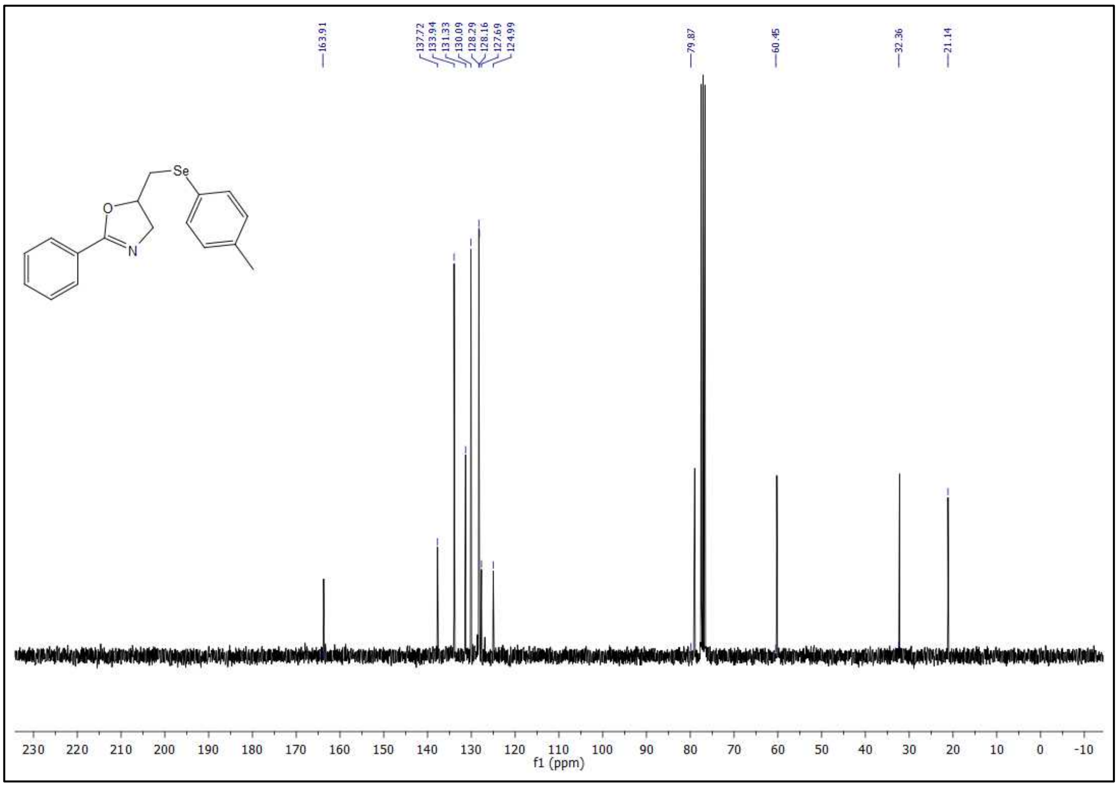

^{1}H NMR (300 MHz, CDCl$_3$) of 5-((benzylselanyl)methyl)-2-phenyl-4,5-dihydrooxazole (6ag)

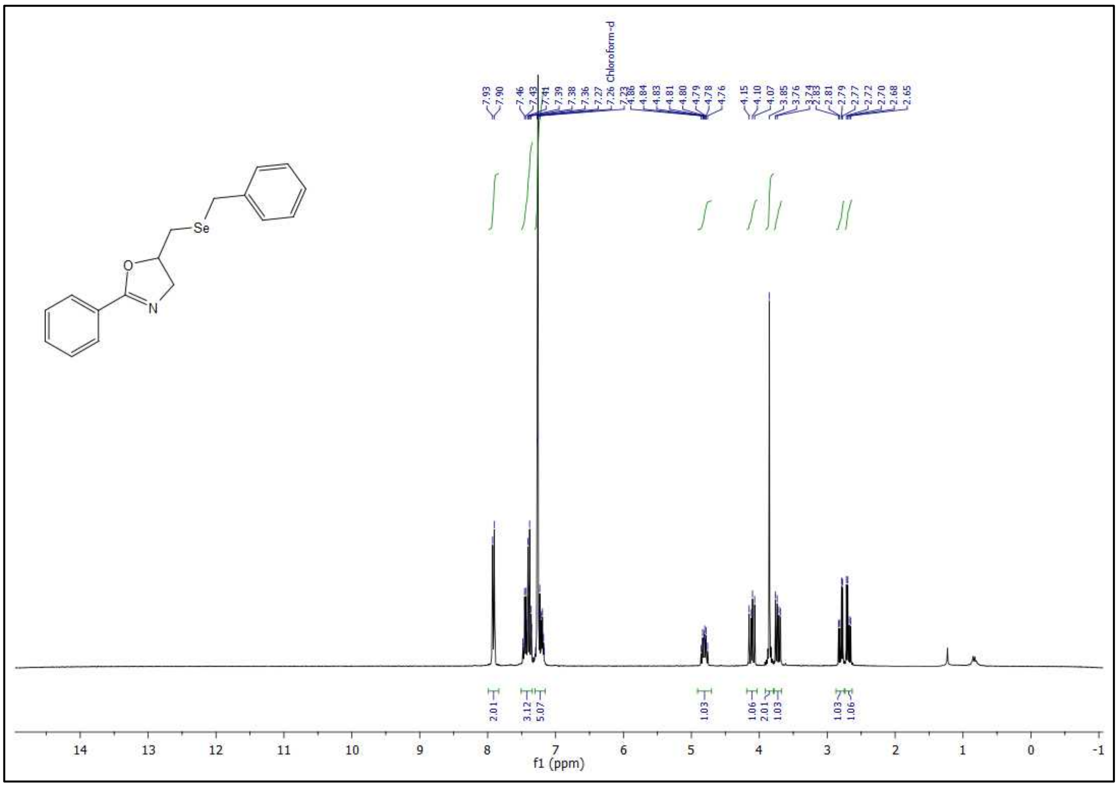

^{13}C {^{1}H} NMR (75 MHz, CDCl$_3$) of 5-((benzylselanyl)methyl)-2-phenyl-4,5-dihydrooxazole (6ag)

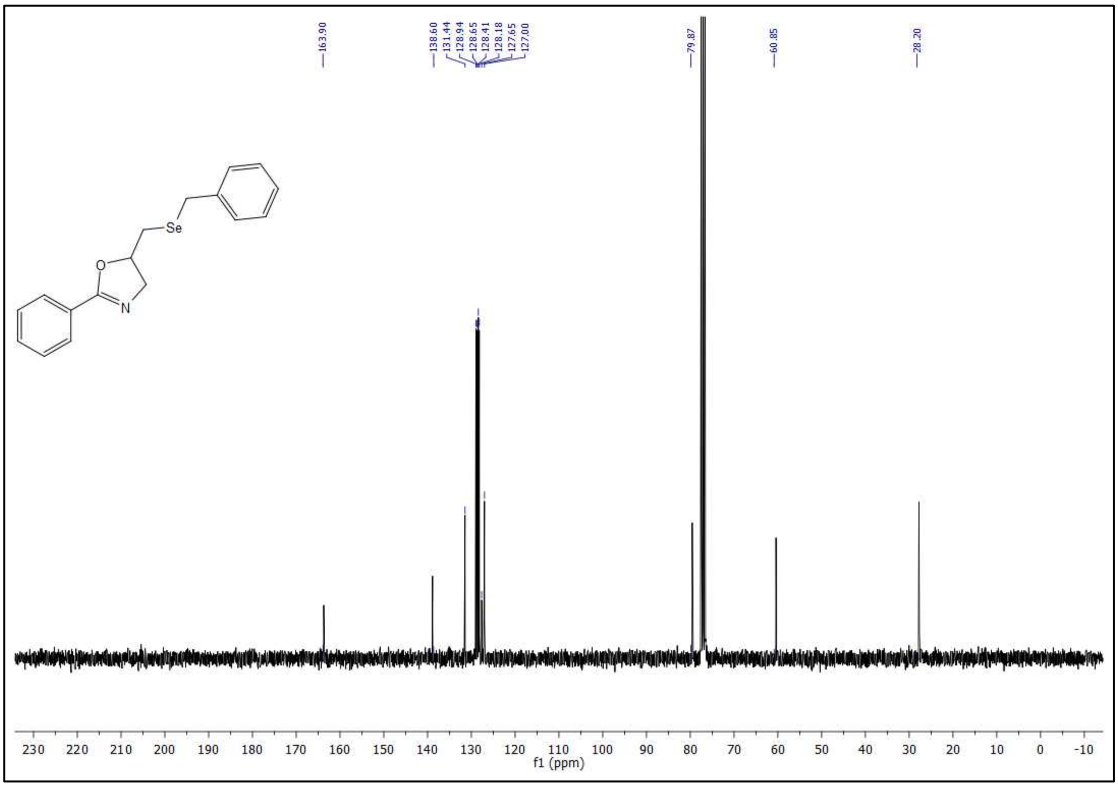

^{1}H NMR (300 MHz, $CDCl_3$) of 1-phenylbut-3-en-1-one oxime (7a)

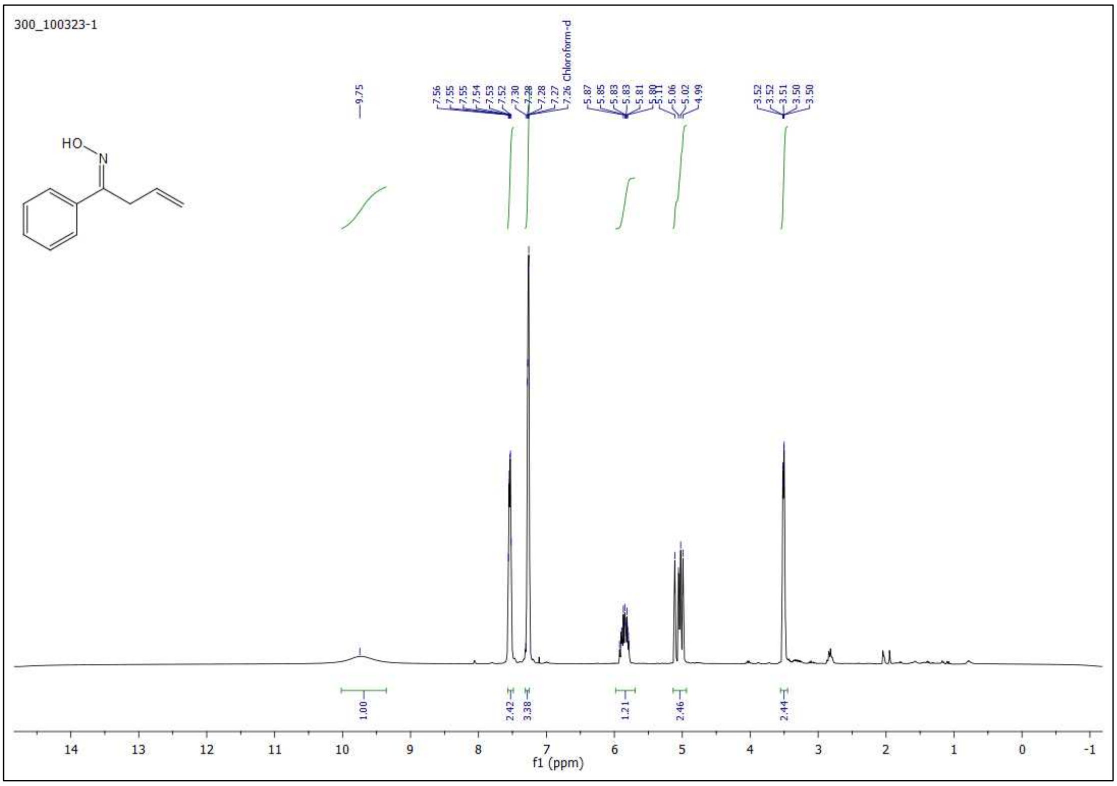

$^{13}C\{^{1}H\}$ NMR (75 MHz, $CDCl_3$) of 1-phenylbut-3-en-1-one oxime (7a)

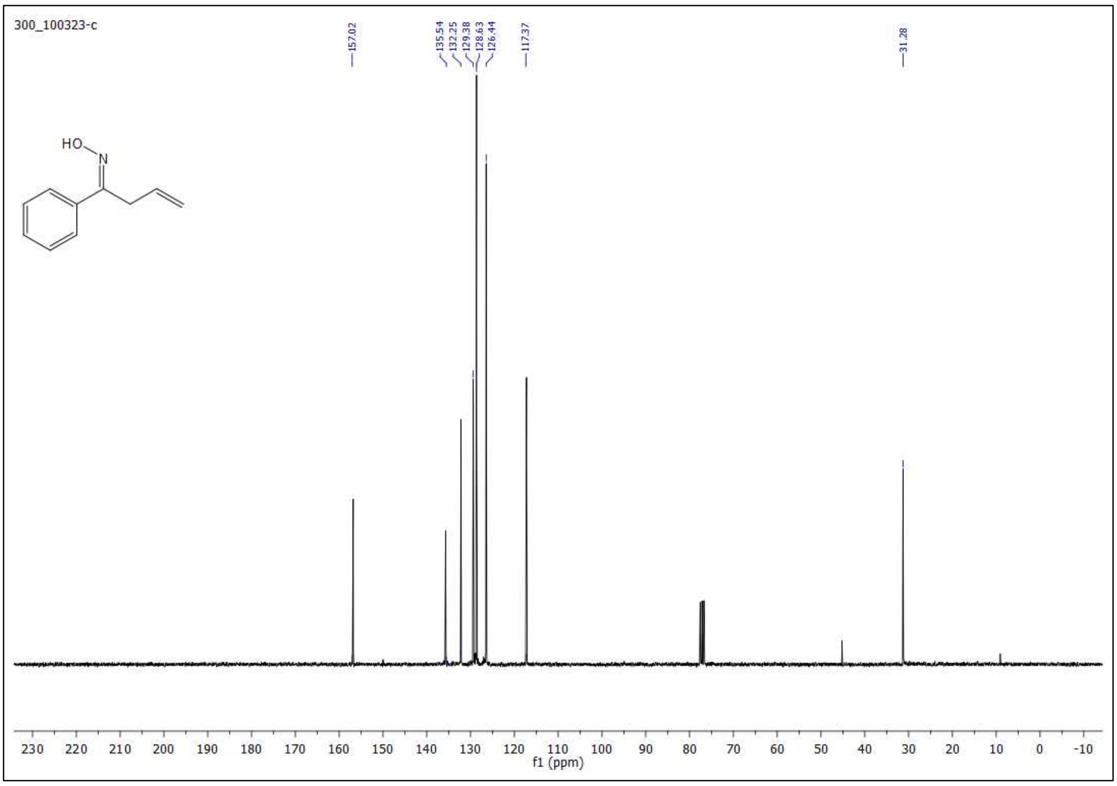

^{1}H NMR (300 MHz, $CDCl_3$) of 1-(4-methoxyphenyl)but-3-en-1-one oxime (7b)

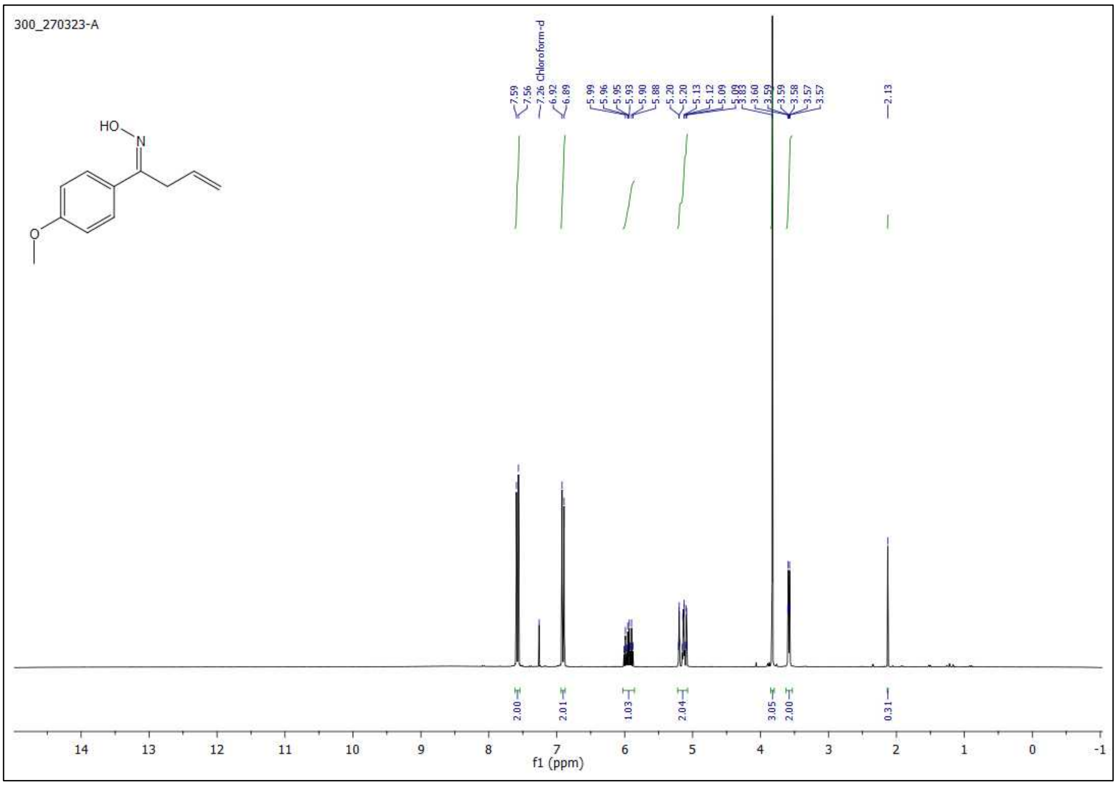

$^{13}C\{^{1}H\}$ NMR (75 MHz, $CDCl_3$) of 1-(4-methoxyphenyl)but-3-en-1-one oxime (7b)

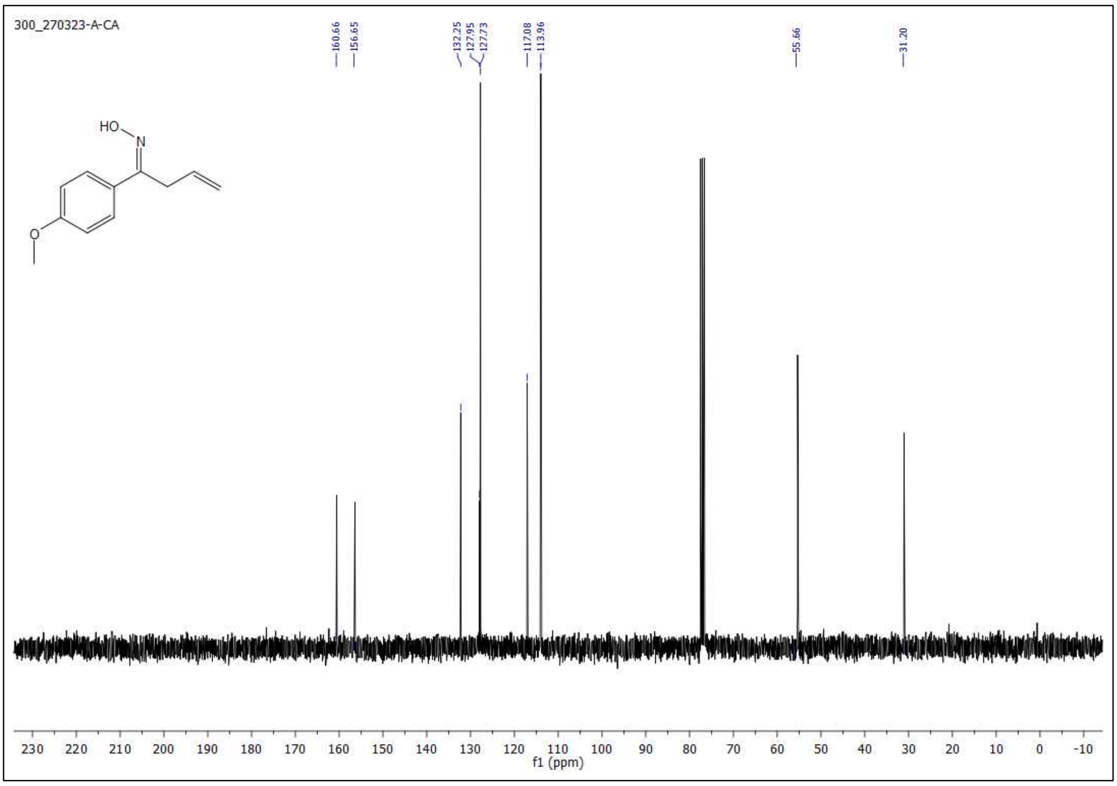

^{1}H NMR (300 MHz, CDCl$_3$) of 1-(*p*-tolyl)but-3-en-1-one oxime (7c)

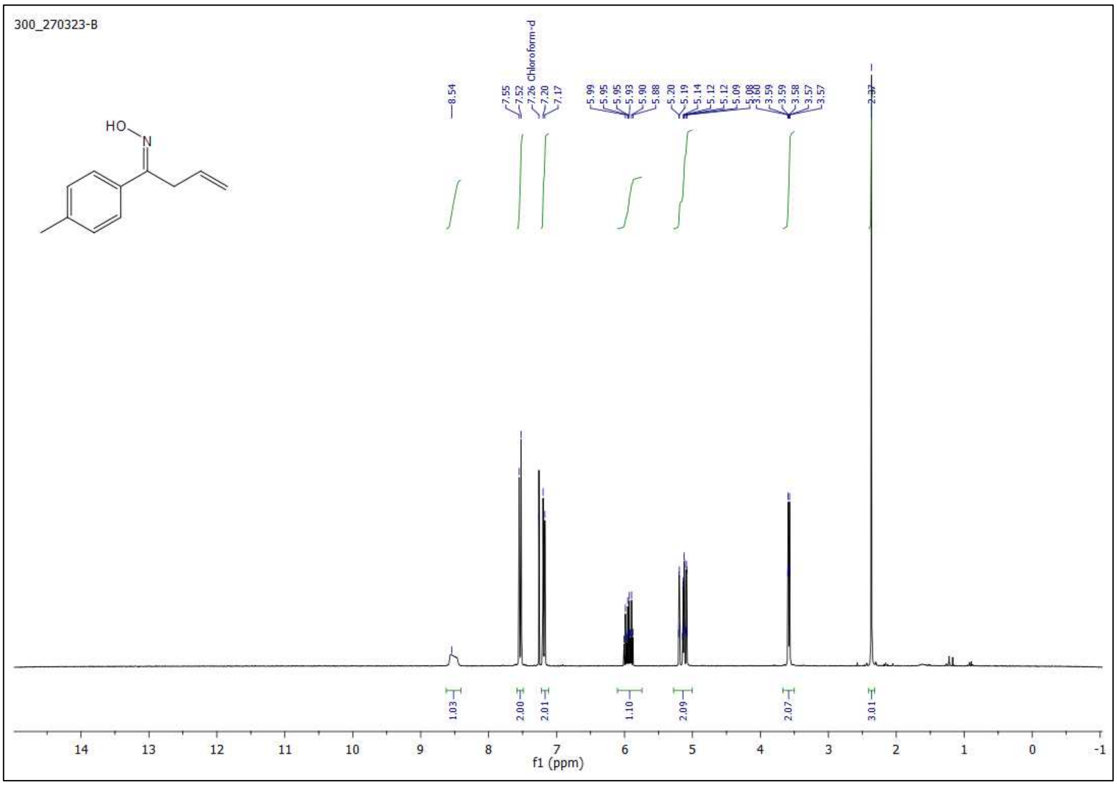

^{13}C {^{1}H} NMR (75 MHz, CDCl$_3$) of 1-(*p*-tolyl)but-3-en-1-one oxime (7c)

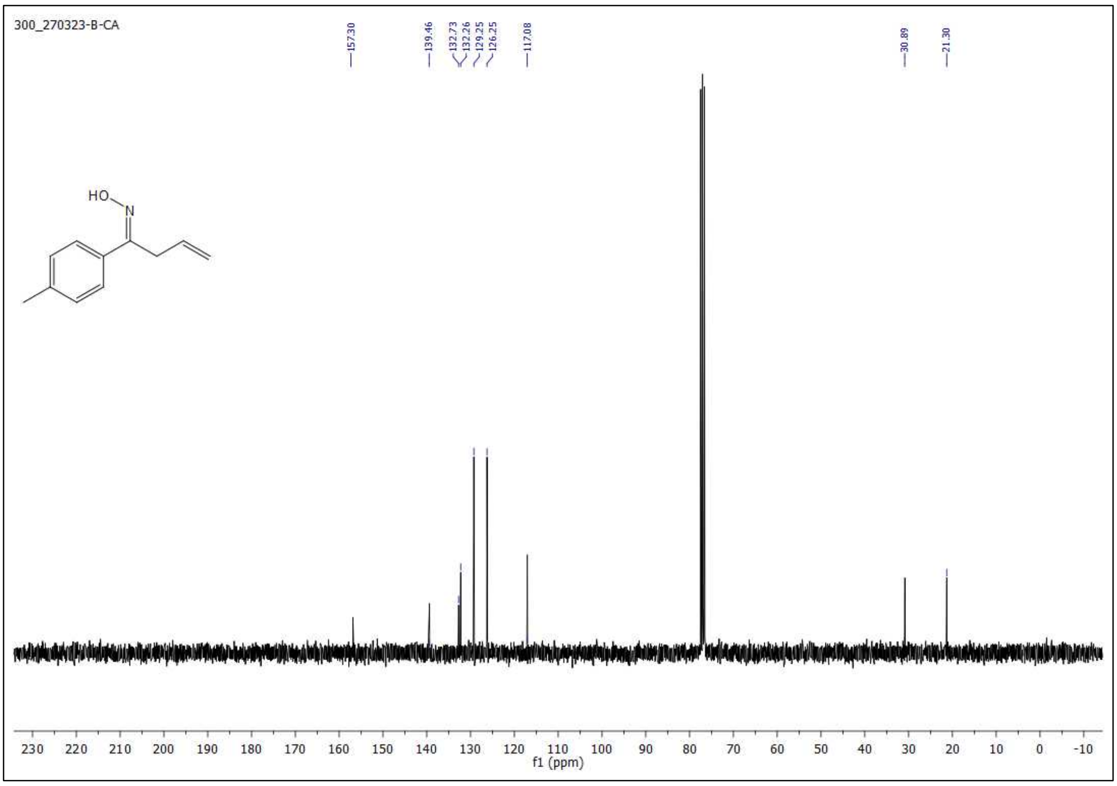

^{1}H NMR (500 MHz, $CDCl_3$) of 1-(4-fluorophenyl)but-3-en-1-one oxime (7d)

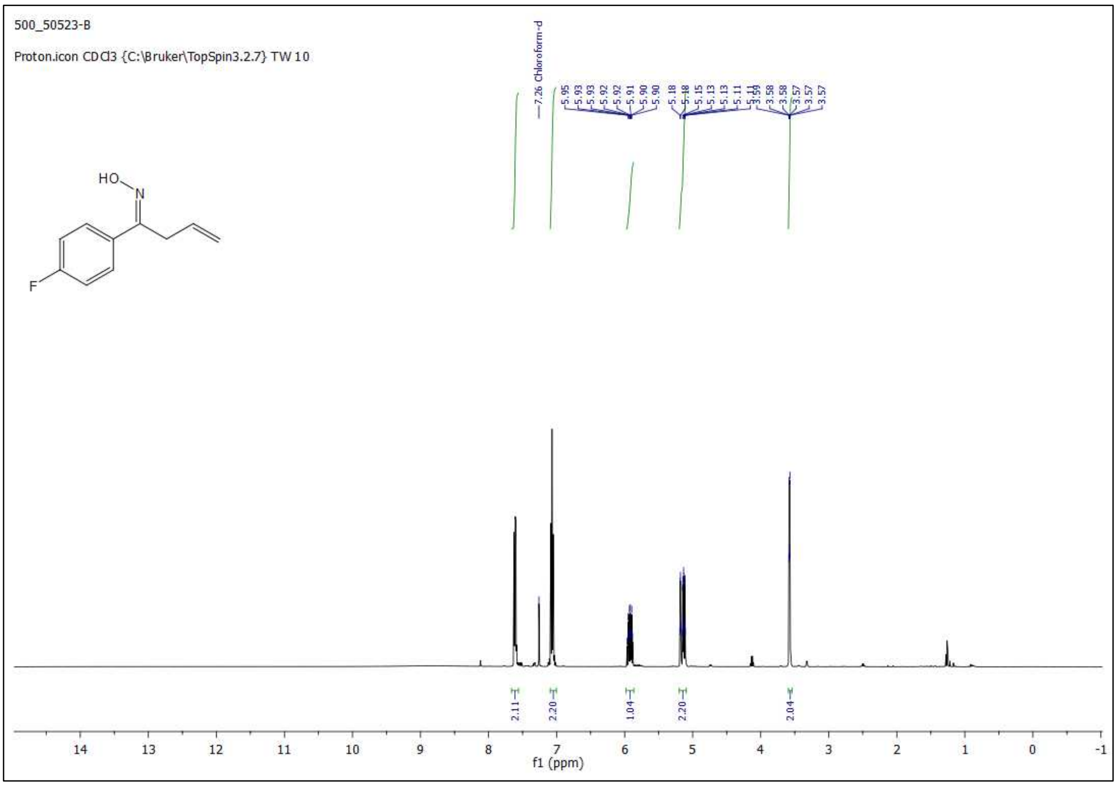

^{13}C {^{1}H} NMR (75 MHz, $CDCl_3$) of 1-(4-fluorophenyl)but-3-en-1-one oxime (7d)

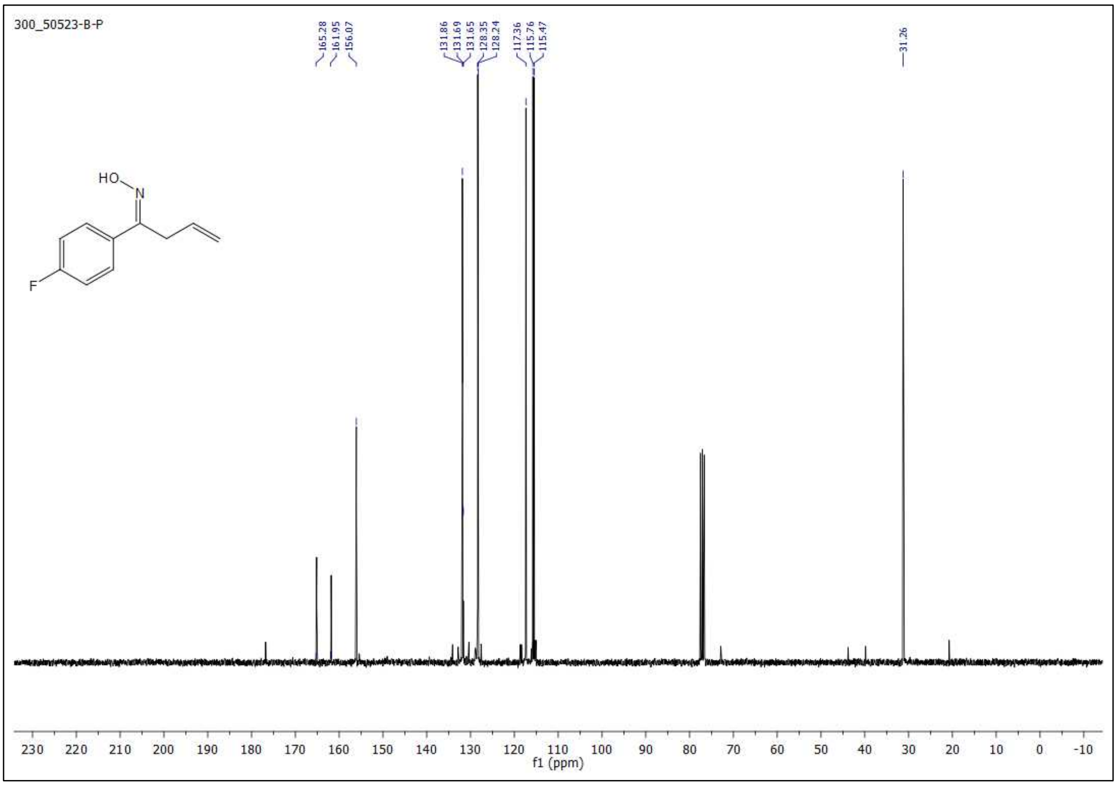

^{1}H NMR (500 MHz, $CDCl_3$) of 1-(4-bromophenyl)but-3-en-1-one oxime (7e)

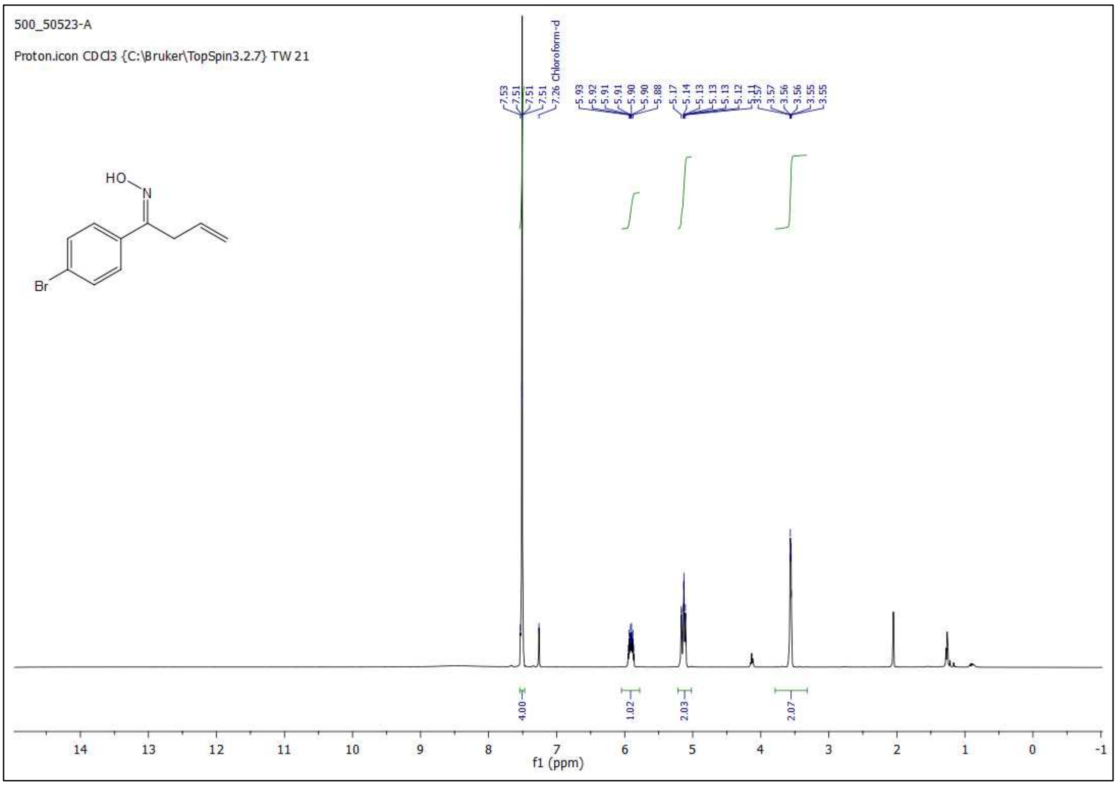

^{13}C {^{1}H} NMR (75 MHz, $CDCl_3$) of 1-(4-bromophenyl)but-3-en-1-one oxime (7e)

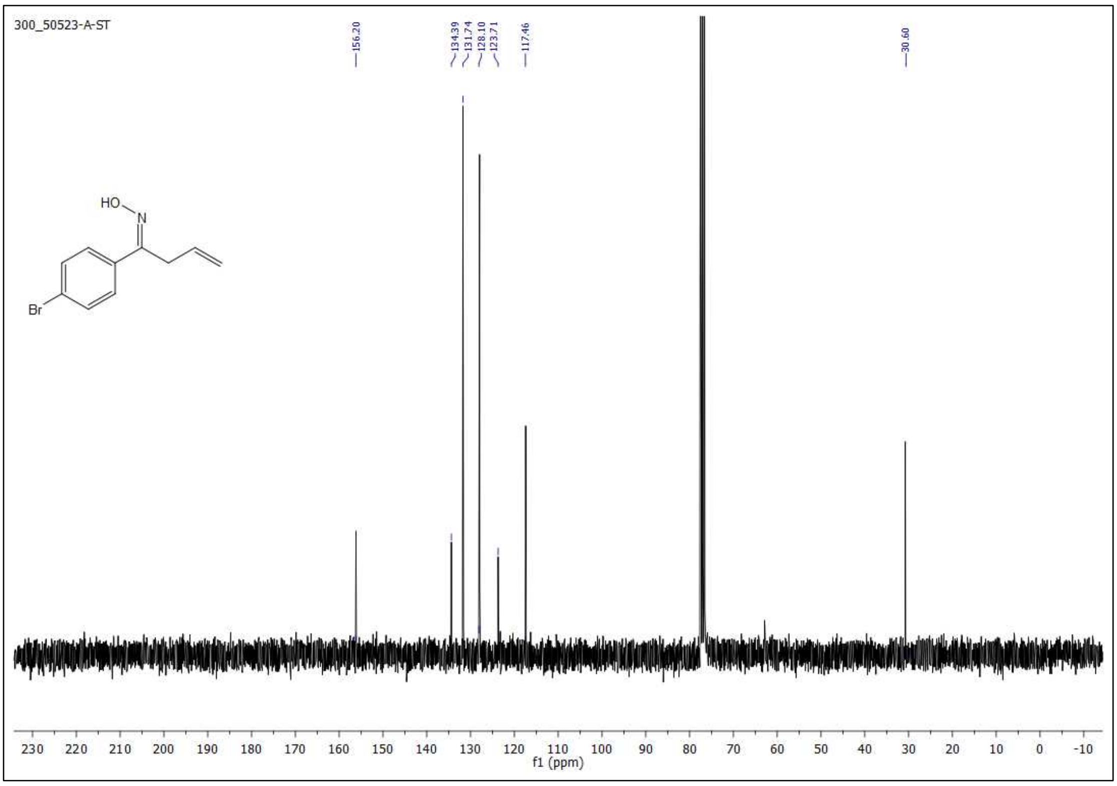

^{1}H NMR (300 MHz, $CDCl_3$) of 1-(3-methoxyphenyl)but-3-en-1-one oxime (7f)

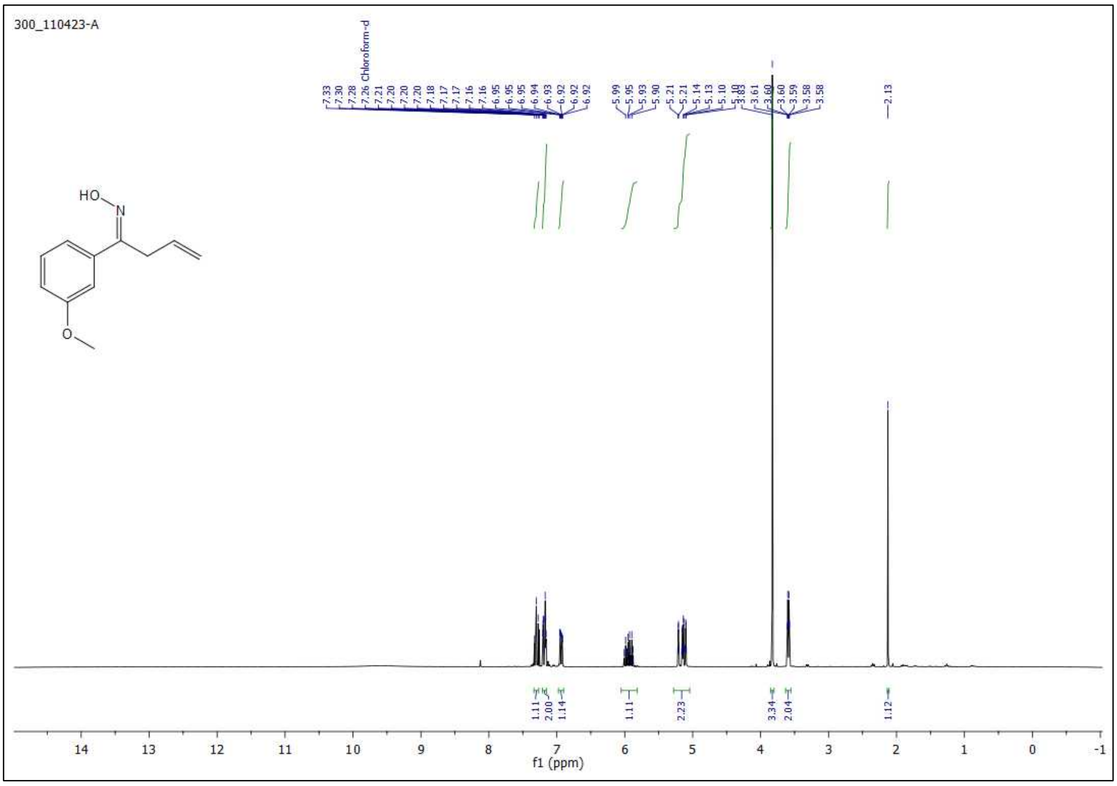

^{13}C {^{1}H} NMR (75 MHz, $CDCl_3$) of 1-(3-methoxyphenyl)but-3-en-1-one oxime (7f)

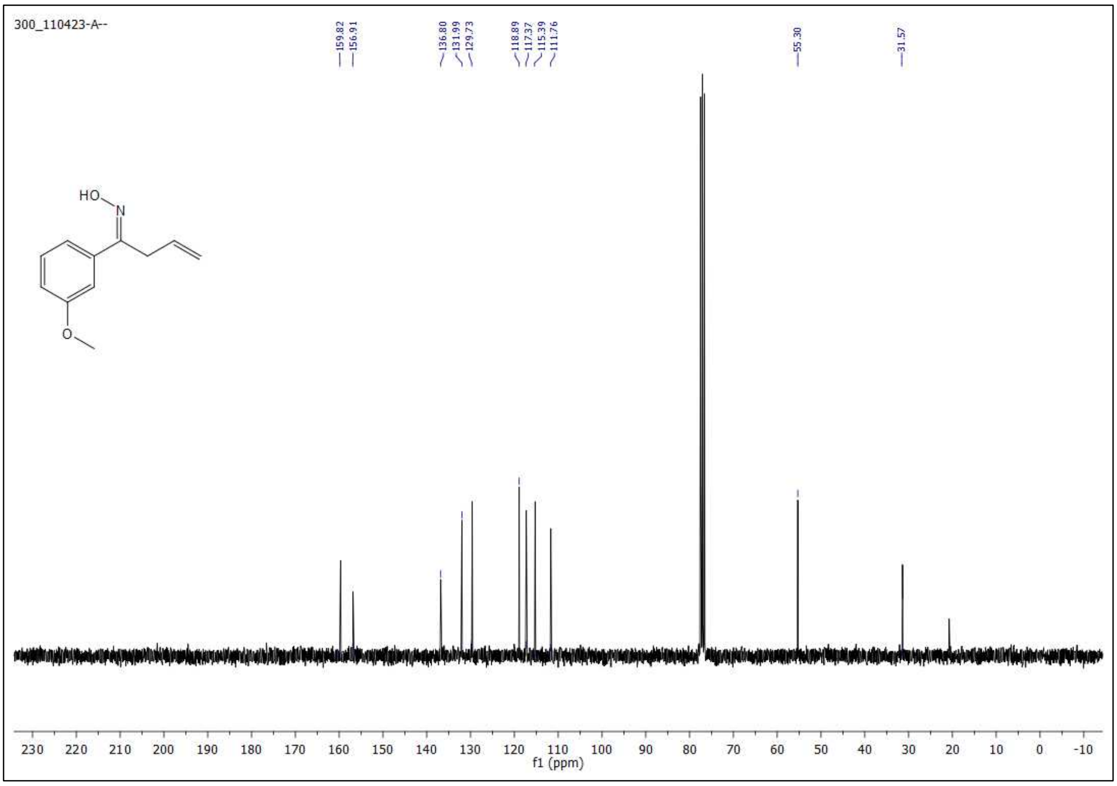

^{1}H NMR (300 MHz, $CDCl_3$) of 3-phenyl-5-((phenylselanyl)methyl)-4,5-dihydroisoxazole (8a)

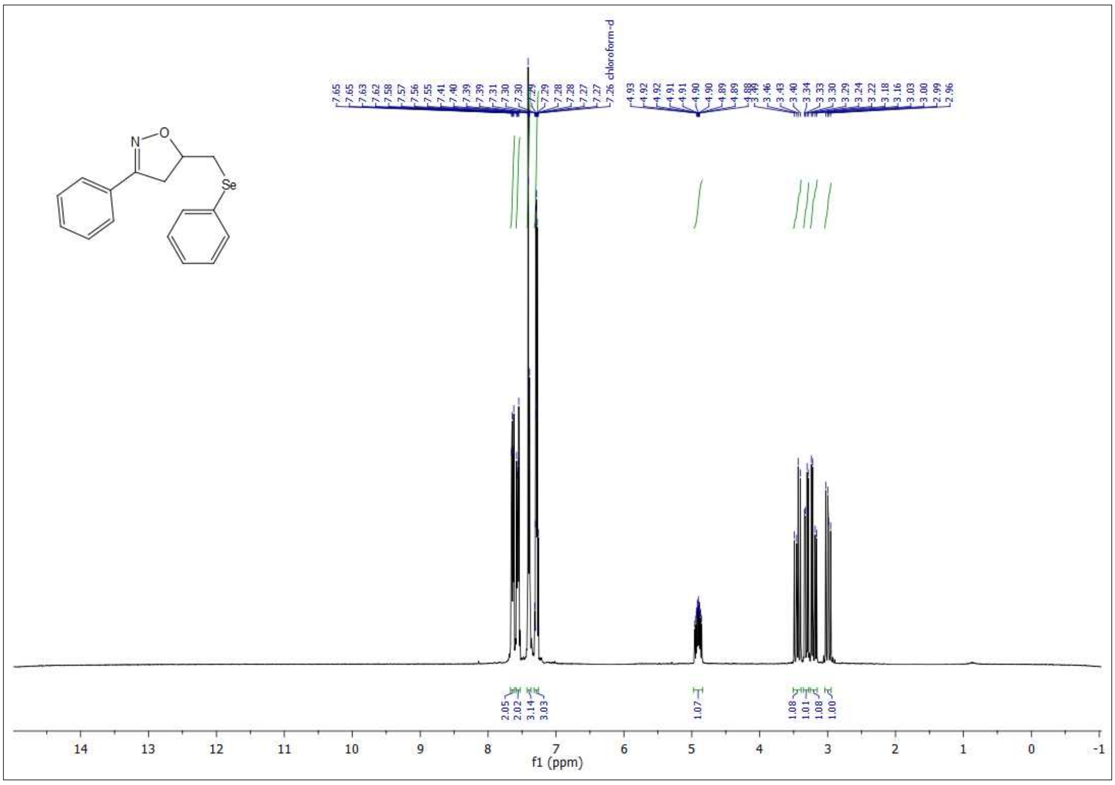

$^{13}C\{^{1}H\}$ NMR (75 MHz, $CDCl_3$) of 3-phenyl-5-((phenylselanyl)methyl)-4,5-dihydroisoxazole (8a)

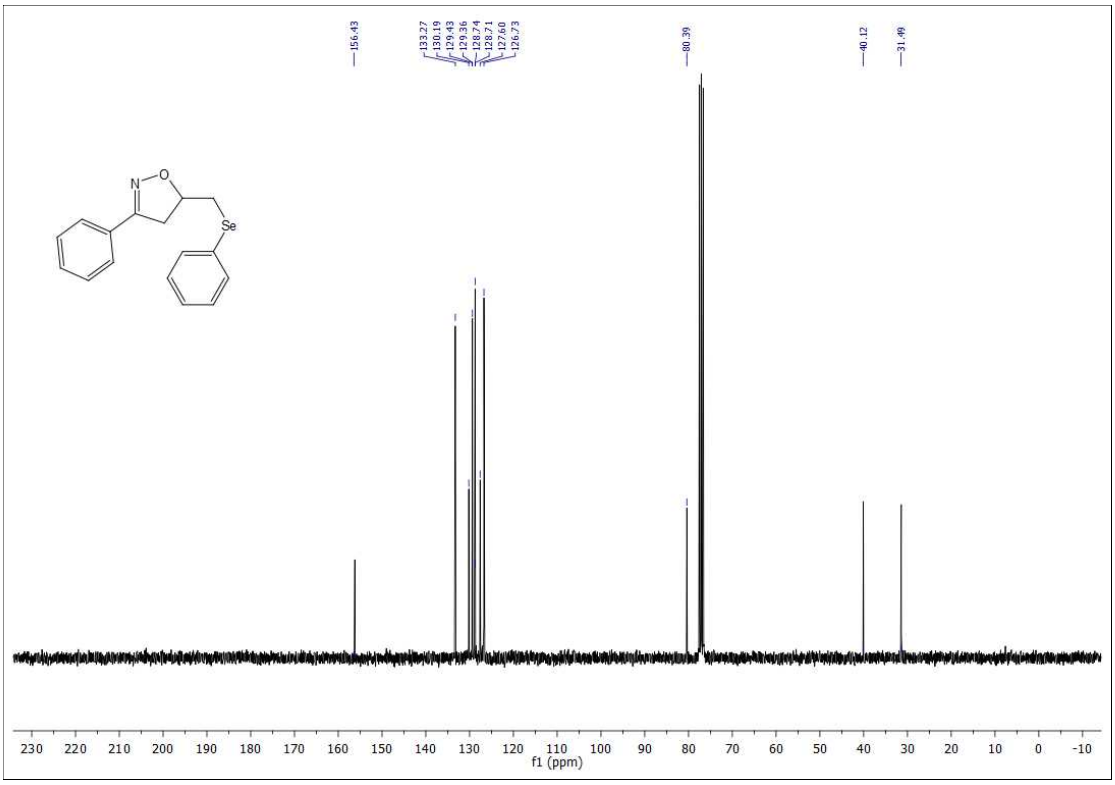

^{1}H NMR (300 MHz, $CDCl_3$) of 3-(4-methoxyphenyl)-5-((phenylselanyl)methyl)-4,5-dihydroisoxazole (8b)

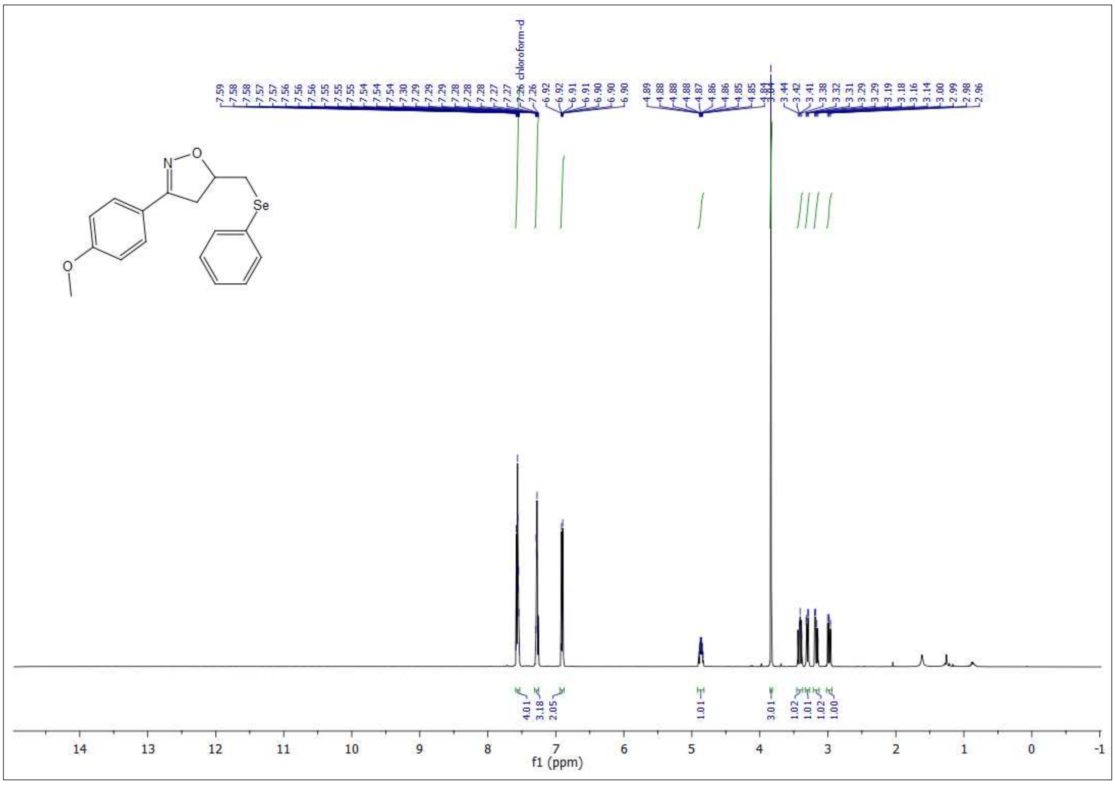

^{13}C {^{1}H} NMR (75 MHz, $CDCl_3$) of 3-(4-methoxyphenyl)-5-((phenylselanyl)methyl)-4,5-dihydroisoxazole (8b)

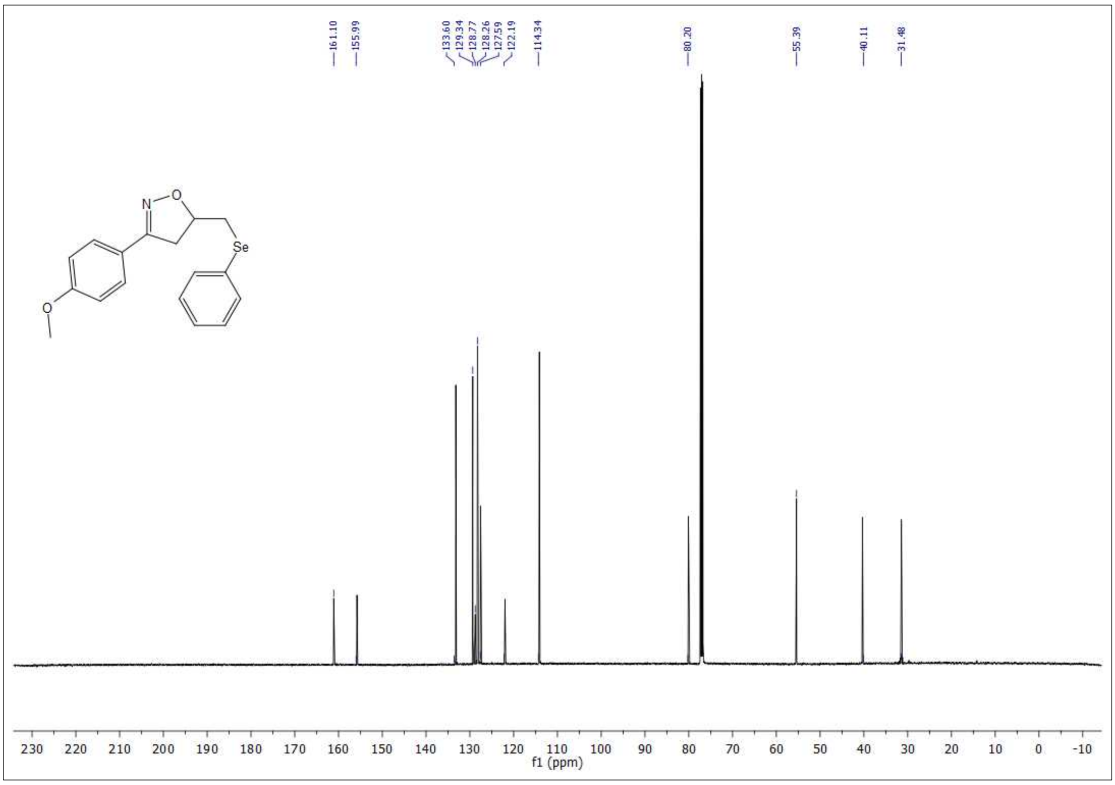

^{1}H NMR (300 MHz, $CDCl_3$) of 5-((phenylselanyl)methyl)-3-(p-tolyl)-4,5-dihydroisoxazole (8c)

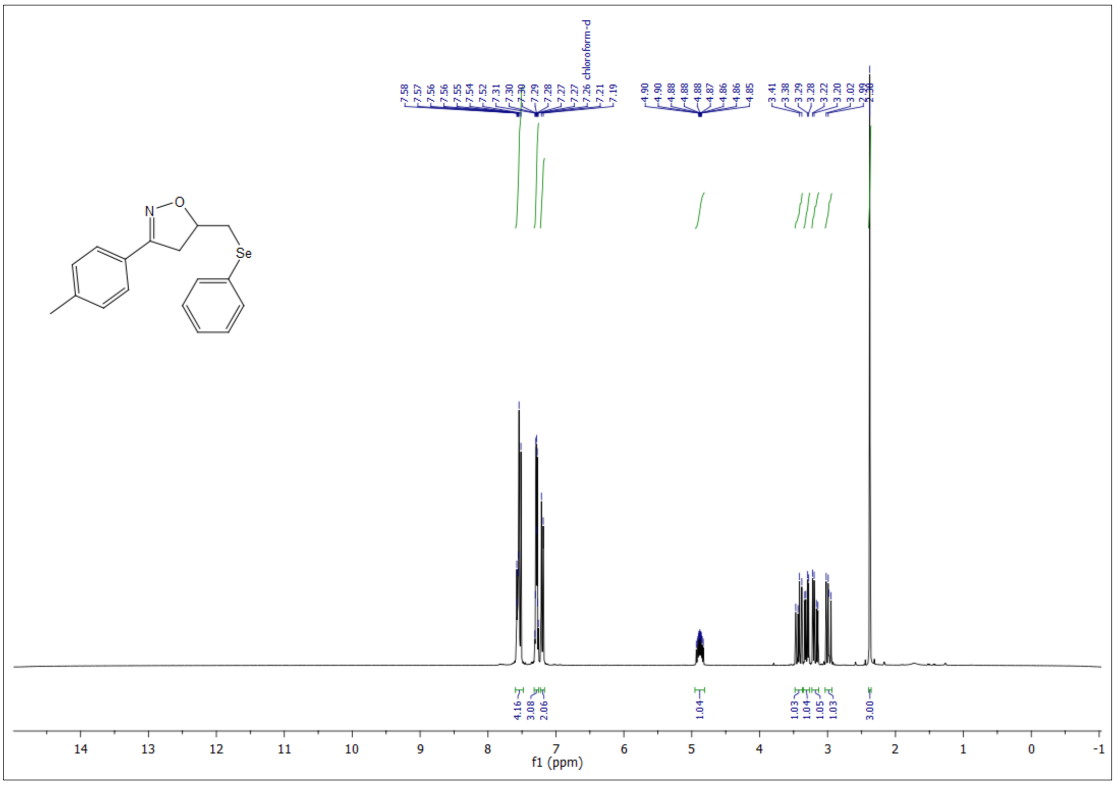

^{13}C {^{1}H} NMR (75 MHz, $CDCl_3$) of 5-((phenylselanyl)methyl)-3-(p-tolyl)-4,5-dihydroisoxazole (8c)

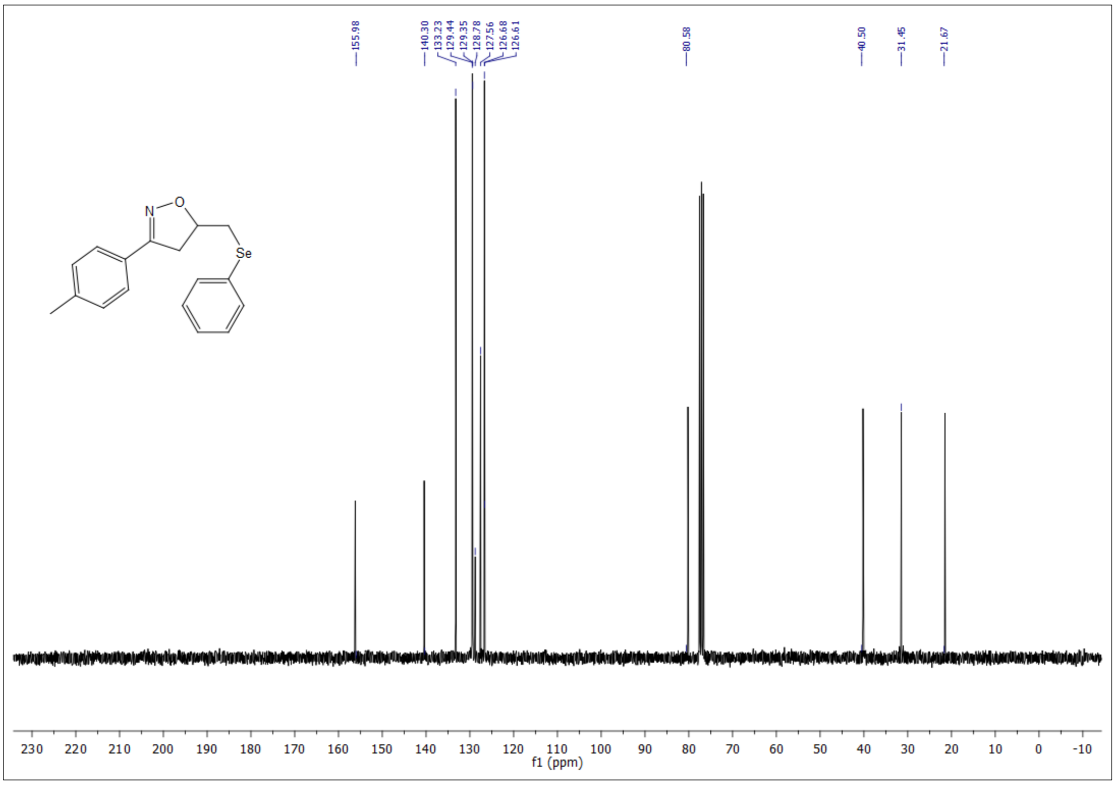

^{1}H NMR (300 MHz, $CDCl_3$) of 3-(4-fluorophenyl)-5-((phenylselanyl)methyl)-4,5-dihydroisoxazole (8d)

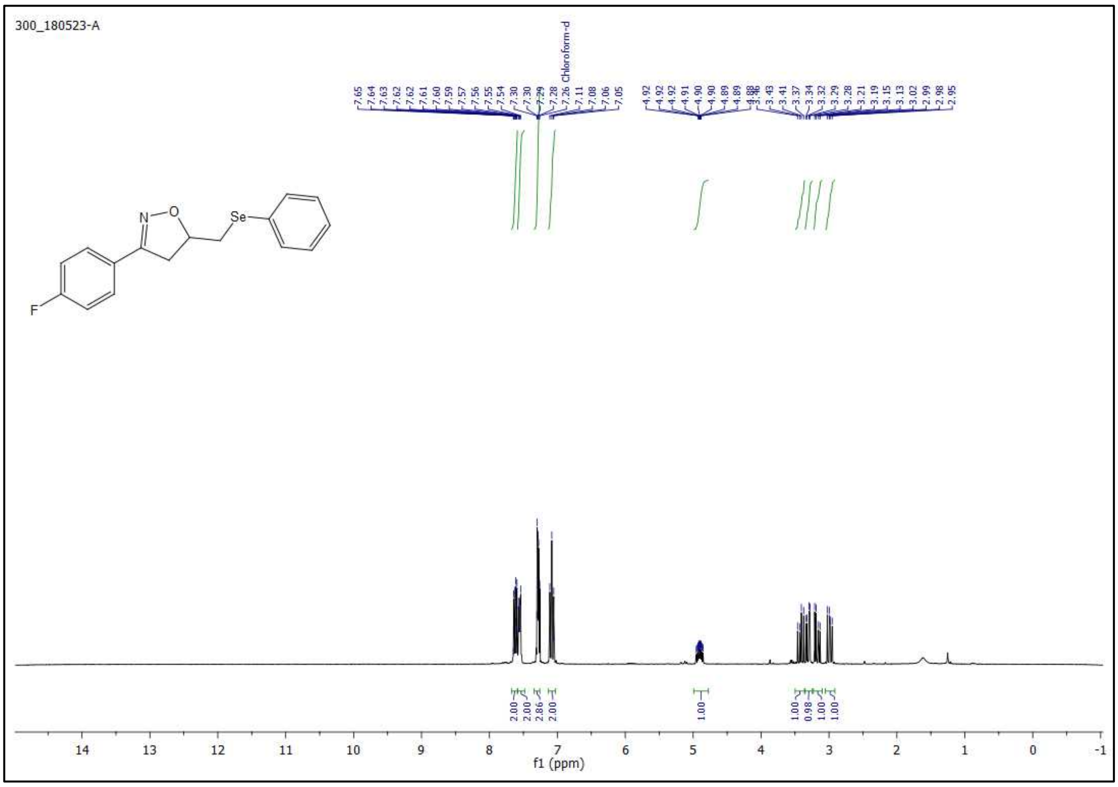

$^{13}C\{^{1}H\}$ NMR (75 MHz, $CDCl_3$) of 3-(4-fluorophenyl)-5-((phenylselanyl)methyl)-4,5-dihydroisoxazole (8d)

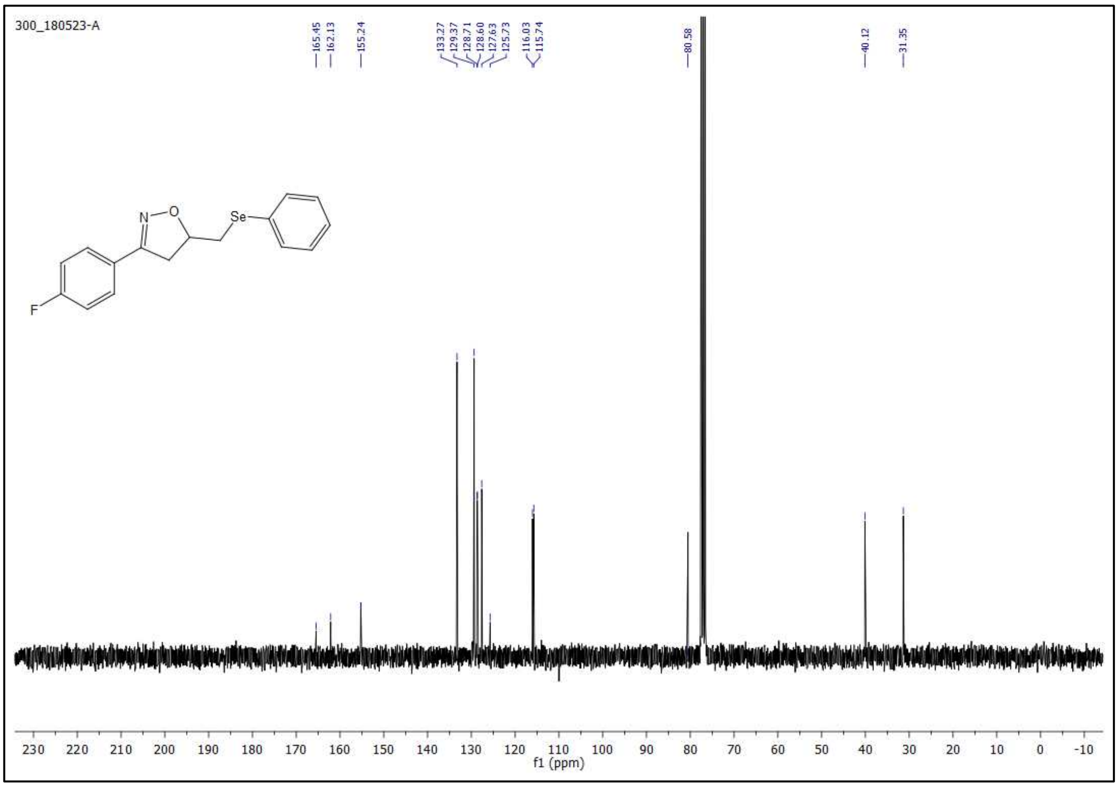

^{1}H NMR (300 MHz, $CDCl_3$) of 3-(4-bromophenyl)-5-((phenylselanyl)methyl)-4,5-dihydroisoxazole (8e)

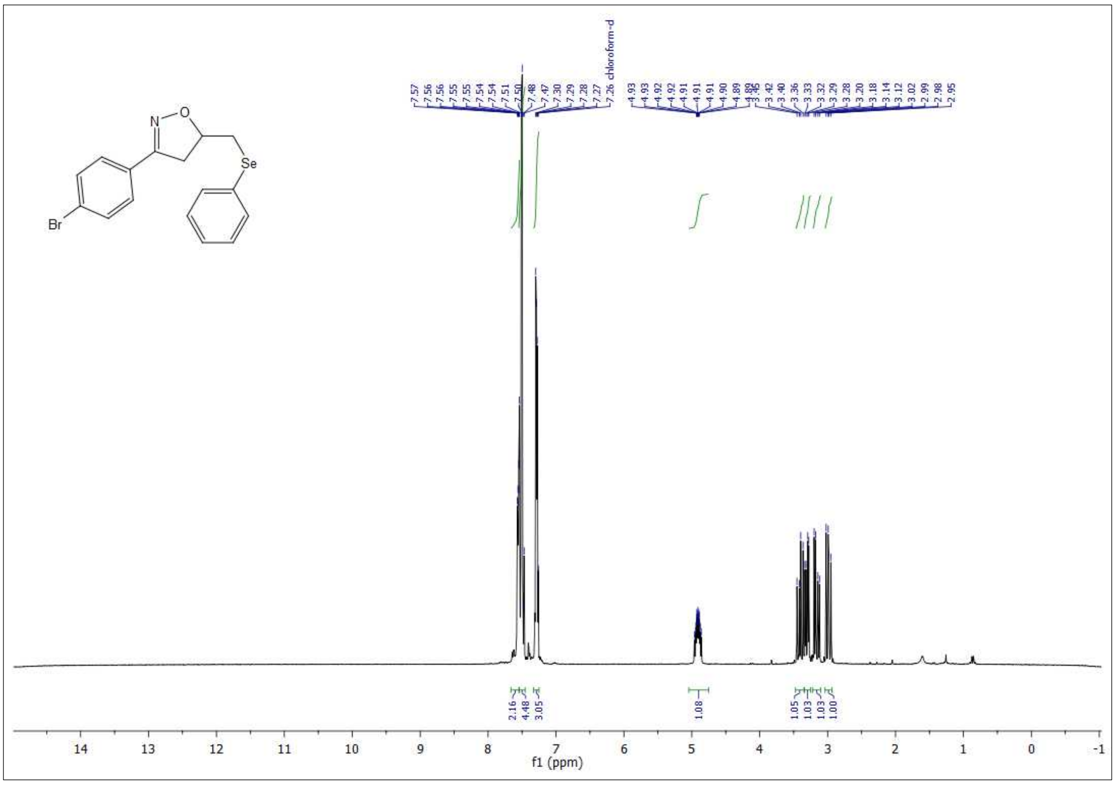

^{13}C {^{1}H} NMR (75 MHz, $CDCl_3$) of 3-(4-bromophenyl)-5-((phenylselanyl)methyl)-4,5-dihydroisoxazole (8e)

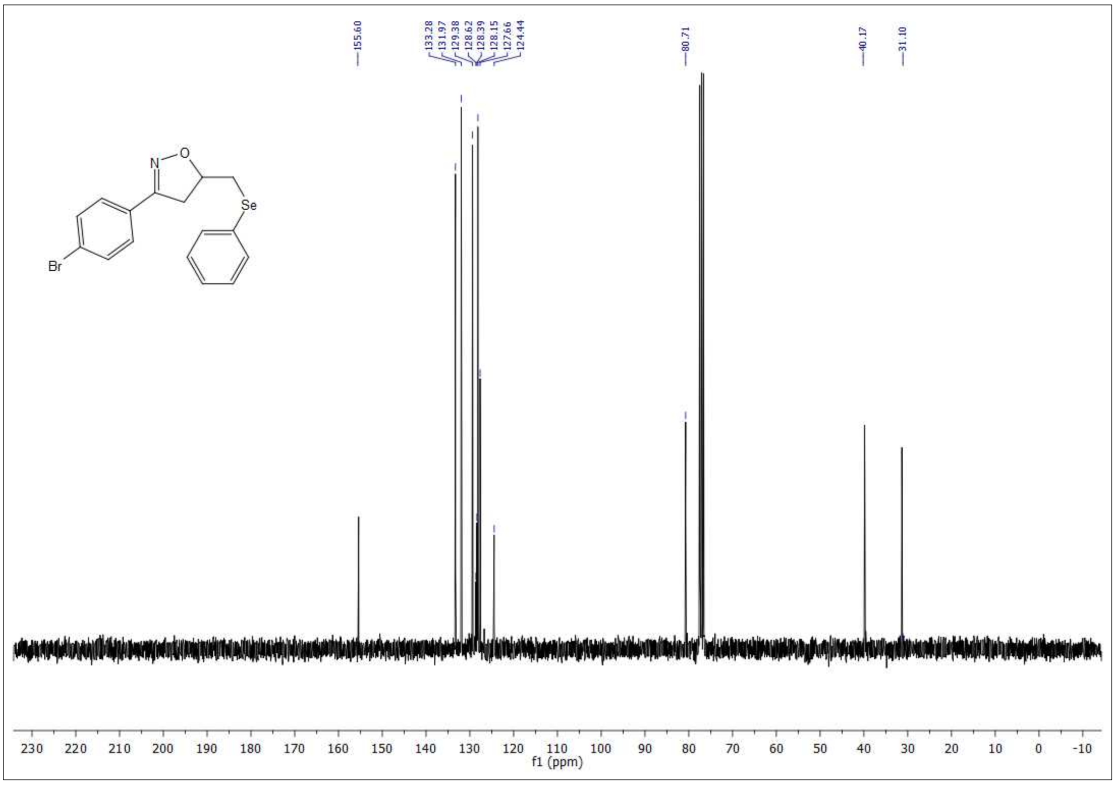

^{1}H NMR (300 MHz, $CDCl_3$) of 3-(3-methoxyphenyl)-5-((phenylselanyl)methyl)-4,5-dihydroisoxazole (8f)

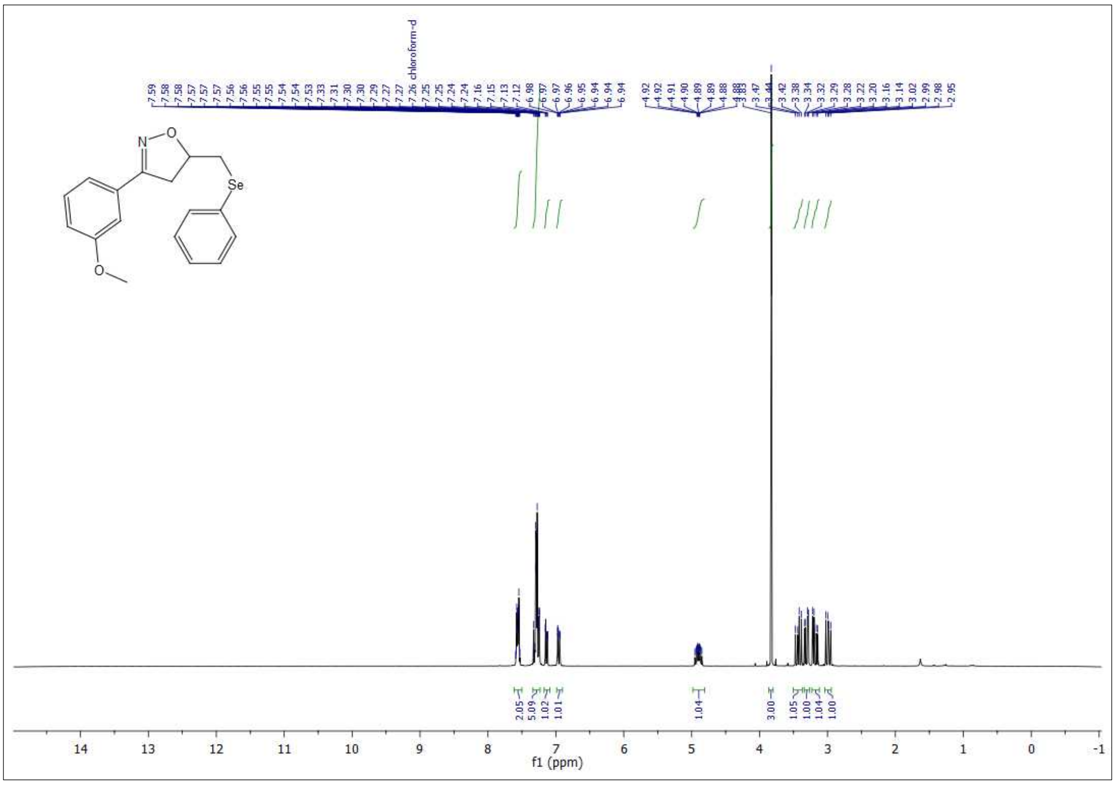

^{13}C {^{1}H} NMR (75 MHz, $CDCl_3$) of 3-(3-methoxyphenyl)-5-((phenylselanyl)methyl)-4,5-dihydroisoxazole (8f)

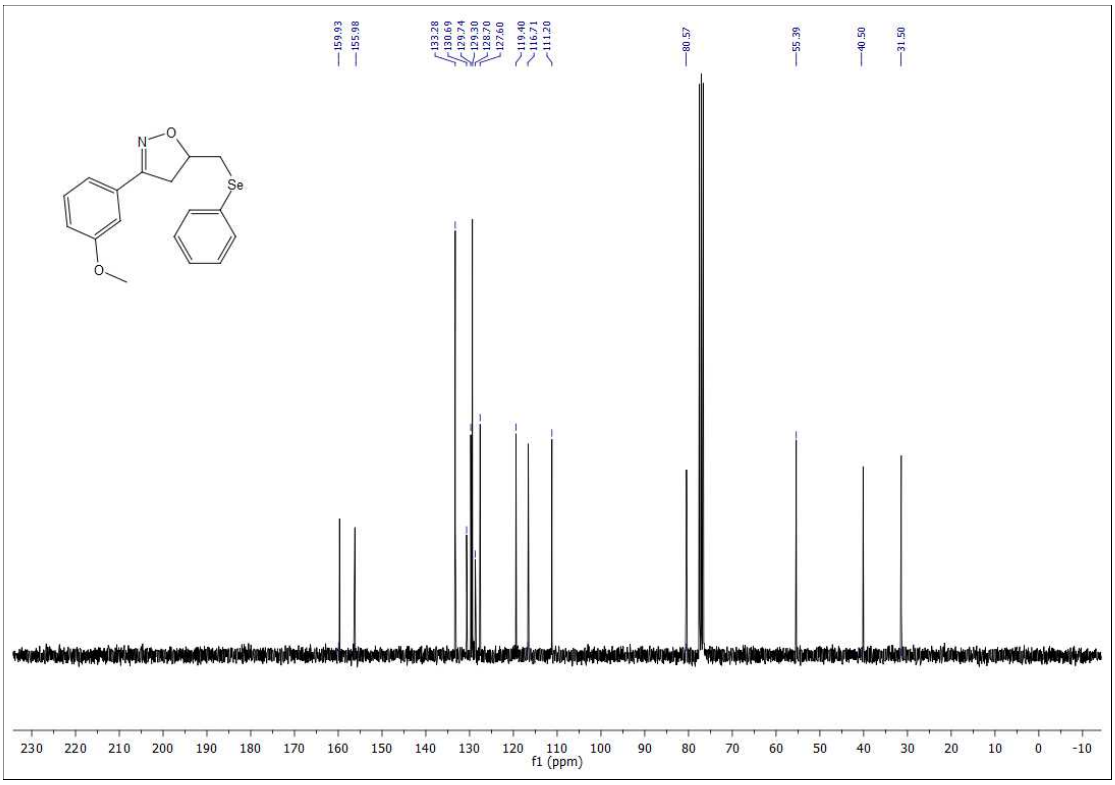

^{1}H NMR (300 MHz, $CDCl_3$) of 2-phenyl-5-((phenylthio)methyl)-4,5-dihydrooxazole (9a)

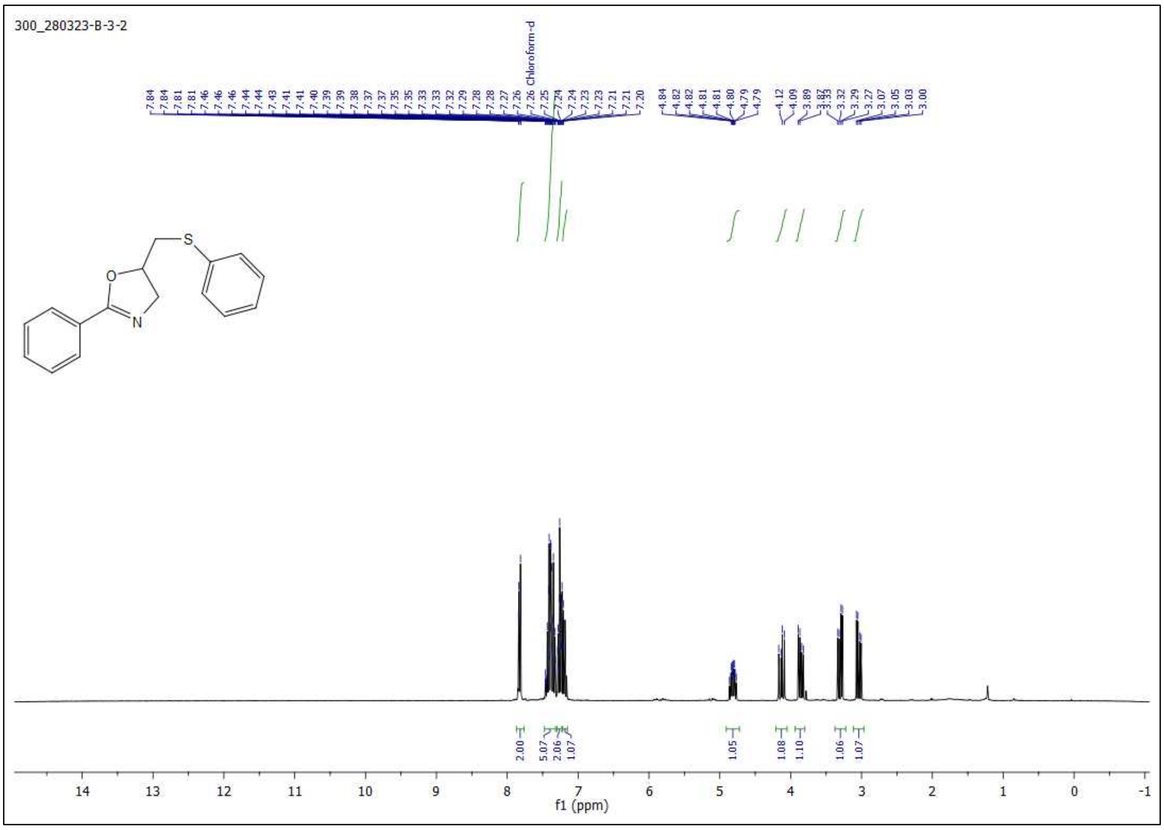

^{13}C {^{1}H} NMR (75 MHz, $CDCl_3$) of 2-phenyl-5-((phenylthio)methyl)-4,5-dihydrooxazole (9a)

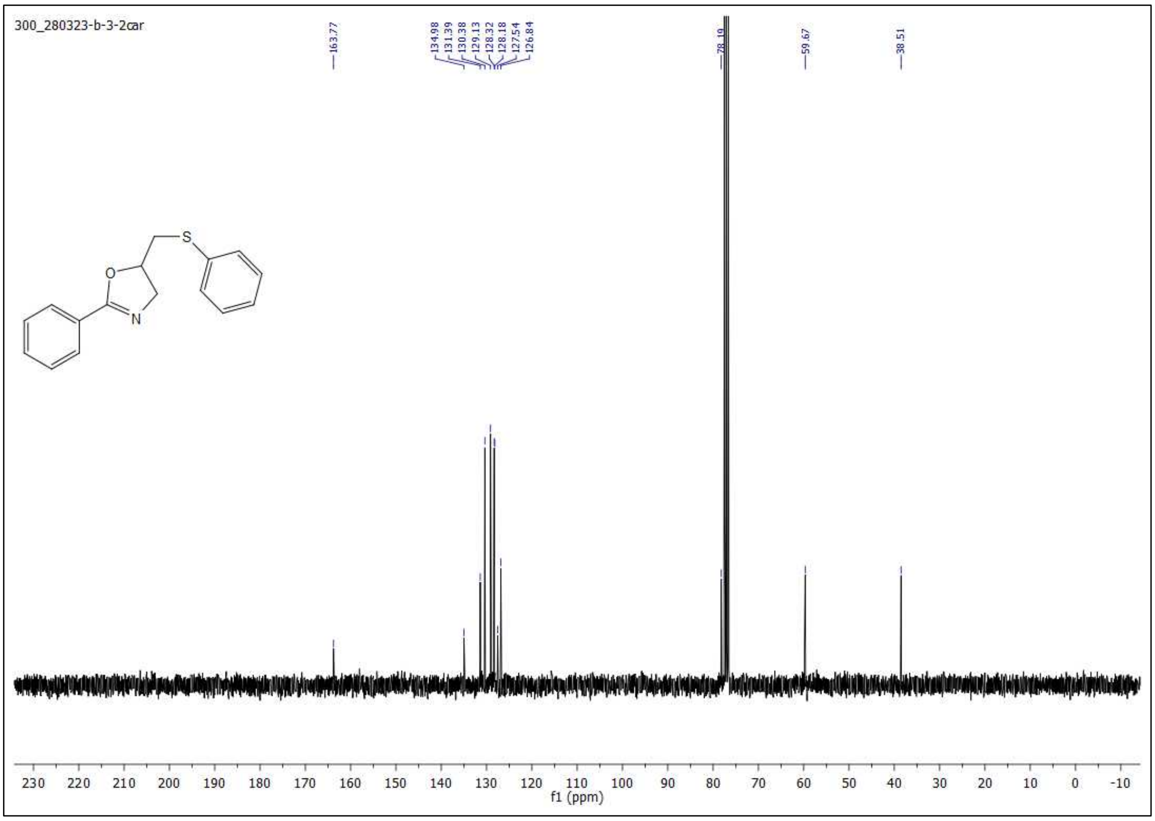

References:

[1] Elsherbini, M.; Moran, W. J. Toward a General Protocol for Catalytic Oxidative Transformations Using Electrochemically Generated Hypervalent Iodine Species. *J. Org. Chem*. **2023**, *88*, 1424−1433.

[2] Gilbert, A.; Bertrand, X.; Paquin, J.-F. Silver-Promoted Synthesis of 5 [(Pentafluorosulfanyl)methyl]-2-oxazolines. *Org. Lett.* **2018**, *20*, 7257−7260.

[3] Abazid, A. H.; Hollwedel, T.-N.; Nachtsheim, B.-J. Stereoselective Oxidative Cyclization of N-Allyl Benzamides to Oxaz(ol)ines. *Org. Lett*. **2021**, *23*, 5076−5080.

[4] Bal, A.; Dinda, T.-K.; Mal, P. Mechanochemical Aliphjatic Iodination (and Bromination) by Cascaded Cyclization. *Asian J. Org. Chem*. **2022**, *11*, e2022000.

[5] Haupt, J.-D.; Berger, M.; Waldvogel, S.-R. Electrochemical Fluorocyclization of N-Allylcarboxamides to 2-Oxazolines by Hypervalent Iodine Mediator. *Org. Lett.* **2019**, *21*, 242–245.

[6] Appukkuttan, P.; Axelsson, L.; Van der Eycken, E.; Larhed, M. Microwave-assisted, $Mo(CO)_6$-mediated, palladium-catalyzed amino-carbonylation of aryl halides using allylamine: from exploration to scale-up. *Tetrahedron Lett*. **2008**, *49,* 5625–5628.

[7] Gross, U.; Koos, P.; O'Brien, M.; Polyzos, A.; Ley, S.-V. A General Continuous Flow Method for Palladium Catalysed Carbonylation Reactions Using Single and Multiple Tube-in-Tube Gas-Liquid Microreactors. *Eur. J. Org. Chem*. **2014**, 6418– 6430.

[8] Nammalwar, B.; Muddala, N.-P; Watts, F.-M. Bunce, R.-A. Efficient conversion of acids and esters to amides and transamidation of primary amides using OSU-6. *Tetrahedron* **2015**, *71*, 9101–9111.

[9] Zhou, M.; Zhao, H-Y.; Zhang, S.; Zhang, Y.; Zhang, X. Nickel-Catalyzed Four-Component Carbocarbonylation of Alkenesunder 1 atm of CO. *J. Am. Chem. Soc.* **2020**, *142*, 18191−18199.

[10] Alhalib, A.; Kamouka, S.; Moran, W.-J. Iodoarene-Catalyzed Cyclizations of Unsaturated Amides. *Org. Lett.* **2015**, *17*, 1453−1456.

[11] Liu, G.-Q.; Yang, C.-H.; Li, Y.-M. Modular Preparation of 5-Halomethyl-2-oxazolines *via* $PhI(OAc)_2$-Promoted Intramolecular Halooxygenation of N-Allylcarboxamides. *J. Org. Chem.* **2015**, *80*, 11339−11350.

[12] Dalling, A.-G.; Yamauchi, T.; McCreanor, N.-G.; Cox, L.; Bower, J.-F. Carbonylative C-C Bond Activation of Electron-Poor Cyclopropanes: Rhodium-Catalyzed (3+1+2) Cycloadditions of Cyclopropylamides. *Angew. Chem. Int. Ed.* **2019**, *58*, 221–225.

[13] Triandafillidi, I.; Kokotos*,* C.-G. Green Organocatalytic Synthesis of Isoxazolines via a One-Pot Oxidation of Allyloximes. *Org. Lett.* **2017**, *19*, 106−109.

[14] Wang, L.-J.; Chen, M.; Qi, L.; Xu, Z.; Li, W. Copper-mediated oxysulfonylation of alkenyl oximes with sodium sulfinates: a facile synthesis of isoxazolines featuring a sulfone substituent. *Chem. Commun.* **2017**, *53*, 2056–2059.

[15] https://www.ika.com/en/Products-Lab-Eq/Electrochemistry-Kit-csp-516/ElectraSyn-20-pro-Package-cpdt-40003261/, (accessed January 2024).

[16] Ion electrochemical reactor – features, https://www.vapourtec.com/products/flow-reactors/ion-electrochemical-reactor-features/Ion electrochemical reactor – features/, (accessed January 2024)

[17] Makhal, P.-N.; Dannarm, S.-R.; Shaikh, A.-S. Sonti, R.-V.; Kaki, R. TBHP-Mediated Selenocyclization of N-Allylbenzamides/Benzthioamides via In-Situ Generation of "PhSeOH" Species. *ChemistrySelect* **2022**, *7*, e202200933.

[18] Zhang, Q.-B.; Yuan, P.-F.; Kai, L.-L.; Liu, K.; Ban, Y.-L.; Wang, X.-Y.; Wu, L.-Z.; Liu, Q. Preparation of Heterocycles via Visible-Light-Driven Aerobic Selenation of Olefins with Diselenides. *Org. Lett.* **2019**, 21, 885−889.

[19] Guan, Z.; Wang, Y.; Wang, H.; Huang, Y.; Wang, S.; Tang, H.; Zhang, H.; Lei, A. Electrochemical oxidative cyclization of olefinic carbonyls with diselenides. *Green Chem.* **2019**, 21, 4976–4980.

[20] Mallick, S.; Baidya, M.; Mahanty, K.; D.; Maiti, Sarkar, S.-De. Electrochemical Chalcogenation of β,γ-Unsaturated Amides and Oximes to Corresponding Oxazolines and Isoxazolines. *Adv. Synth. Catal.* **2020**, 362, 1046–1052.

[21] Zhou, C.-F.; Zhang, Y.-Q.; Ling, Y.; Ming, L.; Xi, X.; Liu, G.-Q.; Zhang, Y. Time-economical synthesis of selenofunctionalized heterocycles via I_2O_5-mediated selenylative heterocyclization. *Org. Biomol. Chem.* **2022**, *20*, 420–426.

[22] Araujo, D.-R.; Lima, Y.-R.; Barcellos, A.-M.; Silva, M.-S.; Jacob, R.-G.; Lenardão, E. J.; Bagnoli, L.; Santi, C.; Perin, G. Ultrasound-Promoted Radical Synthesis of 5-Methylselanyl-4,5dihydroisoxazoles. *Eur. J. Org. Chem.* **2020**, 586–592.

[23] Lopes, E.-F.; Penteado, F.; Thurow, S.; Pinz, M.; Reis, A.-S.; Wilhelm, E.-A.; Luchese, C.; Barcellos, T.; Dalberto, B.; Alves, D.; da Silva, M.-S.; Lenardão, E.-J. Synthesis of Isoxazolines by the Electrophilic Chalcogenation of β , γ -Unsaturated Oximes: Fishing Novel Anti-Inflammatory Agents. *J. Org. Chem*. **2019**, *84*, 12452−12462.

[24] Stein, P.-M.; Rudolph, M.; Hashmi, A. S. K. Water Can Accelerate Homogeneous Gold Catalysis. *Adv. Synth. Catal.* **2021**, *363*, 4264– 4271.
